# Plant polyadenylation factors: conservation and variety in the polyadenylation complex in plants

**DOI:** 10.1186/1471-2164-13-641

**Published:** 2012-11-20

**Authors:** Arthur G Hunt, Denghui Xing, Qingshun Q Li

**Affiliations:** 1 Department of Plant and Soil Sciences, University of Kentucky, Lexington, KY, 40546, USA; 2 Department of Botany, Miami University, Oxford, OH, 45056, USA; 3 Rice Research Institute, Fujian Academy of Agricultural Sciences, Fuzhou, Fujian, 350019, China; 4 Key Laboratory of the Ministry of Education for Coastal and Wetland Ecosystem, and College of the Environment and Ecology, Xiamen University, Xiamen, Fujian, 361102, China

**Keywords:** Polyadenylation, RNA processing, Evolutionary conservation

## Abstract

**Background:**

Polyadenylation, an essential step in eukaryotic gene expression, requires both *cis*-elements and a plethora of *trans-*acting polyadenylation factors. The polyadenylation factors are largely conserved across mammals and fungi. The conservation seems also extended to plants based on the analyses of Arabidopsis polyadenylation factors. To extend this observation, we systemically identified the orthologs of yeast and human polyadenylation factors from 10 plant species chosen based on both the availability of their genome sequences and their positions in the evolutionary tree, which render them representatives of different plant lineages.

**Results:**

The evolutionary trajectories revealed several interesting features of plant polyadenylation factors. First, the number of genes encoding plant polyadenylation factors was clearly increased from “lower” to “higher” plants. Second, the gene expansion in higher plants was biased to some polyadenylation factors, particularly those involved in RNA binding. Finally, while there are clear commonalities, the differences in the polyadenylation apparatus were obvious across different species, suggesting an ongoing process of evolutionary change. These features lead to a model in which the plant polyadenylation complex consists of a conserved core, which is rather rigid in terms of evolutionary conservation, and a panoply of peripheral subunits, which are less conserved and associated with the core in various combinations, forming a collection of somewhat distinct complex assemblies.

**Conclusions:**

The multiple forms of plant polyadenylation complex, together with the diversified polyA signals may explain the intensive alternative polyadenylation (APA) and its regulatory role in biological functions of higher plants.

## Background

Messenger RNA 3’end polyadenylation is an essential step for most of eukaryotic mRNA biogenesis. It requires both *cis*-elements within a pre-mRNA sequence and *trans*-acting factors consisting of dynamic and complicated polyadenylation complexes [1-4]. Although 3’ end cleavage and polyadenylation could be performed with 10-15 proteins *in vitro*, mRNA 3’ end processing is coupled with most steps of mRNA biogenesis *in vivo*, from the initiation of transcription to the export of the mature mRNA [5-7]. Reflecting this, a recent study suggests that more than 80 proteins from different pathways of RNA biogenesis associate with active polyadenylation complexes [8]. More interestingly, studies in recent years also suggest that 3’ end processing could serve as a robust step for regulating gene expression in higher eukaryotes by means of alternative polyadenylation (APA), with which the same gene could produce multiple transcripts with varied stability, special RNA motifs, and coding capacities [9-11]. Indeed, APA was estimated to occur in more than 50% of human genes based on a genome level analysis [11,12]. In *Arabidopsis*, more than 70% of genes have detectable APA sites [13,14]. Similarly, both rice and *Chlamydomonas reinhardtii* (green alga) have extensive APA sites, with the former being 80% and the latter 50% of their genes [15-17]. In genes that possess multiple sites, different patterns of poly(A) site choice are seen in different tissues and different development stages, indicating that APA may be regulated by developmental or environmental cues [9,11,18-20]. While the molecular mechanisms of APA in regulating gene expression are largely unknown, there is evidence that both *cis*-elements and *trans*-acting factors are involved in APA [21-23]. In some tissues, there are isoforms of “canonical” polyadenylation factors functioning in the preferred APAs of those tissues [24-28]. These data support the notion that multiple complexes, which likely share the core factors of polyadenylation machinery, operate in the polyadenylation of subsets of genes in response to different developmental and environmental cues.

Our current understanding of polyadenylation mechanisms is largely from studies in human and yeast. The essential polyadenylation factors and *cis*-elements involved in *in vitro* 3’ end processing have been well defined in these organisms [23]. Towards a better understanding of the molecular and biochemical mechanisms of plant polyadenylation, we have identified *Arabidopsis* proteins similar to the essential yeast and human polyadenylation factors [3]. Based on an array of protein interaction assays, including yeast two-hybrid, *in vitro* pull-down, immunoprecipitation and affinity purification assays, the deduced interaction topology of those *Arabidopsis* polyadenylation factors seems to be similar to those of yeast and human ones [3]. Some *Arabidopsis* (and by extension, higher plants) unique features, however, have been noted [3,16].

In this report, we describe the plant orthologs of yeast and human polyadenylation factors from several representative organisms of the plant lineage. The results reveal several interesting features including the biased expansion of the genes encoding plant polyadenylation factors from “lower” to “higher plants” and variations in the composition of the polyadenylation apparatus across different species. They lend themselves to a model whereby the plant polyadenylation complex is dynamic and amenable to regulation and evolutionary changes.

## Results

### The sets of genes encoding polyadenylation factor subunits vary in different plant genomes

Previously, we identified and characterized *Arabidopsis thaliana* proteins similar to subunits of yeast and human polyadenylation factors [3]. To extend these observations, and to better understand the nature of the polyadenylation apparatus in plants, we identified orthologs of yeast and human polyadenylation factors from several representative organisms of the plant lineage: *Arabidopsis lyrata*, *Glycine max* (soybean), *Vitis vinifera* (grape), *Populus trichocarpa* (poplar), *Oryza sativa* subsp. Japonica (rice), *Sorghum bicolor* (sorghum), *Brachypodium distachyon* (purple false brome), *Selaginella moellendorffii* (lycophyte), *Physcomitrella patens* (moss), and *Chlamydomonas reinhardtii* (green alga)*.* This collection of organisms was selected in part because of the availability of completed genome sequences, and because they represent different aspects of the plant evolution. Thus*, P. patens* and *S. moellendorffii* are representatives of so-called “lower” plants while the “higher” plants are represented by the five dicots including closely-related (and recently-diverged) species (*A. thaliana* and *A. lyrata*), a legume (*G. max*), a tree (P*. trichocarpa*), and three grasses (rice, sorghum and *B. distachyon*). The selected “higher” plants should provide insight into possible differences between monocots and dicots. This collection also spans various of the large-scale genome duplications proposed to have occurred in the evolution of plants [29].

**Figure 1:**
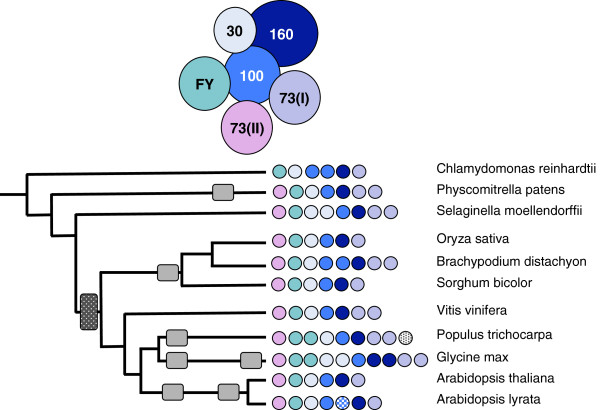
**Complement of genes encoding CPSF subunits in the plant lineage.** The genomes studied in this figure (as well as Figures 2-8) are represented as a phylogenetic tree whose branches are arbitrarily drawn to facilitate the representation. Occurrences of large-scale genome duplications are indicated as rectangular boxes along the phylogenetic tree, and are adapted from Van de Peer et al. [29]. The color coding for each subunit (FY, CPSF160, etc.) is noted above the illustration of the tree. The numbers of genes encoding the various subunits are denoted as small color-coded circles next to each species; hatched circles indicate “partial” proteins. For the CPSF100/CPSF73(I)/CPSF73(II) family, assignments were based on the amino acid sequence alignments summarized in Additional file 2: Figure S1.

**Figure 2:**
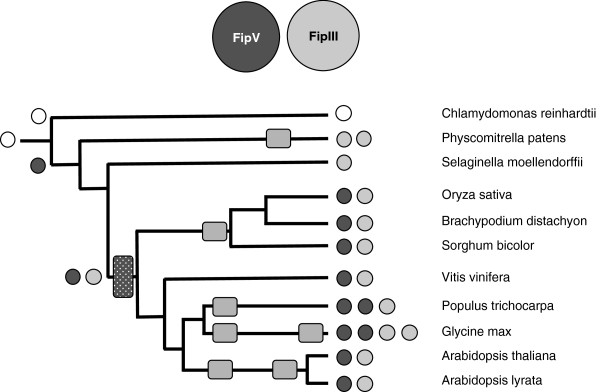
**Complement of genes encoding Fip domain-containing proteins in the plant lineage.** The coding legend and representation are as described for Figure 1; assignments as FIPS5- or FIPS3- like are based on the alignment summarized in Additional file 2: Figure S2.

**Figure 3:**
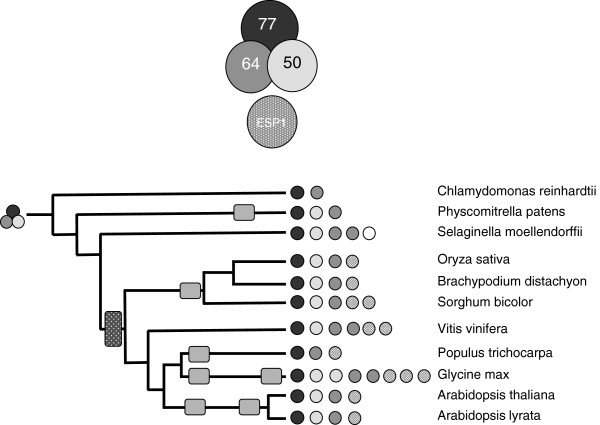
**Complement of genes encoding CstF in the plant lineage.** The coding legend and representation are as described for Figure 1. Assignment of CstF64-related proteins as “complete” (At1g71800-like) or “partial” (ESP1-like) are based on the alignment shown in Additional file 2: Figure S3.

**Figure 4:**
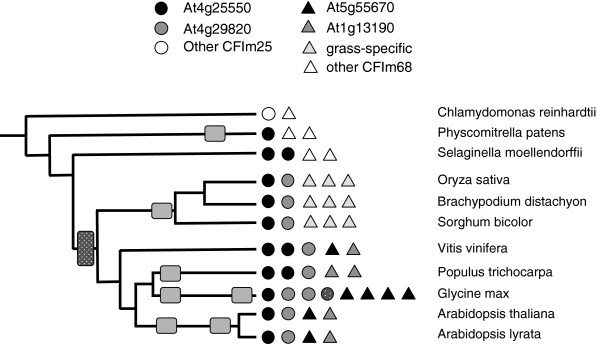
**Complement of genes encoding CFIm subunits in the plant lineage.** The coding legend and representation are as described for Figure 1. Assignments as At4g25550- or At4g29820- like are based on the alignment summarized in Additional file 2: Figure S4. The hatched circle for one of the G. max CFIm25 isoforms indicates a gene that is incompletely annotated and thus not included in the analysis shown in Additional file 2: Figure S4. For CFIm68, assignments were based on the alignment shown in Additional file 2: Figure S5.

**Figure 5:**
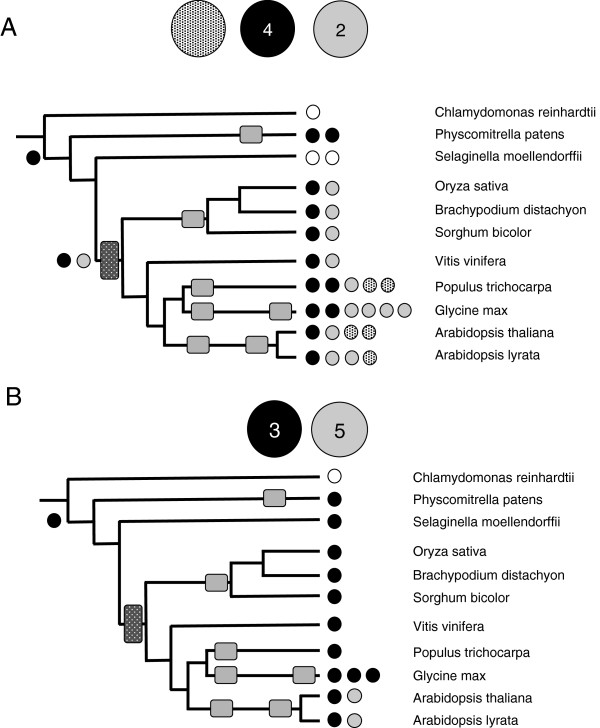
**Complement of genes encoding CFIIm subunits in the plant lineage.** The coding legend and representation are as described for Figure 1. **A**. PCFS subunits. **B**. CLPS subunits. PCFS subclassifications were based on the alignments shown in Additional file 2: Figure S6.

**Figure 6:**
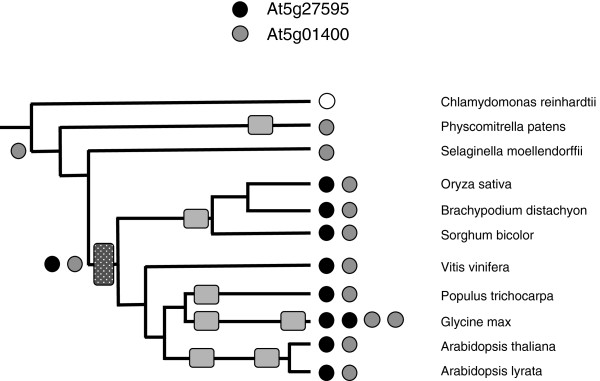
**Complement of genes encoding symplekin in the plant lineage.** The coding legend and representation are as described for Figure 1. Assignment as At5g01400- or At5g27595- like were based on the alignment shown in Additional file 2: Figure S7.

**Figure 7:**
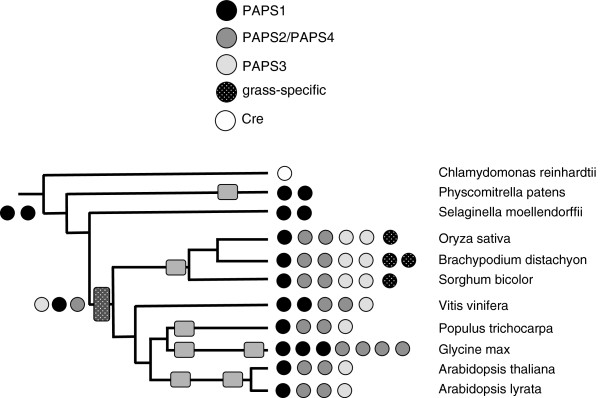
**Complement of genes encoding poly(A) polymerases in the plant lineage.** The coding legend and representation are as described for Figure 1. Subclassifications were based on the alignment shown in Additional file 2: Figure S8.

**Figure 8:**
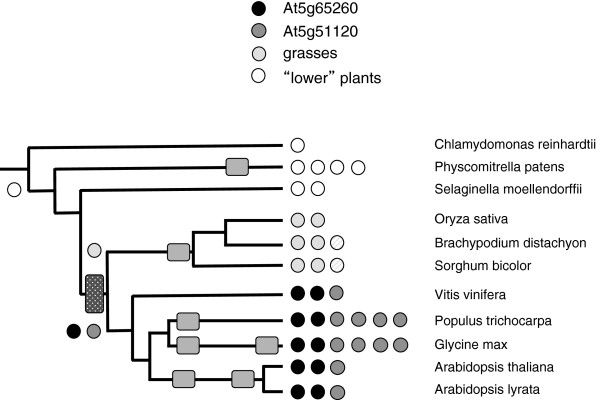
**Complement of genes encoding nuclear poly(A) binding proteins in the plant lineage.** The coding legend and representation are as described for Figure 1. Subclassifications were based on the alignment shown in Additional file 2: Figure S11.

The orthologs so identified are listed in Additional file 1: Table S1. The number of genes encoding polyadenylation factor subunit orthologs was greater in higher plants than in *S. moellendorffii*, *P. patens* and *C. reinhardtii* (Table 1). The total number of such genes in higher plants ranged from 30 to 56, a range that probably reflects episodic large-scale duplications along with instances of gene loss. *S. moellendorffii* and *P. patens* possessed 25 and 26 such genes, while only 16 such genes could be found in the *C. reinhardtii* genome (Table 1). The gene complements for each species are illustrated in Figures 1, 2, 3, 4, 5, 6, 7, 8 and described in more detail in the following section.

**Table 1:** Numbers of genes that encode polyadenylation factor subunits

**Species**	**CstF**	**CPSF**	**PAP**	**PABN**	**Fip1**	**CFIm**	**CFIIm**	**Symplekin**	**total**
*C. reinhardtii*	2	6	1	1	1	2	2	1	16
*P. patens*	3	7	2	4	2	3	3	1	25
*S. moellendorffii*	5	8	2	2	1	4	3	1	26
*O. sativa*	4	6	6	2	2	5	3	2	30
*B. distachyon*	4	8	7	3	2	5	4	2	35
*S. bicolor*	5	6	6	3	2	5	3	2	32
*A. thaliana*	4	6	4	3	2	4	6	2	31
*A. lyrata*	4	7	4	3	2	4	6	2	32
*G. max*	8	10	7	6	4	8	9	4	56
*P. trichocarpa*	3	9	4	6	3	5	6	2	38
*V. vinifera*	6	7	5	2	2	5	4	2	33

### The complement of protein subunits and their encoding genes

#### Cleavage and Polyadenylation Specificity Factor (CPSF)

In mammals, the canonical CPSF complex consists of four subunits of 160, 100, 73, and 30 kD. In plants, another subunit (typified by the protein encoded by At2g01730), related to CPSF73 as well as subunit 11 of the Integrator complex (Additional file 2: Figure S1), is recognized as a CPSF subunit, based on the copurification of this protein with other CPSF subunits [30,31]. FY (the ortholog of the yeast Pfs2p protein) is also recognized as a part of this complex based on the same criteria [31,32]. For the most part, in plants, there are single genes that encode each of the six subunits of CPSF (Figure 1). The exceptions to this are the duplicates for CPSF160, CPSF30, and FY in *G. max* (duplicates that probably arose via a recent genome duplication event), the different numbers of CPSF73(I) genes that are seen in different species, and a partial CPSF100 gene in *A. lyrata*. Interestingly, while *C. reinhardtii* possesses three genes that encode metallo-β-lactamase domain proteins similar to CPSF73 or CPSF100, it lacks a probable ortholog of the product of the Arabidopsis At2g01730 gene (Figure 1, Additional file 2: Figure S1).

Another subunit that is considered a part of the CPSF complex in mammals is the hFip1 protein. In *C. reinhardtii*, and *S. moellendorffii*, there exists a single gene encoding Fip1 orthologs while *P. patens* possess two such orthologs (Figure 2, Additional file 1: Table S1). These orthologs are identifiable by the presence of a conserved domain (PF05182); outside of this domain, these proteins diverge significantly from each other and from animal and yeast Fip1 orthologs (Additional file 2: Figure S2, Additional file 3). The flowering plant lineages possess two distinct gene families whose protein products are related to Fip1 (Additional file 2: Figure S2). These genes are typified by the *Arabidopsis* At5g58040 gene, that encodes a well-characterized protein with substantial biochemical similarity with the human Fip1 ortholog [33], and the At3g66652 gene (Figure 2, Additional file 2: Figure S2).

#### Cleavage Stimulatory Factor (CstF)

With few exceptions, the three subunits of the CstF complex are encoded by single genes in plants (Figure 3). CstF50 and CstF64 (but not CstF77) are encoded by two genes in *G. max*, similar to what is seen with CPSF160, CPSF30, and FY. In addition, *S. moellendorfii* possesses two genes that encode probable CstF64 orthologs.

An addition class of protein that is related to CstF64 is also found in higher plants as well as *S. moellendorfii*; this protein is typified by the product of the *Arabidopsis* At1g73840 gene. This protein lacks the RRM domain found in full-sized CstF64 proteins [34] but retains domains responsible for interactions with CstF77 and with factors that mediate transcription termination. Higher plants have between one and three At1g73840-like genes that form a separate clade in amino acid sequence alignments (Additional file 2: Figure S3). Interestingly, *S. moellendorfii* also possesses a gene whose product retains the CstF77- and termination factor- interacting domains but lacks the RRM motif found in canonical CstF64 proteins. However, in sequence alignments, this truncated protein aligns more closely with the full-sized *S. moellendofrii* CstF64 orthologs than with the At1g73840-like proteins (Additional file 2: Figure S3). *P. patens* and *C. reinhardtii* lacks these truncated CstF64-like proteins.

Curiously, no obvious CstF50 orthologs could be seen in the *C. reinhardtii* or *P. trichocarpa* genomes (Figure 3). This is true even when TBLASTN is used to mine the respective genomes, ruling out the possibility that these genes have not yet been annotated (and thus included in the sets of proteins encoded by the respective genomes). This observation raises the possibility that CstF50 may be dispensable for 3’ end processing in plants that possess the protein.

#### Cleavage Factor I (CF-Im)

In mammals, CFIm is a heteromeric complex that consists of a larger and smaller subunit [35-38]. The two lower plants possess a single family of genes encoding the smaller subunit (CFIm25), while the higher plants possess two CFIm25 gene families typified by the *Arabidopsis* At4g25550 and At4g29820 genes, respectively (Figure 4). The *P. patens* CFIm25 ortholog protein bears a closer resemblance to the At4g25550-encoded protein (Additional file 2: Figure S4).

Sequence alignments reveal that at least four distinct classes of the larger subunit of CFIm (termed here as CFIm68) can be found in plants (Figure 4, Additional file 2: Figure S5). One of these is specific to grasses, two are found in eudicots, and one is a collection of more distantly-related polypeptides, found in *C. reinhardtii*, *P. patens*, and *S. moellendorffii*, that cannot be clearly associated with any of the higher plant isoforms.

#### Cleavage factor II (CF-IIm)

Two subunits, the orthologs of yeast Pcf11p and Clp1p, constitute CFII in mammals. *C. reinhardtii*, *P. patens*, and *S. moellendorffii* all possess genes encoding Pcf11 (termed PCFS). Higher plants possess two gene families that encode PCFS, typified by the *Arabidopsis* At4g04885 and At2g36480 genes (Figure 5A, Additional file 2: Figure S6A). The At4g04885-encoded protein possesses similarities to two of the three functional domains (the RNA polymerase II-terminal interacting domain, or CID, and the Clp1-interacting domain) of Pcf11 (Additional file 4), with little or no sequence similarity in the domain reported to function in the interaction of Pcf11 with RNA14/RNA15. The At2g36480-encoded protein lacks part of the N-terminal CID and also any noticeable similarity with the RNA14/RNA15-interacting domain of Pcf11. However, an adjacent gene located 5’ of At2g36480 (At2g36485) encodes the “missing” section of the CID. The homologous rice gene (Os09g39270) is a fusion of the two *Arabidopsis* genes; there is EST support for a single transcript from the rice gene (Additional file 2: Figure S6B). At the present, no such support can be found in *Arabidopsis* EST collections or after mapping of more than 170 million RNA-Seq reads (A. G. Hunt, unpublished observations).

Some higher plants possess additional genes whose encoded proteins are somewhat similar to the Clp1-binding C-termini of those encoded by At4g04885 and At2g36480 (Figure 5A, Additional file 2: Figure S6A, Additional file 4); these proteins are typified by the products of the *Arabidopsis* At1g66500 and At5g43620 genes. The occurrence of these is somewhat sporadic; they are not seen in the grasses or in two of the five eudicots in the study (*V. vinifera* and *G. max*).

Another CFIIm subunit is the Clp1 protein. For the most part, plants possess single genes encoding this subunit (Figure 5B); the exceptions are *G. max* (that possesses three closely-related genes) and *A. thaliana* and *A. lyrata* (that each possess two genes that encode different CLPS isoforms). One class of *Arabidopsis* CLPS isoform (At3g04680) is related to the other plant CLPS proteins, while the other *Arabidopsis* isoform (At5g39930) is less similar.

#### Symplekin

*P. patens* and *S. moellendorffii* both possess single symplekin genes whose protein products resemble the protein encoded by the *Arabidopsis* AT5g01400 gene (Figure 6, Additional file 1: Table S1, Additional file 2: Figure S7). In flowering plants, there is a second class of symplekin gene, typified by the *Arabidopsis* At5g27595/At5g27590 gene (Figure 6, Additional file 2: Figure S7), which seems originated from an intact symplekin gene being split by an intergenic region. The “split” nature of the At5g27595/At5g27590 gene has been noted before [34]; other higher plant orthologs do not share this organization.

#### Poly(A) polymerse (PAP)

*C. reindardtii*, *P. patens*, and *S. moellendorfii* all possess relatively simple PAP gene families (Figure 7, Additional file 2: Figure S8), with each organism possessing either a single isoform or two closely-related isoforms. In contrast, flowering plants possess an expanded suite of PAP genes that can be sorted into four families (Figure 7, Additional file 2: Figure S8). One of these families (termed PAPS1, typified by the product of the *Arabidopsis* AT1g17980 gene) is similar to the *P. patens* and *S. moellendorffii* PAP proteins (Additional file 2: Figure S8). One of the two additional families is typified by the *Arabidopsis* At2g25850 and At4g32850 genes; like At1g17980, these encode nucleus-localized proteins [39]. With the exception of *G. max*, flowering plants possess two isoforms or paralogs of this second class of PAP (Figure 7, Additional file 2: Figure S8); *G. max* possesses four possible PAPS2/4-encoding genes. Again with the exception of *G. max*, one or two copies of a gene that encodes a cytoplasmic form of PAP (corresponding to the *Arabidopsis* At3g06560 gene product) can be seen in the flowering plants, but not in *P. patens* or *S. moellendorffii* (Figure 7). These three classes of PAP are seen in all flowering plants. The three grass species possess an additional family of genes that may encode PAPs (Figure 7). As is the case with the PAPS3 family, these PAPs appear to lack nuclear localization signals (Additional file 2: Figure S9). While all of the other higher plant PAPS genes share a common intron-exon organization [39], this grass-specific family either lacks introns or possesses but a single intron whose position is not conserved in other PAPS genes (Additional file 2: Figure S10; [39]). All of the predicted proteins seem to possess a functional catalytic site (Additional file 2: Figure S9), but one of the *B. distachyon* isoforms has a deletion near the N-terminus of the putative primer-binding site.

#### Poly(A) Binding Protein – nuclear (PABN)

Perhaps the most fascinating family of genes is that encoding plant PABN subunits (Figure 8, Additional file 2: Figure S11). *P. patens* possesses four PABN genes that form a separate clade in amino acid sequence alignments (Additional file 2: Figure S11). *S. moellendorffii* possesses two PABN genes that are distinct from the *P. patens* genes and those seen in higher plants. The grass PABN genes form yet another distinct group; interestingly, there seems to have been a duplication early in the evolution of monocots, yielding two sub-groups of monocot-specific PABN isoforms (Additional file 2: Figure S11). There are two groups of eudicot PABN isoforms as well (Additional file 2: Figure S11). One member of this group is typified by At5g51120, while the others are represented by At5g65260 and At5g10350 and form a distinct clade.

### Novel organization of genes encoding plant polyadenylation factor subunits

For some of the genes that encode plant polyadenylation factor subunits, novel or unusual gene organizations were seen. With the exception of *C. reinhardtii*, all of the plant CPSF30 genes possess the novel architecture seen in *Arabidopsis* (illustrated in Additional file 2: Figure S12A). Thus, the plant CPSF30 genes encode proteins with three CCCH-type zinc fingers and an extended domain that bears similarity to the so-called YTH domain reported first in neuronal splicing factors [40,41]. This domain is similar to one (the so-called ECT domain) found in a family of *Arabidopsis* proteins that interact with calcineurin B-Like-Interacting Protein Kinases [42]. In *Arabidopsis*, CPSF30-encoding mRNAs are alternatively processed, such that two proteins are produced. One of these consists just of a 250 amino acid polypeptide that includes the three zinc finger motifs but lacks the YTH domain. The larger consists of the CPSF30-YTH protein. Besides *Arabidopsis*, there is EST evidence for a similar alternative processing in *G. max* (Additional file 2: Figure S12B).

In *Arabidopsis*, a number of other genes that encode polyadenylation factor subunits exhibit a similar sort of alternative polyadenylation, in which some transcripts end within upstream introns (Additional file 2: Figure S13). This is seen with FIPS5, one symplekin isoform (ESP4), CstF77, and one of the two CFIm25 isoforms (At4g25550). There is EST support for similar alternative processing of FIPS5 transcripts in poplar, soybean, rice, and *Brachypodium* (Additional file 5).

In *Arabidopsis*, one of the two symplekin isoforms is encoded by a split gene, At5g27595/At5g27590 [34]. A similar situation is evident with one of the *Arabidopsis* Pcf11 orthologs; specifically, At2g36480 encodes a polypeptide that lacks the very N-terminus of the Pcf11-related polypeptide PCFS2, while the adjacent gene At2g36485 encodes the corresponding N-terminal segment (Additional file 2: Figure S14). Analysis of *Arabidopsis* EST sequences as well as high-throughput poly(A) tag data [14] indicates that the upstream gene encodes mRNAs that are polyadenylated so as to yield the short mRNA and thus predicted N-terminal segment (Additional file 2: Figure S14). Further analysis of RNA-Seq data failed to identify sequence tags that span the two genes (A. G. Hunt, unpublished results). Most of the other eudicot PCFS2-like genes are annotated as “split”, but none of the grass PCFS2-like genes are.

## Discussion

For the most part, the results described in this report indicate a broad evolutionary conservation of the polyadenylation complex, with plants possessing identifiable orthologs of all of the core mammalian polyadenylation factor subunits. However, there are interesting aspects of the sets of genes that encode these subunits in plants. Thus, of the sixteen identifiable orthologs of the subunits of the core mammalian polyadenylation complex, seven are encoded by more than one gene in at least one of the plant species studied. (Note that, for the sake of this discussion, the apparent duplication of virtually all genes in *G. max* is not considered, nor is the *Arabidopsis*-specific CLPS5 gene.) The subunits encoded by single genes are orthologs of the core subunits of CPSF and CstF. With two exceptions (CPSF73-I and Fip1), subunits encoded by expanded gene families reside in other factors in mammals (e.g., CFIm and CFIIm) or they play roles in the last step of the process (poly(A) tail addition and poly(A) length control). The degrees and evolutionary timing of expansion of the various gene families vary greatly, ranging from events that involved but one lineage (e.g., CPSF73-I, CLPS5) to those that occurred before the divergence of the higher plant lineages, but after the divergence of higher plants from *Selaginella*.

These considerations lend themselves to a model where the plant polyadenylation complex consists of a core (consisting of the CPSF and CstF subunits) that is rather rigid in terms of evolutionary conservation, and an associated panoply of peripheral subunits. These peripheral subunits likely do not all exist in a single large, monolithic complex, but rather associate in various and sundry combinations with the CPSF/CstF core; this is because many of the peripheral subunits are isoforms of other subunits and likely interact with the same site(s) of the CPSF/CstF core, and thus are expected to assemble in mutually-exclusive manners. Therefore, the polyadenylation complex may actually be a collection of somewhat distinct assemblies, each with different representatives of the products of the gene families. Such a complex would be amenable to considerable evolutionary and physiological flexibility. Different combinations of peripheral subunits may play dominant roles at special times during development, or in response to stresses. While not exactly analogous, this suggestion brings to mind the specialized functioning of the male-specific CstF64 and PAP isoforms in mammals [43-47].

This model may help to explain some of the poorly-understood features of the plant polyadenylation signal. This signal consists of three distinct *cis*-elements, none of which can be defined by a highly-conserved sequence [48,49]. Of the eight protein subunits that are encoded by gene families in plants, at least four (CFIm25, CFIm68, FIPS, and PABN) are RNA-binding proteins. If the different members of these families encode proteins with somewhat different RNA sequence preferences, the sum of these preferences might be a degenerate, poorly-defined consensus. The sequence characteristics of the three *cis*-elements that have been defined by experimental and computational work would reflect a sum of the preferences of the individual RNA-binding isoforms.

This model also has ramifications for possible mechanisms of alternative poly(A) site choice in plants. For example, in mammals, PABN has been implicated in the differential recognition of weak poly(A) signals that are often associated with promoter-proximal poly(A) sites in genes whose de-regulation is associated with oncogenic transformation [26]. There is but a single PABN isoform in mammals; in contrast, plants possess several potential isoforms (Figure 8). This raises the possibility that different sub-complexes may possess different PABN isoforms, and that differential poly(A) site choice would be accomplished by the action of sub-complexes of different PABN composition. The *Arabidopsis* CPSF30 protein is inhibited *in vitro* by calmodulin and by sulfhydryl reagents [50,51]. Should similar effects be manifest inside cells, then this protein should be inactivated in response to various stimuli. The possibility that the CPSF complex may be of variable composition, with CPSF30-independent configurations, would explain why polyadenylation could continue under such circumstances, and is consistent with a role for CPSF30 in alternative poly(A) site choice mediated by differential inactivation of the protein. Compositional variability would lend itself to additional modes of regulated poly(A) site choice through the directed activation or inactivation of specific subunit isoforms. While little is known about this possibility in plants, mammalian orthologs of plant subunits encoded by gene families are known to be subject to modification by phosphorylation, SUMOylation, ubiquitination, and arginine methylation [52].

An additional layer of complexity in the plant polyadenylation complex is provided by the existence of “partial” protein isoforms, either through alternative RNA processing (as with CPSF30, FIPS, CstF77, symplekin, and CFIm-25; Additional file 2: Figure S13) or coding by separate genes (such as with ESP1 and two of the PCFS variants). These partial proteins possess some of the functionalities of their respective “complete” proteins, but not others; as such they may serve to affect the functioning of other subunits and thus redirect a subcomplex towards a subset of pre-mRNA targets.

Finally, it is noteworthy that, while conserved for the most part, the lower number and distinguishable sequence divergence of *C. reinhardtii* polyadenylation factors sets *C. reinhardtii* apart from the rest of the plant linage. Interestingly, it has been demonstrated that *C. reinhardtii* and other green algae use a different set of poly(A) signals where the UGUAA motif in the near upstream elements is prevalent (found in 52% of the genes) over any other signals [16]. It is probable that the differences in polyadenyation factors contribute to the difference in poly(A) signals, but it is difficult to pinpoint a single subunit as being responsible for the differences (because so many *C. reinhardtii* subunits are noticeably different from their higher plant counterparts). Further experiments are needed to test this hypothesis.

## Conclusions

To summarize, the results presented here reveal both evolutionary conservation and novelty in the plant polyadenylation complex. They indicate that the subunit composition of the plant complex has undergone expansion (probably via gene duplication) in the course of evolution, and that this expansion may have introduced much of this novelty. Together, the data support a model whereby the plant polyadenylation complex consists of a relatively constant core and numerous combinations of peripheral subunits, such that the complex as a whole is actually a population of many different assemblies, which might explain the highly degenerate nature of the plant poly(A) signals.

## Methods

### Ortholog identification

To identify plant orthologs of polyadenylation factor subunits, the genome sequences of these organisms were screened with BLASTP and TBLASTN [53,54] using the amino acid sequences of *A. thaliana* polyadenylation factor subunits [3,25] as queries. The first screen utilized BLASTP to identify orthologs in the respective proteomes. This was supplemented with searches using TBLASTN to identify the corresponding genomic regions, and to find additional sequences that are missing from the protein databases due to incorrect identification of protein- and mRNA- encoding regions. In these screens, no effort was made to sort out possible pseudogenes since these become apparent in the larger comparative studies performed (see the following). Finally, the resulting amino acid sequences were aligned with EXPRESSO3D or TCOFFEE (the former was used when structural information could be applied to the alignments, otherwise the latter was used [55,56]). The results were then used to sort the sequence collections into sub-families, and to otherwise derive a hypothetical evolutionary trajectory for the various polyadenylation factor subunits. The amino acid sequences and their GI or accession numbers are provided in Additional file 6.

### Analysis of high throughput poly(A) tags

High throughput poly(A) tags prepared from wild-type *Arabidopsis* leaf tissue [14] were mapped onto a set of reference sequences that consisted of the genomic sequences of all of the *Arabidopsis* genes described here and listed in Additional file 1: Table S1; for this, the CLC Genomics Workbench was used (http://www.clcbio.com/products/clc-genomics-workbench. CLC bio, Aarhus, Denmark). The results were saved as graphics files and used as shown in the relevant figures in this study.

### Other bioinformatics methods

Representations of various genes and EST data were obtained using the gBrowse link embedded in the gene pages at the Phytozome 8.0 web site [57].

## Competing interests

The authors declare that they have no competing interests.

## Authors’ contributions

AGH, DX and QQL were responsible for the strategy, data interpretation and writing the manuscript. AGH and DX did most of the data collection and analysis. All authors read and approved the final manuscript.

## Supplementary Material

Additional file 1 **Table S1.** Designations for the genes described in this study. Click here for file

Additional file 2 **Figures S1 to S13.** This file contains all the Supplemental Figures including the phylogenetic trees, gene structure, EST and/or sequencing evidence of APA. Click here for file

Additional file 3 **Sequence alignment of Fip1 orthologs.** This file contains the sequence alignment of Fip1 orthologs, showing their significant divergence across regions other than the conserved domain (PF05182). Click here for file

Additional file 4 **The domain conservation of Pcf11 orthologs.** The file contains the figure showing the sequence similarities of different orthologs around the three functional domains. Click here for file

Additional file 5 **Alternative processing of FIPS5.** This file contains the EST support for alternative processing of FIPS5 transcripts in poplar, soybean, rice, and *Brachypodium*. Click here for file

Additional file 6 This file contains all the sequences used to derive the phylogenetic trees in this paper. Click here for file
