# Supplementary material for: Plant polyadenylation factors: conservation and variety in the polyadenylation complex in plants
Source: BMC Genomics. 2012 Nov 20;13:641. doi: 10.1186/1471-2164-13-641 (PMC3538716; doi:10.1186/1471-2164-13-641)
Supplement: Additional file 2 — Figures S1 to S13. This file contains all the Supplemental Figures including the phylogenetic trees, gene structure, EST and/or sequencing evidence of APA. [file 1471-2164-13-641-S2.pdf]

Figure S1

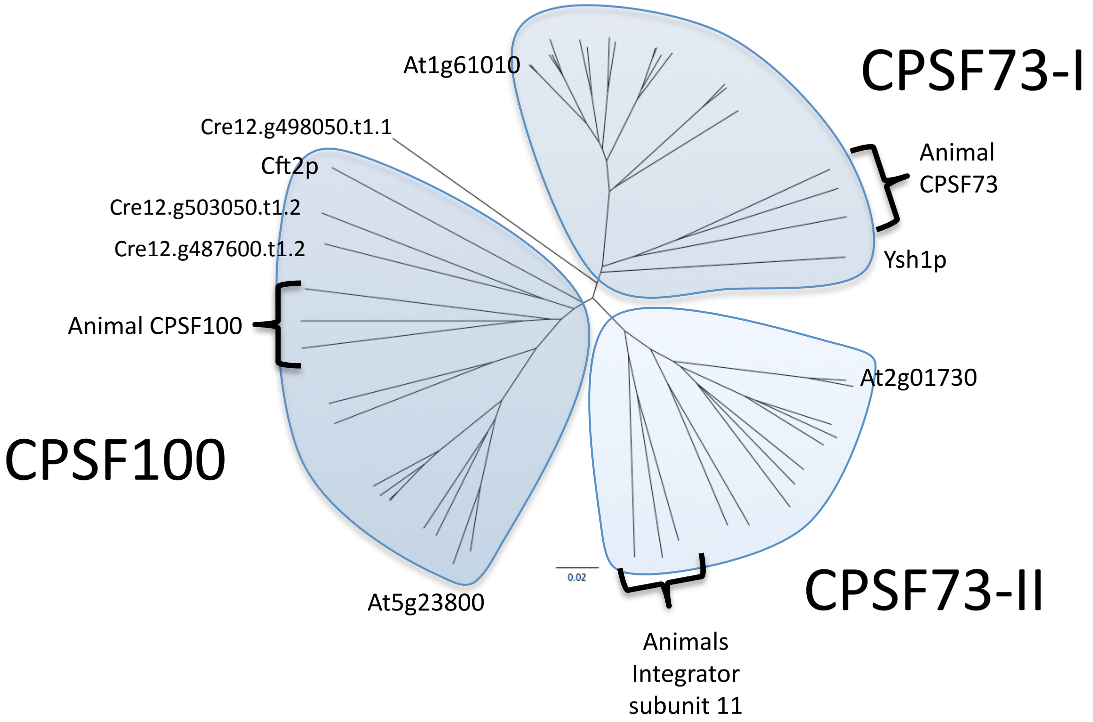

Figure S1. Tree showing the results of amino acid alignments of metallo-beta-lactamase-related CPSF subunits in plants. The three classes of proteins (CPSF100, CPSF73(I), and CPSF73(II)) are set apart with shaded balloons, and the affinities of selected proteins indicated. Labeled branches indicate the relationships of the three Chlamydomonas proteins (“Cre…), animal CPSF and related subunits, and the yeast metallo-beta-lactamase subunits of CPF (Cft2p and Ysh1p). Unlabeled branches represent plant proteins. The sequences used to generate this tree are provided in Supplemental File 4.

Figure S2

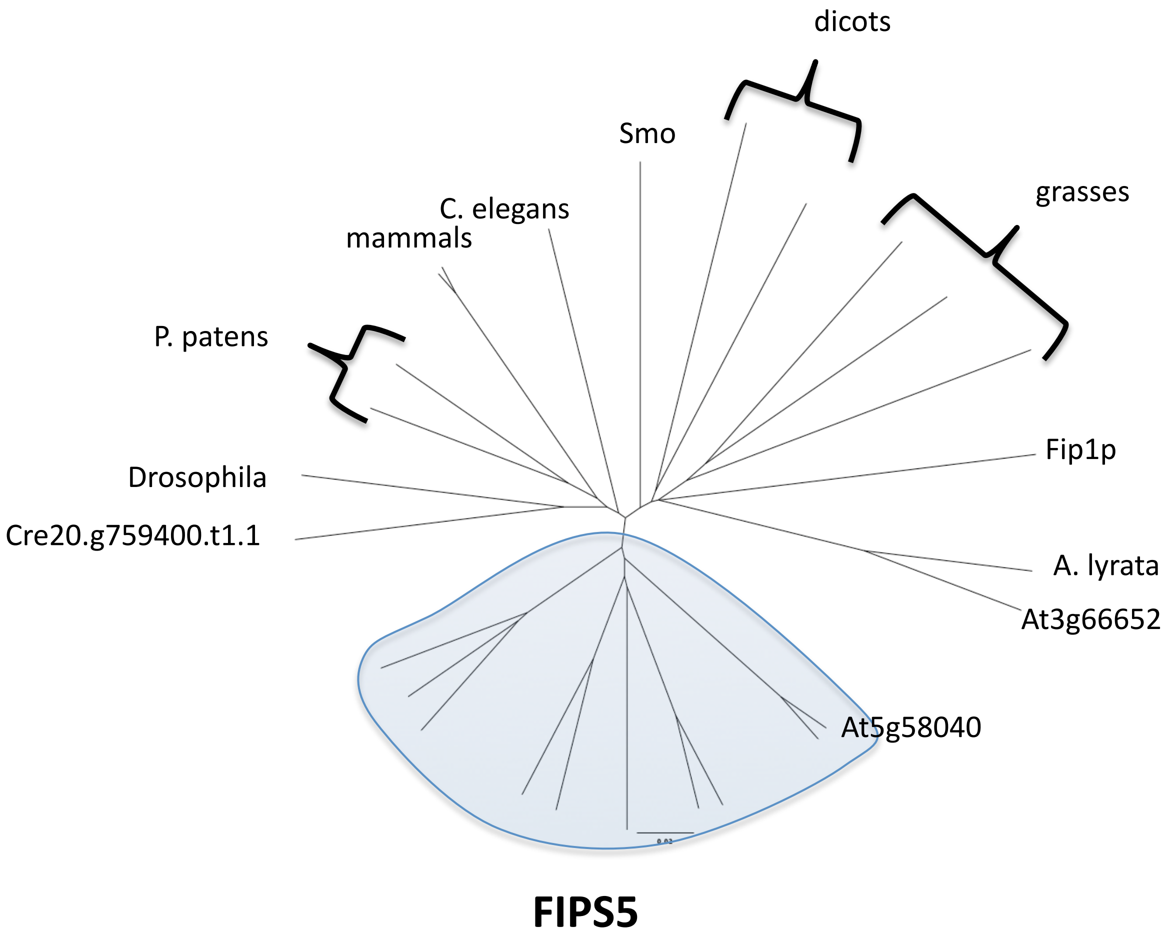

Figure S2. Tree showing the results of amino acid alignments of Fip domain-containing proteins in plants. The FIPS5-like plant proteins (related to At5g58040) are shaded, and the identities of the unshaded branches noted. Proteins whose annotated sequences are not complete were not included in this alignment, but are included in the summation shown in Figure 2. Note the longer branch lengths for the unassigned orthologs compared with those in the FIPS5 family. The sequences used to generate this tree are provided in Supplemental File 4.

Figure S3

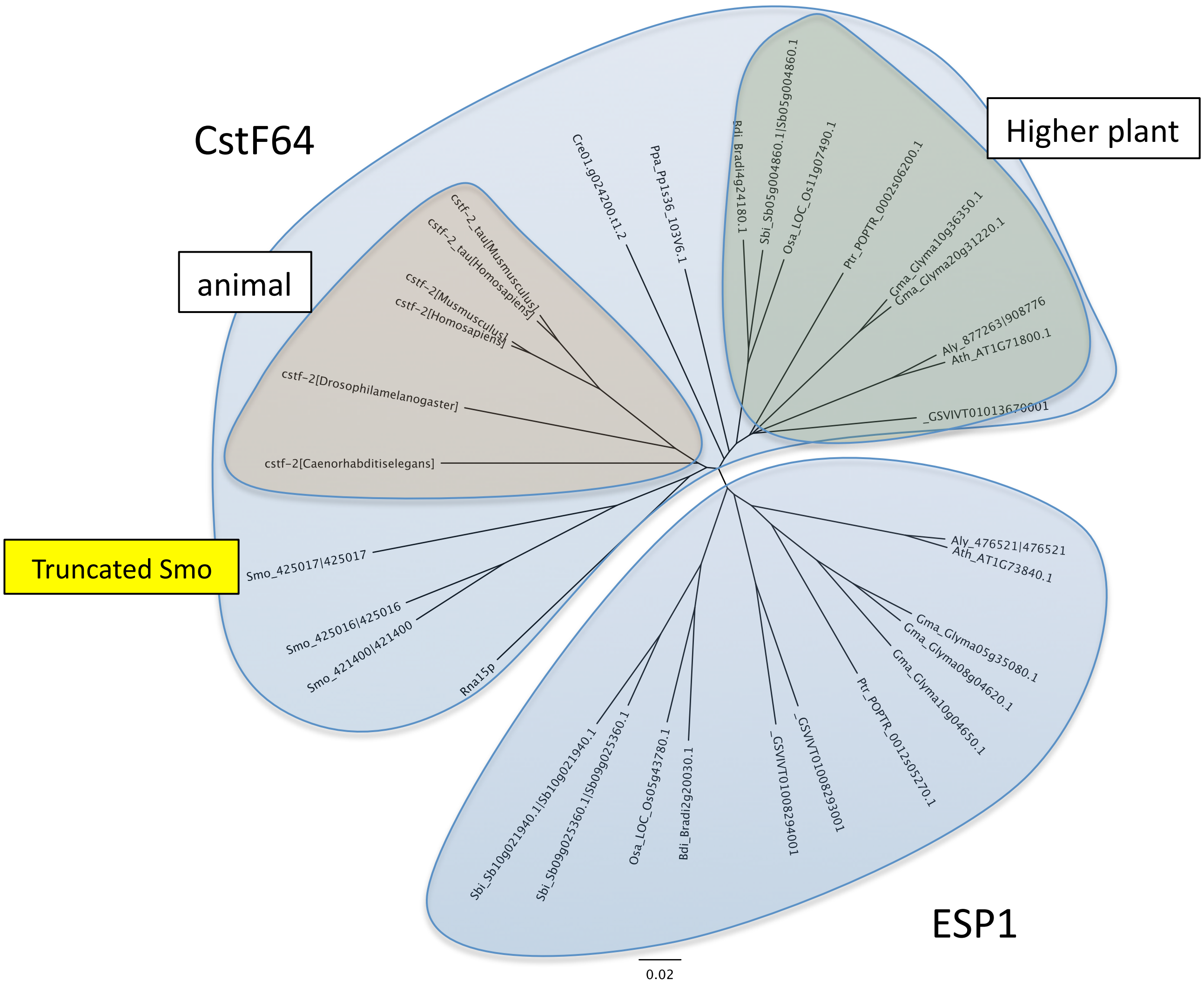

Figure S3. Tree showing the results of amino acid alignments of CstF64-related proteins in plants. The two classes of protein, CstF64- and ESP1- like, are set apart with shaded balloons, and subsets of CstF64-related proteins (animal and higher plant) also set apart. The novel S. moellendorfii CstF64-related protein that is truncated is highlighted. The sequences used to generate this tree are provided in Supplemental File 4.

Figure S4

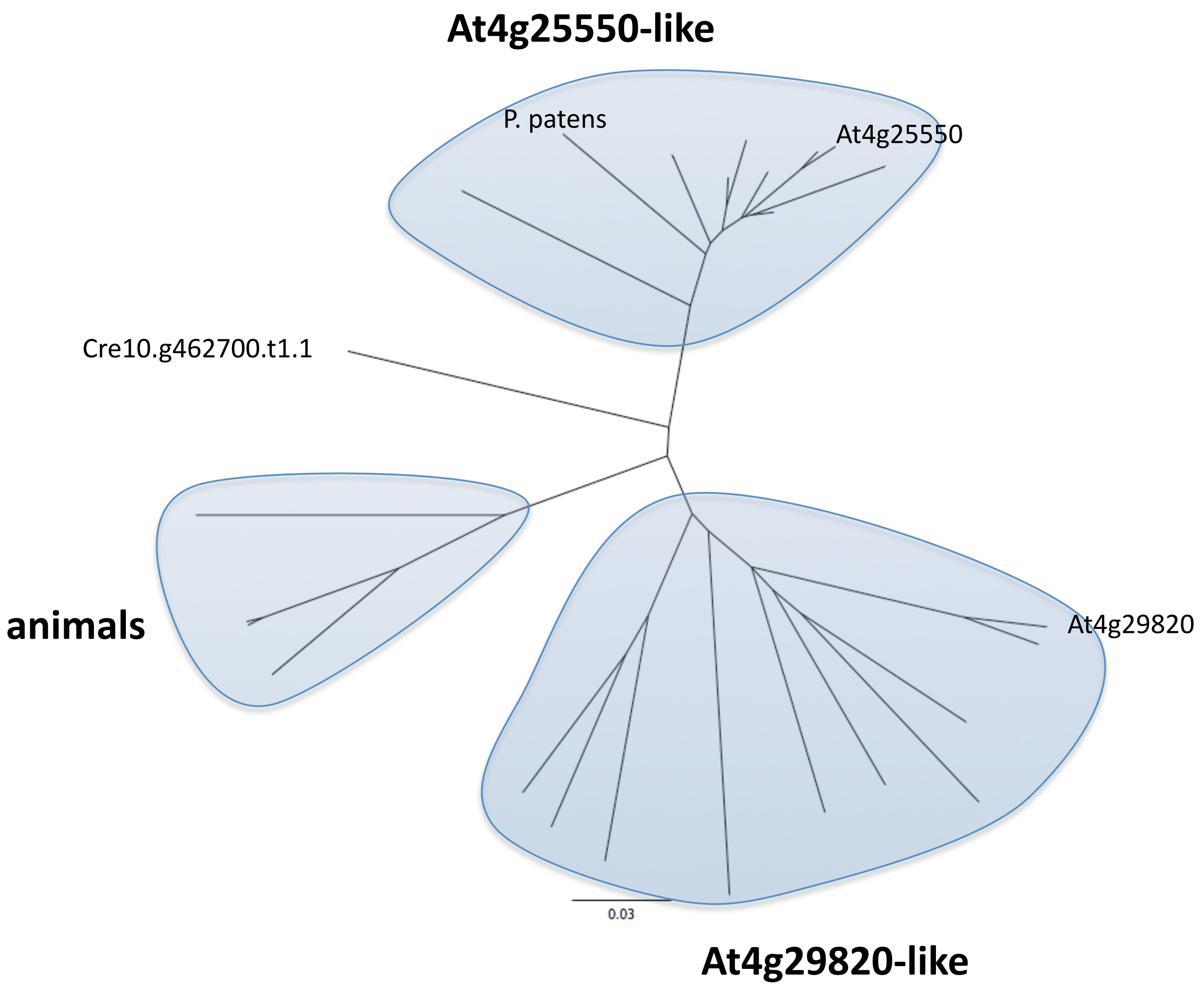

Figure S4. Tree showing the results of amino acid alignments of CFIm-25-related proteins in plants. The two classes of plant proteins are shaded with light blue balloons and labeled according to the respective Arabidopsis ortholog. Similarly, representative animal orthologs are shaded and labeled as such. The Chlamydomonas and P. patens orthologs are noted to emphasize points made in Figure 4 and the text. The sequences used to generate this tree are provided in Supplemental File 4.

Figure S5

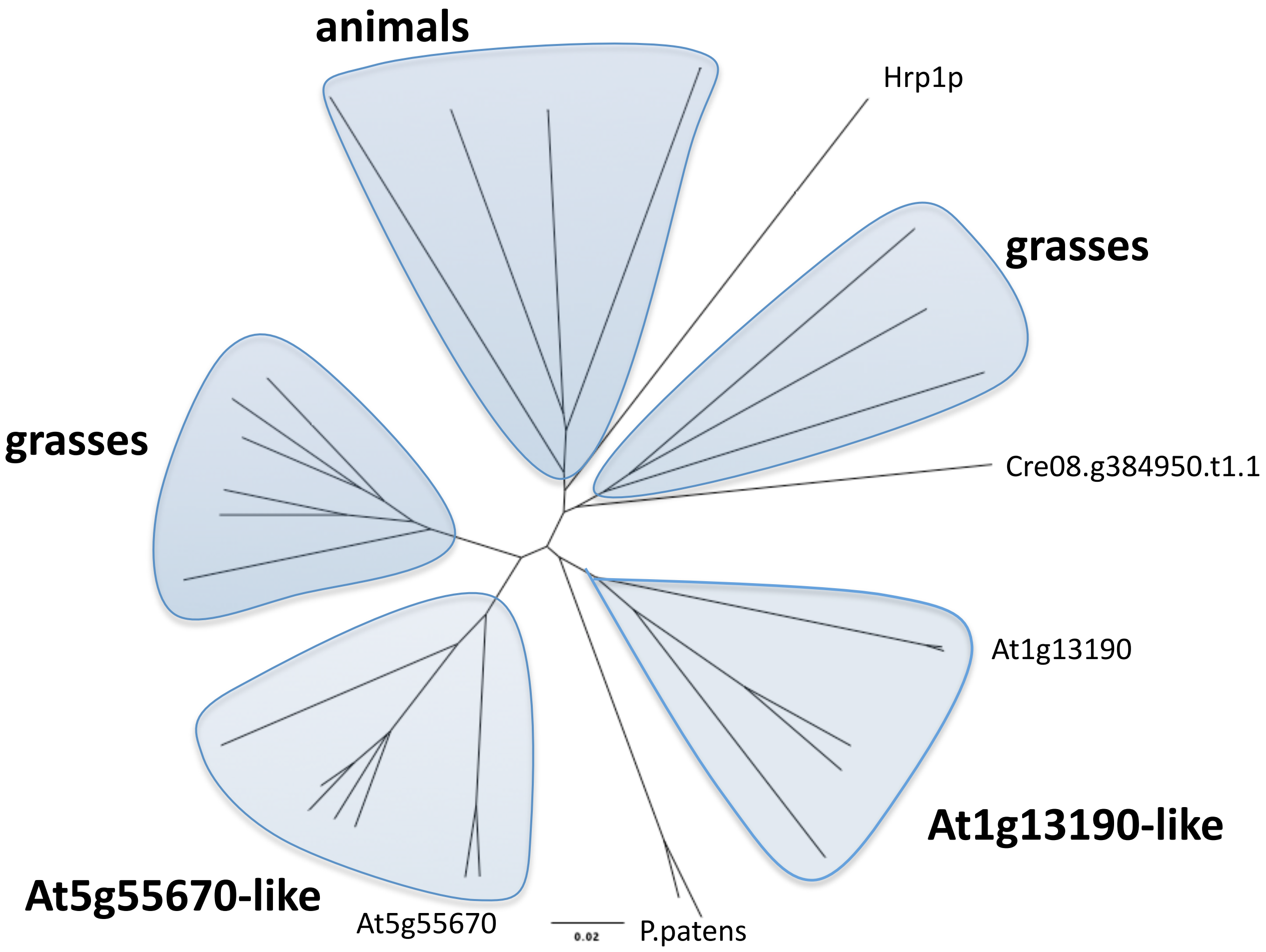

Figure S5. Tree showing the results of amino acid alignments of CFIm-68-related proteins in plants. The various classes of plant proteins are shaded with light blue balloons and labeled according to the respective Arabidopsis ortholog or as defining the two grass-specific clades. Similarly, representative animal orthologs are shaded and labeled as such. The Chlamydomonas and P. patens orthologs are noted to emphasize points made in Figure 4 and the text. The S. moellendorfii orthologs are not included here because the genes are incompletely annotated. The sequences used to generate this tree are provided in Supplemental File 4.

Figure S6

A

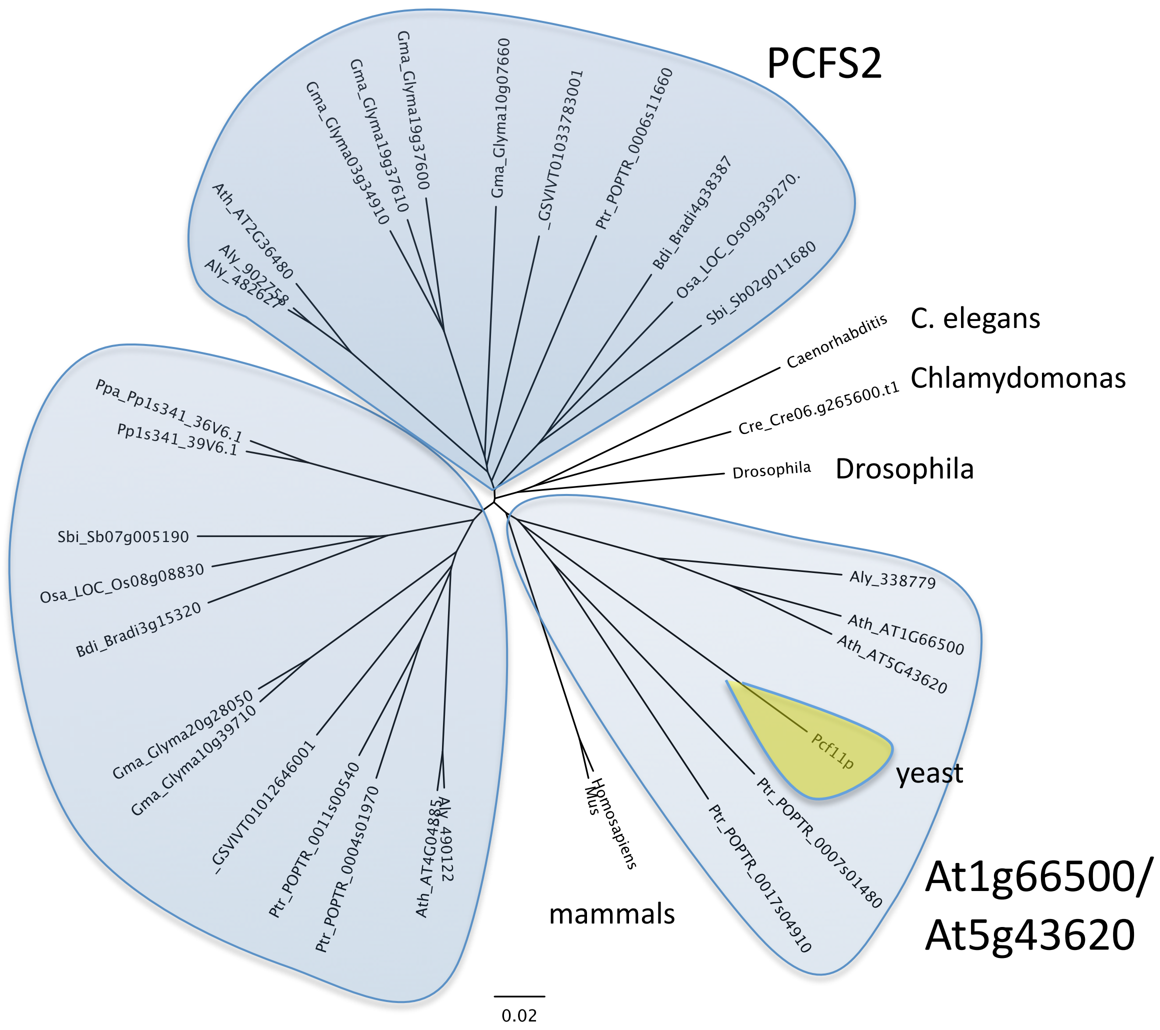

B. Rice EST

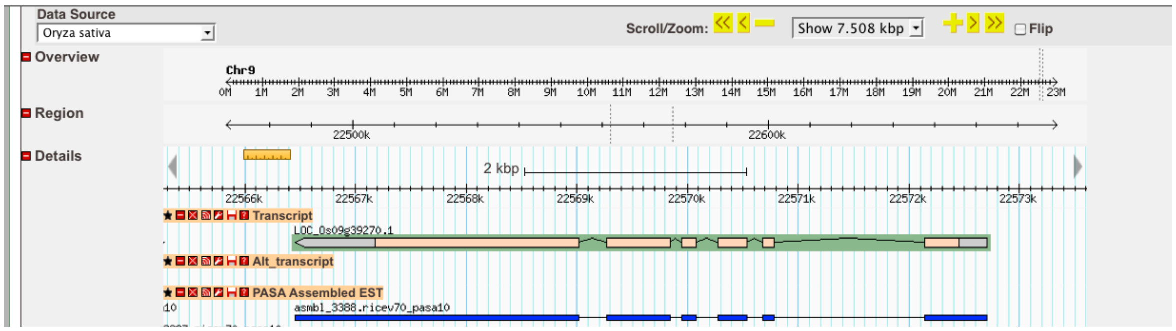

Figure S6. A. Tree showing the results of amino acid alignments of PCFS-related proteins in plants. The two classes of plant proteins are shaded with light blue balloons and labeled according to the respective Arabidopsis ortholog. Other orthologs are noted to emphasize points made in the text. The S. moellendorfii orthologs are not included here because the genes are incompletely annotated. The sequences used to generate this tree are provided in Supplemental File 4. B. Illustration of the rice ortholog of At2g36480, showing the "fusion" with the adjacent ortholog of At2g36485. The illustration was generated using the gBrowse utility at the Phytozome web site

Figure S7

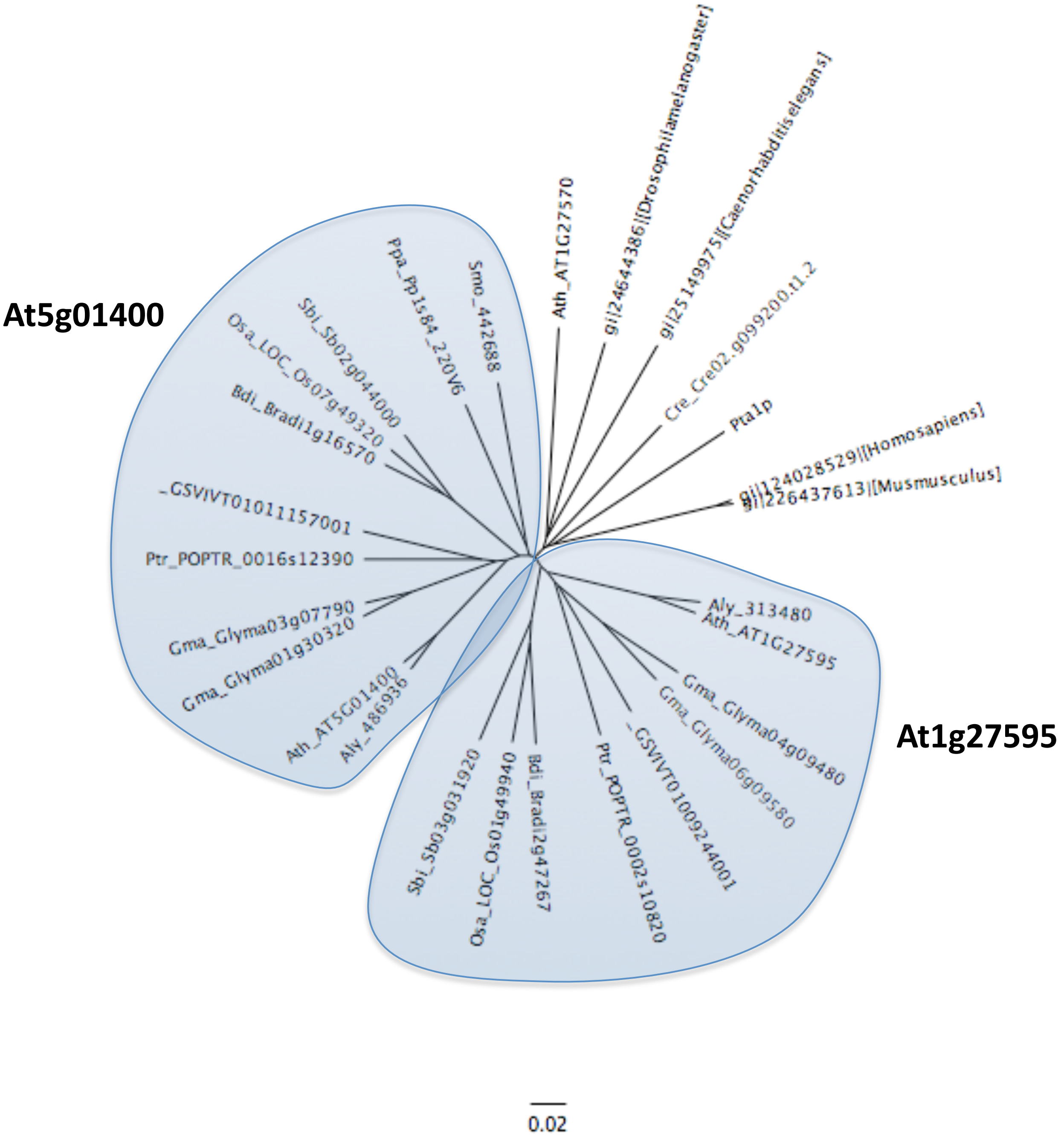

Figure S7. Tree showing the results of amino acid alignments of symplekin-related proteins in plants. The two classes of plant proteins are shaded with light blue balloons and labeled according to the respective Arabidopsis ortholog. The S. moellendorfii orthologs are not included here because the genes are incompletely annotated. The sequences used to generate this tree are provided in Supplemental File 4.

Figure S8

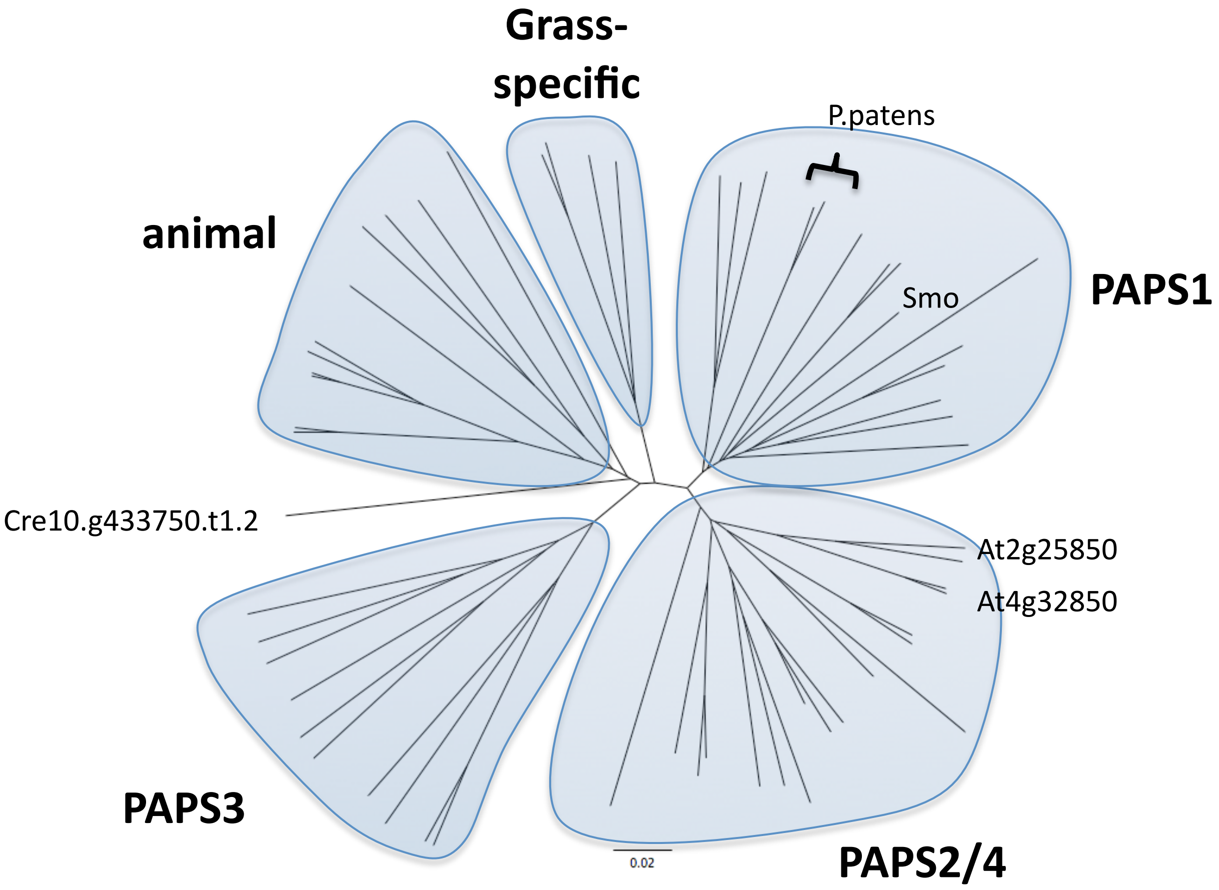

Figure S8. Tree showing the results of amino acid alignments of PAP-related proteins in plants. The four classes of plant proteins are shaded with light blue balloons and labeled according to the respective Arabidopsis ortholog, or as defining a grass-specific clade. Likewise, the animal PAPs are shaded with a separate balloon. The associations of the Chlamydomonas, P. patens, and S. moellendorfii orthologs are indicated. The sequences used to generate this tree are provided in Supplemental File 4.

Figure S9

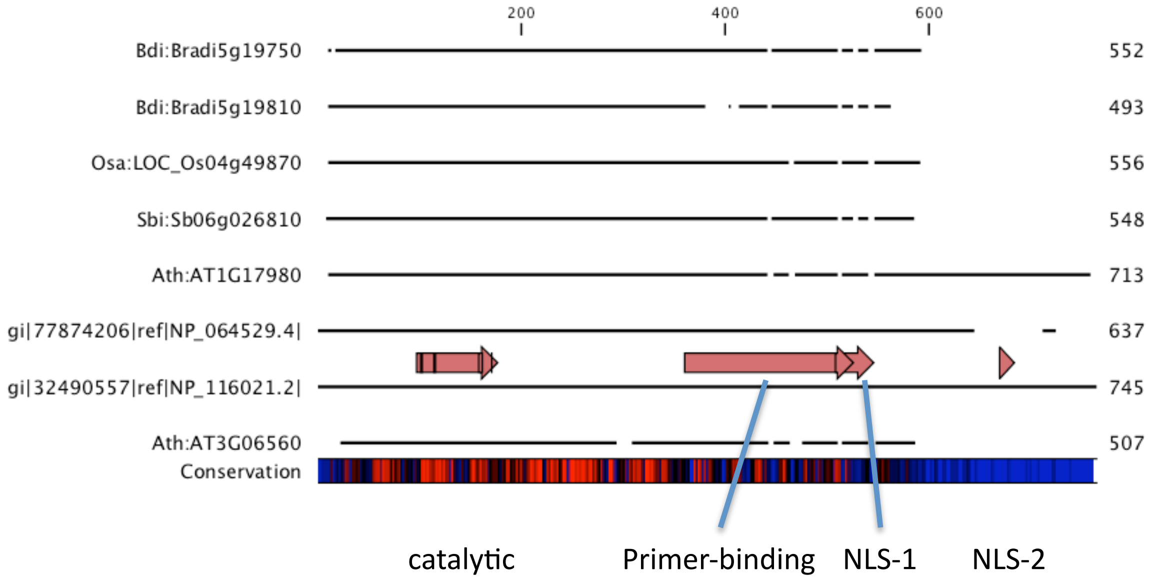

Figure S9. Illustration of the results of aligning the grass-specific PAPs with PAPS1 (At1g17980), the yeast PAP (NP_064529.4), and the mammalian PAP alpha (NP_116021.2). The locations of important functional domains are shown with large red arrowheads; these annotations are labeled beneath the drawing. Solid lines denote contiguous stretches of conserved sequence; the extent of conservation in this set of proteins is indicated by the color in the “conservation” bar (red=highly conserved, blue=not conserved). This figure was assembled using CLC Genomics Workbench.

Figure S10

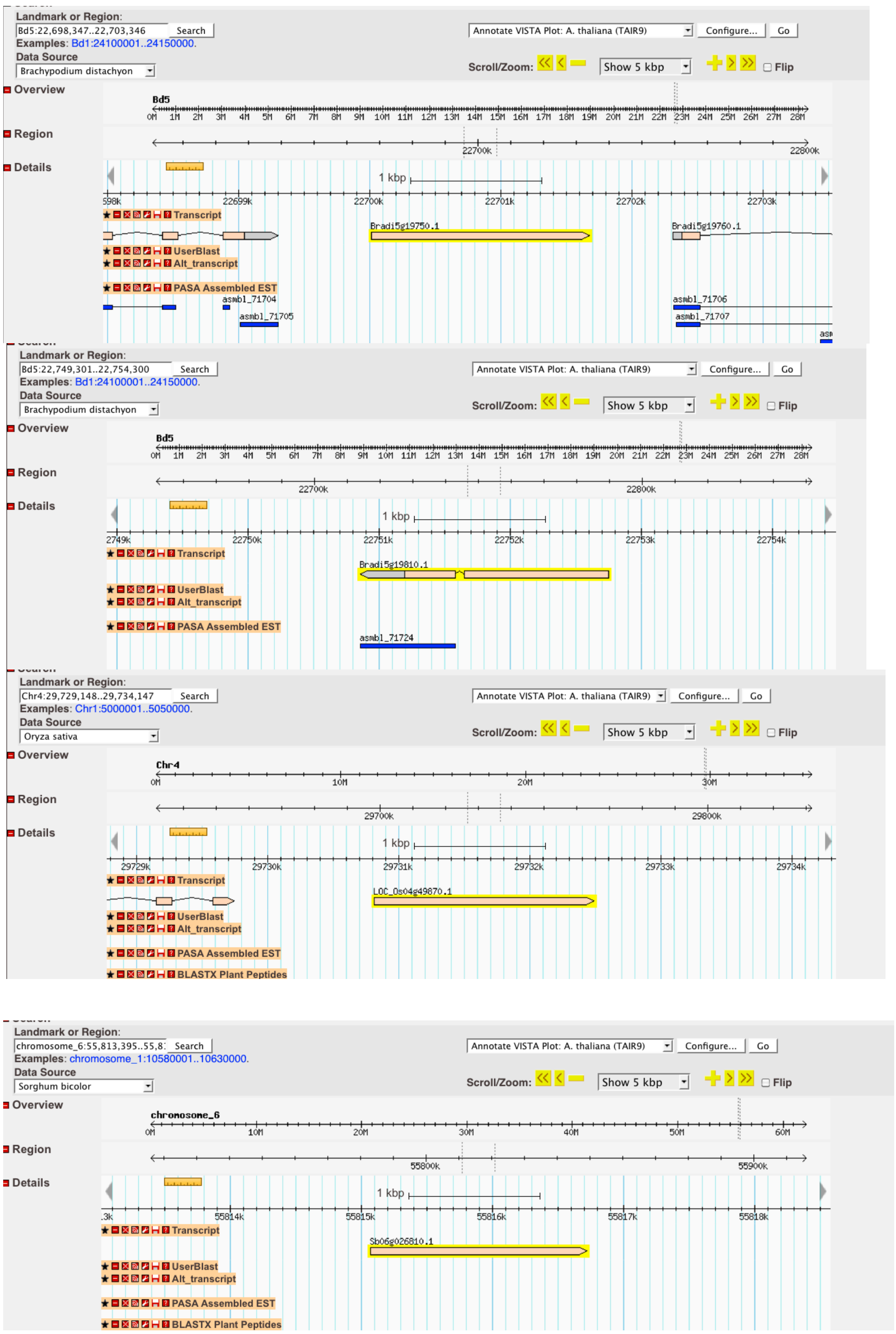

Figure S10. Intron/exon structure of the four grass-specific truncated PAP genes. Each panel was obtained using the gBrowse utility in Phytozome.

Figure S11

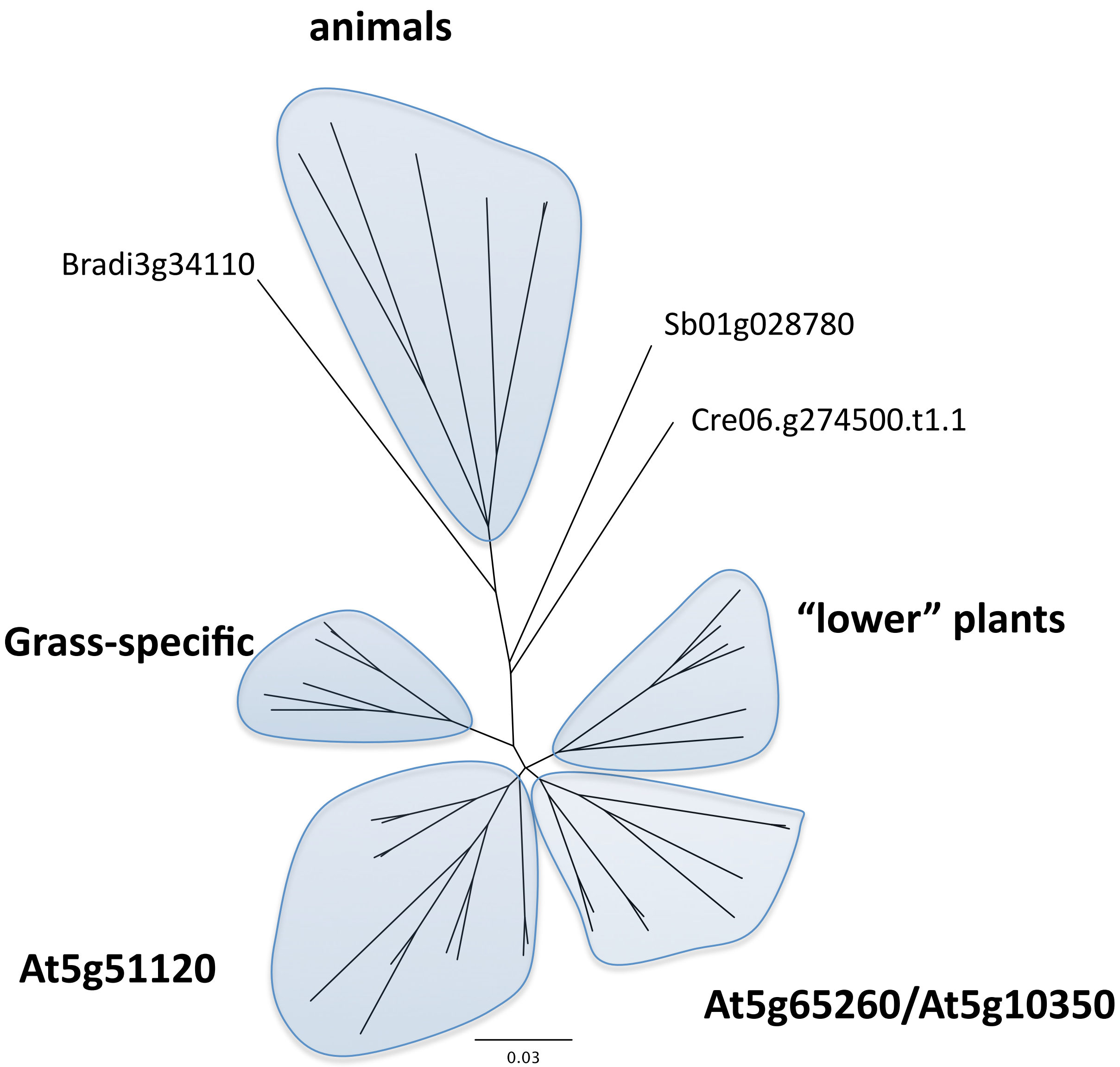

Figure S11. Tree showing the results of amino acid alignments of PABN-related proteins in plants. The four classes of plant proteins are shaded with light blue balloons and labeled according to the respective Arabidopsis ortholog, or as defining a grass- or "lower plant" (P. patens and S. moellendorfii)- specific clade. Likewise, the animal PAPs are shaded with a separate balloon. The associations of the Chlamydomonas ortholog as well as two divergent grass orthologs are indicated. The sequences used to generate this tree are provided in Supplemental File 4.

Figure S12

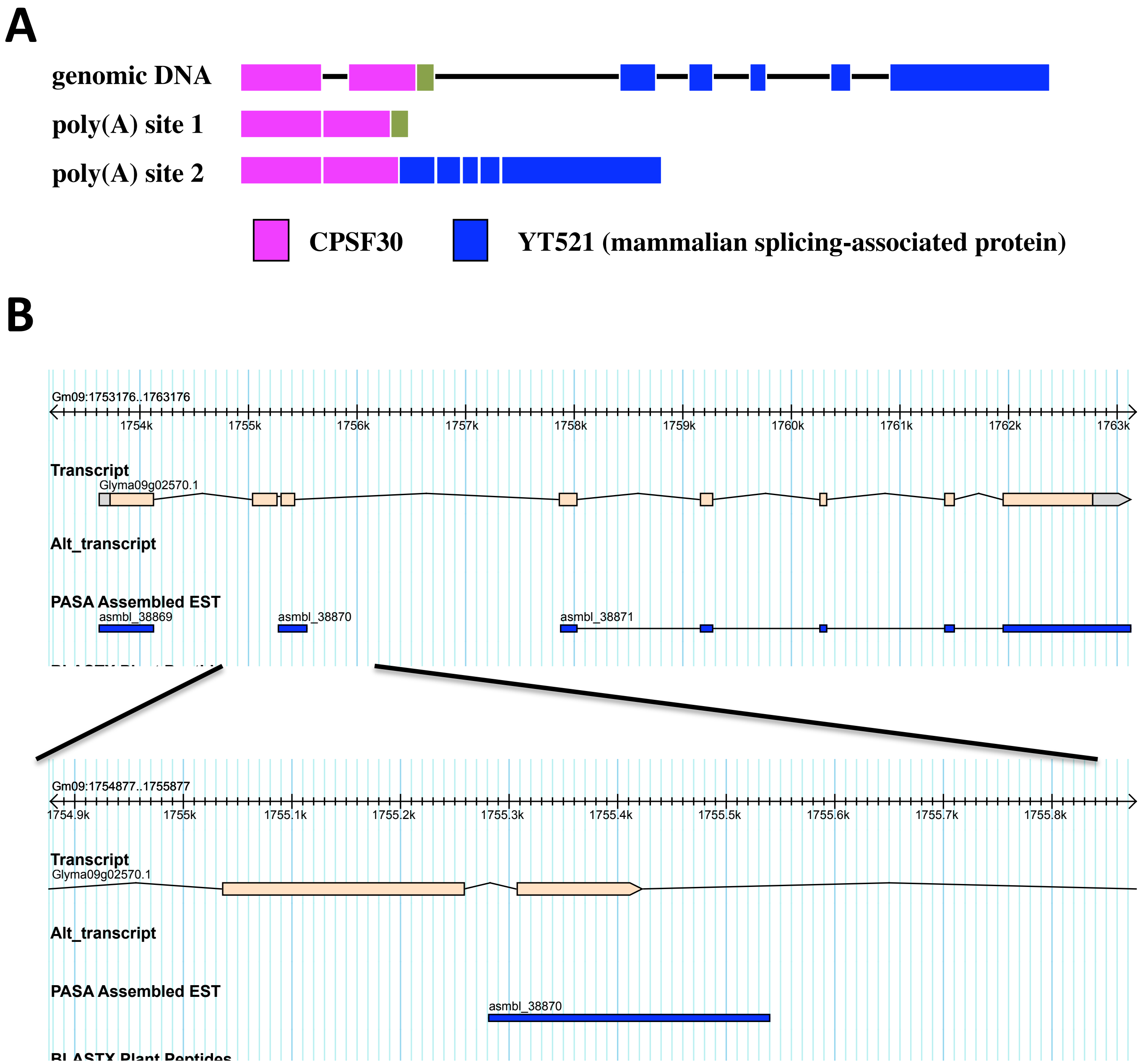

Figure S12. A. Illustration of the structures of plant CPSF30-encoding genes. The natures of the domains is explained in the text. "Genomic DNA" shows the intron-exon organization, with the 3'-UTR associated with the shorter transcript colored green. The structures of the two mRNA isoforms that result from alternative processing of transcripts encoded by plant CPSF30 genes are shown beneath and labeled according to the poly(A) site used to generate the transcript. The color coding of protein domains is shown beneath the representations of the transcripts. B. Illustration of one G. max CPSF30 gene, showing the occurrence of an EST that corresponds to mRNAs polyadenylated within the first intron. This illustration was generated using the gBrowse utility in Phytozome.

Figure S13

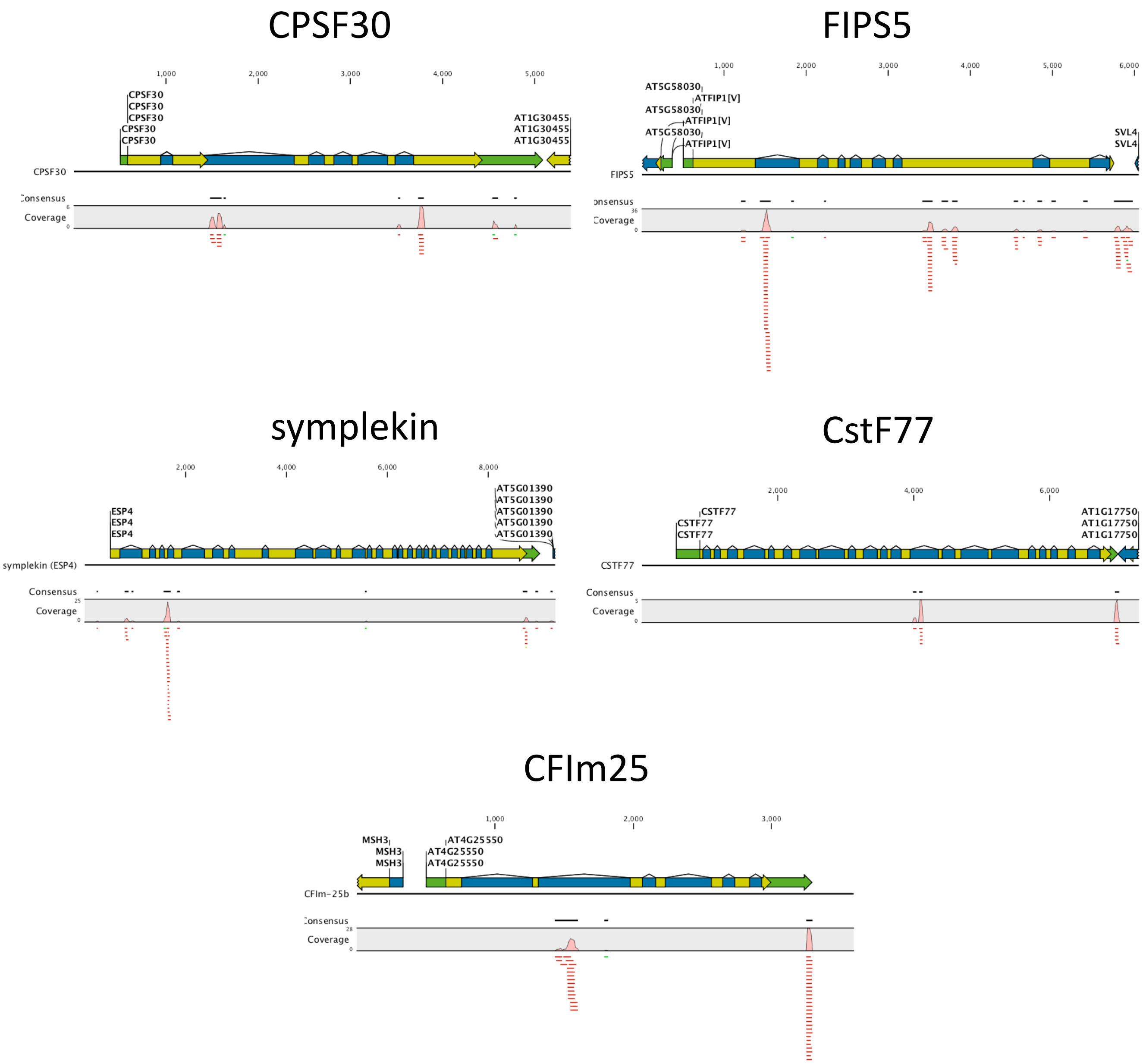

Figure S13. Alternative polyadenylation of transcripts derived from Arabidopsis polyadenylation factor-encoding genes. The identities of each gene are indicated above each illustration. The structure of each gene is shown, with coding regions in yellow, introns in blue, and untranslated regions in green. Beneath each gene representation are the results of mapping of poly(A) tags. The “consensus” line shows the extent of contiguous coverage obtained from the tag mapping, the “coverage” line shows the relative abundance of tags that map to the respective positions along the gene, and the red tics beneath the “coverage” plots show the positions of individual tags that map to each gene.

Figure S14

A.

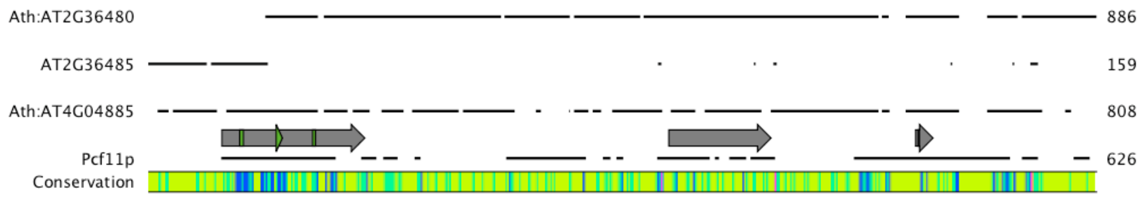

B.

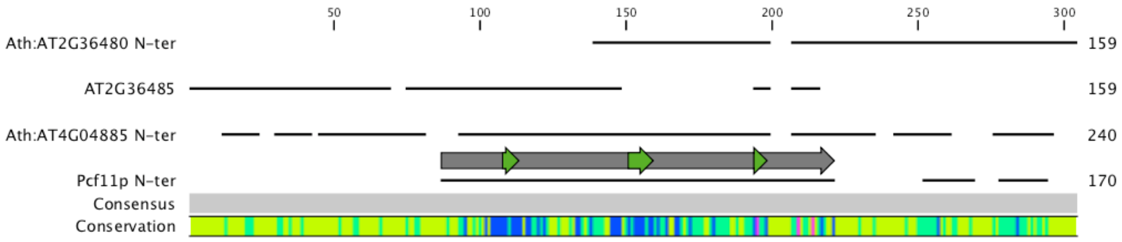

C.

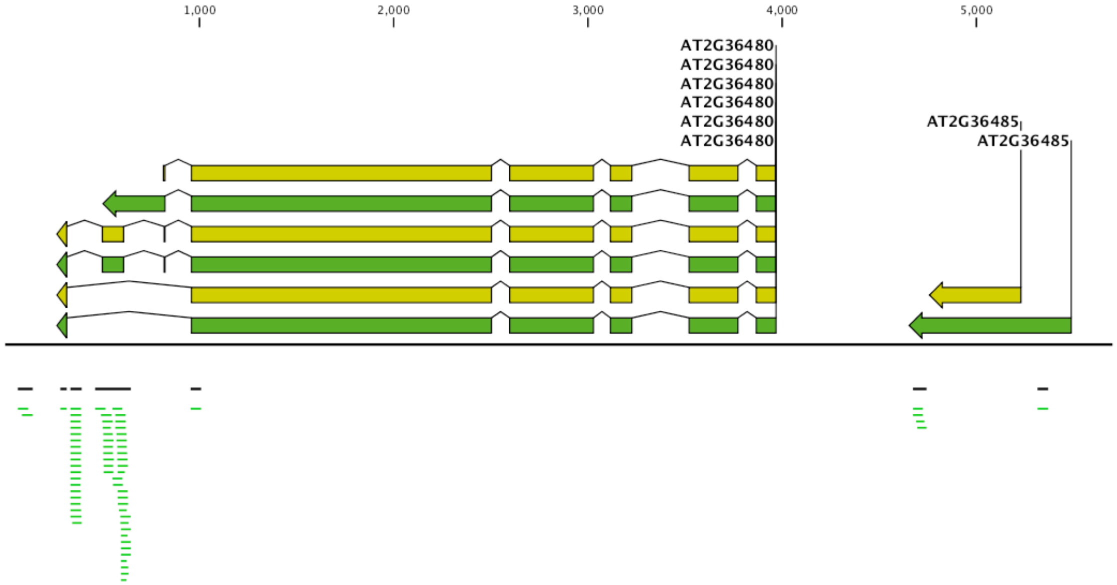

Figure S14. A. Alignment of the At2g36480 and At2g36485 polypeptides with At4g04885 and with the yeast Pcf11p protein. The locations of important functional domains are shown with large gray bars and green arrowheads; these annotations are labeled beneath the drawing. Solid lines denote contiguous stretches of conserved sequence; the extent of conservation in this set of proteins is indicated by the color in the "conservation" bar (blue=highly conserved, yellow=not conserved). This figure was assembled using CLC Genomics Workbench. B. Closeup of the N-terminal region of the alignment shown in panel A. Details are as for panel A. C. Poly(A) tags corresponding to transcripts encoded by the adjacent At2g36480 and At2g36485 genes. The orientations of these genes is right -> left (5' -> 3'), and the corresponding tags colored green. No "coverage" line is shown here; otherwise, details are as for Figure S13.
