# Supplementary figures and images for: Plant polyadenylation factors: conservation and variety in the polyadenylation complex in plants
Source: BMC Genomics. 2012 Nov 20;13:641. doi: 10.1186/1471-2164-13-641 (PMC3538716; doi:10.1186/1471-2164-13-641)

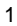

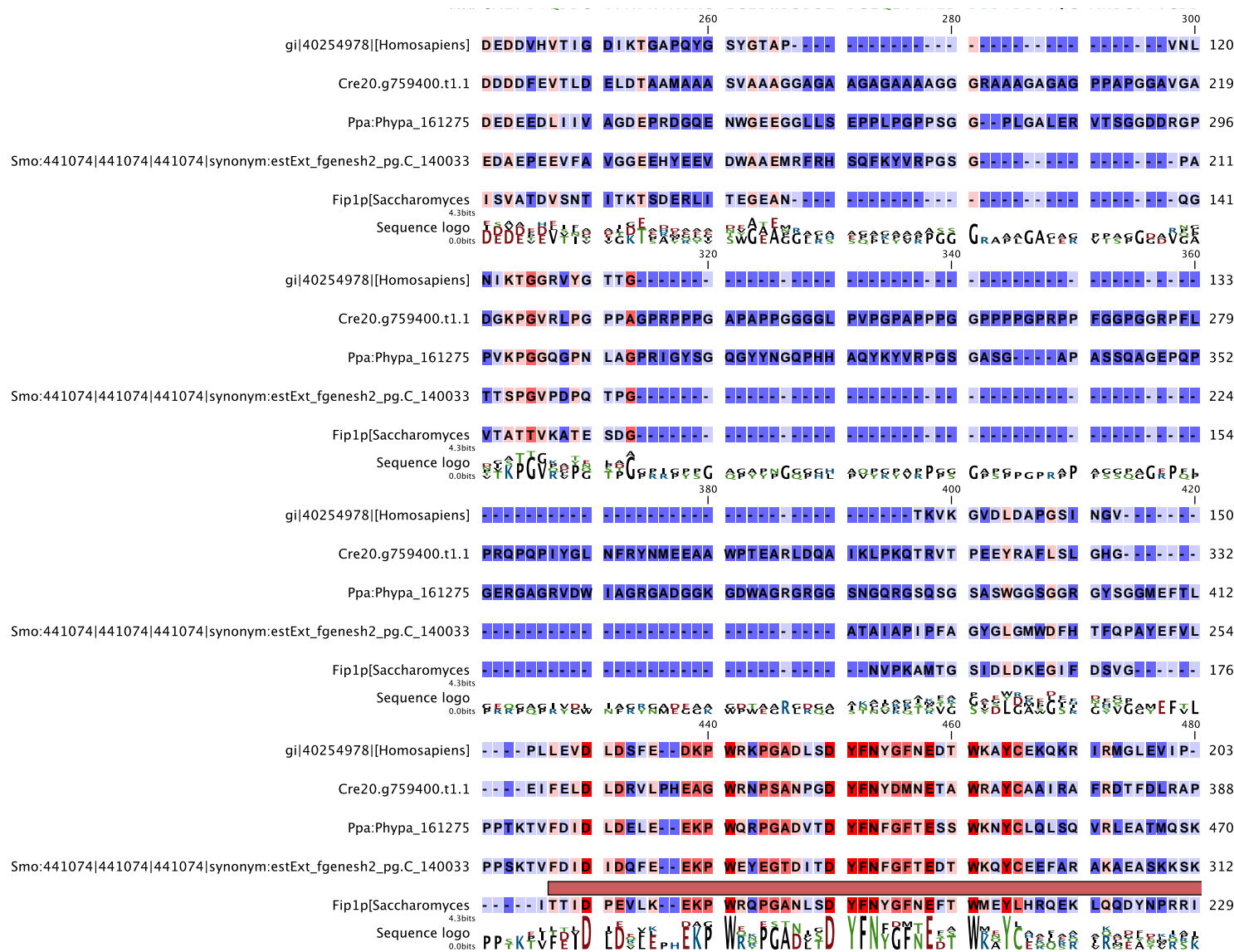

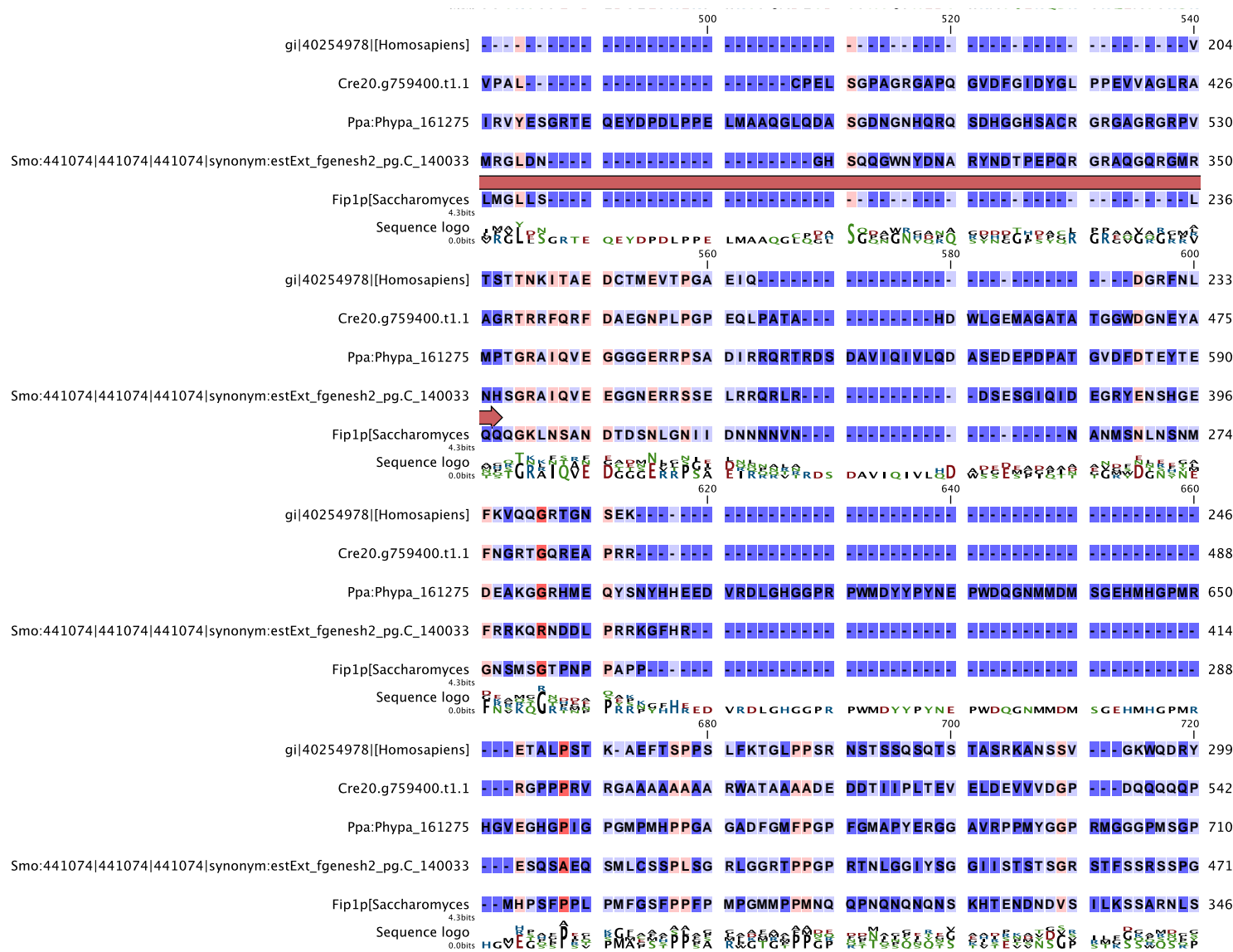

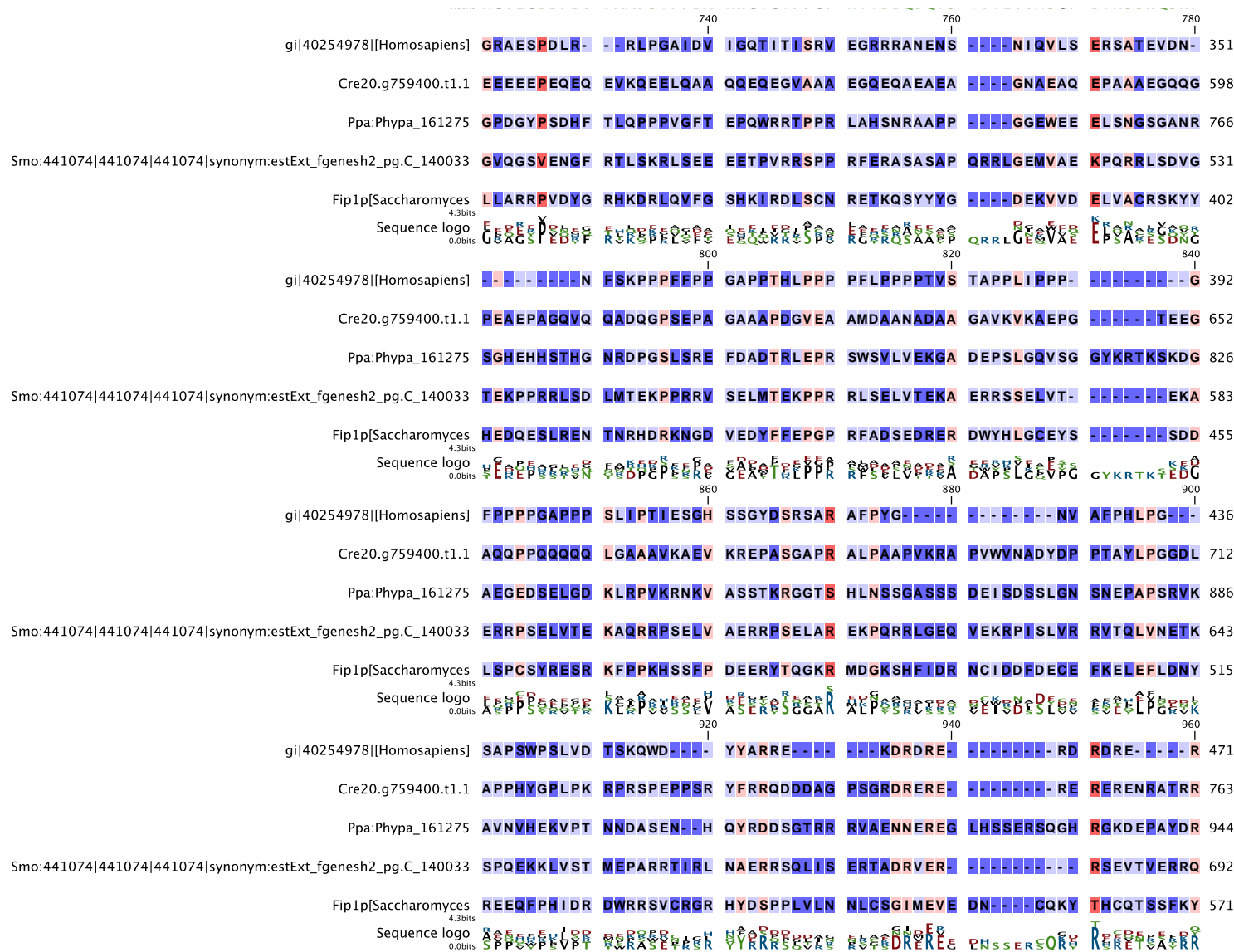

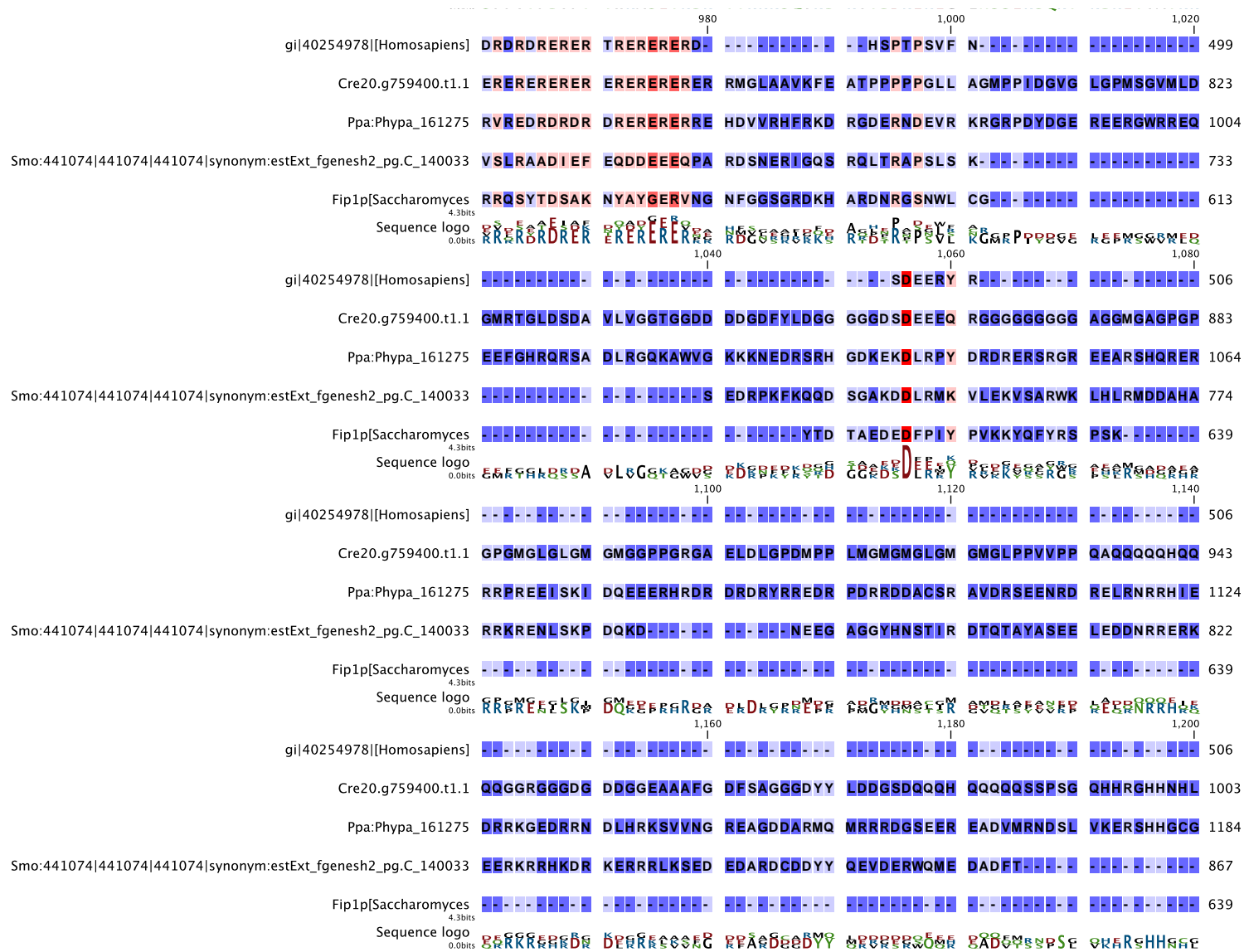

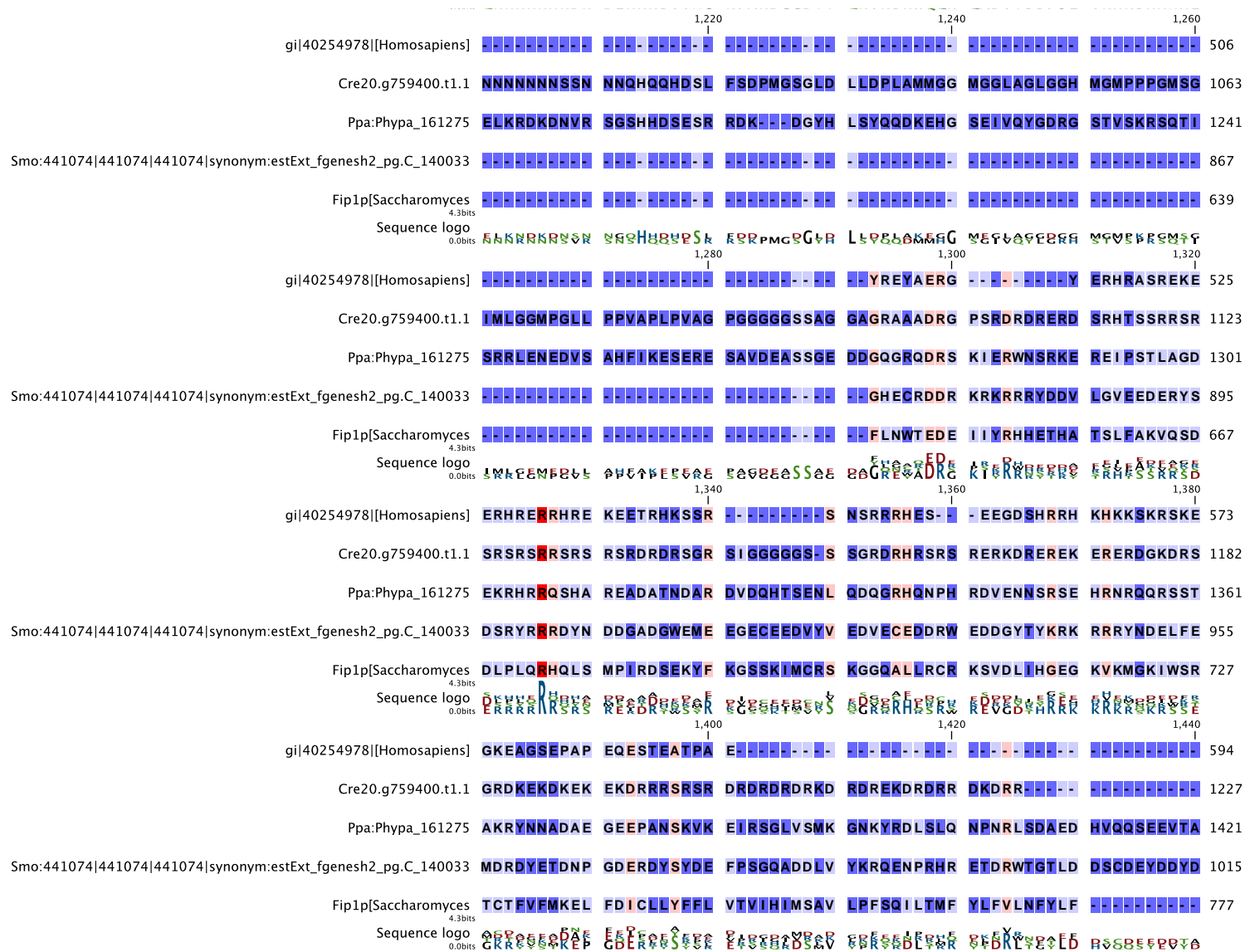

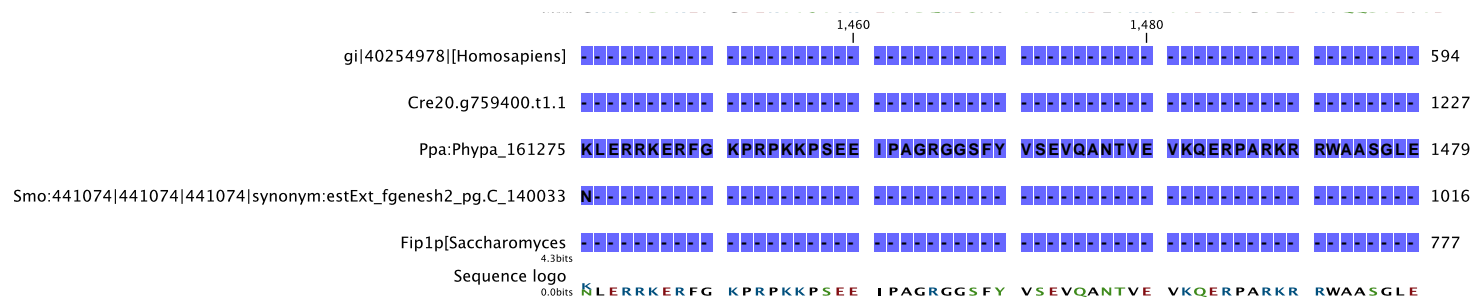

Supplement: Additional file 3 — Sequence alignment of Fip1 orthologs. This file contains the sequence alignment of Fip1 orthologs, showing their significant divergence across regions other than the conserved domain (PF05182). [file 1471-2164-13-641-S3.pdf]

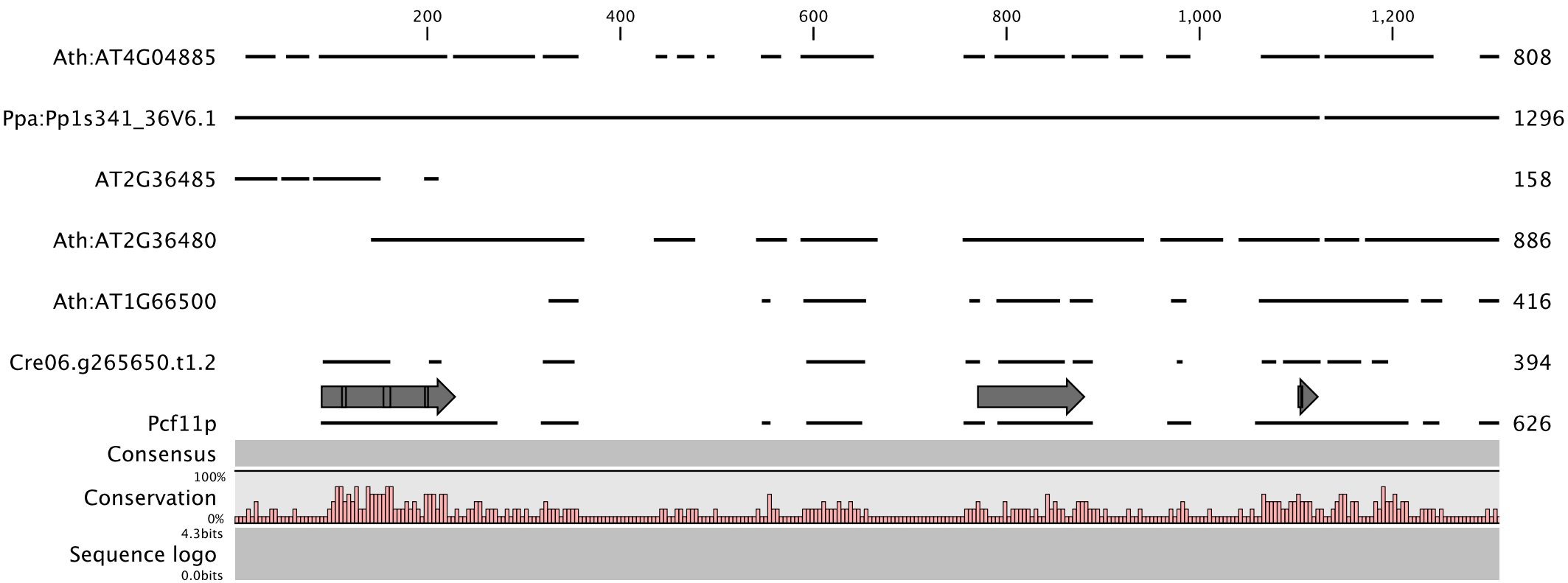

Supplement: Additional file 4 — The domain conservation of Pcf11 orthologs. The file contains the figure showing the sequence similarities of different orthologs around the three functional domains. [file 1471-2164-13-641-S4.pdf]
