# Supplementary material for: Plant polyadenylation factors: conservation and variety in the polyadenylation complex in plants
Source: BMC Genomics. 2012 Nov 20;13:641. doi: 10.1186/1471-2164-13-641 (PMC3538716; doi:10.1186/1471-2164-13-641)
Supplement: Additional file 5 — Alternative processing of FIPS5. This file contains the EST support for alternative processing of FIPS5 transcripts in poplar, soybean, rice, and Brachypodium. [file 1471-2164-13-641-S5.pdf]

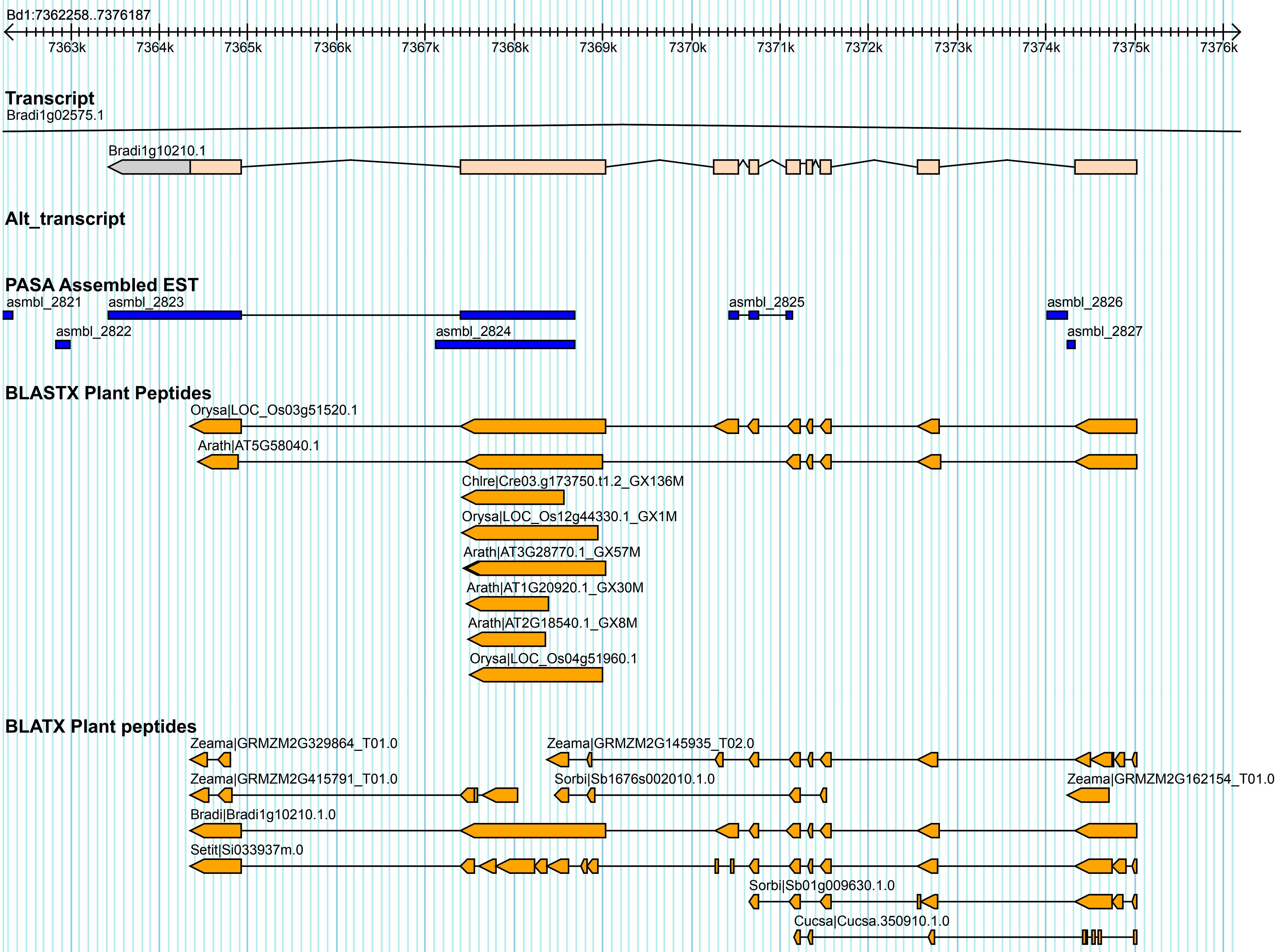
Bd1:7362258..7376187
7363k
7364k
7365k
7366k
7367k
7368k
7369k
7370k
7371k
7372k
7373k
7374k
7375k
7376k
Transcript
Bradi1g02575.1
Bradi1g10210.1
Alt_transcript
PASA Assembled EST
asmbl_2821
asmbl_2823
asmbl_2825
asmbl_2826
asmbl_2822
asmbl_2824
asmbl_2827
BLASTX Plant Peptides
Orysa|LOC_Os03g51520.1
Arath|AT5G58040.1
Chlre|Cre03.g173750.t1.2_GX136M
Orysa|LOC_Os12g44330.1_GX1M
Arath|AT3G28770.1_GX57M
Arath|AT1G20920.1_GX30M
Arath|AT2G18540.1_GX8M
Orysa|LOC_Os04g51960.1
BLATX Plant peptides
Zeama|GRMZM2G329864_T01.0
Zeama|GRMZM2G145935_T02.0
Zeama|GRMZM2G415791_T01.0
Sorbi|Sb1676s002010.1.0
Zeama|GRMZM2G162154_T01.0
Bradi|Bradi1g10210.1.0
Setit|Si033937m.0
Sorbi|Sb01g009630.1.0
Cucsa|Cucsa.350910.1.0

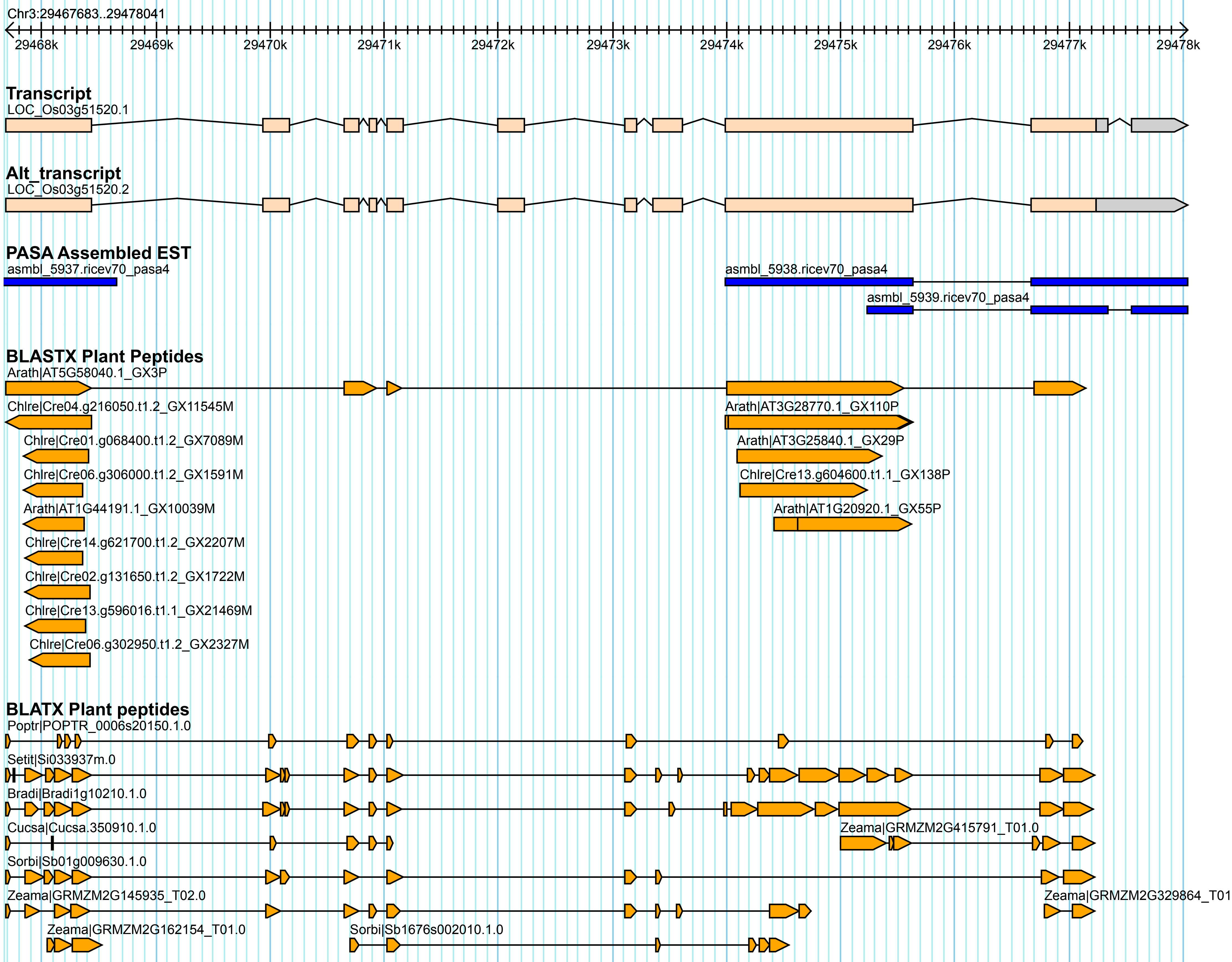
Chr3:29467683..29478041
29468k
29469k
29470k
29471k
29472k
29473k
29474k
29475k
29476k
29477k
29478k
Transcript
LOC_Os03g51520.1
Alt_transcript
LOC_Os03g51520.2
PASA Assembled EST
asmbl_5937.ricev70_pasa4
asmbl_5938.ricev70_pasa4
asmbl_5939.ricev70_pasa4
BLASTX Plant Peptides
Arath|AT5G58040.1_GX3P
Chlre|Cre04.g216050.t1.2_GX11545M
Arath|AT3G28770.1_GX110P
Chlre|Cre01.g068400.t1.2_GX7089M
Arath|AT3G25840.1_GX29P
Chlre|Cre06.g306000.t1.2_GX1591M
Chlre|Cre13.g604600.t1.1_GX138P
Arath|AT1G44191.1_GX10039M
Arath|AT1G20920.1_GX55P
Chlre|Cre14.g621700.t1.2_GX2207M
Chlre|Cre02.g131650.t1.2_GX1722M
Chlre|Cre13.g596016.t1.1_GX21469M
Chlre|Cre06.g302950.t1.2_GX2327M
BLATX Plant peptides
Poptr|POPTR_0006s20150.1.0
Setit|Si033937m.0
Bradi|Bradi1g10210.1.0
Cucsa|Cucsa.350910.1.0
Zeama|GRMZM2G415791_T01.0
Sorbi|Sb01g009630.1.0
Zeama|GRMZM2G145935_T02.0
Zeama|GRMZM2G329864_T01
Zeama|GRMZM2G162154_T01.0
Sorbi|Sb1676s002010.1.0

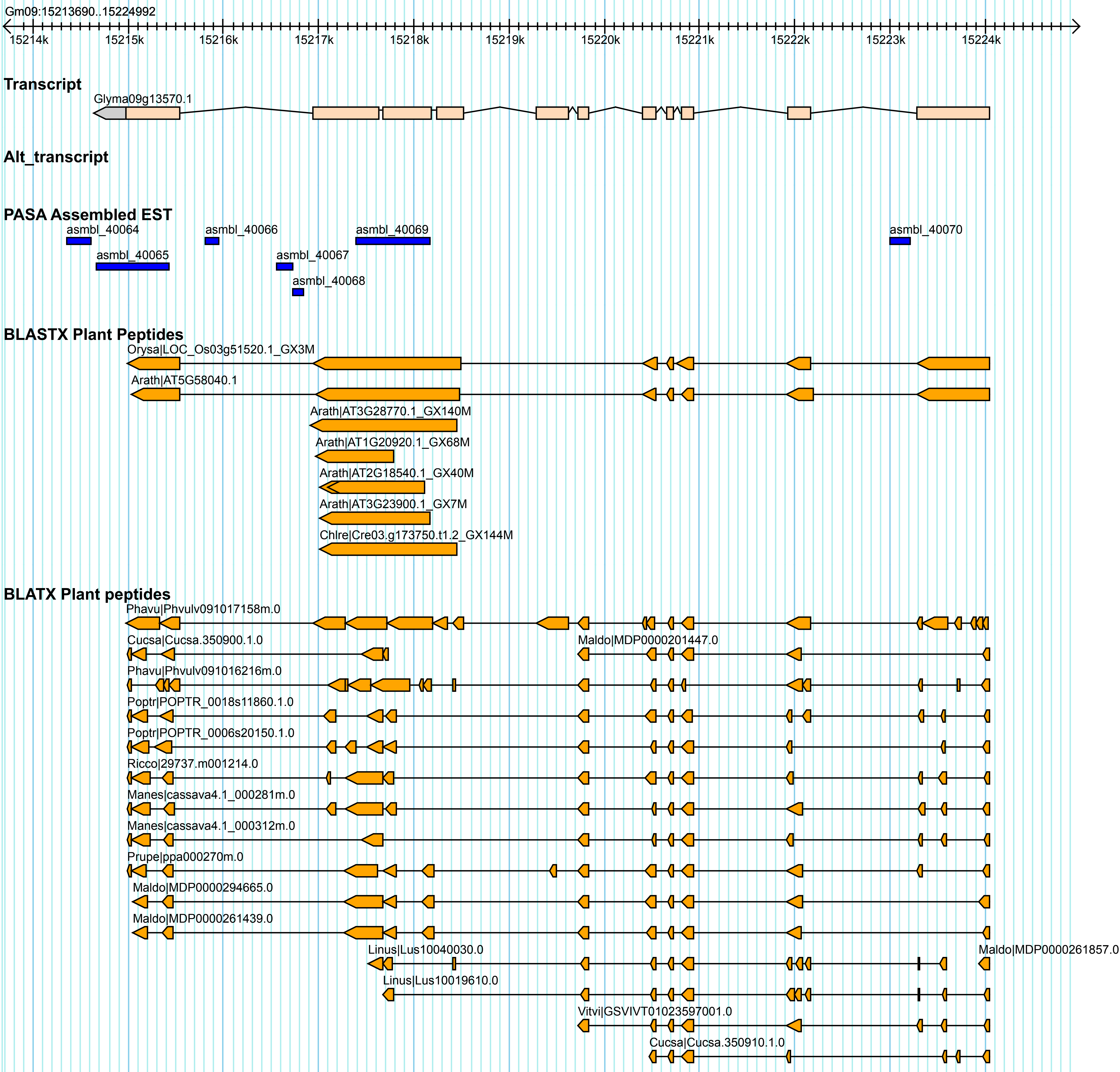
Gm09:15213690..15224992
15214k
15215k
15216k
15217k
15218k
15219k
15220k
15221k
15222k
15223k
15224k
Transcript
Glyma09g13570.1
Alt_transcript
PASA Assembled EST
asmbl_40064
asmbl_40065
asmbl_40066
asmbl_40067
asmbl_40068
asmbl_40069
asmbl_40070
BLASTX Plant Peptides
Orysa|LOC_Os03g51520.1_GX3M
Arath|AT5G58040.1
Arath|AT3G28770.1_GX140M
Arath|AT1G20920.1_GX68M
Arath|AT2G18540.1_GX40M
Arath|AT3G23900.1_GX7M
Chlre|Cre03.g173750.t1.2_GX144M
BLATX Plant peptides
Phavu|Phvulv091017158m.0
Cucsa|Cucsa.350900.1.0
Maldo|MDP0000201447.0
Phavu|Phvulv091016216m.0
Poptr|POPTR_0018s11860.1.0
Poptr|POPTR_0006s20150.1.0
Ricco|29737.m001214.0
Manes|cassava4.1_000281m.0
Manes|cassava4.1_000312m.0
Prupe|ppa000270m.0
Maldo|MDP0000294665.0
Maldo|MDP0000261439.0
Linus|Lus10040030.0
Maldo|MDP0000261857.0
Linus|Lus10019610.0
Vitvi|GSVIVT01023597001.0
Cucsa|Cucsa.350910.1.0

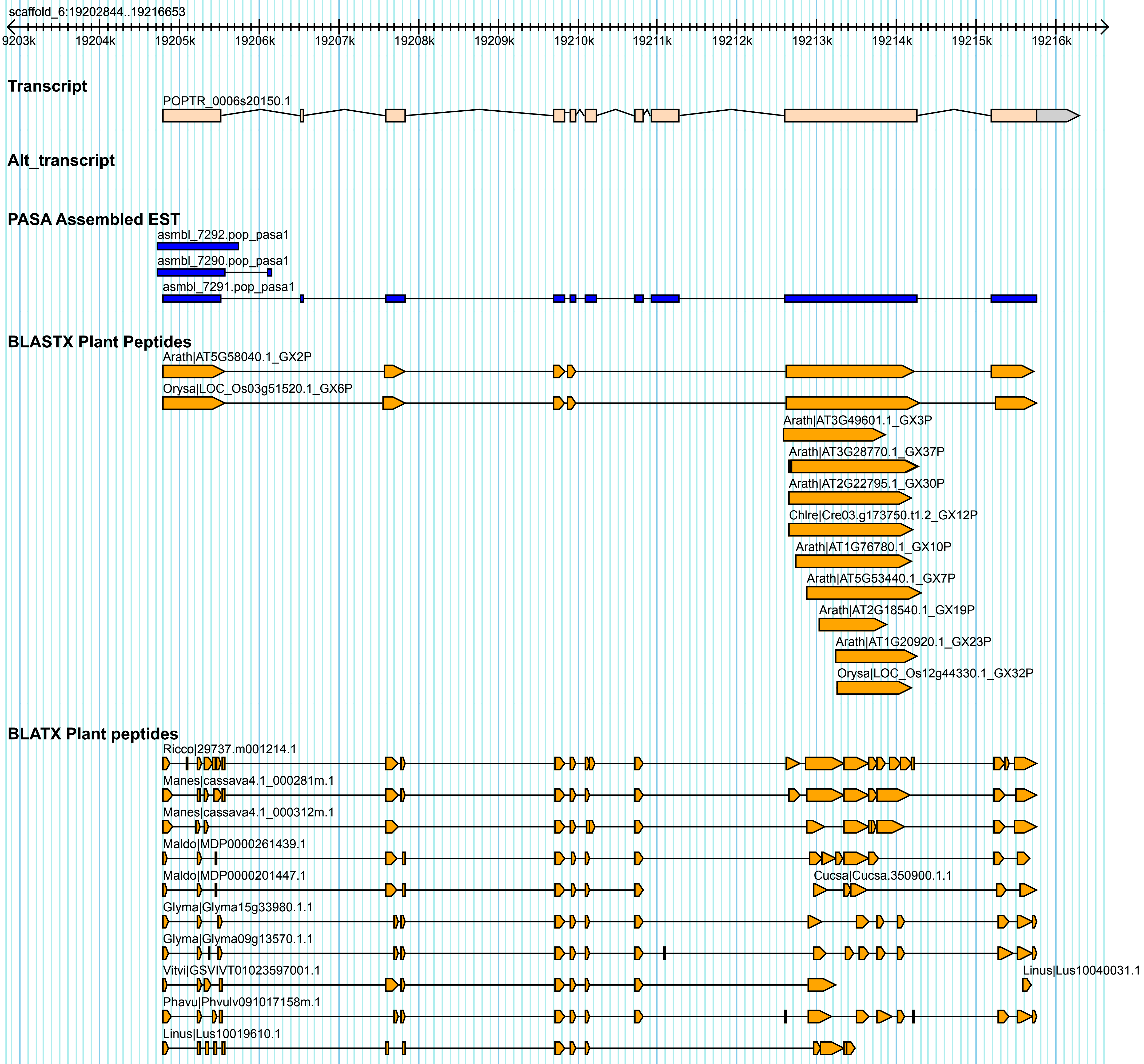
scaffold_6:19202844..19216653
19203k
19204k
19205k
19206k
19207k
19208k
19209k
19210k
19211k
19212k
19213k
19214k
19215k
19216k
Transcript
POPTR_0006s20150.1
Alt_transcript
PASA Assembled EST
asmbl_7292.pop_pasa1
asmbl_7290.pop_pasa1
asmbl_7291.pop_pasa1
BLASTX Plant Peptides
Arath|AT5G58040.1_GX2P
Orysa|LOC_Os03g51520.1_GX6P
Arath|AT3G49601.1_GX3P
Arath|AT3G28770.1_GX37P
Arath|AT2G22795.1_GX30P
Chlre|Cre03.g173750.t1.2_GX12P
Arath|AT1G76780.1_GX10P
Arath|AT5G53440.1_GX7P
Arath|AT2G18540.1_GX19P
Arath|AT1G20920.1_GX23P
Orysa|LOC_Os12g44330.1_GX32P
BLATX Plant peptides
Ricco|29737.m001214.1
Manes|cassava4.1_000281m.1
Manes|cassava4.1_000312m.1
Maldo|MDP0000261439.1
Maldo|MDP0000201447.1
Cucsa|Cucsa.350900.1.1
Glyma|Glyma15g33980.1.1
Glyma|Glyma09g13570.1.1
Vitvi|GSVIVT01023597001.1
Linus|Lus10040031.1
Phavu|Phvulv091017158m.1
Linus|Lus10019610.1

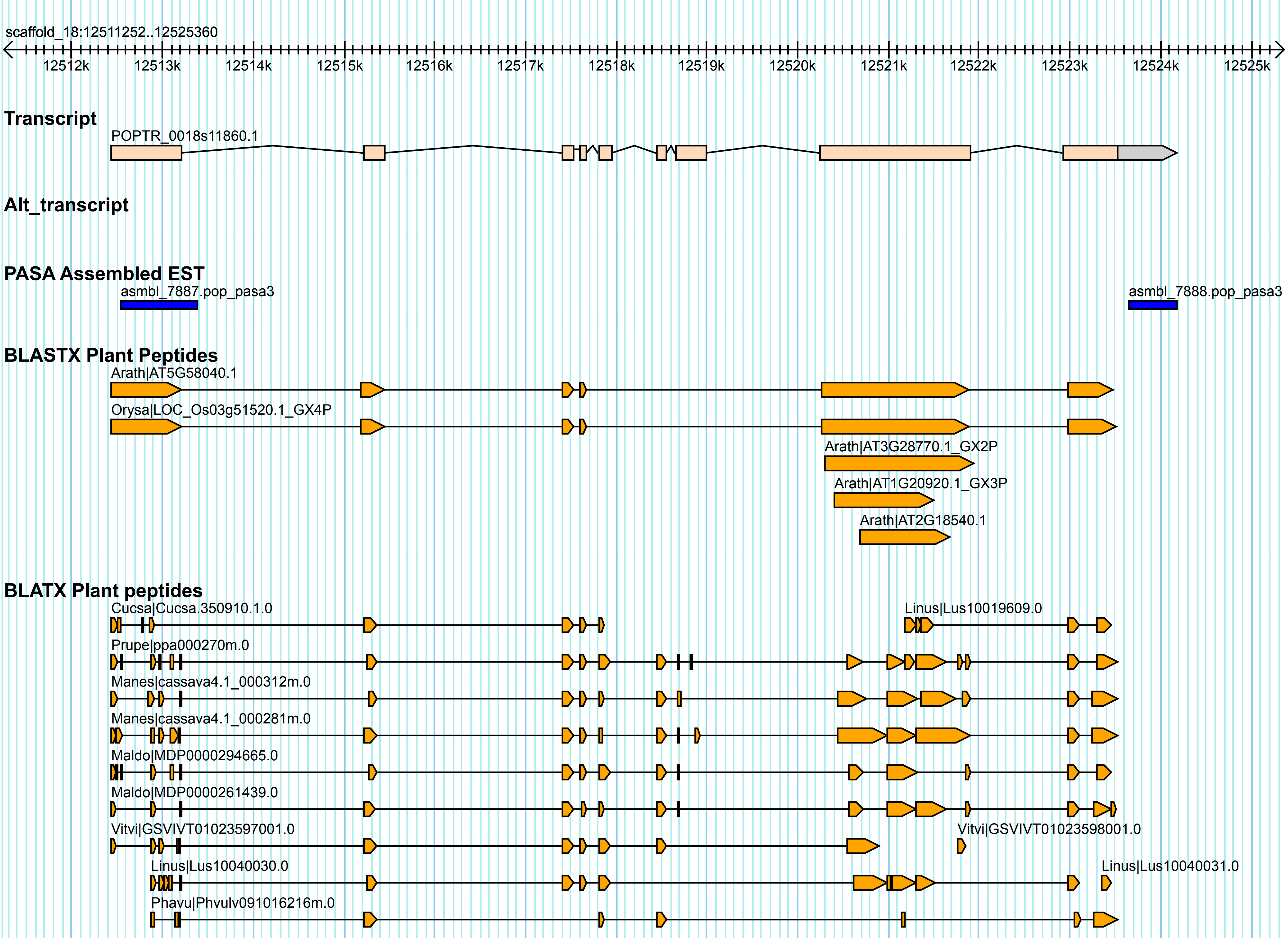
scaffold_18:12511252..12525360
12512k
12513k
12514k
12515k
12516k
12517k
12518k
12519k
12520k
12521k
12522k
12523k
12524k
12525k
Transcript
POPTR_0018s11860.1
Alt_transcript
PASA Assembled EST
asmbl_7887.pop_pasa3
asmbl_7888.pop_pasa3
BLASTX Plant Peptides
Arath|AT5G58040.1
Orysa|LOC_Os03g51520.1_GX4P
Arath|AT3G28770.1_GX2P
Arath|AT1G20920.1_GX3P
Arath|AT2G18540.1
BLATX Plant peptides
Cucsa|Cucsa.350910.1.0
Linus|Lus10019609.0
Prupe|ppa000270m.0
Manes|cassava4.1_000312m.0
Manes|cassava4.1_000281m.0
Maldo|MDP0000294665.0
Maldo|MDP0000261439.0
Vitvi|GSVIVT01023597001.0
Vitvi|GSVIVT01023598001.0
Linus|Lus10040030.0
Linus|Lus10040031.0
Phavu|Phvulv091016216m.0
