# Supplementary material for: Plant polyadenylation factors: conservation and variety in the polyadenylation complex in plants
Source: BMC Genomics. 2012 Nov 20;13:641. doi: 10.1186/1471-2164-13-641 (PMC3538716; doi:10.1186/1471-2164-13-641)
Supplement: Additional file 6 — This file contains all the sequences used to derive the phylogenetic trees in this paper. [file 1471-2164-13-641-S6.docx]

Supplemental File 4. Amino acid sequences used to generate the alignments summarized in the Supplemental Figures.

Figure S1.

>Sbi:Sb01g001930

MGTSVQVTPLSGAYGEGPLCYLLAVDGFRFLLDCGWTDLCDTSQLQPLAKVAPTVDAVLLSHPDMMHLGALPYAMKHLGLSAPVYATEPVFRLGLLTMYDHFLSRWQVSDFDLFTLDDVDAAFQNVVRLKYSQNYLLNDKGEGIVIAPHVAGHLLGGTVWKITKDGEDVVYAVDFNHRKERHLNGTVLGSFVRPAVLITDAYNALNNQGYRKKQDQDFIDSLIKVLATGGSVLLPVDTAGRVLELLLLLDTYWDERRLQYPIYFLTNVSTSTVDYVKSFLEWMRDQIAKSFESNRANAFLLKKVMLIINKEELEKLGDAPKVVLASMASLEVGFSHDIFVEMANEARNLVLFTEKGQFGTLARMLQVDPPPKAVKVTMSKRIPLVGDELKAYEEEQERIKKEKALKASLVKEEELKASLGSNAKASDPMVIDASSSRKSANAGSHFGGNTDILIDGFVPPSTSVAPMFPFFENTAEWDDFGEVINPDDYMMKQEEMDNTLMLGPGDGLDGKIDDGSARLLLDSTPSKVISNEMTVQVKCSLVYMDFEGRSDGRSVKSVIAHVAPLKLVLVHGSAEATEHLKMHCTKNLDLHVHAPQIEETIDVTSDLCAYKVQLSEKLMSNIISKKLGEHEIAWVDAEVGKEDEKLILLPPSSTPPPHKPVLVGDLKLSDFKQFLENKGWQVEFAGGALRCGEYIMVRKIGDSSQKGSTGSQQIVIEGPLCEDYYKIRELLYSQFYLL*

>Smo:441578

MGTSVQLTPLAGAHSEGPLCYLLQVDDFRFLLDCGWNDVFDVSLLQPLVSVAPTIDAVLLSHSDTLHLGALPYAIAKLGLNATVYCTHPIRSMGHMQMYDHCLSRTAVSHFDLFSLDDVDTAFSNTCPLKYSQHFPLQGKGQGITITPFPAARLLGGTIWKITKDTEDIIYAVDFNHRKERHLNATVLESFTRPAVLITDAYNALNSQPVRRQRDQEFLDIILRTLRSSGNVLLPVEPSGRVLEIILYLDQHWSQHRINVPLVFLTYVVGSVTDFVKSSLEWMNDAIGKAFEQNRENPFALRSVKLCTSRKQLDELPPGPRVVLASMASLETGFAKELFLEWAVDPKNLVLFTERAQVGTLARQLQVEPPPKIVKITISKKVLLVGEELEAYEREQSRLREEARNAASQQEPVQPASSSDDLMPSAPDESSTPSEGKQQAVTVHHDIFIDGFTVPADTVAPMFPVYDDSNERDEYGEIINPDDFVIKEEFMDYSQTQANANNIKLETEGDTSAEKPSKVVTTDTAVVPLCALTFMDFEGRADGRSIKSILAHVAPLKLVLIHGSAESTEHLKQHCLKNVCPFVYTPRVGENMNVTSDLNAYKLRLTERIMSSVLFRKLGDYELAWVDGEIGQNEEDLLPLLPLDGTPPPHKTVFVGDLRLADFKQLLATKGIQAEFAGGVLRCADNIAVRKSGGQQLVIEGSLSDDYYKVRELLYSQYHIV*

>Aly:489246

MGTSVQVTPLSGVYNENPLSYLVSIDGFNFLIDCGWNDLFDTSLLEPLSRVASSIDAVLLSHPDTLHLGALPYAMKQLGLSAPVYATEPVHRLGLLTMYDQFLSRKQVSDFDLFTLDDIDSAFQNVIRLTYSQNYHLSGKGEGIVIAPHVAGHMLGGSIWRITKDGEDVIYAVDYNHRKERHLNGTVLQSFVRPAVLITDAYHALYTNQTARQQRDKEFLDTISKHLEVGGNVLLPVDTAGRVLELLLILEQHWSQRGFSFPIYFLTYVSSSTIDYVKSFLEWMSDSISKSFETSRDNAFLLRHVTLLINKTDLDNAPPGPKVVLASMASLEAGFAREIFVEWANDPRNLVLFTETGQFGTLARMLQSAPPPKFVKVTMSKRVPLAGEELIAYEEEQNRLKREEALRASLVKEEETKASHGSDDNSSEPMVIDTKTTHDVVGSHGPAYKDILIDGFVPPSSSVAPMFPFYDNTSEWDDFGEIINPDDYVIKDEDMDRGAMHNGGDVDGRLDEATASLMLDTRPSKVISNELIVTVSCSLVKMDYEGRSDGRSIKSMIAHVSPLKLVLVHAIAEATEHLKQHCLNNICPHVYAPQIEETVDVTSDLCAYKVQLSEKLMSNVIFKKLGDSEVAWVDSEVGKTESDMRSLLPMSGAASPHKPVLVGDLKIADFKQFLSSKGVQVEFAGGGALRCGEYVTLRKVGPTGQKGGASGPQQILIEGPLCEDYYKIRDYLYSQFYLL*

>:GSVIVT01028919001

MGTSVQVTPLCGVYNENPLSYLVSIDGFNFLVDCGWNDHFDPSFLQPLARVASTIDAVLLAHPDTLHLGALPYAMKQLGLSAPVYSTEPVYRLGLLTMYDQYLSRKQVSDFDLFTLDDIDSAFQNVTRLTYSQNYHLFGKGEGIVIAPHVAGHLLGGTVWKITKDGEDVIYAVDFNHRKERLLNGTVLESFVRPAVLITDAYNALNNQPSRRQRDQEFLDVILKTLRGDGNVLLPVDTAGRVLELMLILEQYWTQHHLNYPIFFLTYVASSTIDYVKSFLEWMSDSIAKSFEHTRDNAFLLKHVTLLISKSELEKVPDGPKIVLASMASLEAGFSHDIFVEWATDAKNLVLFSERGQFATLARMLQADPPPKAVKVTMSKRVPLVGEELAAYEEEQERIKKEEALKASLSKEDEMKASRGSDNKLGDPMVIDTTTPPASSDVAVPHVGGHRDILIDGFVPPSTSVAPMFPFYENSSEWDDFGEVINPEDYVIKDEDMDQATMQVGDDLNGKLDEGAASLIFDTTPSKVISNELTVQVKCMLVYMDFEGRSDGRSIKSILSHVAPLKLVLVHGSAEATEHLKQHCLKHVCPHVYAPQIGETIDVTSDLCAYKVQLSEKLMSNVLFKKLGDYEVAWVDAEVGKTESGSLSLLPLSTPPPSHDTVFVGDIKMADFKQFLASKGIQVEFSGGALRCGEYVTLRKVGDASQKGGGAIIQQIVMEGPLCDEYYKIREYLYSQYYLL*

>Ppa:Pp1s29_309V6

MGTSVQVTPLSGAHSEAPLCYLLQVDGFRFLLDCGWTDSFDLSLLEPLKSVAPTIDAVLLSYPDTIHLGAFTYAFAKLGLQATMYCTLPVHHMGQMYMYDHVLSRKAVSNFDLFTLDDVDTSFANSVQLKYQQHYQLQGKGEGMTITPYAAGHLLGGTIWKITKDTEEIIYAVDFNHRKERHLNKTVLENFVRPAVLITDAYNALNNQPPRKQRDQEFIDMILKVLRAEGNVLLPVETAGRVLELILHLESNWAHQRLSYPVALLTNVSYSTVEFAKSLLEWMSDSIARSFGSSRENSFLLKYLKLCHDRKEFDELPSGPKVVFASMASLEGGFARDLFVEWATDSRNLVLFTERGQMGTLAKKLQAEPPPKIVKVTMSQKIPLTGEELQAYELEQRLKMATETEVDLVEEVGPNSPEAKAVTGPLPLTVAEPGGGVPLGVEGSLATNEIPSQRQILIDGFTASDKTAGPMFPLYENPSDWDEYGEVINPEDYRVEDTEMMDYQSSQQAPVADVEDNTDQEAEAILADRPSKVVVKDYTVYVKCALYYMDFEGRSDGRSIKNILAHVAPIKLVLVHGSAEATEHLRQHCVKNVCRDVYAPRIGETQDVTSDLCAYKVRLTERLMSSVLFRKLGDYEVAWIDGEIGSQESEGMLPLLPSETPPPHKSVFVGDLRLADFKQLLATKGIQAEFAGGVLRCGDAFAVRRSGGSQQLVIEGPLSEEYYKLRDLLYSQFYML*

>Ptr:POPTR_0001s23890

MGTSVQVTPLSGVYNENPLSYLVSIDGFNFLIDCGWNDHFDPSLLQPLSKVASKIDAVLLSYGDMLHLGALPFAMKQFGLNAPVFSTEPVYRLGLLTMYDQSFSRKAVSEFDLFSLDDIDSAFQNFTRLTYSQNHHLSGKGEGIVIAPHVAGHLLGGTVWKITKDGEDVVYAVDFNHRKERHLNGTVLESFYRPAVLITDAYNALNSQPSRQQRDKQFLETILKTLEGGGNVLLPVDSAGRVLELLLILEQFWGQRFLNYPIFFLSYVSSSTIDYIKSFLEWMSDSIAKSFETSRDNAFLMKHVTLLISKDELDNASTGPKVVLASVASLEAGFSHDIFAEWAADVKNLVLFTERGQFGTLARMLQADPPPKAVKMTMSRRVPLVGDELIAYEEEQKRLKREEELKASLIKEEESKVSHGPDNNLSDPMVIDSGNTHSPLDVVGSRGSGHRDILIDGFVPPSTSVAPMFPFYENSLEWDEFGEVINPDDYVVQDEDMDQAAMHVGADIDGKLDEGSASLILDTKPSKVVSNELTVQVKCSLIYMDYEGRSDGRSIKSILTHVAPLKLVMVHGSAEATEHLKQHFLNIKNVQVYAPQIEETIDVTSDLCAYKVQLSEKLMSNVLFKKLGDYEVAWVDAEVGKTENGMLSLLPISSPAPPHKSVLVGDLKMADFKQFLASKGVQVEFAGGALRCGEYVTLRKVGNPSQKGGTSGTQQIIIEGPLCEDYYKIREYLYSQFYLL*

>Ath:AT5G23880

MGTSVQVTPLCGVYNENPLSYLVSIDGFNFLIDCGWNDLFDTSLLEPLSRVASTIDAVLLSHPDTLHIGALPYAMKQLGLSAPVYATEPVHRLGLLTMYDQFLSRKQVSDFDLFTLDDIDSAFQNVIRLTYSQNYHLSGKGEGIVIAPHVAGHMLGGSIWRITKDGEDVIYAVDYNHRKERHLNGTVLQSFVRPAVLITDAYHALYTNQTARQQRDKEFLDTISKHLEVGGNVLLPVDTAGRVLELLLILEQHWSQRGFSFPIYFLTYVSSSTIDYVKSFLEWMSDSISKSFETSRDNAFLLRHVTLLINKTDLDNAPPGPKVVLASMASLEAGFAREIFVEWANDPRNLVLFTETGQFGTLARMLQSAPPPKFVKVTMSKRVPLAGEELIAYEEEQNRLKREEALRASLVKEEETKASHGSDDNSSEPMIIDTKTTHDVIGSHGPAYKDILIDGFVPPSSSVAPMFPYYDNTSEWDDFGEIINPDDYVIKDEDMDRGAMHNGGDVDGRLDEATASLMLDTRPSKVMSNELIVTVSCSLVKMDYEGRSDGRSIKSMIAHVSPLKLVLVHAIAEATEHLKQHCLNNICPHVYAPQIEETVDVTSDLCAYKVQLSEKLMSNVIFKKLGDSEVAWVDSEVGKTERDMRSLLPMPGAASPHKPVLVGDLKIADFKQFLSSKGVQVEFAGGGALRCGEYVTLRKVGPTGQKGGASGPQQILIEGPLCEDYYKIRDYLYSQFYLL*

>Cre:Cre12.g487600.t1.2

METVVRYTPLCGVGEDSPLCSLLEIDDYTILLDCGWDDSFDVALLDPVLKVLPRIDAVLLSHPSPAHLGSLPYLVGRCGLAAPVFSTKPTRRMGEMFMFEACLAHQAVSDFAAYDLDDVDAGFRLHPRWTELRYSQKHLLLPPAAPGATAGEAGAAGAAGGGQGPAGGGIAITPLPAGRYPGGAVWRLTLLGSGQEVVYAVDFNHRKERLLNETTFTTALAALQPALLIGDAVNGLAPPAPPRHKRDEEFLDAITATVEGEGNVLIPTDAAGRVLELALLLDEHFARARIAATPVVLSYTIKTVLEFARTQLEYLGSEMVQAFSHKRTIPFTFRKLAVITRLEDLGAIPGPKVVLATLPSLDCGPARQLLVDWAAAPRNTIIFTERANPGTLAHALQNHVAAPGGEPLRLPLRLASRVPLEGEELAAWQAGKEREAAEAVSRRASGALDSLPSLTLTSSTHLTAAARLAAAARLATSGGVAATPSAAAGSLARGGGGGGGAAGSKSAGPLDQPLRSCTSSISRLVGGGVVCAAAKRAAEEGLLMDGFEPPQAAAYPMFPDEDAELYVEWDEYGAKLGKEEFRVPMVLDAATGLGKAAGGGGVDGMDVDDGGGGGAGGGEGGEGDVADEVDEAPTKLLVSEVQLELRAALRFFDFEGRCDGRALRDYLAAVAPRRLALVRGSPQAIAELAGGLAADLSDYGSGVDTPGPGEVVEVRLAASHVAALSEALAGGLAVRTAGQYGVAWLEGVFGSGGPAPPLDPITGAPLLQLEPMEETEEGGGGGAAAMAAAAALAAAGGGAGSVFLASAGAALTLSKLKAALAMQDIQSEFMGRGVLAVYPSGPGGETLVVTLGGPGAVAGVATDRAHVSLEGPACDGYYTVREVIYAQFGVC*

>Bdi:Bradi2g00840

MGTSVQVTPLSGAYGEGPLCYLLAVDGFRFLLDCGWTDHCDPSLLQPLARVAPTIDAVLLSHPDIMHLGALPYAMKHLGLSAPVYATEPVFRLGLLTMYDYFLSRWQVADFDLFTLDDIDAAFQNVVRLKYSQNHLLNDKGEGIVIAPHVSGHLLGGTVWKITKDGEDVVYAVDFNHRKERHLNGTALGSFVRPAVLITDAYNALNNQVYKRQQDQDFIDSMVKVLASGGSVLLPVDTAGRVLELLLIMEQYWAQRHLVYPIYFLTNVSTSTVDYVKSFLEWMSDSISKSFEHTRDNAFLLRYVSLIINKEELEKLGDAPKVVLASMASLEVGFSHDIFVEMANEAKNLVLFTEKGQFGTLARMLQVDPPPKAVKVTMGKRIPLVGDELKAYEEEQERIKKEELLKASLSKDEELKASHGSNAKASDPMVVDASSSRKSSNAGSHVGGNVDILIDGFVPSTTSFAPMFPFFENTADWDDFGEVINPDDYMMKQDEMDNNMMLGAGDGMDGKLDEGSARLLLDSAPSKVISNEMTVQVKCSLAYMDFEGRSDGRSVKSVIAHVAPLKLVLVHGSAEATEHLKMHCAKNSDLHVYAPQIEETIDVTSDLCAYKVQLSEKLMSNVISKKLGEHEIAWVDAEVGKVDEKLNLLPPSSTPSAHKSVLVGDLKLADFKQFLANKGLQVEFAGGALRCGEYITVRKIGDSNQKGSTGSQQIVIEGPLCEDYYKIRELLYSQFFLL*

>Bdi:Bradi4g38660

MGTSVQVTPLSGAYGEGPLCYLLAVDGFRFLLDCGWTDHCDPSLLQPLARVAPTIDAVLLSHPDIMHLGALPYAMKHLGLSAPVYVTEPVFRLGLLTMYDYFLSRWQVADFDLFTLDDIDAAFQNVVRLKYSQNHLLNDKGEGIVIAPHVSGHLLGGTVWKITKDGEDVVYAVDFNHRKERHLNGTALGSFVRPAVLITDAYNALNNQVYKRQQDQDFIDSMVKVLASGGSVLLPVDTAGRVLELLLIMEQYWAQRHLVYPIYFLTNVSTSTVDYVKSFLEWMSDSISKSFEHTRDNAFLLRYVSLIINKEELEKLGDAPKVVLASMASLEVGFSHDIFVEMANEAKNLVLFTEKGQFGTLARMLQVDPPPKAVKVTMGKRIPLVGDELKAYEEEQERIKKEELLKASLSKDEELKASHGSNAKASDPMVVDASSSRKSSNAGSHVGGNVDILIDGFVPSTTSVAPMFPFFENTADWDDFGEVINPDDYMMKQDEMDNNMMLGAGDGMDGKLDEGSARLLLDSAPSKVISNEMTVQVKCSLVYMDFEGRSDGRSVKSVIAHVAPLKLVLVHGSAEATEHLKMHCAKNSDLHVYAPQIEETIDVTSDLCAYKVQLSEKLMSNVISKKLGEHEIAWVDAEVGKVDEKLNLLPPSSTPSAHKSVLVGDLKLADFKQFLANKGLQVEFAGGALRCGEYITVRKIGDSNQKGSTVSQQIVIEGPLCEDYYKIRELLYSQFFLL*

>Osa:LOC_Os09g39590

MGTSVQVTPLSGAYGEGPLCYLLAVDGFRFLLDCGWTDLCDPSHLQPLAKVAPTIDAVLLSHADTMHLGALPYAMKHLGLSAPVYATEPVFRLGILTLYDYFISRRQVSDFDLFTLDDIDAAFQNVVRLKYSQNHLLNDKGEGIVIAPHVAGHDLGGTVWKITKDGEDVVYAVDFNHRKERHLNGTALGSFVRPAVLITDAYNALNNHVYKRQQDQDFIDALVKVLTGGGSVLLPIDTAGRVLEILLILEQYWAQRHLIYPIYFLTNVSTSTVDYVKSFLEWMNDSISKSFEHTRDNAFLLKCVTQIINKDELEKLGDAPKVVLASMASLEVGFSHDIFVDMANEAKNLVLFTEKGQFGTLARMLQVDPPPKAVKVTMSKRIPLVGDELKAYEEEQERIKKEEALKASLNKEEEKKASLGSNAKASDPMVIDASTSRKPSNAGSKFGGNVDILIDGFVPPSSSVAPMFPFFENTSEWDDFGEVINPEDYLMKQEEMDNTLMPGAGDGMDSMLDEGSARLLLDSTPSKVISNEMTVQVKCSLAYMDFEGRSDGRSVKSVIAHVAPLKLVLVHGSAEATEHLKMHCSKNSDLHVYAPQIEETIDVTSDLCAYKVQLSEKLMSNVISKKLGEHEIAWVDAEVGKTDDKLTLLPPSSTPAAHKSVLVGDLKLADFKQFLANKGLQVEFAGGALRCGEYITLRKIGDAGQKGSTGSQQIVIEGPLCEDYYKIRELLYSQFYLL*

>Sbi:Sb03g028040

MAIECLVLGAGQEVGKSCVVVTIGGKRVMFDCGMHMGYHDHRHYPDFARALAAWGAPDFTTAISCVVITHFHLDHIGALPYFTEICGYHGPIYMTYPTKALAPFMLEDYRKVTMDQRGEEEQYSYEDILRCMKKVIPMDLKQTIQVDKDLVIRAYYAGHVIGAAMIYAKVGDAAMVYTGDYNMTPDRHLGAAQIDHLKLDLLITESTYAKTIRDSKHAREREFLKAVHKCVSGGGKVLIPTFALGRAQELCMLLDDYWERMDLKVPIYFSAGLTIQANVYYKMLIGWTSQKIKDSHAVHNPFDFKHVCHFERSFINNPGPCVLFATPGMISGGFSLEAFKKWAPSEKNLITLPGYCVSGTIGHKLMCGKPTRIDYKDIHIDVRCQIHQLAFSPHTDSKGIMDLTEFLSPKHVILVHGEKPQMAFLKERIESELGMPCYYPGNNESVSIPTTQNLKMSATERFITSCAVEQGKRSLHKRNLICGTGLSEVIGSDEEAAEGILLMEKHKSPKILCEDELLEVLGMEQHLVQFEPMVSRIVADVESELQRAKAADLDSDGK*

>Smo:52099

GAGQDVGKSCVIVSMGGKKIMFDCGMHMGYQDERRFPDFSQISKTGDFTHEIDCVIVTHFHLDHVGALPYFTEVCGYEGPVYMTYPTKALAPIMLEDYRKIMVDRRGEEEQFSTLHIQQCMKKVIAVDLRQTIRVSRDLAFRAYYAGHVLGAAMFYVKAGNSTVVYTGDYNMTPDRHLGAAQIDRLKPDLLITESTYATTIRESRLAKEAEFLNVVHTCVSKGGKVLIPISALGRAQELCILLDEYWERMNLKVPIYFSAGLTMQSNAYYKLLISWTNQRIKDTYVTRNAFDFKHVFPFDRTQLDGPGPCILFATPGMLTGGLSLEVLKHWAPVEQNLLIIPGFCLAGTVAQKLCSGKPTRVEVDKRTTIDVRCQIHLLAFSAHTDAKGIMDLVRQVEPHNVILVHGEKLKMDVLKARINNELGIPCHNPANHDVVEVPSHCLFNVEASKELVSLSMSSYYSSTQSAMEDSMTSDRCKPGIPVEGIVLTTNSSKIKLVHPFEVPSVLGEPKHDISFS

>Aly:910398

MKQLGLSAPVYATEPVHRLGLLTMYDQFLSRKQVSDFDLFTLDDIDSAFQNVIRLTYSQNYHLSGRGIVIAPHVAGHMLGGSIWRITKDGEDVIYAVDYNHRKERHLNGTVLQSFVRPAVLITDAYHALYTNQTARQQRDKEFLDTISKHLEVGGNVLLPVDTAGRVLELLLILEQHWSQRGFSFPIYFLTYVSSSTIDYVKSFLEWMSDSISKSFETSRDNAFLLSLEAGFAREIFVEWANDPRNLVLFTETGQFGTLARMLQSAPPPKFVKVTMSKRVPLAGEELIAYEEEQNRLKREEALRASLVKEVETKASHGSDDNSSEPMVIDTKTTHDVVGSHGPAYKDILIDGFVPPSSSVAPMFPFYDNTSEWDDFGEVINPDDYVIKDEDMDRGAMHNGGDVDGRLDEATASLMLDTRPSKVISNELIRIGFTRHLRGGLFTPKVACFKEGVMFVKRKKYYYSLKFYHEKLIKTFTEMQRLRVLYGKKLGNNSRLLLWSEQTQTGNLKLLDLNRVPSEEMDSATCRFKTPNVVKPVERNRRKS*

>Aly:322516

MAIDCLVLGAGQEIGKSCVVVTINGKRIMFDCGMHMGCDDHNRYPDFSLVSKSGDFDNAISCIIITHFHMDHVGALPYFTEVCGYNGPIYMSYPTKALSPLMLEDYRRVMVDRRGEDELFTTAHIANCMKKVIAIDLKQTIQVDEDLQIRAYYAGHVLGAVMVYAKVGDAAIVYTGDYNMTTDRHLGAAKIDRLQLDLLISESTYATTIRGSKYPREREFLQAVHKCVAGGGKALIPSFALGRAQELCMLLDDYWERMNIKVPIYFSSGLTIQANMYYKMLISWTSQNVKEKHNTHNPFDFKNVKDFDRSLIHAPGPCVLFATPGMLCAGFSLEVFKHWAPSPLNLVALPGYSVAGTVGHKLMSGKPTTVDLYNGTKVDVRCKIHQVAFSPHTDAKGIMDLTKFLSPKNVVLVHGEKPSMMILKDKITSELDIPCFVPANGETVSVASTTYIKANASDMFLKSCSSPNFKFSNSTQLRVTDQRTADGVLVIEKSKKAKIVHQDEVSEVLHEKNHVVSLAYCCPVKVKGESDNDADLIKQLSEKILKTVSGAQIHESENFVKKKLMSSSDSTAARDQHAPLLRPRHDGSSSSSSSSSSARPTALAVLLGRITGHRAPSMLVRETAARALEERRIDWGYSKPVVAADILWNAALVLASAVMLVGTVEERPNEPIRVWICGYGLQCLIHVVLVWSEYWRRNTTRRARDLESGDHEDYSVYDYEQDSDNSTTYRLSVIFLAIDVFFAIFCVVLACLVGIALCCCLPCIIALLYAVAGTEGVSEAELGVLPLYKFKAFHSNEKNITGPGLLHMSELIRGWCRAS*

>Gma:Glyma02g09380

MAIETLVLGAGQEVGKSCVVVTINAKRIMFDCGMHMGYLDHRRYPDFTRISPSRDLNSALSCIIITHFHLDHVGALAYFTEVLGYNGPVYMTYPTKALAPLMLEDYRKVMVDRRGEEELFSSDQIAECMKKVIAVDLRQTVQVEKDLQIRAYYAGHVIGAAMFYAKVGDAEMVYTGDYNMTPDRHLGAAQIDRLRLDLLITESTYATTIRDSRYAREREFLKAVHKCVSCGGKVLIPTFALGRAQELCILLEDYWERMNLKVPIYFSAGLTIQANAYYKMLIRWTRQKIKDTYSKHNAFDFKNVQKFERSMIDAPGPCVLFATPGMLSGGFSVEVFKHWAVSENNLVSLPGYCVPGTIGHKLMSDKHDKVDLDPNTKIDVRCQIHQLAFSPHTDSKGIMDLVNFLSPKHVILVHGEKHKMASLKEKIHSELGIQCYDPANNETVTIPSANYVYAETSDTFIRSCLSPNFTFQKCSSVDLCNSTTVDRNLMPELQVEDERVAEGVLVLEKGKKAKIVHQDELLLMLEEQKHDA*

>:GSVIVT01011657001

MAIECLVLGAGQEVGKSCAVVTINGKRIMFDCGMHMGYLDHRRFPDFSLISKSADFNTAIDCIVITHFHLDHVGALPYFTEVCGYSGPIYMTVIPKQGGAVYSVRGYRFGKQKDINGENLRGSSGAIKGERERSGEGGARQEGAEPDGPLGHMTPLVPPPVRRVGLGIRGPDSLWSVIYFFGFYIAVIAVDLKQTVQVDKDLQIRAYYAGHVLGAAMFYAKVGDAAMVYTGDYNMTPDRHLGAAQIDRLQLDLLITESTYATTVRDSKYAREREFLKAVHKCVADGGKVLIPTFALGRAQELCILLDNYWERMNLKVPIYFSAGLTIQANMYYKMLISWTNQRVKETYATHNAFDFKNVRSFDRSLINAPGPCVLFATPGMISGGFSLEVFKLWAPSEMNLVTLPGYCLAGTIGHKLTTGKPTKIDLDKDIQISVRCQIHQLSFSPHTDAKGIMDLVKFLSPKHVILVHGEKPKMASLKGKIESDLGIQCYYPANNDTVCIPSTCWLKADTSKTFIRSSLNPNFKFVKTISEDKSNLVSKETEATSVLQVHDERVAEGILIVEKSKKAKVVHQNELLLMIGKDKHDVQFAYCCPNVVSTSDECSWLHLLFAKLATKLGGNIQDFGQHLQVDSIHISVCLKDICPYRTTDGPQKEPAVFFCCTWSVADVNLAWEIISIMENLDLSII*

>Ppa:Pp1s116_47V6

MLIKCVPLGAGQDVGKSCVIVTIGGKNIMFDCGMHMGYQDERRYPDFSFISKSGDFTHVIDCVIVTHFHLDHIGALPYFTEVCGYDGPIYMTYPTKALAPLMLEDYRKVMVERKGEQEQFSVLQIQKCMKKVTAVDLRQTIKVGADLEFRAYYAGHVLGAAMFWVKAGDDTVVYTGDYNMTPDRHLGAAQIDRLEPDLLITESTYATTVRDSKRAREREFLKAVHKCVAAGGKVLIPVFALGRAQELCILLDEYWERTNLDMPIYISAGLTMQANVYYKLLISWTNQKVKDTYVTRNTFDFKHVIPFERSKIDAPGPCVLFATPGMLSGGLSLEVFKHWAPSESNMIILPGFCVAGTVGSKLMPGKPAKIDLDKRTTLDVRCQIQHLSFSAHTDAKGILDLVRHVAPRNVVLVHGEKPKMAILKKKISSDLCIPCYDPANLETVEITPRCPIKVGVSKQFLESNLKKSEQECILDVPLEEEGSQTEIVASLDLSQLQTEDRQFQLPRAREDILVNGFVIMEDSLRIKVIHPDEASSVLGLTRHHMSFSCLCPIYPAHNDGKNDLPVRCPSPVDLNAAEDGRQETLVGIKRQEEEPSKKRHRMDLQRNLSLDLEREPVLEESTEDACEFESSSRDFFNGPPQETNHNTVRGLGGGISLDINATPDPSTPAESPSVGSRGQDMEFVSVWPAQSQQRLEPLSVLHVIFMCLVMQLGEGVVEEKHECIQGRSIRISVSPEGKNLDLENVSHSNDGMTTTKPVVVLLCEWSSEAQILADRILNVLTKLKLKL*

>Ptr:POPTR_0010s11650

MAIECLVLGAGQEVGKSCVVVTINGKRIMFDCGMHMGYDDHRRYPDFSLISKSRDFDHSLDCVIITHFHLDHVGALPYFTEVCGYNGPIYMTYPTKALAPLMLEDFRKVLVDRRGEEEQFTSLHISQCMEKVIAVDLKQTVQVDDDLQIRAYYAGHVLGAAMFYAKVGDSAMVYTGDYNMTPDRHLGAAQIDRLELDLLITESTYATTIRDSKYAREREFLKAVHECVAGGGKVLIPTFALGRAQELCILLDDYWERMNLKVPIYFSAGLTIQANLYYKILISWTSQKVKETYATRNAFDFKHVHNFDRSLINAPGPCVLFATPGMISGGFSLEVFKQWAPCEMNLITLPGYCVAGTVGHKLMSGKPTKINLDKDTQIDVRCQIHQLSFSPHTDSKGIMDLTKFLSPRNVILVHGEKPKMVSLKERIQTELRIPCYLPANCDAVHIPSTIYVKAHASNTFIRSCLNPNFRFLKRSKEDNSDQVLRNTNPTAPLQVNDERVAEGILIMEKGKKARVVHQDDLLLMLRQKKHDVQFAYCCAAQLDNLEETRNRDDALGLSDKCSSLQLLFKELSNYFSGVNIEDLGEHLQVESFHVSVCLKDNCPYRIIDNSQKEAVTVYFCCSCTN*

>Ath:AT2G01730

MAIDCLVLGAGQEIGKSCVVVTINGKKIMFDCGMHMGCDDHNRYPNFSLISKSGDFDNAISCIIITHFHMDHVGALPYFTEVCGYNGPIYMSYPTKALSPLMLEDYRRVMVDRRGEEELFTTTHIANCMKKVIAIDLKQTIQVDEDLQIRAYYAGHVLGAVMVYAKMGDAAIVYTGDYNMTTDRHLGAAKIDRLQLDLLISESTYATTIRGSKYPREREFLQAVHKCVAGGGKALIPSFALGRAQELCMLLDDYWERMNIKVPIYFSSGLTIQANMYYKMLISWTSQNVKEKHNTHNPFDFKNVKDFDRSLIHAPGPCVLFATPGMLCAGFSLEVFKHWAPSPLNLVALPGYSVAGTVGHKLMAGKPTTVDLYNGTKVDVRCKVHQVAFSPHTDAKGIMDLTKFLSPKNVVLVHGEKPSMMILKEKITSELDIPCFVPANGETVSFASTTYIKANASDMFLKSCSNPNFKFSNSTQLRVTDHRTADGVLVIEKSKKAKIVHQDEISEVLHEKNHVVSLAHCCPVKVKGESEDDDVDLIKQLSAKILKTVSGAQIHESENCLQVASFKGSLCLKDKCMHRSSSSSSEAVFLCCNWSIADLELGWEIINAIKLNH*

>Cre:Cre12.g503050.t1.2

MKLICAGVGASRCLVLRFRKANILLDCAVPLDSALYAAAGAGEGSAGGDADTAVLGGSPGPHPQSPTTGPAGSGLLDLTPFLPLLHTLELHAALISSAEALLALPLLLTQPLPEPAPGTQHLERVQKPEPQTHPPAITTVNGDVHDVAAAAPEGSVGPRSTGRSNGAGIGSPQRGKGGEAVGAAEWVPGSGAAWHHVGFLRGPIYATQAAIDAAEQLVAERWAAQRAAAAAAAAAARNGTSGQGFKTHTEAAQEEQAYNHKDTGDLRTNSKGAAGLPSAADLGLWDLPDRQVGLLAGSCWRRRPSLAAVRYVLDRVRPVRYGQVVPLDDYELTATPYPCGSGFGHAAWQVMDGAERCRTVLYLPNAAPTHAFAPPLPLALLRQPDALLLAPHMLAAAGPAPGPPPPAPPPPPARPGLAGIKEAVLGAVMGGGVALLPVHPTGDTAWELLEALAASLASADLADSVPLVYVGPRARTSLALASVSLEALGPDRRAAVYRPAHPFAFDTLLKSGKLVVGGSLADSEVRRRLGPEQHGQQAEEQRPRPAVVLVSADSLLHPGDTWELLRRLGPDPCSVLILPHAAAPAALRGIRRIYAQAAAAAAADHASQSSGPGSLPANPRLAEPPPQQPLRMRLLHLPLHGAGASPGPSPAIMLELLQLLQPRHLLLSSRDHELLQQAALMRQQQHLLQLPPQPQPQGGSAALPPHVPAPAIQLARESVVPYGWLSQVAVSLPRNVHSALISPDLLARLQWLGAGPGLQVARLNCVLVFRGGAWHADPVPGSATSADSVAAAVATAAGGAVAADQLLLLAAPGQAAAQAASLGSGAGGAGAGAGGDAAAAPAVKVEAASWAPGPELGPLLAPLAERGVTHVGVEADGEVTRLVVSGTDASLELRRGSAHIHTACPVLRQVLTDCLMRQLTAI*

>Bdi:Bradi4g29760

MAIECLVLGAGQEVGKSCVVVTIGGKRIMFDCGMHMGYHDCNRYPDFARILAAAPETTDFTSAISCVIITHFHLDHIGALPYFTEVCGYHGPIYMTYPTKALAPLMLEDYRKVMVDQRGEEEQYSYEDILRCMKKVIPVDLKQTIQVNRDLVIRAYYAGHVLGAAMVYAKVGDAAMVYTGDYNMTPDRHLGAAQIERLKLDLLITESTYAKTIRDSKHAREREFLKAVHKCVSEGGKVLIPTFALGRAQELCILLDDYWERMNLKIPIYFSAGLTIQANMYYKMLIGWTSQKIKDSYTVQNPFDFKHVCHFERSFINDPGPCVLFATPGMISGGFSLEVFKRWATSDKNLVTLPGYCVAGTIGHKLMSGKPTRIDIDKDTHVDVRCQIHQLSFSPHTDSKGIMDLTEFLSPNHVILVHGEKPQMAFLKDRIESELGMSCYYPANNETVSIPTTQNMKISATEKFITNCAALQARDSLQKSDLISSIHLSGVNEDERLAEGILLMEKSKAPKILCEDEFLKLLGTDRHSVQFEPLLPSRIEEAEAAIVDDLTSE*

>Osa:LOC_Os09g23380

MAIECLVLGAGQEVGKSCVVVTFGGGKRVMFDCGMHMGHRDSRRYPDFDRLLADGAADYTAAISCVVITHFHLDHIGALPYFTEVCGYHGPVYMTYPTKALAPLMLEDYRKVMVDHRGEEEQYSYEDILRCMRKVIPLDLKQTIQVDKDLSIRAYYAGHVLGAAMIYAKVGDAAIVYTGDYNMTPDRHLGAAQIDRLKLDLLITESTYAKTVRDSKHAREREFLKAVHKCVSGGGKVLIPAFALGRAQELCILLDDYWERMNLKIPIYFSAGLTIQANMYYKMLIGWTSQKIKNSYTVHNPFDFKHVCHFERSFINNPGPCVLFATPGMISGGFSLEVFKKWAPSEKNLVTLPGYCVAGTIGHKLMSGKPTRIDIDKDTHIDVRCQIHQLSFSPHTDSKGIMDLTEFLSPSHVILVHGEKPQMAFLKERIESELGMQCCYPANNETVSIPTSQNLKINATEKFIVSFCMDETENDPQKQNLNFGGDMPQGCRTEGVAEGVLLMEKSKTPKILREDELLHSLGMETHFVHFEPLHPSSIEVKHTGESAVQQSSLEHLDCE*

>Sbi:Sb01g000850

MASSAAAPSGAPPAGKRPASSGREGDQMVITPLGAGSEVGRSCVHMTFKGRTVLFDCGIHPAYSGMAALPYFDEIDPSTIDVLLITHFHLDHAASLPYFLEKTTFKGRVFMTHATKAIYRLLLSDYVKVSKVSVEDMLYDENDIARSMEKIEVIDFHQTLEVNGIRFWCYTAGHVLGAAMFMVDIAGVRILYTGDYSREEDRHLRAAELPQFSPDICIIESTYGVQQHQPRIVREKRFTEVIHNTVSQGGRVLIPAFALGRAQELLLILDEYWSKHPELHKIPIYYASPLAKRCMAVYQTYINSMNERIRNQFAQSNPFHFKHIESLNSIDNFHDVGPSVVMASPGGLQSGLSRQLFDKWCTDKKNACVIPGYVVEGTLAKTIINEPREVTLANGLTAPLHMSVHYISFSAHADFPQTSNFLDELRPPNIILVHGEANEMSRLKQKLKTQFDGTNIVSPKNCQSVEMYFTCEKMAKTIGRLAEKVPEGGESSGGLLVKKGFTYQIMAPEDLRVFTQLSTANITQRIAVPYSGSFEVIKYRLKQIYESVESATEESDVPALIVHERVTVRLDSESYVTLQWSSDPISDMVSDSVVAMILNIGREGPKVVPVEEAVKTKEETEKVAQKVVYALMTSLFGDVKVTAEGKFVISVDGNVAHLDGMSGDVKCENATLKERIKTAFRRIQSAVRPIPLSAS*

>Smo:234972

MASSASTQRQRKDSMARNEGEKMEIMPLGAGSEVGRSCCHMTYKGKTILFDCGIHPGYTGMAALPYFDEIDPSTIDVLLVTHFHLDHAASLPYFLEKTTFKGRVFMTHATKAIYKLLLTDYVKISKGSVEDMLYDEQDVLKTMDKIEVIDFHQTMEVNGIRFWCYTAGHVLGAAMFMVDIAGIRVLYTGDYSREEDRHLKAAEMPEFSPDVCIIESTYGVQIHQPRHVREKRFTETIAQTVSHGGRVLIPAFALGRAQELLLILDEYWEAHPELQHIPIYYASPLAKKCMAVYQTYINSMNDKIKSQYENSNPFNFKHISPLKSIEQFEDVGPSIVMASPSGLQSGLSRQLFDRWCQDRKNACVIPGYVVEGTLAKTILNEPKEVALVSGLVVPLNMRVVYISFSAHADFAQTSAFLGELRPPHIVLVHGEQNEMGRLKVKLQAQFAEQNVKINSPKNCQPVEFFFKGEKVAKAVGRLAEKTPDEGGAVSGLLIKKSFTYQLMAPDDLHVYTQLSRGSIQQRLSVPYDGAFVVLRHRIEQMYDGVEHVTKAESQTLRIHDKVTISQESNEHVVLQWVSDPVSDMIADSIIAIITKMDPRLLTDANGLPKIEEDLTVVQSLLTSLFGDVKVDEEGKKLVISVDGVAATVDYTSRSVECDDDNLKERIKSALHRMHNVLHPLAIES*

>Aly:475225

MASSSASLKRREQPISRDGDQLIVTPLGAGSEVGRSCVYMSFRGKNILFDCGIHPAYSGMAALPYFDEIDPSSIDVLLITHFHIDHAASLPYFLEKTTFNGRVFMTHATKAIYKLLLTDYVKVSKVSVEDMLFDEQDINKSMDKIEVIDFHQTVEVNGIKFWCYTAGHVLGAAMFMVDIAGVRILYTGDYSREEDRHLRAAELPQFSPDICIIESTSGVQLHQSRHIREKRFTDVIHSTVAQGGRVLIPAFALGRAQELLLILDEYWANHPDLHNIPIYYASPLAKKCMAVYQTYILSMNDRIRNQFANSNPFVFKHISPLNSIDDFNDVGPSVVMATPGGLQSGLSRQLFDSWCSDKKNACIIPGYMVEGTLAKTIINEPKEVTLMNGLTAPLNMQVHYISFSAHADYAQTSTFLKELMPPNIILVHGEANEMMRLKQKLFTEFPDGNTKIMTPKNCESVEMYFNSEKLAKTIGRLAGKTPDVGDTVSGILVKKGFTYQIMAPDELHVFSQLSTATVTQRITIPFVGAFGVIKHRLEKIFESVESSTDEESGLPALKVHERVTVKQESEKHISLQWSSDPISDMVSDSIVALILNISREVPKIVVEEEDAVKSEEENGKKVEKVIYALLVSLFGDVKLGENGKLVIRVDGNVAQLDKESGEVESEHSGLKERVRVAFERIQSAVKPIPLSAS*

>Gma:Glyma08g20140

MMDCGVKRRESVREEDDEVMIVTPLGAGNEVGRSCVYMSYKGKSILFDCGIHLGFSGMSALPYFDEIDPSTLDVLLITHFHLDHAASLPYFLEKTTFRGRVFMTYATKAIYKLLLSDFVKVSKVSVEDMLFDEQDINRSMDKIEVIDFHQTVEVNGIRFWCYAAGHVLGAAMFMVDIAGVRVLYTGDYSREEDRHLRAAEIPQFSPDVCIIESTYGVQHHQPRHTREKRFTDVIHSTISQGGRVLIPAYALGRAQELLLILDEYWANHPELHNIPIYYASPLAKKCLTVYETYTLSMNDRVQNAKSNPFSFKHISALSSIEVFKDVGPSVVMASPGGLQSGLSRQLFDKWCSDKKNTCVLPGFVVEGTLAKTIMTEPKEVTLMNGLSAPLNMQVHYISFSAHADSAQTSAFLEELNPPNIILVHGEANQMGRLKQKLTSQFADRNTKILTPKNCQSVEMHFNSQKMAKTIGRLAEKTPEVGETVSGLLVKKGFTYQIMAPDDLHVFSQLSTTNITQRITVPYSGAFSFIQHRLKRIYESVEQSVDEESGVPELQVHECVTVKHEAEKHISLHWTSDPISDMVSDSIVALILNINRDVPKIMAESDVIKIEEENKKKAEKVMHALLVSLFGDVKAGENGKLIINIDGNVAVLNKESGEVESENEGLKERVRAAFQRIQSSVKPIPLSAP*

>Gma:Glyma12g29040

MNNCTAKRRENARSSREEDQLIVTPLGAGNEVGRSCVYMSYKGKTVLFDCGIHPAYSGMAALPYFDEIDPSTVDVLLITHFHLDHAASLPYFLEKTTFRGRVFMTYATKAIYKLLLSDFVKVSKVSVEDMLFDEQDINRSMDKIEVIDFHQTVEVNGIRFWCYTAGHVLGAAMFMVDIAGVRVLYTGDYSREEDRHLRAAETPQFSPDVCIIESTYGVQHHQPRHTREKRFTDVIHSTISQGGRVLIPAFALGRAQELLLILDEYWANHPELQNIPIYYASPLAKKCLTVYETYTLSMNDRIQNAKSNPFSFKHVSALSSIEVFKDVGPSVVMASPGGLQSGLSRQLFDMWCSDKKNSCVLPGYVVEGTLAKTIINEPKEVTLMNGLTAPLNMQVHYISFSAHADSAQTSAFLEELNPPNIILVHGEANEMGRLKQKLISQFADRNTKILTPKNCQSVEMYFNSQKMAKTIGKLAEKTPEVGETVSGLLVKKGFTYQIMAADDLHVFSQLSTANITQRITIPYSGAFNVIQHRLKQIYESVAQSVDEESGVPTLQVHECVTVKHESEKHVSLHWASDPMSDMVSDSIVALVLNINRDVPKIVNESDAIKIEEENEKKAEKVMQALLVSLFGDVKVGENGKLIINIDGNVAELNKESGEVESENEGLKERVRTAFRRIQSSVKPIPVSAP*

>:GSVIVT01023351001

MASTGPPQSLKRPDSSLTREGDQLIITPLGAGNEVGRSCVYMSYKGKTILFDCGIHPAYSGMAALPYFDEIDPSTIDVLLVTHFHLDHAASLPYFLEKTTFKGRVFMTHATKAIYKLLLSDYVKVSKVSVEDMLYDEQDILRSMDKIEVIDFHQTLEVNGIRFWCYTAGHVLGAAMFMVDIAGVRVLYTGDYSREEDRHLRAAEIPQFSPDICIIESTYGVQLHQPRHVREKRFTDVIHSTISQGGRVLIPAFALGRAQELLLILDEYWSNHPELHNIPIYYASPLAKRCMAVYQTYINSMNERIRNQFANSNPFDFKHISPLKSIENFNDVGPSVVMASPSGLQSGLSRQLFDMWCSDKKNACVIPGYVVEGTLAKTIINEPKEMGRLKQKLITQFADRNTKIISPKNCQSVEMYFNSEKMAKTIGRLAEKTPGVGETVSGLLVKKGFTYQIMAPDDLHVFSQLSTANVTQRITIPYTGAFGVIKHRLKQIYESVESLPDEESEVPAFRVHERVTVKHESEKHISLHWTSDPISDMVSDSIVALVLNISREIPKVVVESEAIKTEEENGKKAEKVIHALLVSLFGDVKLGENGNLVISVDGNVAHLDKQTPSLRKILNIDPPQSQARSHTSTFSSLYLLFGNAREIFLSDFCDSSELHKCINLVEQRYIPRNYGFQCIT*

>:GSVIVT01023380001

MASTGPSQSLKRPDSSLTRGDQLIITPLGAGNEVGRSCVYMSYKGKTILFDCGIHPAYSGMAALPYFDEIDPSTIDVLLVTHFHLDHAASLPYFLEKTTFKGRVFMTHATKAIYKLLLSDYVKVSKVSVEDMLYDEQDILRSMDKIEVIDFHQTLEVNGIRFWCYTAGHVLGAAMFMVDIAGVRVLYTGDYSREEDRHLRAAEIPQFCPDICIIESTYGVQLHQPRHVREKRFTDVIHSTISQGGRVLIPAYALGRAQELLLILDEYWSNHPELHNVPIYYASPLAKRCMAVYQTYINSMNERIRNQFANSNPFDFKHISPLKSIENFNDVGPSVVMASPGGLQSGLSRQLFDMWCSDKKNACVIPGYVVGGTLAKTIINEPKENCQSVEMYFNSEKMAKTIGRLAEKTPEVGETVSGLLVKKGFTYQIMAPDDLHVFWQLSTANVTQRITIPYTGAFGKHISLHWTSDPISDMVSDSIVALVLNISLEIPKVIVESEAIKTEEENGKKAEKVIHALLVSLFGDVKLEGNGNLVISVDGNVVHLDKQSGNVESENEGLKERVRVAFQRIQNAVKPIPPSVS*

>Ppa:Pp1s77_275V6

MSKRKDTATVITREGDKLEVTPLGAGNEVGRSCVYMTYKGKTVMFDCGIHPGYSGMAALPYFDEIDPISIDVLLVTHFHLDHCASLPYFLEKTNFKGRVFMTHATKAIYKLLLSDFVKISKVSVDDMLYDEHDIARTMEKIEVIDFHQTMEVNGIRFWCYTAGHVLGAAMFMVDIAGMRVLYTGDYSCEEDRHLRAAEMPRFSPDVCIIESTYGVQIHQPRIMRERRFTDTVAQTVSQGGKVLIPAFALGRAQELLLILDEYWEAHPELQHIPIYYASPLAKKCMAVYQTYINAMNERIQKQFEVSNPFDFKHIQPLKNIDEFDDIGPAVVMASPGGLQSGLSRQLFDIWCQDKKNSCVIPGYVVEGTPAKAIMNEPKEVTLLSGLVVPLNMRVHYISFSAHADFTQTSAFLHELRPPNIILVHGEANEMGRLKAKLTTQFAEQNVKILSPKNCQTVEMFFKGEKIAKAVGRLAEKPAKEGDIVSGLLVRKGFTYQLMAPDDLHSFTQLSTGSVMQRQSVPYKGTFIVLRHRLQQMYEQVEAVAKADSPILKVHGNLTITHEGADQVILQWVSDPISDMVADSVVAMILKLDSQSMFAVGGERSKETRLEKNEIKIVHSLLVSLFGDVTLDEKQQTLTVNVDGTVATVDHVKKGIECKDENLKERIKVALRRIQTALYPLDA*

>Ppa:Pp1s23_196V6

MSKRKDPTTVVSREGDKLEVTPLGAGNEVGRSCVYMTYKGKTVMFDCGIHPGYSGMAALPYFDEIDPISIDVLLVTHFHLDHCASLPYFLEKTNFKGRVFMTHATKAIYKLLLSDFVKISKVSVDDMLYDEHDIARTMEKIEVIDFHQTMEVNGIRFWCYTAGHVLGAAMFMVDIAGMRVLYTGDYSCEEDRHLRAAEMPHFSPDVCIIESTYGVQIHQPRIMRERRFTDTVAQTVSQGGKVLIPAFALGRAQELLLILDEYWEAHPELQHIPIYYASPLAKKCMAVYQTYINAMNDRIQKQFEVSNPFDFKHIQPLKNIDGFDDIGPAVVMASPGGLQSGLSRQLFDIWCQDKKNSCIIPGYVVEGTLAKAIMNEPKEVTLLSGLVVPLNMRVHYISFSAHADFTQTNAFLHELRPPNIILVHGEANEMGRLKAKLITQFAEQNVKILSPKNCQTVEMFFKGEKIAKAVGRLAEKSAKEGDIVSGLLVRKGFTYQLMAPDDLHSFTQLSTGSVMQRQSVPYKGTFTVLRHRLQQMYEQVEVVTKADSPTLKVHGNLTVTHEGADYVILQWVSDPISDMVADSVVAMILKLDSQSMFAVGGGRAKEARLEKEEIKIVHSLLVSLFGDVTLDEEHQSLTVNVDGIEATIDHVKRGIECKDENLKDRIKVALRRIQTALYPLDA*

>Ptr:POPTR_0017s11270

MLSACSFHLDHAASLPYFLEKTTFRGRVFMTHATKAIFKLLLTNYVKVSKVSVEDMLFDEKDINRSMDKIEVIDFHQTVDVNGIKFWCYTAGHVLGAAMFMVDIAGVRVLYTGDYSREEDRHLCAAEMPQFSPDICIIESTYGVQLHQPRHLREKRFTDVIHSTISLGGRVLIPAFALGRAQELLLILDEYWSNHPELHNIPIYYASPLAKKCMTVYQTYILSMNERIRNQFANSNPFKFKHISPLNSIEDFTDVGPSVVMASPSGLQSGLSRQLFDMWCSDKKNACVIPGYVVEGTLAKTIINEPKEVQLMNGLTAPLNMQVHYISFSAHADYAQTSTFLKELMPPNIILVHGEANEMGRLKQKLITEFTDGNTKIITPKNCQSVEIYFNSEKMAKTIGKLAERTPNVGETVSGILVKKGFTYQIMAPGDLHVFSQLSTGNITQRITIPFSGAFGVIKHRLEQIYESVESGTYEEESGSPTLQVHELVTVKQESDRHISLHWTADPIIDMVSDSIVALVLNISREVPKVIVESEDIKSKEEKEKKAEKVIYALLVSLFGDVKLGGNGKLVIRVDGNVAELDKQSGDVESEHEGLKERVRTAFRRIQSAVRPIPLPAS*

>Ptr:POPTR_0017s11240

MASTGQSQSLKRRDAPVTREGGDQLTLTPLGAGNEVGRSCVYMSFKGKTVLFDCGIHPAYSGMAALPYFDEIDPSTIDVLLVTHFHLDHAASLPYFLEKTTFRGRVFMTHATKAIYKLLLTDYVKVSKVSVEDMLFDEKDINRSMDKIEVIDFHQTLDVNGIKFWCYTAGHVLGAAMFMVDIAGVRVLYTGDYSREEDRHLRAAEMPQFSPDICIIESTYGVQLHQPRHLREKRFTDVIHSTISLGGRVLIPAFALGRAQELLLILDEYWANHPELHNIPIYYASPLAKKCMTVYQTYILSMNERIRNQFANSNPFKFKHISPLNSIEDFSDVGPSVVMASPGGLQSGLSRQLFDMWCSDKKNACVLPGYVVEGTLAKTIINEPKEVQLMNGLTAPLNMQVHYISFSAHADYAQTSTFLKELMPPNIILVHGEANEMGRLKQKLITEFADGNTKIITPKNCQSVEMYFNSEKMAKTIGKLAERTPDVGETVSGILVKKGFTYQIMAPGDLHVFSQLSTGNITQRITIPFSGAFGVIKHRLEQIYESVESGTDEESGFPTLQVHELVTVKQESDRHISLHWTADPISDMVSDSIVALVLNISREVPKVIVESEDIKSEEENEKKAEKVIYALLVSLFGDVKLGENGKLVLRVDGNVAELDKQSGDVESENEGLKERVRTAFRRIRSAVRPIPLPLPAPAS*

>Ptr:POPTR_0017s11250

MASTGQSQSLKRRDAPVTREGGDQLTLTPLGAGNEVGRSCVYMSFKGKTVLFDCGIHLAYSGMAALPYFDEIDPSTIDVLLVTHFHLDHAASLPYFLEKTTFRGRVFMTHATKAIYKLLLTDYVKVSKVSVEDMLFDEKDINRSMDKIEVIDFHQTVDVNGIKFWCYTAGHVLGAAMFMVDIAGVRVLYTGDYSREEDRHLRAAEMPQFSPDICIIESTYGVQLHQPRHIREKRFTDVIHSTISLGGRVLIPAFALGRAQELLLILDEYWSNHPELHNIPVYYASPLAKKCMTVYQTYILSMNERIRNQFADSNPFKFKHISPLNSIEDFTDVGPSVVMATPGGLQSGLSRQLFDMWCSDKKNACVIPGFLVEGTLAKTIINEPKEVQLMNGLTAPLNMQVHYISFSAHADYAQTSTFLKELMPPNIILVHGEANEMGRLKQKLITEFTDGNTKIITPKNCQSVEMYFNSEKMAKTTGKLAERTPDVGETVSGILVKKGFTYQIMAPEDLHVFSQLSTGNITQRITIPFSGAFGVIKHRLEQIYESVESGTDEESGSPTLQVHELVTVKQESDRHISLHWTADPISDMVSDSIVALVLNISREVPKVIVESEDIKSEEENEKKAEKVIYAFLVSLFGDVKLGENGKLVISVDGNVAELDKQSGDVESENEGLKERVRTAFRRIQSAVRPIPLPAS*

>Ath:AT1G61010

MASSSTSLKRREQPISRDGDQLIVTPLGAGSEVGRSCVYMSFRGKNILFDCGIHPAYSGMAALPYFDEIDPSSIDVLLITHFHIDHAASLPYFLEKTTFNGRVFMTHATKAIYKLLLTDYVKVSKVSVEDMLFDEQDINKSMDKIEVIDFHQTVEVNGIKFWCYTAGHVLGAAMFMVDIAGVRILYTGDYSREEDRHLRAAELPQFSPDICIIESTSGVQLHQSRHIREKRFTDVIHSTVAQGGRVLIPAFALGRAQELLLILDEYWANHPDLHNIPIYYASPLAKKCMAVYQTYILSMNDRIRNQFANSNPFVFKHISPLNSIDDFNDVGPSVVMATPGGLQSGLSRQLFDSWCSDKKNACIIPGYMVEGTLAKTIINEPKEVTLMNGLTAPLNMQVHYISFSAHADYAQTSTFLKELMPPNIILVHGEANEMMRLKQKLLTEFPDGNTKIMTPKNCESVEMYFNSEKLAKTIGRLAEKTPDVGDTVSGILVKKGFTYQIMAPDELHVFSQLSTATVTQRITIPFVGAFGVIKHRLEKIFESVEFSTDEESGLPALKVHERVTVKQESEKHISLQWSSDPISDMVSDSIVALILNISREVPKIVMEEEDAVKSEEENGKKVEKVIYALLVSLFGDVKLGENGKLVIRVDGNVAQLDKESGEVESEHSGLKERVRVAFERIQSAVKPIPLSAS*

>Cre:Cre12.g498050.t1.1

MAATAKRKIDEGPADGEPDTSTHAHIIPLGAGSEVGRSCIIFKYQDKTVMFDCGIHPAFKGMDSLPLLDEIDIDTVDVALITHFHLDHCAAVPYLLRKTRFKGRIFMTHPTKAIYYSLLRDLAKGSKHSSEEALFNEDDLEASMQRIEVVDFYQTIEVAGMQITPYRAGHVLGAAMFLVEVAGCRCLYTGDYSRLPDRHLPAADIPPVKPHIVIVESTYGTSRHLPRLQREQLLLDTIRNTINRGGRVIMPVVALGRAQELLLLLDEYWEAHKSELSGIPIYQASSMMSKALGVYQTYVESLNDDIKRVFHERNPFKFRHVQTLKNPAHFISDYSGPCVIMATPSGLQSGASRDFFEAWCEDSRNTCIICDFAVQGTLAKEILGGPSSITTREGRRVPLRIAVHNISFSAHADYDQTSGFLDAVRPPHVVLVHGEYGEMRKLAKALKDGAKAAGVAREVYTPILAQTVAVEHKPDRSVRLQGRLGEKPPREGAAVRGVLVRQGGGFATQLLAPSDLPRYTKLLKGSVTQRQAISVDVPFTAIRLALEVMFEGVEGAGTLPVATAPDAEGGKGGESLAVVVGELVTVRYVPADDASGVVSHVVLEWEGGRQGDMVADAVVAVVLQSIGEPPEATSAESAMLAARAAGDEAAAAAAELHLIGALLRGQFGPAAVDEAGGLVRLDVDGVEAAVDYRAGKVVCGEPGLRARIEKSLDRLAAAIRPAPLDRPHD*

>Bdi:Bradi1g01140

MASVATAPPAGKRPTSGGREGDQMVITPLGAGSEVGRSCVHMTFKGRTVLFDCGIHPAYSGMAALPYFDEIDPSAIDVLLVTHFHLDHAASLPYFLEKTTFKGRVFMTHATKAIYRLLLSDYVKVSKVSVEDMLFDEQDIIRSMDKIEVIDFHQTLEVNGIRFWCYTAGHVLGAAMFMVDIAGVRILYTGDYSREEDRHLKAAEIPQFSPDVCIVESTYGVQQHQPRHVREKRFTDAIHNTVSQGGRVLIPAFALGRAQELLLILDEYWSNHPELHKIPIYYASPLAKKCMAVYQTYINSMNERIRNQFAQSNPFHFKHIEPLNSIDNFHDVGPSVVMASPGTLQSGLSRQLFDKWCTDKKNTCVIPGFVIEGTLSKTIINEPREVTLANGLTAPLHMQIFYISFSAHADFPQTSAFLDELRPPNIILVHGEANEMGRLKQKLITQFDGTNIKIVSPKNCQSVEMYFSSEKMAKTIGRLAEKVPEVGESVSGLLVKKGFTYQIMAPEDLRVYTQLSTANITQRIAVPYSGSFEVIKYRLKQIYESVESCPEEPDVPTLIVHERVTIRLESESYVTLQWSSDPISDMVSDSVVAMILNIGREGPKVVPVEEAEKTQEETEKVARKVVYALMVSLFGDVKVAEGGKFVISVDGDVAHLDGSSGDVECENAALKERIKTAFRRIQGAVRPIPLSSS*

>Bdi:Bradi1g01200

MASVAPAGKRPASGGREGDQMVITPLGAGSEVGRSCVHMTFKGRTVLFDCGIHPAYSGMAALPYFDEIDPSAIDVLLVTHFHLDHAASLPYFLEKTTFKGRVFMTHATKAIYRLLLSDYVKVSKVSVEDMLFDEQDIIRSMDKIEVIDFHQTLEVNGIRFWCYTAGHVLGAAMFMVDIAGVRILYTGDYSREEDRHLKAAEIPQFSPDVCIVESTYGVQQHQPRHVREKRFTDAIHNTVSQGGRVLIPAFALGRAQELLLILDEYWSNHPELQKIPIYYASPLAKKCMAVYQTYINSMNERIRNQFAQSNPFHFKHIEPLNSIDNFHDVGPSVVMASPGSLQSGLSRQLFDKWCTDKKNTCVIPGYVIEGTLAKTIINEPREVTLANGLTAPLHMQIFYISFSAHADFPQTSGFLDELRPPNIILVHGEANEMGRLKQKLITQFDGTNIKIVSPKNCQSVEMYFSSEKMAKTIGRLAEKVPDVGEPVSGLLVKKGFTYQIMAPEDLRVYTQLSTANVTQQIAVPYSGSFEVIKYRLKQIYESVESCPEEPEVPTLIVHERVTIRLESESYVTLQWSSDPISDMVSDSVVAMILNIGREGPKVVPVEEAEKTQDETEKVARKVVYALMVSLFADVKVAEEGKLVISVDGDVAHLDGSSGDVECENAALKERIKTAFRRIQGAVRPIPLSSS*

>Osa:LOC_Os03g63590

MASSVAAAAAPTAGGPPGKRQASGGREGDQLIITPLGAGNEVGRSCVYMSFKGRTVLFDCGIHPAYSGMAALPYFDEIDPSTIDVLLITHFHLDHAASLPYFLEKTTFKGRVFMTHATKAIYRLLLSDYVKVSKVSVEDMLFDEQDILRSMDKIEVIDFHQTLEVNGIRFWCYTAGHVLGAAMFMVDIAGVRVLYTGDYSREEDRHLKAAELPQFSPDICIIESTYGVQQHQPRHVREKRFTDVIHTTVSQGGRVLIPAFALGRAQELLLILDEYWANHPELHKIPIYYASPLAKKCMAVYQTYINSMNERIRNQFAQSNPFHFKHIESLNSIDNFHDVGPSVVMASPGGLQSGLSRQLFDKWCTDKKNSCVIPGYVVEGTLAKTIINEPREVTLANGLTAPLHMQVHYISFSAHADFPQTSTFLDELQPPNIVLVHGEANEMSRLKQKLISQFDGTNIKVVNPKNCQSVEMYFSSEKMAKTIGRLAEKVPEAGESVNGLLVKKGFTYQIMAPEDLRVYTQLSTANITQRIAVPYSGSFEVIKYRLKQIYESVESSTEESDVPTLIVHERVTIRLESESYVTLQWSSDPISDMVSDSVVAMVLNIGREGPKVVPVEEAVKTQEETERVAQKVVYALMVSLFGDVKVAEEGKLVISVDGQVAHLDGRSGGVECENATLRERIKTAFRRIQGAVRPIPLISS*

>NP_651658.1[Drosophilamelanogaster]

MTSIIKLHTISGAMDESPPCYILQIDDVRILLDCGWDEKFDANFIKELKRQVHTLDAVLLSHPDAYHLGALPYLVGKLGLNCPIYATIPVFKMGQMFMYDLYMSHFNMGDFDLFSLDDVDTAFEKITQLKYNQTVSLKDKGYGISITPLNAGHMIGGTIWKIVKVGEEDIVYATDFNHKKERHLSGCELDRLQRPSLLITDAYNAQYQQARRRARDEKLMTNILQTVRNNGNVLIAVDTAGRVLELAHMLDQLWKNKESGLMAYSLALLNNVSYNVIEFAKSQIEWMSDKLTKAFEGARNNPFQFKHIQLCHSLADVYKLPAGPKVVLASTPDLESGFTRDLFVQWASNANNSIILTTRTSPGTLAMELVENCAPGKQIELDVRRRVDLEGAELEEYLRTQGEKLNPLIVKPDVEEESSSESEDDIEMSVITGKHDIVVRPEGRHHSGFFKSNKRHHVMFPYHEEKVKCDEYGEIINLDDYRIADATGYEFVPMEEQNKENVKKEEPGIGAEQQANGGIVDNDVQLLEKPTKLISQRKTIEVNAQVQRIDFEGRSDGESMLKILSQLRPRRVIVIHGTAEGTQVVARHCEQNVGARVFTPQKGEIIDVTSEIHIYQVRLTEGLVSQLQFQKGKDAEVAWVDGRLGMRVKAIEAPMDVTVEQDASVQEGKTLTLETLADDEIPIHNSVLINELKLSDFKQTLMRNNINSEFSGGVLWCSNGTLALRRVDAGKVAMEGCLSEEYYKIRELLYEQYAIV

>CPSF100[Homosapiens]

MTSIIKLTTLSGVQEESALCYLLQVDEFRFLLDCGWDEHFSMDIIDSLRKHVHQIDAVLLSHPDPLHLGALPYAVGKLGLNCAIYATIPVYKMGQMFMYDLYQSRHNTEDFTLFTLDDVDAAFDKIQQLKFSQIVNLKGKGHGLSITPLPAGHMIGGTIWKIVKDGEEEIVYAVDFNHKREIHLNGCSLEMLSRPSLLITDSFNATYVQPRRKQRDEQLLTNVLETLRGDGNVLIAVDTAGRVLELAQLLDQIWRTKDAGLGVYSLALLNNVSYNVVEFSKSQVEWMSDKLMRCFEDKRNNPFQFRHLSLCHGLSDLARVPSPKVVLASQPDLECGFSRDLFIQWCQDPKNSIILTYRTTPGTLARFLIDNPSEKITEIELRKRVKLEGKELEEYLEKEKLKKEAAKKLEQSKEADIDSSDESDIEEDIDQPSAHKTKHDLMMKGEGSRKGSFFKQAKKSYPMFPAPEERIKWDEYGEIIKPEDFLVPELQATEEEKSKLESGLTNGDEPMDQDLSDVPTKCISTTESIEIKARVTYIDYEGRSDGDSIKKIINQMKPRQLIIVHGPPEASQDLAECCRAFGGKDIKVYMPKLHETVDATSETHIYQVRLKDSLVSSLQFCKAKDAELAWIDGVLDMRVSKVDTGVILEEGELKDDGEDSEMQVEAPSDSSVIAQQKAMKSLFGDDEKETGEESEIIPTLEPLPPHEVPGHQSVFMNEPRLSDFKQVLLREGIQAEFVGGVLVCNNQVAVRRTETGRIGLEGCLCQDFYRIRDLLYEQYAIV

>Cft2p[Saccharomycescerevisiae]

MTYKYNCCDDGSGTTVGSVVRFDNVTLLIDPGWNPSKVSYEQCIKYWEKVIPEIDVIILSQPTIECLGAHSLLYYNFTSHFISRIQVYATLPVINLGRVSTIDSYASAGVIGPYDTNKLDLEDIEISFDHIVPLKYSQLVDLRSRYDGLTLLAYNAGVCPGGSIWCISTYSEKLVYAKRWNHTRDNILNAASILDATGKPLSTLMRPSAIITTLDRFGSSQPFKKRSKIFKDTLKKGLSSDGSVIIPVDMSGKFLDLFTQVHELLFESTKINAHTQVPVLILSYARGRTLTYAKSMLEWLSPSLLKTWENRNNTSPFEIGSRIKIIAPNELSKYPGSKICFVSEVGALINEVIIKVGNSEKTTLILTKPSFECASSLDKILEIVEQDERNWKTFPEDGKSFLCDNYISIDTIKEEPLSKEETEAFKVQLKEKKRDRNKKILLVKRESKKLANGNAIIDDTNGERAMRNQDILVENVNGVPPIDHIMGGDEDDDEEEENDNLLNLLKDNSEKSAAKKNTEVPVDIIIQPSAASKHKMFPFNPAKIKKDDYGTVVDFTMFLPDDSDNVNQNSRKRPLKDGAKTTSPVNEEDNKNEEEDGYNMSDPISKRSKHRASRYSGFSGTGEAENFDNLDYLKIDKTLSKRTISTVNVQLKCSVVILNLQSLVDQRSASIIWPSLKSRKIVLSAPKQIQNEEITAKLIKKNIEVVNMPLNKIVEFSTTIKTLDISIDSNLDNLLKWQRISDSYTVATVVGRLVKESLPQVNNHQKTASRSKLVLKPLHGSSRSHKTGALSIGDVRLAQLKKLLTEKNYIAEFKGEGTLVINEKVAVRKINDAETIIDGTPSELFDTVKKLVTDMLAKI

>Ysh1p[Saccharomycescerevisiae]

MERTNTTTFKFFSLGGSNEVGRSCHILQYKGKTVMLDAGIHPAYQGLASLPFYDEFDLSKVDILLISHFHLDHAASLPYVMQRTNFQGRVFMTHPTKAIYRWLLRDFVRVTSIGSSSSSMGTKDEGLFSDEDLVDSFDKIETVDYHSTVDVNGIKFTAFHAGHVLGAAMFQIEIAGLRVLFTGDYSREVDRHLNSAEVPPLSSNVLIVESTFGTATHEPRLNRERKLTQLIHSTVMRGGRVLLPVFALGRAQEIMLILDEYWSQHADELGGGQVPIFYASNLAKKCMSVFQTYVNMMNDDIRKKFRDSQTNPFIFKNISYLRNLEDFQDFGPSVMLASPGMLQSGLSRDLLERWCPEDKNLVLITGYSIEGTMAKFIMLEPDTIPSINNPEITIPRRCQVEEISFAAHVDFQENLEFIEKISAPNIILVHGEANPMGRLKSALLSNFASLKGTDNEVHVFNPRNCVEVDLEFQGVKVAKAVGNIVNEIYKEENVEIKEEIAAKIEPIKEENEDNLDSQAEKGLVDEEEHKDIVVSGILVSDDKNFELDFLSLSDLREHHPDLSTTILRERQSVRVNCKKELIYWHILQMFGEAEVLQDDDRVTNQEPKVKEESKDNLTNTGKLILQIMGDIKLTIVNTLAVVEWTQDLMNDTVADSIIAILMNVDSAPASVKLSSHSCDDHDHNNVQSNAQGKIDEVERVKQISRLFKEQFGDCFTLFLNKDEYASNKEETITGVVTIGKSTAKIDFNNMKILECNSNPLKGRVESLLNIGGNLVTPLC

>NP_504822.1[Caenorhabditiselegans]

MTSIIKLKVFSGAKDEGPLCYLLQVDGDYILLDCGWDERFGLQYFEELKPFIPKISAVLISHPDPLHLGGLPYLVSKCGLTAPVYATVPVYKMGQMFIYDMVYSHLDVEEFEHYTLDDVDTAFEKVEQVKYNQTVVLKGDSGVHFTALPAGHMLGGSIWRICRVTGEDIVYCVDFNHKKERHLNGCSFDNFNRPHLLITGAHHISLPQMRRKDRDEQLVTKILRTVRQKGDCMIVIDTAGRVLELAHLLDQLWSNADAGLSTYNLVMMSHVASSVVQFAKSQLEWMNEKLFKYDSSSARYNPFTLKHVTLCHSHQELMRVRSPKVVLCSSQDMESGFSRELFLDWCSDPRNGVILTARPASFTLAAKLVNMAERANDGVLKHEDRLISLVVKKRVALEGEELLEYKRRKAERDAEETRLRMERARRQAQANESDDSDDDDIAAPIVPRHSEKDFRSFDGSENDAHTFDIMAKWDNQQKASFFKTTKKSFPMFPYIEEKVKWDDYGEVIKPEDYTVISKIDLRKGQNKDEPVVVKKREEEEEVYNPNDHVEEMPTKCVEFKNRVEVSCRIEFIEYEGISDGESTKKLLAGLLPRQIIVVHGSRDDTRDLVAYFADSGFDTTMLKAPEAGALVDASVESFIYQVALSDALLADIQFKEVSEGNSLAWIDARVMEKEAIDNMLAVGTSNLMIDDKNREEDVNDQEENGATEGEGNAEPMEIGENGSQESLAISESGKEVENGHTNDSRTKKGTKGKIRGNLILDPLPKRLIPIHQAVFVNDPKLSDFKNLLTDKGYKAEFLSGTLLINGGNCSIRRNDTGVFQMEGAFTKDYYKLRRLFYDQFAVL

>NP_495706.2[Caenorhabditiselegans]

MPDKIPEIKIVPLGAGQDVGRSCILITIGGKNIMVDCGMHMGYQDDRRFPDFSYIGGGGRLTDYLDCVIISHFHLDHCGSLPHMSEIVGYDGPIYMTYPTKAICPVLLEDYRKVQCDIKGETNFFTSDDIKNCMKKVVGCALHEIIHVDNELSIRAFYAGHVLGAAMFEIRLGDHSVLYTGDYNMTPDRHLGAARVLPGVRPTVLISESTYATTIRDSKRARERDFLRKVHECVMKGGKVIIPVFALGRAQELCILLESYWERMALNVPIYFSQGLAERANQYYRLFISWTNENIKKTFVERNMFEFKHIKPMEKGCEDQPGPQVLFSTPGMLHGGQSLKVFKKWCSDPLNMIIMPGYCVAGTVGARVINGEKKIEIDQKMHEIRLGVEYMSFSAHADAKGIMQLIRQCEPQHVMFVHGEASKMEFLKGKVEKEYKVPVHMPANGETVVISAQPKLDIRVPLEKIDRSLSLDPNPAKSECPFVAELVYDQENERLNIMSTAESEDLIKDKNCMPITLSLSEIIKGKKVNWKELSNELLLYDPHLQLKDDGIEMFDGEITILANNSDETELELIWDECREQWFKVIHKAITDLISPESSPVPPAISVKA

>NP_502553.2[Caenorhabditiselegans]

MEEEGDNSDSLCFTPLGSGQEVGRSCHLLEYKGKRVMLDCGVHPGLHGVDALPFVDFVEIENIDLLLITHFHLDHCGALPWLLQKTAFQGKCFMTHATKAIYRMLLGDYVRISKYGGPDRNQLYTEDDLEKSMAKIETIDFREQKEVNGIRFWPYVAGHVLGACQFMIEIAGVRVLYTGDFSCLEDRHLCAAEIPPITPQVLITESTYGTQTHEDRAVREKRFTQMVHDIVTRGGRCLIPAFAIGPAQELMLILDEYWESHQELHDIPVYYASSLAKKCMSVYQTFVNGMNSRIQKQIAVKNPFIFKHVSTLRGMDQFEDAGPCVVLATPGMLQSGFSRELFESWCPDTKNGCIIAGYCVEGTLAKHILSEPEEIVSLSGEKLPMRMQVGYVSFSAHTDYHQTSNFVKALKPPHLVLVHGELHEMSRLKSGIERQFQDDNIPIEVHNPRNTERLQLQFRGEKTAKVIGKLAQRVPENNETISGVLVKNNFSYSIMVPEELGSYTSLRISSLEQRMSVHYSGSLKLLIFNLQQLNDDACLIQNIKLKEISKKGSVTQAITVFQGKVNVTVYGNDHVVVVRWDSNPVYDMYADSVVAAILHAQANPVPDKYLPSNSSFPQFNTAIEGMVKHICGDDVSIVMSERGLLAQFEEDGRRLLVEGSSDGPVMMGGDDPMDDPTTSHLLQNLTEKMRQIVTTNTEVNEIDDMEC

>NP_060341.2[Homosapiens]

MPEIRVTPLGAGQDVGRSCILVSIAGKNVMLDCGMHMGFNDDRRFPDFSYITQNGRLTDFLDCVIISHFHLDHCGALPYFSEMVGYDGPIYMTHPTQAICPILLEDYRKIAVDKKGEANFFTSQMIKDCMKKVVAVHLHQTVQVDDELEIKAYYAGHVLGAAMFQIKVGSESVVYTGDYNMTPDRHLGAAWIDKCRPNLLITESTYATTIRDSKRCRERDFLKKVHETVERGGKVLIPVFALGRAQELCILLETFWERMNLKVPIYFSTGLTEKANHYYKLFIPWTNQKIRKTFVQRNMFEFKHIKAFDRAFADNPGPMVVFATPGMLHAGQSLQIFRKWAGNEKNMVIMPGYCVQGTVGHKILSGQRKLEMEGRQVLEVKMQVEYMSFSAHADAKGIMQLVGQAEPESVLLVHGEAKKMEFLKQKIEQELRVNCYMPANGETVTLPTSPSIPVGISLGLLKREMAQGLLPEAKKPRLLHGTLIMKDSNFRLVSSEQALKELGLAEHQLRFTCRVHLHDTRKEQETALRVYSHLKSVLKDHCVQHLPDGSVTVESVLLQAAAPSEDPGTKVLLVSWTYQDEELGSFLTSLLKKGLPQAPS

>NP_057291.1[Homosapiens]

MSAIPAEESDQLLIRPLGAGQEVGRSCIILEFKGRKIMLDCGIHPGLEGMDALPYIDLIDPAEIDLLLISHFHLDHCGALPWFLQKTSFKGRTFMTHATKAIYRWLLSDYVKVSNISADDMLYTETDLEESMDKIETINFHEVKEVAGIKFWCYHAGHVLGAAMFMIEIAGVKLLYTGDFSRQEDRHLMAAEIPNIKPDILIIESTYGTHIHEKREEREARFCNTVHDIVNRGGRGLIPVFALGRAQELLLILDEYWQNHPELHDIPIYYASSLAKKCMAVYQTYVNAMNDKIRKQININNPFVFKHISNLKSMDHFDDIGPSVVMASPGMMQSGLSRELFESWCTDKRNGVIIAGYCVEGTLAKHIMSEPEEITTMSGQKLPLKMSVDYISFSAHTDYQQTSEFIRALKPPHVILVHGEQNEMARLKAALIREYEDNDEVHIEVHNPRNTEAVTLNFRGEKLAKVMGFLADKKPEQGQRVSGILVKRNFNYHILSPCDLSNYTDLAMSTVKQTQAIPYTGPFNLLCYQLQKLTGDVEELEIQEKPALKVFKNITVIQEPGMVVLEWLANPSNDMYADTVTTVILEVQSNPKIRKGAVQKVSKKLEMHVYSKRLEIMLQDIFGEDCVSVKDDSILSVTVDGKTANLNLETRTVECEEGSEDDESLREMVELAAQRLYEALTPVH

>NP_651721.1[Drosophilamelanogaster]

MPDIKITPLGAGQDVGRSCLLLSMGGKNIMLDCGMHMGYNDERRFPDFSYIVPEGPITSHIDCVIISHFHLDHCGALPYMSEIVGYTGPIYMTHPTKAIAPILLEDMRKVAVERKGESNFFTTQMIKDCMKKVIPVTLHQSMMVDTDLEIKAYYAGHVLGAAMFWIKVGSQSVVYTGDYNMTPDRHLGAAWIDKCRPDLLISESTYATTIRDSKRCRERDFLKKVHECVAKGGKVLIPVFALGRAQELCILLETYWERMNLKYPIYFALGLTEKANTYYKMFITWTNQKIRKTFVHRNMFDFKHIKPFDKAYIDNPGAMVVFATPGMLHAGLSLQIFKKWAPNENNMVIMPGYCVQGTVGNKILGGAKKVEFENRQVVEVKMAVEYMSFSAHADAKGIMQLIQNCEPKNVMLVHGEAGKMKFLRSKIKDEFNLETYMPANGETCVISTPVKIPVDASVSLLKAEARSYNAQPPDPKRRRLIHGVLVMKDNRIMLQNLTDALKEIGINRHVMRFTSKVKMDDSGPVIRTSERLKTLLEEKLAGWTVTMQENGSIAIESVEVKVEEDEKDPKQKNILISWTNQDEDIGAYILNVLQNMC

>NP_650738.1[Drosophilamelanogaster]

MTQATGDARMPDEESDLLQIKPLGAGQEVGRSCIMLEFKGKKIMLDCGIHPGLSGMDALPYVDLIEADEIDLLFISHFHLDHCGALPWFLMKTSFKGRCFMTHATKAIYRWMLSDYIKISNISTEQMLYTEADLEASMEKIETINFHEERDVMGVRFCAYIAGHVLGAAMFMIEIAGIKILYTGDFSRQEDRHLMAAEVPPMKPDVLITESTYGTHIHEKREDRENRFTSLVQKIVQQGGRCLIPVFALGRAQELLLILDEFWSQNPDLHEIPIYYASSLAKKCMAVYQTYINAMNDRIRRQIAVNNPFVFRHISNLKGIDHFEDIGPCVIMASPGMMQSGLSRELFESWCTDPKNGVIIAGYCVEGTLAKAVLSEPEEITTLSGQKLPLNMSVDYISFSAHTDYQQTSEFIRLLKPTHVVLVHGEQNEMSRLKLALQREYEADASTDIKFYNPRNTHAVDLYFRGEKTAKVMGSLAAKNSEVGSKLSGVLVKRDFKYHLLAPSDLGKYTDMSMSVVTQRQSIPWGSSLSTLELLLDRIGAGCVEVLEAERKLRVFGCIELTVEQKIIVMEWQATHVNDVYADAVLACIMQSELGGTNLKGATKQTKSEDSRFRECLIETLQDTFGDNCVPKMFEGDLLPVTVSGKRAEINLETLAISCAEDDVLRQMLNTTVQKLHQTLVSAL

Figure S2.

>Sbi:Sb01g009630.1|Sb01g009630.1|Sb01g009630|weakly similar to Putative uncharacterized protein

MEEDDEFGDLYTDILIPTQTPASTSALSNSVPVETLPRPPPNPNPTPVAAAAVEEDDDWLLGGSDPIPGVDPTGDWADEDDDGGEPAPPAKHEVAAPAKRPAAADDLDPLMGGGAGDSGPAIPGLSSSAAAGAAGSDEWDSDSEDDIQIVLNETDGRRGLGEDEGDDEDGEDLVIVADGPHITGMEEQDWGEDPAAAGAEGERKDGGEPGKTVAAPGGRIGYSGGGPGFHQQHHSMFKYVRPGAPGASVGGVPGGPGQFRPPGPSGPFSGRGRGDWRPAAGRGMNKSFHSGYGITPWGGSGRGFGGLDFALPPHKTIFDIDVETAFEEKPWKFPGADISDFFNFGLDEEKWKDFCKQLDYDPDLPPELAAVTGHPDISADSRNKTDNSHTDFSAQGRGPANVRTPVMTGRPIQVETNYTDRFPSVDSRPHRMRESDSVIEIVCQGRDSMDDETVDQTEKDSQGGHHNIYWFYQRVQVTNITARDRHANSDKYINLQPGDVETKEVNEQESSRGHATLSKKSQNPQPDNSLLNQVEEDAISDDENHEDSRRGRSKLERWTSHKEIEYSNIDDDSTQTFPTIKADAEADEVGKSEVSAAVGNSDINSNVDTGQTSDKMTEERDRHLDTVERLKRRSERFKLPMPGEKDAPQSKKVDTEVQPPQNESAAVDMEVKPERPARKRRWTGS

>Sbi:Sb01g037370.1|Sb01g037370.1|Sb01g037370|Predicted protein

MLMSIDHPGERHLNVSCFLLGRVVDKEVHDGGSSEGVGSKLDRRDSYFARDQSRSPDYSDMLSGESKENLYFKRSDRHSDFRDFFEDTKLKDEHVKFDFNCYSSRSDREDSESCSRGYTPSADDRKVSSKLLWRGEAPFAGQDKSSKLFVGCNSDRDVKSRHETRKGQRRHNLDDRRNAIFVEKEKPTDSYPSRYDRKYEKRRSSSSSLRTNYHNSVRNQSYEQRYSPLERIALKNDEHYFSNESNYHRRQSLSCDISEGEDVDECFSSANEWQRDRDHIYHSMVKTDMPDADDGQMYRERYSQEKRRAIHDRSMDVEFSHYTDYRICEWQSPVRGRYRDKGRFAKSNDWHFRHANHLELYPGLNNSERDRPATGFSSMSSRSRCINNKKVRNAKMAQNDCHGYHQKNKQHDSSFCIGNSRSALRTDTFAETGRFVLPIKRKLHSDLGSVDQKTLADLPLLKGRRLMHGQSIVSDRRIYALKLHKSTEKINTEAICSSLDMRNSNTVSNICVGRRHELDNADNIHLNDRKIKFERQGNELRRVIENNQKGHHPVDRDLHASGHKHVHQKPWKQNMGHHHSGNQDLDKSADQKWLNEDVEVEEGELIEEDHHDIISKSKLKPRNVVLKSVIETGSAEQLQVNNTMSKDAACNNRATRECDDKHILEVMEKMQKRRERFKEAIAPQKEDGDKKDLSSLACSTDYIQNQRPARKRRWGGNSWMLEMP

>Smo:441074|441074|441074|synonym:estExt_fgenesh2_pg.C_140033

MEDLEPSAASLEESIKRVVCIEDRALDRSFVVTEVMYSGVQEAVKDCETSGKASEIRVIDVVSGRTPQAENLEPGSDASKVKVIDSETSLESDEVAPQAENFGPPQAENLGPAATSEGSDEQLKVVDGQEPQPQDDGDPVSDDSDSEDDLQLIVNDEDIFYPHGVEGAEDAEPEEVFAVGGEEHYEEVDWAAEMRFRHSQFKYVRPGSGPATTSPGVPDPQTPGATAIAPIPFAGYGLGMWDFHTFQPAYEFVLPPSKTVFDIDIDQFEEKPWEYEGTDITDYFNFGFTEDTWKQYCEEFARAKAEASKKSKMRGLDNGHSQQGWNYDNARYNDTPEPQRGRAQGQRGMRNHSGRAIQVEEGGNERRSSELRRQRLRDSESGIQIDEGRYENSHGEFRRKQRNDDLPRRKGFHRESQSAEQSMLCSSPLSGRLGGRTPPGPRTNLGGIYSGGIISTSTSGRSTFSSRSSPGGVQGSVENGFRTLSKRLSEEEETPVRRSPPRFERASASAPQRRLGEMVAEKPQRRLSDVGTEKPPRRLSDLMTEKPPRRVSELMTEKPPRRLSELVTEKAERRSSELVTEKAERRPSELVTEKAQRRPSELVAERRPSELAREKPQRRLGEQVEKRPISLVRRVTQLVNETKSPQEKKLVSTMEPARRTIRLNAERRSQLISERTADRVERRSEVTVERRQVSLRAADIEFEQDDEEEQPARDSNERIGQSRQLTRAPSLSKSEDRPKFKQQDSGAKDDLRMKVLEKVSARWKLHLRMDDAHARRKRENLSKPDQKDNEEGAGGYHNSTIRDTQTAYASEELEDDNRRERKEERKRRHKDRKERRRLKSEDEDARDCDDYYQEVDERWQMEDADFTGHECRDDRKRKRRRYDDVLGVEEDERYSDSRYRRRDYNDDGADGWEMEEGECEEDVYVEDVECEDDRWEDDGYTYKRKRRRYNDELFEMDRDYETDNPGDERDYSYDEFPSGQADDLVYKRQENPRHRETDRWTGTLDDSCDEYDDYDN

>Aly:477974|477974|477974|synonym:fgenesh2_kg.3__675__AT3G66652.1

MDSTDDDFGDLYVDDAKVQASDASADDVGFVKSCKESECATNSSDFEGTVKPDLEGEMKKFDVVAKDSSPCDDDCAVNLTEADEELEFSDSDSDDDLNIVLKDDDSKPLPAACVFNTNYGGSKACSFQRRWTRNASANNACIDPSLGMSQYGYSFSNPWSRTPFDVNFDVFEKKPWRNPGMDTTDFFNFGLNEQSWKDYCKPLGRAIEVGGGTLERIPSADLRRPRDSDPGVVIQILVTNDVEELSIMTPERARCITSNEASRSDDSHSNDRNDLNSVDDSPKDEAFVGCQEKNAASFSGEKSPPTENCCSREVTPSDKEMLEEEKEEGFCNSDETDPSSVERESSLGDRIRLSPTSSCSAGKNEESDDYETESLKDSATDDQREVSTPPQQARLAEHEAISIKRGGNSGTMHSRPRRSHEDSSKRHCGRAGYAGYVKDASPTPDPGCGKKVRSRHGSLYRDSNKNWQKGPRVTLERDETEGKGVHYYRENCHGRLYSSVDHAKHRKHRFGWRNIKESSLGRGFDHSNSYKCGAHPKEYTSRSSFDLNQRNSRSSFKEEDDRYGWHHCERKYGHERSPVRAYENYKERNGCNWLREPYYEDCIPITGMDYRYRSDYTSAHAIHNLNQSPENDIYCRRRGGYDSNLHRHRYEDVVHRAESRIPFERAYREVRSFAEVEMREYQGYKRHEEFSEIEKRRHYTHDWNLDRFVSEKDDCKYRTQDDWSSPSLSLRDSWYTKEAKGDFWRDDARDFRTAEAYDNQNNQFHKAATRDGRTQNLGRSDNVSIKDRLKDDDDWVCPDRGRYNTADDIQCSMREVTYSGHPSYTDEILRHIRENDERHHKSKKLRGDGHSFIKRQDPVDLAGRQGKRSNQSNKRFSNGEQQDLQKPRKLVGKSEEKAMQTRDINDKEEGEIIEEATNVKGVEIDNERIQESLKKMEKRRERFKGTKMARTVEATFKSETERRAKTDVTNQQRPVRKRRWCAS

>Aly:495910|495910|495910|synonym:fgenesh2_kg.8__1726__AT5G58040.1

MEEDDEFGDLYSDVLQPFQPPVVLPPPPPLPLRSIDLNLRSQDQDVTEPNSASISRVSDNDALKLSTTLSQDATRQAIVDGGGDDKDMSFDIEEPDADSTPTIPGLFVTASEAGALPGLATDRGVSQVTTRIEQQVGGGGDGQGEGDDWDSDSEDDLQIVLNDSSRNVMIGGADRRSRMGDNEDDDDEDDEDPLVIVADTDPNQPMEEQLWGEDGLQGIEGDGKDGGEAGKGSGPGGATGPPKAGYSSHGYHPFHSQFKYVRPGAAPIPGGAASVGGTSSGQVRPPANLGPLAGRGRGDWRPLGMRNASAAQKGFHQPWGSNTAGRGLDFTLPSHKTIFEVDIESFEEKPWRYPGVDMTDYFNFGLNEESWKDYCKQLDQHRIETTMQSRIRVYESGRTDQGYDPDLPPELAAATGAQGVPVDSSNLVKPDSVQGDSAKVPANVRPSLPPGRPIPVEAGSGERLPSIDTRAPRMRDLDAIIEIVCQDSHEDEPSGENGTNQADSSLPEENVPVETSYVNSRRPDTESAEHSPAQDEPLKNLLKKQDDEISRSTDSGQSFRSSSPVGDRGTRSSSVDRENVGGEAGKDVEMGEEHKMSSKFPQSAVQEDDGGESKTERSSESSKARSGSHKDYQQLKDGAEEEVIQDKHYTRPASNRKQHDNNAPHQSRKNQDRGKEVERTRAASKGGRENSNPHMELDSSYIYSIANREDFDKRKERDVDGGVWRRKEDDPYSRRGGDDGSRKRDREDDPGFRQRGKMRENEIRSKDDHVPSRKHMDDAGMRNNYEADDHISKRRKDEEYLRRSRPEKNEISYGQRESISRLKRERDDRLEHQKRDVQHKIRDDFDDHSSLRHRDDIYMQRDGNERLRERDDLDKLKLTHEDGISARGRERQVAVRAHRGSEDRLSRMKDEYKASDKDHLTKDTLRHAKQTKRRDYPGEESSSHHRGHEDFSARTDDIVNNEKKPRQERTGAKIDKFIDTLDGQRLQDRKHKDSRRKIKEQREGTESLRSKQGEQNGSSVVTGSKGTNEARNCRSENPQQPNPTKRHKENASSGDELHDSKRGRTKLERWASHKERDDTVSAKSSSISSKLEEKDKNTNGRLSEPVHGSISKSRDVTEEKSGHNLAETKDGSEKGPGDWHLDTVEKLKKRSERFKLPMPTEKDTTGVKKMESETLPSAKIEGPVDSEGEYVWDELSCVRIGREYA

>Gma:Glyma09g13570.1|Glyma09g13570.1|Glyma09g13570

MEDDDEFGDLYTDVLRPFASSSPSSSSAQQLHQLSPAPPSLDLNLNPNPDAAQIPCDAPHTYSPAPTNPLPEPDPREPPPESPKIPDAEPLPDSNLVAAVVAGVDPMDREVKFDIEEDDDDGGCGGDVVGETVIPGLSGEAAAAVPPEGEGDDWDSDSEDDLKIVLNENNHMAMERGGVADGDEEEEDGDEELVIVAGGDLNQGVEEPEWGENAALAAGDGDRKDAAGELAKVGGAAVPPKIGYSNHGYHPFHSPFKYVRPGAALMPGAAASAPGGPPGQIRPLANMAGRGRGEWRPPGIKGGAAMQKGFHAGPGLPGWGSSAAGRGFGGGLEFTLPSHKTIFDVEIENFEEKPWKYPNVDISDFFNFGLNEESWKDYCKQLEQLRLESTMQSKIRVYESGRTEQEYDPDLPPELAAATGIHDVPGEHTNSLKSDVGQSDVMKGSGTGRVRPPLPTGRAIQVEGGYGDRLPSIDTRPPRIRDSDAIIEIVLQDTEDDESSAGIAQDPPESGDPHREDFREDHVAGDEIPRLEPKYFDGFPQDYNGRKKEIAGRRMPFINSCAANMPNGDEKLFFPQEEPIEYSGSRGQNRRNYGGNFSSSHDERQMQRRVRGQSPPIIPIQELATDNSQKEESAESMEGRHRSSPAVKDVGESSVEYKDIELEDTETADGSSRLEKEETVDRVDTLEDGVAKRQKDSKAAKSSDNSKARSASSRDNQKRQEGFEEEVVQDPQSAHLGSIRQHPDEIEPGFYKREHDAKQEPERNRMMLKGRERSYPYKDRHPSSAPQLHANTDGFDGQKERDNSEMDWARRDDDLYNRRVRNDEPRKRDRAKVRENERNDKEDSLHSRKQLDNGSYRVLYEKDVGYEAVEDYRGKRRKDEEYLRREHIDKEEVLHGYRENASRRRRERDEVLDPRKRDDLQRARDNPDDQYATRQKDDAWVPRERGDRQRDREEWHRMKQSHEEHLPKREREEGRSSVRSGRGAEHKLSEKEYQSREAMRQNDQLKRRDRIQDESPHHKGRDDASARGNQYTTEERRSRQERSSSRSDRVANFSDNQKVKHREGSRKSKERDVSDLNSLGLSKRSQENQIGPTNEKGLKGSGDEERAEHEIPGHRLSRKQREDMSSDDEQQDSRRGRSKLERWTSHKERDFSVNKSSSSLKYKDIDKDNNDGSSEAGKPADEPAKTVDVDNQHLLLAEARDSADMENRDADTKELGDRHLDTVERLKKRSERFKLPMPSEKETLVIKKLESEPLPSAKSENPVVDSEVKQERPARKRRWVTN

>Gma:Glyma15g33980.1|Glyma15g33980.1|Glyma15g33980

MEDDDEFGDLYTDVLRPFASSPSLSSAPQPHQPSPAPPSLDLSPNPDDAQIPCDAPHANSPAPTNPLPEPDPREAPTEPPKIPDAKPTTDSNLAAAAVAVDPMDREVKFDIEEDEEDGGEPVIPGLTGELAAPTEGEGDDWDSDSEDDLKIVLNENNHMAMERGGMADGDEEEEDGDEELVIVAGGDPNQGAEEPEWGENATLAAGDGERKDAAGELAKAGGAAVPPKIGYSNQGYHPFHSPFKYVRPGAALMPGAAASAPGGPPGQIRPLANMAGRGRGDWRPPGIKGGAAMQKGFHAGPGLPGWGNGAAGRGFGGGLEFTLPSHKTIFDVDIENFEEKPWQYPNIDTSDFFNFGLNEESWKDYCKQLEQLRLESTMQSKIRVYESGRTEQEYDPDLPPELAAATGIHDSPVENTNSLKSDVGQSDVMKGSGTGRVRPPLPTGRAIQVEGGYGDRLPSIDTRPPRIRDSDAIIEIVLQDTEDDQSSAGVAQDPPEGGEPHREDFREDHKEIAGRRMSFINSSAANMPNGDEKLFFPQEEPIEYSGSKGQNRRSYGGNCSSSHDERQMQRRVGGQSPSITPIQELATDNSLKEESAESMEGRHRSSPAVKDIRESSVEEKDIELEDTGTADGSSRLEKEETVDKRQKLTSRVEPPLLDEVDDWEDSKAAKSSDNSKARSASSRDNQKRREGFEEEVVQDPRSAQLRDSRQRERDEGLRIRYEAVEDYRGKKRKDEEYLRREHIDKEEVLHGYREIASSRRRRERDEVLDPRKRDDLQRARDNPDDQYATRQKDEAWVLKERGDRQRDREEWCRMKQSHEEHLPKREREGRSSVRSGRGAEHKLSEKEYQSREAMRHNDQLKRRDRIQDESPHHKGRDDASARGNQYTTEERRSRLERSSSRSDRVANVSDNQKVKHREGSRKSKERDVSDLNSLGLSKRSQENQSGPTNEKGLKGSGDEERAEHEISGHRLSRKQREDMSSDDEQQDSRRGRSKLERWTSHKERDFNVNKSSSSLKFKDIDKDNNDASSEAGKPAYEPAKTVDADNQHILSVEARDSADMENRDADTKESGDRHLDTVERLKKRSERFKLPMPSEKEALVIKKLESEPLPSAKSENPVVDSEVKQERPARKRRWVTN

>:GSVIVT01023597001|GSVIVT01023597001|GSVIVG01023597001

MEDDDEFGDLYTDVLRPFSSSSAPQPHQSSSNPASFNPSIDLNTHSDDEDFLYPPPELGTAQSRDSGQNFGGGDVLVEQGLGKGGDFVGGSKNCAADSLELGGSRVLESGDVKLPDGASEDDKSGVDAGRGLEPQNREKTNVVARDDASVQGDDWDSDSEDDLQIVLNDNNHGPMAAERNGVMGSDDEDEDGDPLVIVADGDQTHPPLEEQEWGEDTAVDGERKEGADAAKVNGAIAGPPKIGYSSHGYHPFHSQFKYVRPGAAPIPGAAAVVPGGTPGQVRPLANIGPVPGRGRGDWRPAGIKNAPPMQKNFHSGFGAPAWGGNMAGRGFGGGLEFTLPSHKTIFDVDIDSFEEKPWRHPGVDISDFFNFGFNEESWKQYCKQLEQLRLEATMQTKIRVYESGRTEQEYDPDLPPELAAAVGIHDVSAENGNLGRADVGPSDLAKASARVRPPIPTGRAIQVEGGCGERLPSVDTRPPRVRDSDAIIEITLQGSLDDDSPTGNGAPEPPDNDLPREDLRVGNEVEDDAAQEDTEYFDSFSTTYSGRNRELVGRSAPFMNSLRDDMPGGDGILPFPPEAPVQYRPGSRGQDPVHPGGNFGTPHEDRRIRGRAHGKSPHMTPIQSTRDNRFLDSQKEESVESMDVKGMTSSPVRVAPPREPSVEKKDALDGGIVLADGTSGMEREELTSNTMTSTDALKDENLIPFGKKQKLSSRVEQPPPQELDGDEDLKATRSSENSKARSESSRDLQKWHDGGEEEVIEDGSSVRMGNSKRHLDEDEQSFRRKDRDGRQEMERSRMVVKGREDTYPHRDWDSIPNHHSHNST

>:GSVIVT01034996001|GSVIVT01034996001|GSVIVG01034996001

MEDVGDDFGDLYADVEIPASSAINGAPNFVRFYENDTHKAEDFASGSGSKELDIGDAGSSRKSENEESNVVDNGSDSEDDFNIVLNDEDGQRFPVRSGVGVLGGSDGEDGDGMEQGFAGGERGNGAKSGYHLQFSQYKYIRSHSTVFPSNAKANGTAKVASFSSMLARGDWEENGSNQHKGSSSVEIASTHTRAAPLVAQGGYGFSLPWYRTILDVNIDTFEQKPWRHPGVDLTDFFNFGFNEETWKNYCNSLEQYRKQMHILNQTPVHHSSKPNQTEEGGLEHEKDGQEPVCKQGSIVSPTSKSTDRLELPKGRAIQVEGSTGERQPSMDVRRPRHRDSGVVIHIAVQDSVDDEIDNIDSTEDESSENGDFKVGDNKDIHCYGSGNGNKPCLEKNVTLDRSSVLKRFSKLSTASNPVSVDSDNVGTGKIPDGDKHCSQNMNAHVPEGISEVLDALNNSKEMVGRNTCNTDPCMMETELSLDEQVSHSPSSSRRGSHSVASQDGGYIDPEKNQNARRKPSSNLLTDRPELIKSEYYLHKNSKNKVGKTKPIDCKDSFRNRSPVQEARKHRDSSTCSVDKMAIRSGNDIASPMSKTVDSLYDRNHSSVGHGRQKERLHDFGSHDDDVSPMSNSEGLHYKHYLSAGRRRRKERLCDLGSYDGDFSPMSDVEGMHSRAHSSVVRQGRKERLDDFGSYDNDIFPVSETEGLSDKGHSFASRRRRKELHDFDSYDRKGFSYYRETELSFNYCSEKFANNHVQTASAENPHWKDHRSFRDEMYPHFRNKYIFEKRITRAGNKMMERDWYHRERNVSIEDIDTLTHRESRRLVLKYSYSDKERDTRRRKKNDKLQFQEGPDNDDDLFQCKNTDDVAQEKITRSVPFMCKERNSLAEKYGRHVPSTGRKVNLYGRRKRYEDGHLDLDSSWSIGVEDEYGRHVDHQSLSSWSYREPHTANGRNDVNDSRLTERHGRDRRQICPQGYRESDWFGNDNDAYNTKDSIIGPDDQVQIGRRRSRRQYEALHWTEKELISSHLDENLYNEEASLSYERTSGHTRIHTKYGSAHVGMLVHNKKSQQQRYKRIREGRSDDFIDRSSNVLGQGNHEQAVLRSRASVDLIVGEGKSSGRRSEARSAVHHDRFENMDWKIDEDQGILKDVNGPQRGKIIQPDLKSESNWNNEKCLDKFLVTEHDEALDIEEGQIIPEEMNEDDSVETKDASESITPSRNVKRRLGNANAANGNKVVAECDNQRILQTLAKMEKRQERFKKPITLKKEPDKIPKPQVDPIVEMAETMQQRPLRKRRWNGS

>Ppa:Pp1s136_136V6.1|Pp1s136_136V6.1|Pp1s136_136V6

MSDDDDFGDLYAEDVGGGGGGGGGGTGTLYEASKEVRVEESWGYGGEGDVNDEDREDEEELLYGTSSASAVAPAVVGGLTAGFEVGVKDVDNEETFLYGELYGSAAAAATTTSKGDGLESASVEDTRSREAGAAAAIENGNSYGAESDASGLLGRMQPQTPGTVGGLRALESFRGVGGIVVAPGLAPSGSVGLREKEEVTAGEMGDDSAAAAGVAGTGEGEDWDSDSDDGLQIVLNDDAPAFGNPEGEAKSEYYEGSDDEDEEDLIIVAEDEPLDGQENWGEEGGPLSEPPLSGPPSGGPPGALERVVSGGDDRGAPGKTGGQGPGLAGPRIGYSGQGYYTSQPHHAQYKYVRPGAGASGAPASSLAGEPQSGGRGAGRGDWGAGRGAEGGRGDWAGRGRGGSNGQRGSQSGPAAWGGSGGRGYSSGVEFNLPPTKTVFDIDLETLEEKPWQRPGVDITDYFNFGFNENSWKSYCQQLAQLRLEATMQSKIRVYESGRSEQEYDPDLPPELMAAQGLQDAPGDFSNHQRQSDHGGQSGSRGRGSGRGRPQMPTGRAIQVEGGGGERRPSADIRRQRTRDSDAVIHIVLQDASEDEPDVAAAAAVDYDKEYIVDEAKGGKQLEPYSNYHHEEEAREFGPGGPAPWMDYYPYEHWEQGNMMDMAGEHMHGPVRPGMGMEGHGPFPMGPGMSMHPPGAGHDFGMFPGPFGMPPYERSGVGRPPMYGGPRLGGGPMSGPGPDGYLPDHFAMQPHPGGFTEPQRLRTPPGLAHPNRVGPLSGEWEEELSNGSGANRSGHEHHGAHVNRDRESLSWDNDVDKRPQRRSRSVLVEKAAEEPSPEQVSGGPKRSKSKEDGGEGEDSELGGKGRPAKRKRVGSVTMRGDNSQLYSSGASSSDESSDSSLDNIKEVAPGRAKAASLYEKVPRQNDASEQHQYRGDSGSRRRMAESKEREELHRSERSQGLRGKDEPVHDRRSREDRDRDRERDRDRDREREREREREREREREREREREKERERREYDEARHYRKDRGEERNDEVRKRGRPEHGYDREREDRSRKREQEEPGHRRRAGELQGQKAWVDEKRNDDRSRHGDREKDARPYDRDLERSRGREELRSHQREENRPREEVSRVDQEERYREKREGNRSREEISRTGQEEERYRDRDKLRRDDRLDERRDDARSKGGDRAEENRERDLKSRRHMEDRPRISEDRRKGEDRRYELHRKSVANSREVGDDARVHIRRRDGSEEKEADVARNGSLAKDRSHHGYDEHKREKERLGSQHDDESRRDKDSYHSSHQQDKEHGSDIVQRGDRDASRSKRSHAGNRRSENEDVPGSHLTKEPKPESVVEDVSSGEDDGQPRRGRSKTERWNSRREREIPTTSVVDGKRHRRQSRAREADLTNEARNVDQLTNEKRQDQGRHENHQQDAEHSSRNEPRERQQTSISAKRYNNVDIEGEEDPANSKTKEIRSGVVSLKESNRRESPLRSTHRHSGSEDHVQLSEEVTAKLERRKERFGKPRLKKPSEEEMAAGRDEPSYAVEVQADPVEVKQERPARKRRWAGSG

>Ppa:Pp1s31_181V6.2|Pp1s31_181V6.2|Pp1s31_181V6|synonym:Phypa_161275

MNDDDDFGDLYAEDVGRGGTGTLYEASKDVGVERRRRFEDGDDEEQLLYGTSSSSAVVPAVAGGLMAGFEVGGKDVDNEETFLYGELYGSAVTTMSSKAEELQSASIEDFRGIETGSAAGLGNGHSGGIEAGANAGLGRVQQENAGSTSFENPRSVGESVVAPGLAPSGSTGLREAEDVAVGDRADESAPAAKVAVAGEGEDWDSDSDDGLQIVLNDDALGYDNPTIEAKSEFYVGSDDEDEEDLIIVAGDEPRDGQENWGEEGGLLSEPPLPGPPSGGPLGALERVTSGGDDRGPPVKPGGQGPNLAGPRIGYSGQGYYNGQPHHAQYKYVRPGSGASGAPASSQAGEPQPGERGAGRVDWIAGRGADGGKGDWAGRGRGGSNGQRGSQSGSASWGGSGGRGYSGGMEFTLPPTKTVFDIDLDELEEKPWQRPGADVTDYFNFGFTESSWKNYCLQLSQVRLEATMQSKIRVYESGRTEQEYDPDLPPELMAAQGLQDASGDNGNHQRQSDHGGHSACRGRGAGRGRPVMPTGRAIQVEGGGGERRPSADIRRQRTRDSDAVIQIVLQDASEDEPDPATGVDFDTEYTEDEAKGGRHMEQYSNYHHEEDVRDLGHGGPRPWMDYYPYNEPWDQGNMMDMSGEHMHGPMRHGVEGHGPIGPGMPMHPPGAGADFGMFPGPFGMAPYERGGAVRPPMYGGPRMGGGPMSGPGPDGYPSDHFTLQPPPVGFTEPQWRRTPPRLAHSNRAAPPGGEWEEELSNGSGANRSGHEHHSTHGNRDPGSLSREFDADTRLEPRSWSVLVEKGADEPSLGQVSGGYKRTKSKDGAEGEDSELGDKLRPVKRNKVASSTKRGGTSHLNSSGASSSDEISDSSLGNSNEPAPSRVKAVNVHEKVPTNNDASENHQYRDDSGTRRRVAENNEREGLHSSERSQGHRGKDEPAYDRRVREDRDRDRDRERERERREHDVVRHFRKDRGDERNDEVRKRGRPDYDGEREERGWRREQEEFGHRQRSADLRGQKAWVGKKKNEDRSRHGDKEKDLRPYDRDRERSRGREEARSHQRERRRPREEISKIDQEEERHRDRDRDRYRREDRPDRRDDACSRAVDRSEENRDRELRNRRHIEDRRKGEDRRNDLHRKSVVNGREAGDDARMQMRRRDGSEEREADVMRNDSLVKERSHHGCGELKRDKDNVRSGSHHDSESRRDKDGYHLSYQQDKEHGSEIVQYGDRGSTVSKRSQTISRRLENEDVSAHFIKESERESAVDEASSGEDDGQGRQDRSKIERWNSRKEREIPSTLAGDEKRHRRQSHAREADATNDARDVDQHTSENLQDQGRHQNPHRDVENNSRSEHRNRQQRSSTAKRYNNADAEGEEPANSKVKEIRSGLVSMKGNKYRDLSLQNPNRLSDAEDHVQQSEEVTAKLERRKERFGKPRPKKPSEEIPAGRGGSFYVSEVQANTVEVKQERPARKRRWAASGLE

>Ptr:POPTR_0006s20150.1|POPTR_0006s20150.1|POPTR_0006s20150|synonym:eugene3.00061262

MEDDDEFGDLYTDVLRPFSSSSSSTPQPTQPLSAPSYLHRPIDINDAVKDDDDEILHGNPPDPTNQNSIQITSFSAPRIRVLGDAESPIKASIGDDTEVSFDIEEVNTGILEDSGPIIPGLTEDDSRKMEASAEISGGGGDWQDEEESDSEDDLQIVLNDNTHPGGTMGIDREIGDDDDDDEDGDPLVIVADGDGPNQAIEEQDWGGGEDGVAAAGGGAEGERKEGGEAVGKGNAVVGPKIGGNAVVGTAEKYVRPGAAPMPAATSVGPGGTPGQVRPPMNMGAMAGRGRGDWRPVGIKGAPQKNFHPGFGGSAWGAGRGFGSGMEFTLPSHKTIFDFDIDGFEEKPWKYPGVDISDYFNFGLNEESWKDYCKQLEQYRLETTMQSKIRVYESGRAEQEYDPDLPPELAAATGFHATADNSNAGKSDIGQSDLAKGSARMRPQIPTGRAIQVETGYGERIPSIEGRAPRLRDSDAIIEIVCQGSLEDSPPRDGVQDGAHNDPQKDDFKVSDASEDDMEQTENEYAGGFPQAYNGRKGGRRTPYMNSAHNMSEGDVLPIHPKAPAPYHQTGSRGHPPSYPGRESGTPHEERRMQGRSCDSSPHLTPSQNSRDKKFLDDVEEESTESMDDKLSPRISSPITVRDARELSSEEKDDVEPLQAEESSRLGRDEMTENEETANDKDGNVHHSTRKQKVSSHVEQPALQQLDDEEDSKAARSSENSKARSGSSKDYQKWQDGVEEEVVQDRRSTRSGSIRRHLDENEQNFQRKDRDVRREMERNRGVIRGREDSYPHRDLDPSLPHHLHMKHESYDKRKERENPDISWQQRDEDPHSRKHRTEDRKREHGDEMGSRHRGKIRETERSDKDEHLHSRKQLENGSYRIHHDKDGSSRHRERDDNLKSRFEMVDDYHSKRRKDEEYVKREYADKEEILHGHRENTSRRRHERDDQQRIRDNLDGYHSVKHKDEVWLQRERGERQRQREREREELYRVKQSSEENLPKREREEGRASARSGRVVDDKAWAGHAWGKDEYKVSDKEYQLKDTVRISEHQKRRDRMEDESLSHHRGQDDVYARGNQFSNEERRSRQERSSSRVDRTVDTSVSQRVHEKKHKENPRKNKESDGDHGTWGPSKRNQDNLNGHSDETVLKRSREPGSREAEILMQLNSSKRLKKNASSDDEQQDSRRGRSKLERWTSHKERDYNISKASASLKFKETDRNNNGGSLQGSKLSDEPPKKVETVEKQAKIETVEKHCTGEEKDVADAENKDTDTKPSGDRHLDTVEKLKKRSERFKLPMPSEKDAFSVKKMESEAVPSVKPETPADSEIKPERPPRKRRWISN

>Ptr:POPTR_0018s11860.1|POPTR_0018s11860.1|POPTR_0018s11860|synonym:fgenesh4_pg.C_LG_XVIII000956

MEDDDEFGDLYTDVLRPFSSSLSSAPQPLSATSSLHRPIDLNDAIKDDDDDILHVVSHRNPSAPSNQNPIEITAFSAPQVRVLGDAESPIKGSIAEDRDLNFDIEDVNTGILEDSRPIIPGLMEDDSTKIEASAVVSGGGGGGDWEEDEESDSEDDLQIVLNDNSHPGGPMGIDREIGDDDDDDEDGDPLVIVTDGDGPNQAIEEKDWGGGEDGVAAVGGGAEGERKEGGEATGKGNAVVGPKIGYNNHGYHHHPFHSQFKYVRPGAALMPAAPIVGPGGTPGQVRPPMNMSTIAGRGRGDWRPVGIKGGPQKNFHPGFGGPAWGAGRGFGSGLEFMLPSHKMIFDVDIDGFEEKPWKYSGVDVSDYFNFGLNEESWKDYCKQLEQYRLETTMQSKIRVYESGRAEQEFDPDLPPELAAATGFRDAPADNSNAGKSDNAQSDWTKGSARFRAQIPTGRAIQVETGHGERIPSIEGRAPRLRDSDAIIEIICQDSLDDSSTGDGVQDAANDEPQRDDFRGSDVAEDDMAETENEYAGDFPQAYNDRKGGRTPHMNSARNMPEGDGVSPFHPEATAPYPHAGSRGHPPSYPGRDFGTPREERQMQGRSRDRSPHLTPAQSSCDKKFVDNAEEESTESMVGKHSLRVSSPITVQDARELSSEKKDDPEPLQAEGSSRLGRDEMSENEETTNDTPKDGNMHHSTRKQKVSSHVEQPALQQLDDEEDSKAARSSENSKARSGSSKDYQKWKDGVEEEVVQGGRSTRSGSIRRHLDENEQNFRRKDRDVRHEMERSRVIIRGREDSYPRRDLDPSLPHHLHMKHEGYDRRKERENSDISWQQRDEDPHSSKHRTEDRKRELGDEMGSRHRSKIRETERSDKDEHLHPRKQLENGSYRIHHDKDGSSQHRERDDSLKSRFEMVDDYHSKRRKDEEYMKREYADKEEILHGHRENTSRRRRERDDQQWIRDNLDDYHSVRHKDEVWFQRERGERPREREDLYRLKQSNEENLPRREREEGRASARSGRGVDDKAWAGHPRGKDEYKVSDKDYQLKDAVRSSEHQKRRDRMEDESLSHHRVRDDVYARGNQFSSDERRSRQERSSTRIDRTLDTSDNQRVHEKKHKENTRKNKESDGGDHGTLGPSRRNQEDQSGHSDEMILKRSRAPGNGDAGISIQRNSSKRHKEDASSDDEQEDLRRGRSKLERWTSHKERDYNISKSSASLKFKEIHRNSNSNGRSLEGSKLPNELPKKVEVVEKRTKVETVEKHPVSEEKDVAEVVNKDTDMKPSEDRHLDTVEKLKKRSERFKLPMPGEKDALAIKKMENEALPSVKPETPADSEIKPERPPRKRRWISN

>Ptr:POPTR_0010s15530.1|POPTR_0010s15530.1|POPTR_0010s15530

MDDLTGDDFGDLYLDVVIQASSVINSLPNSSNLYTESESHQGNEGSDHNKVNQSEKEENLVSESKELNKEKLGVLEEVGDGSDSEDDLNIVLNDEDCKGFEVGRARNGNGNGGGGFEEEEGEGGGFVGAKNGVECNASGNGVKGIHHLPHLHYKNVRPYGSSFPSNKKGNESAGVAFRSSSSARVLSHGGYGYSLPWYRSILDVNIDAFEEKQWRYPGVDISNFFNFGFNEDSWKQYCMSLAYEFRPEQNSIAIADHISQVDSSSEYADRGGKQLGLRKGRAIQVEDSTGERQPTMDLRRPRVWDSDVVIKSMSVSNKMSPNTDNYEANQISNADGYHHQKENAHSSEGIAEKMETVDEEEEEIDRKECKSDQFLSEPELSLSDYSYFSPSLSYSDSDSEASRDNICHTLKDSPGPLRKPSLGTKDRSSLDCSRQIENLHDFDYRNGEDFSYSRQKDPSCGYNGKRCRDDHAQAVYKKHCHRKYNHSFKDEMIPQNRRDWNEKEFFHERSCRIDDQDTCKDLYSSGRGLFPEDVIPPTYWRSRHLVSKYNNHKEREWKRKSGNIQFQKSTHSMRLLGHENEDDLMQENCGRSVPLANQKRFSLNEKCERRNFIGREGNSSSRRVKYGCDPRMDLNSSWNMETEEDYCDPLHQHFFSKYQRETYESNDGRWQEKLPSRSVMFNSRLTERYRRPKRRICIREGQDRGWDGSFNDTFDAEDGIIYPDDQVHLERRKYSRRSRVLKWKQDESILRHHVGDFYVERKPCFYGVTSTHEKIHAKHRSATGGVVVNGMDFSRHSYKMIREGSNARCVNRNPGRMYRRGHEKMVRRCCDPVDLNVGEVKVNLGKLRSKPCTTVLVCISALLLSKSLFLLMLSHCVFIRHFFSC

>Ath:AT3G66652.1|AT3G66652.1|AT3G66652|fip1 motif-containing protein

MDSTDDDFGDLYVDDAKFQATDAFESECATNSGEDKGFEETVKSDSEGEVKKFDVVAKDSSPCDDDDCAMNLTEADEESEFSDSDDDLNIVLKDDDSKALPASCVFNTNFGGYEASKASSFQRRWTRNASANNACIDPSLGMSQYRYSFPNPWSRTPFDVNLDVLEKKPWRDPGTDTSDFFNFGLNEQSWKDYCKPLGRAIEVRGGTLERIPSADLRRPRDPDPGVVIQIPVTNDVEELPVRTPEKARCITSNEASRSDVSHSYGSKDLNSVYGSPKDEAFVGCQEENAGSFSGEKSLPTENCCSREATPSDKEMLEKEKEESVCNSDETDPSSVERESSLGDRIRLSPTSSSSVGINEESDDYETESLKDSATDDQREVSTPPQEARLAEHEAISIKRGEDSGTMHSRHRRSHEDSSKRHCGRAGYARYVKDASPTPDPGRGKKVGSLQGLYRDSNKNWQNGPPITLERDETEGKGVHYYREKSHGRLNSSVDHDRHREHRFGWRNNKESSLGRGFDHSNSYKCGTHLKEYTSRSSFDLNQRNSRSSFKEEDDRYGWHHRERKYVHERSPIRAYENYKERNGCDWLREPYYEDCIPITDMDYRYRSENSSAHAIHNLKHSPENDLYCRRRGGYDYNLHRDRYEDGVHRVESRIPFELAYREMRSFAEVEMREYQGYKRHEEFSEIEKRHHYIHDWHLDRFVSEEDGYKYRIQDGWSSPSLSLRDSWYTKEAKGDFRRDDTRDFRTPEAYDSQNNHFHKAAPRDGWTQNLGRSHNVSVKDRLQYDADWVGPDRGRYNMADDMQCSMREVSNSEHPSYTDEIFVRDIRVPTHNRMATKQRFGYLQSHIHENDERHHRSKKLRGDGHAFIKRQDHVDLAGRQGKVSNQSKKRFSNGGDTIEQQDVQKPRKLMGKSEEKAMQNRDINDKEEGEIIEEVKGVEIDNERIQESLKKMEKRRERFKGTKLAVEATFKSQTELRAKADVTNQQRPVRKRRWCAS

>Ath:AT5G58040.1|AT5G58040.1|AT5G58040|synonym:ATFIP1[V],ATFIPS5,FIP1[V],FIPS5|homolog of yeast FIP1 [V]

MEEDDEFGDLYSDVLQPFQPPVVLPPPPPLPHRSIDLNLRSQDQDVSEPNSAPISRVSDNDAVKLSTQDATRQAIVDGGGDDKDMSFDIEEPDADSTPTIPGLFVTGALPGLATDRGVSQVTTRIEQQVGGGGDGGYGGQGEGDDWDSDSEDDLQIVLNDSSRNVMIGGADRRSRMGDNEDDDDEDDEDPLVIVADTDPNQPMEEQMWGEDGLQGIEGDGKDGGEAGKGSGPGGATGPPKAGYSSHGYHPFHSQFKYVRPGAAPIPGGAASVGGPSSGQVRPPANLGPMAGRGRGDWRPLGMRNASAAQKGFHQPWGSNTAGRGLDFTLPSHKTIFEVDIDSFEEKPWRYPGVEMTDYFNFGLNEESWKDYCKQLDQHRIQTTMQSRIRVYESGRTDQGYDPDLPPELAAATGAQGVPVDSSNLVKPDSVQGDSAKVPANVRPTLPPGRPIPVETGSGERLPSIDTRAPRMRDLDAIIEIVCQDSHEDEPSGENGTDQADSSLPGENVPVETSYVNNKRPDTESAEHSPAQDEPHKNLLKKQDDEISRSTDSGQSFRSSSPVGDRGTRSSSVDREDVGGEAGKDAEMGEELKMSFTSPQSAVQEDDGGESKTERSSESSKARSGSHRDFQQEEDVIQDKHSSRPANNRKQYDNNAPHQSRKNQDRGKEMERTRAASKGGRENSNPHMELDSTYIYSIASREDFDKRKERDVDGAVWRRKEDDPYSRRGGDEGSRKRDREDDPGFRQRGKMRENEIRSKDDQVPSRKHMDDAGMRNIYEPDDHINKRRKDEEYLRRSRPEKNEISYGQRESMSRVKRERDDRLEHQKRDVQHKIRDDFDDHGSLRQRDDIYMQRDGNERLRERDVLDKLKLPHEDGISARGRERQVAVRGHRGSEDRSSRMKDEYKASDKEHVTKDTLRHAKQTKRRDYPGEESSSHHRGHEDFSARTDNIVNNEKKPRQERTGAKIDKFIDTLDGQRLQDRKHKDSRRKIKEQREGTESLSKQGEQNGSSVVTGSKGTNDARNCRSEIPHQPNTAKRHKENASSGDEIHDSKRGRTKLERWASHKEREDAVSAKSSSISSKLEEKENNTNGRLSEPVHGSIGKSRDVTEEKIGHDLADTKDGSEKGPGDRHLDTVEKLKKRSERFKLPMPTEKDTTGVKKMESETLPSAKIEGPVDSEGEYVWDERSCVRIGREYA

>Bdi:Bradi1g64380.1|Bradi1g64380.1|Bradi1g64380

MANPADAASAAANDDDVDDLYADLDDQVAAALAAAGESGGSNAKDSDPATDAEADANEAVDLGDELACYSSSDEDSEDDLHIVLNEDGCAPPPPSTGRCEGWADESEEGEVRGSLEKGLSINDGGPRKLGGFHPQGLLDKTTVPITGQGDLGYQHAFQKDYYFFLPRNRTVFDINIEAFQQKPWRQHGVDLTDYFNFDLDEEGWRKYCFGMKQFADGARSPAEKLPGMDQESYHNLESIKLMPKSATYCGFEGSNDLSKPKGRAIHVEGGVFERLPSADLWRPRQRDSDVVIQVNMMLSPSNHSTSDDNSTVNDKCMTTKRIVDPGVKCLKDTSLVVDRVVDKEVLNGGSSECTRNKLDVRDSDCTRDHSSSSDMLSEESTEDCYFKRAGRHSNSKALCSGTKLKDVHAKSDFCRHSRKSDLESSTDDSHSYTPSPADDRYHKTIKVARTDEADFRSSGVFMNCQNDSRLLESGHKGKEQKRKDSARVRDDVFEKEEKIVDSYPTRYARSYEKRSRSTFLSNDRHNAVHDQVYEKCDYSPIERSAFRNDMQHLSNISSHRRRSSWHEFSDDEDVVPSFSSVKGWHQRHDNRYRYKSMRKAEISDDIDGRMYRQSYYHETRRVRNDHSEDNDFFHNDYRFDELRGPAVRGKYRNRRSAERNDEHLRHPYHLGLSPQANDYPKNFERDWTTPGLTSLRSRNRCIDNNRIQSTKMMQYHHDGYYQNNEHRNSSFHVDGIQQPALYTAASADTGYCVLPVKRKLHADLGPMNRKDLVGLAFPKGRRFTHDQSVICDRKLYAMEVHSSTKEIGRADIYSFSDMRNSNTISNIHDERSHELVVFQPKDADSIHLNDRKRKFKRHGNEVRREVGRANEECLPAEKDLHSSKHKDVHVKMQKLNGSYHDSVYQDLEKTRYQKSQNGNEEDEIEEGELIEEDHQDSFPKSKLNHPRKATLKSVIEASSAGQLEMINAMSKDVCDKEVSWECDNKHILEVMEKMQKRRERFKEPVVTQNDEDGKNELLAVACSANDIKNLRPARKRRWGGSG

>Bdi:Bradi1g10210.1|Bradi1g10210.1|Bradi1g10210

MEDDDEFGDLYTDIILPTSEPTKPPAAETLAQAAPARNPNSAPAPASTAAADDDDDDDWLLGGSDPVAGVDPTADWVDEDEDVGAPPAKREVDAKPSAAAEESDPLMGGGVGGPGAAIPGLSSTAAAGAAGSEDWDSDSEDDLQIVLNETDGRRRLGEDEGDDEDGEDLVIVADGPHIPGMEEQEWGEDATAAGPDGERKEGGESGKTVPVPGGRIGYSGGGQGFHPQHHSMFKYVRPGAAPGAPLTGAPSAPGQFRPTGPPGPFPGRGRGDWRPGAGRGMNKGFNSGYGMSPWGGSGRGFGGGLDFTLPPHKAIFDVDIDTTFEEKPWKYPGADISDYFNFGIDEEKWKDYCKQLDQLRLESTMQSRIRVYESGRSEQDYDPDLPPELAAATGHHDISADNRNKVDNGHTDFSAQGRVPTSMRPAMMTGRPIQVETGYGERFPSADTRLPRMRESDSVIEIVCQVPSDDPIADSSADQSEKDSQGGNKKANGVEESRPYTSEKNSSSGKSDHTRRLPVSSEGDMLAADAQGRSPPNYKTRGSPSRGARSSKGSSMGANPRQETESLNEVPRQTTSSKRRRDSQREKNPVDDSETKDGSEGSPAVGDETADKLSTDQFADNDDKLALVDSAEVDGDDAISEPRTASETNEGDKLSHSYKKQKLISRVEQPPVLNSSDQDELRTVNSENSRGRSGSSKDNQKRLESGEEVLQDRLSRRVNDVRRHHGGEDRDSRRKDEYTRDVKADIERTHLASRGREDIHHPHVNRDRDIRGKSNDRVREPETWQRREDNIHNRRGKEEDLRLDYNADVGARHRNKARNDRDEDPHSRKWLDDGDWRGSRQRERGDMVLNRRESLDDPHIKRKKDEENMRRIKSENEDTVHAYRGRDDPNKRKRERDDVLDQKRRDDSVRMREKADDRSYAKNKEDNWRQREKEDRQRPKHENTLILQREEGRGTGRGGRIMDDKPVSAGRKKDESRSTLLNKETQERSKQNESGRRGQGAEENNMLNKGRSDVRPRDDNSNNSERNSRQEKINKTHDNNRLSSSSDARHASRDRYRESTRKGRGSEPNEQDLHRSSKRRRDDHDSHRSGKVEMKGVREQENGRDQAASSKMSKNPQRHDSFVKQGEEDAMSDDENTEDSRRGRSKLERWTSHKEIDYSNIDNETTHTFPSIKADAQVPTADVSGKSDIPAVVGNSDLKSSGDNGQASEKTAEERDRHLDTVERLKRRSERFKLPMPGEKEPPQSKKADTEVQTPQNESPAAGIEVKPERPARKRRWTGS

>Osa:LOC_Os03g51520.1|LOC_Os03g51520.1|LOC_Os03g51520|fip1 motif family protein, expressed

MEDDDEFGGLYTDILVHAPPAPSNPAPPAAAAAETLAPAPPAPNPNPTPTPASTKAAAAADEEEDDDWLLGGSDPVVGVDPTGDWADEEEDGGAAQPPPKREAAAAAAKPAPVVAEEADPLMGGVAGDAGAAIPGLSSSAAASAAAAGSEEWDSDSEDDIQIVLNETDGRRRLGEDEGDDEDGEDLVIVADGPIPGMEEQDWGEDAAAAGAEGERKEGGEPGKAAAAPGGRIGYSGGGQGFHPQHHSMFKYVRPGATLGAPLGGAPTATGQFRPPGPPGPFAGRGRGDWRPGAGRGMNKGFGYGMPPWGGSGRGFGGLDFTLPPHKTIFDIDVDTTFEEKPWKYPGADISDFFNFGLDDEKWKDYCKQLDQLRLESTMQSRIRVYESGRSEQDYDPDLPPELAAATGHHDISADSRNKTDNGHTDVNTQGRVPTSMRPPVHRQNLCSRWGLAHPYPLSTPFSPSTPSSTCKRKKRRRSCVTIAVPAPLPALTRKPSGTACFCGAQCATADGRGWNSGEVMTGRPIQVETGYGERLPSIDTRLPRMRESDSVIEIVCQGQSDDPLVDDSTVDQTEKDSQRGDKRTHGAEEGRPYTSEMNSSSALGKEEHKKRLPVSSEGDNATDVNGRSSPSYRTRGSPRGVRSSKGSSAREVESSNEILPRQTTSLKRNNDSQREKNPDEGSESKDGPEGSPAAADEAADKLSADHFDGNDGSLALVDSAEVDGDDVISDPHTVSETTTTDGDNLSHSGKKQKLISRAEQPTGHNSSDQDELRTRNSDNSRGRSGSSKDNQKRLESGEEVLQDRRSRRINDARRHHDGEDRNSRRKDEYLRDIKPDVERSHLASRSRDDTYHPYANRDRDMRGRSYDRVRDTEIWQRREESVHNRRAKEEDVRLEHNAEVGARNRNKMRPPVDRNDRIEDPHARKRLDDGDWRGSRPRERGDVVLNRRENIDDSHMKRKKDEENMRRMKPENEDIVHGQHGYRGRDDPNRRKRERDDGIDQKRRDDNARMREKADDRYHTKHKEDNWRQREREDRHRPKHDNTVTLQRDEGRGSGRGGRILDDKLVTSGRKQDESRSAGLSKEAQERSRQNDPLRRDQGAEENNMQNRGRSDVHPRDENPNNSERNTRQEKPNNTHDGNRLSSNSGARQVSRDRYRESTRKGRSSDINEHDLPKSSKRRREDHESHRGGKVDVKGVSEQENSRDHTVSSKKGQNPQRESFVKQAEEDPMSDDENHEDSRRGRSKLERWTSHKEIDYSSIDNENAPTFSSIKSDVQAPTADELGKSEAAAAGNSELKSGGDNGQTSEKNAEERDRHLDTVERLKRRSERFKLPMPGEKDAPQSKKVDTEVQPPQIESASADLEVKPERPARKRRWTGTGS

>Osa:LOC_Os03g19570.1|LOC_Os03g19570.1|LOC_Os03g19570|fip1 motif family protein, expressed

MADPEDAAAAAAAGNEDDVEDLYADLDDQVAAALAAAGESGGSNPATDGEAEAEAPGAHHTEADANEAVDLGDGTAGYISSDEESEDDLHIVLNEDGAAPPPPPPAGRCEEGSEEGEVSGSCVKGLSTDGGRGKLIPPHKGIERDLEGNKLGELHRKGLFEKTTAPITGQGDRSHQHAFQKEFNFFLPRNRTVFDVDIEAFQEKPWRQHGVDLTDYFNFGLDEESWRKYCFDMEHFRHGTRTLANELSGLQQEFHYNLGLSKSVPKSEIYSVLKEGNGIAKPKGRAIHVEGGMHERLPSADMWPPRQRDSDVIQVNMMFPPSNRSSSDDRSTVNDKCITTKRCGPSNNHPGVDEYLKETSSVVDRVVDKEVHKRGSSECTRSKTVLGDSACAGAQSSTPDNSDMLSEESTEDFHFKRKRGKSNSNAFYVETNRKDEHVLSDFCRHASKSDQESSKGESHRYTPSPADDRYHKATKRQRMDEAGACISSRSLNNCQSDHHLHESGHRAKKELKRQSLAGGKHALFERQENTTDNYSSRYARKHKHKRSSSTFLGTNYRVHNQLCEKQEYLPLGRAALRNDEQCSADYNQRHRRSWREINDDEDIVGCYSARRWQQRHDDLHGSHSMLKAEVCDDIDGHMYRERRYEETRKIRHDRNGDDEFFHYTDYRFGKVLDPEDRRRCRSQSAESCDEHFRRSEHLVFDHFTHPDQLMLSHQANDNHRKSEKGWPGPAASLTFMRSRNRFIDNERIQNGKMKYNHDGYYEKKRQHDSVFDVDDIQQPALYTGSVAETGQCIRPVKRRVHADHSMNRKDRFNSSYQKGRRLMHGWSMISDRDLYVAEMHNSPKDIDVEAMCSPNDMRNSNNIPNIYDKIRHEVVNLQPRDTDNMLLIHRKRKFKRQGIEIRRVVESDSEGCLPADSDLHGSKHKNIHQKVRKPRAFRISRNQASEKSEQQKQQHVSNNQEYEEIEEGELIEQDHQDTASRSKSNHQRKVVLKSVIEASSACQGGVINATSKDADCSNGATGECDNKHILEVMKKMQKRSERFKASIATQKEEDEDRKESLAVTCDVDDIKNQRPARKRLWGCSG

>gi|40254978|[Homosapiens]

MSAGEVERLVSELSGGTGGDEEEEWLYGGPWDVHVHSDLAKDLDENEVERPEEENASANPPSGIEDETAENGVPKPKVTETEDDSDSDSDDDEDDVHVTIGDIKTGAPQYGSYGTAPVNLNIKTGGRVYGTTGTKVKGVDLDAPGSINGVPLLEVDLDSFEDKPWRKPGADLSDYFNYGFNEDTWKAYCEKQKRIRMGLEVIPVTSTTNKITAEDCTMEVTPGAEIQDGRFNLFKVQQGRTGNSEKETALPSTKAEFTSPPSLFKTGLPPSRNSTSSQSQTSTASRKANSSVGKWQDRYGRAESPDLRRLPGAIDVIGQTITISRVEGRRRANENSNIQVLSERSATEVDNNFSKPPPFFPPGAPPTHLPPPPFLPPPPTVSTAPPLIPPPGFPPPPGAPPPSLIPTIESGHSSGYDSRSARAFPYGNVAFPHLPGSAPSWPSLVDTSKQWDYYARREKDRDRERDRDRERDRDRDRERERTRERERERDHSPTPSVFNSDEERYRYREYAERGYERHRASREKEERHRERRHREKEETRHKSSRSNSRRRHESEEGDSHRRHKHKKSKRSKEGKEAGSEPAPEQESTEATPAE

>gi|227330595|[Musmusculus]

MSAGEVERLVELSGGTGGDEEEEWLYGGPWDVHVHSDLAKDLDENEVERPEEENASANPPSGIEEEAAENGVAKPKVTETEDDSDSDSDDDEDDVHVTIGDIKTGAPQYGSYGTAPVNLNIKAGGRVYGNTGTKVKGVDLDAPGSINGVPLLEVDLDSFEDKPWRKPGADLSDYFNYGFNEDTWKAYCEKQKRIRMGLEVIPVTSTTNKITVQQGRTGNSEKEAALPSTKAEFTSPPSLFKTGLPPSRNSTSSQSQTSTASRKASSSVGKWQDRYGRAESPDLRRLPGAIDVIGQTITISRVEGRRRANENSNIQVLSDRSATEVDNNFSKPPPFFPPGAPPTHLPPPPFLPPPPTVSTAPPLIPPPGIPITVPPPGFPPPPGAPPPSLIPTIESGHSSGYDSRSARAFPYGNVAFPHLTSSAPSWPSLVDTTKQWDYYARREKDRDRDRERDRDRERERDRDRERERTRERERERDHSPTPSVFNSDEERYRYREYAERGYERHRASREKEERHRERRHREKEETRHKSSRSNSRRRHESEEGDSHRRHKHKKSKRSKEGKEAGSEPVPEQESTEAAPAE

>gi|24644016|[Drosophilamelanogaster]

MADDTNEDSWLYGTSNPDSTTGELGNGGDSLTAEHESAAAEAQALAELVAGEKPGASAGTNSTEDSPRCPKEEVPEYSEFDDPAQEMEEDEDALPNTDSRSRRERDRERDRGRCADRADARSSPDPEDDEMSDGPAARRERNGSGSDDDEDDDSDDDINVVIGDIKQAPSTYNIKQRPNLLAGGTGAAGDKAKPAGQAGKFSIEDFEGAGTINGVAVHEFSIDSLEEKPWRKPGADITDYFNYGFNEETWRAYCERQKRFRVAESGVGLASLTQNVNQNAPIGILTDGGMGMGPPGMHSIQSMVGMGGESGMQMPPPGMPPPMMQHQSRSGMGLMQRPPRPISTTGGDRGGDRERAVKENAIQVMTAECREYSRGGLGPMAPNFPLPGASEEPFFHEPEPFDYGYEPTQESQWNNDNAGWVPSGIKELTPGHAHMQQPPPGMPPPGMSVPPPQMGGPPPNLRGIMPPNMRMPPNMNMGPPPGMMMGGNAPPQMRMGMAPPQRLAMGDRGAYDDDRERRRREKEKLLKKDQLRKDFLDMLRERHDIERHTRWYDIKKKFEADPRYRALDSSYREEYFEDYLHLLKEEKRKERDLKERERHRDKERSRDKDKDKDKEKDKDKDKDKEKDKEKDKDKDKEKEKDKESSRRERSRSREKSSRRKSKSREKDRSERSSKSSSSNTGGSSSRSEKKKSHRKDKEEDD

>gi|17560448|ref|[Caenorhabditiselegans]

MEDFDDEPAVVHLSDDEEHAVAPVEDSENPDVIQLDDDVIPEEDVPDDEETAENLENLENVLDDSEIIAENSGEADQEVEEEDNPFADDDDSDEDGGGVQVTIRKMEPTEKPAARQGKLDLDTTATINDKPIYDLDLAQMEDRPWRKPGADITDYFNYGFTEETWNLYCERQKKLRIEFAGNQKAANEALFSSIKIANPLANPVMNTTSSVVKVLTDNGGRFKQHVHQSAAPTPLMNDQVIRTVISGNNQSAPSLMDFTRPPPGMSMPPPMGTAPPTSVADAPPGVDMSSDLPPGVESAPGASVAPLLPGGLDLNLGLPPLGFNPNMPPPGMPPMGMMSTSLPPPGFAMPPPQFQQHSRAGFGPGPVGAASAPRSLMGSGAFSTVSDDDEERRSSRRKRSRSRSPHRDRDRDRRDRETRRRGERESDRTSSRRHRSRSASGDRRRKRDDREREKRRERRGDDEDRKKRSRRGDEEEESSSGRKERKEKSSRSRHEDEESSTGVVVKEEIPDDE

>Fip1p[Saccharomyces cerevisiae]

MSSSEDEDDKFLYGSDSELALPSSKRSRDDEADAGASSNPDIVKRQKFDSPVEETPATARDDRSDEDIYSDSSDDDSDSDLEVIISLGPDPTRLDAKLLDSYSTAATSSSKDVISVATDVSNTITKTSDERLITEGEANQGVTATTVKATESDGNVPKAMTGSIDLDKEGIFDSVGITTIDPEVLKEKPWRQPGANLSDYFNYGFNEFTWMEYLHRQEKLQQDYNPRRILMGLLSLQQQGKLNSANDTDSNLGNIIDNNNNVNNANMSNLNSNMGNSMSGTPNPPAPPMHPSFPPLPMFGSFPPFPMPGMMPPMNQQPNQNQNQNSKHTENDNDVSILKSSARNLSLLARRPVDYGRHKDRLQVFGSHKIRDLSCNRETKQSYYYGDEKVVDELVACRSKYYHEDQESLRENTNRHDRKNGDVEDYFFEPGPRFADSEDRERDWYHLGCEYSSDDLSPCSYRESRKFPPKHSSFPDEERYTQGKRMDGKSHFIDRNCIDDFDECEFKELEFLDNYREEQFPHIDRDWRRSVCRGRHYDSPPLVLNNLCSGIMEVEDNCQKYTHCQTSSFKYRRQSYTDSAKNYAYGERVNGNFGGSGRDKHARDNRGSNWLCGYTDTAEDEDFPIYPVKKYQFYRSPSKFLNWTEDEIIYRHHETHATSLFAKVQSDDLPLQRHQLSMPIRDSEKYFKGSSKIMCRSKGGQALLRCRKSVDLIHGEGKVKMGKIWSRTCTFVFMKELFDICLLYFFLVTVIHIMSAVLPFSQILTMFYLFVLNFYLF

>Cre20.g759400.t1.1

MHAGIEDDEFGAIFGASAGLAPQPISLPLPAAGMAAAGKGSKPDETDEEVYAQLFGSLPSTVLPLRYGGLPPVKPITVVLALPPPPPARPAAAGAAGRGAGAEAGGQRQTSPAPGSADAAAAAFLAAAAAAGAGPQDEDDMDMDRAGAGAGQGAAGGDDDDDDFEVTLDELDTAAMAAASVAAAGGAGAAGAGAAAAGGGRAAAGAGAGPPAPGGAVGADGKPGVRLPGPPAGPRPPPGAPAPPGGGGLPVPGPAPPPGGPPPPGPRPPFGGPGGRPFLPRQPQPIYGLNFRYNMEEAAWPTEARLDQAIKLPKQTRVTPEEYRAFLSLGHGEIFELDLDRVLPHEAGWRNPSANPGDYFNYDMNETAWRAYCAAIRAFRDTFDLRAPVPALCPELSGPAGRGAPQGVDFGIDYGLPPEVVAGLRAAGRTRRFQRFDAEGNPLPGPEQLPATAHDWLGEMAGATATGGWDGNEYAFNGRTGQREAPRRRGPPPRVRGAAAAAAAARWATAAAADEDDTIIPLTEVELDEVVVDGPDQQQQQPEEEEEPEQEQEVKQEELQAAQQEQEGVAAAEGQEQAEAEAGNAEAQEPAAAEGQQGPEAEPAGQVQQADQGPSEPAGAAAPDGVEAAMDAANADAAGAVKVKAEPGTEEGAQQPPQQQQQLGAAAVKAEVKREPASGAPRALPAAPVKRAPVWVNADYDPPTAYLPGGDLAPPHYGPLPKRPRSPEPPSRYFRRQDDDAGPSGRDRERERERERENRATRRERERERERERERERERERERRMGLAAVKFEATPPPPPGLLAGMPPIDGVGLGPMSGVMLDGMRTGLDSDAVLVGGTGGDDDDGDFYLDGGGGGDSDEEEQRGGGGGGGGGAGGMGAGPGPGPGMGLGLGMGMGGPPGRGAELDLGPDMPPLMGMGMGLGMGMGLPPVVPPQAQQQQQHQQQQGGRGGGDGDDGGEAAAFGDFSAGGGDYYLDDGSDQQQHQQQQQSSPSGQHHRGHHNHLNNNNNNNSSNNNQHQQHDSLFSDPMGSGLDLLDPLAMMGGMGGLAGLGGHMGMPPPGMSGIMLGGMPGLLPPVAPLPVAGPGGGGGSSAGGAGRAAADRGPSRDRDRERDSRHTSSRRSRSRSRSRRSRSRSRDRDRSGRSIGGGGGSSSGRDRHRSRSRERKDREREKERERDGKDRSGRDKEKDKEKEKDRRRSRSRDRDRDRDRKDRDREKDRDRRDKDRR*

Figure S3.

>Sbi:Sb05g004860.1|Sb05g004860.1

MAAAPAGAQNRCVFVGNIPYDATEEQLVQICEEVGPVVSFRLVIDKETGKPKGYGFCEYKDEETALSARRNLQGYEINGRQLRVDFAENGRNTDRNREKVDDPQIFPVEEPFLPLLEFSVNTRSCIVFVPVNDNSCAFSNHLVSPVSVSCKHVCGRGGPGMASNVDSQKQLAGTSVVGETNLHQPVGLPPAIHAASVMAGVLGGAQTANVQNGLPVQYGLGNDPLTHYLARMSRHQLHEVMAELKFLTTQNKEHSKTLLQGIPQLPKALFQAQIMLGMVTPQMMQMAKSQQPSGALAQSSSHLNEPYPQPDAMIPVVSRPLSLPTNILPNPTELHSFPQHQHASQPPVKMFPHGHQSGIAAQSPILHQSLGGSSSVPTQSLATSVGLISQVQPPFVPQHPGPPVMPTSVQQLPLTHPHLAQVAAAPDILPNEIRVADQASHLTEFNHPSKLRKLEDGTSVPGIVNSSHAVYTAPLQSVGPSGPSGSYSAGAVSLQQPGNEGQLTPDVESALLQQVLQLTPEQLSSLPPEQQQQVIELQKMLSAGK*

>Sbi:Sb09g025360.1|Sb09g025360.1

MASKQAVASEALAAQIYGMSRSEMYDMMSKMKTMIDHDQETVRRMLVNNPDVTRALFRAQVVLGMVKTPKTAQSSDMVPPTAVATAPPSVKTTAPDHVSLPPPPLPANQQSVAQLSAPFPSGLSNVGTTMDIPTISANPPQPIQAKGYPIHQMPSSAPQPSQHPNMALPHAPPQYSNLPSHIPIVHSQPQQPLQSPAIYNQQLQPPLPQMSRPLSMQPFSHQMHPQVPNSFGLTHANAPQHMLQQPMFHPGANPQTNFLPGQPPLPSQPPPQQLYQQASSHYNTQSTTPMGVDRSAPWGRAPEGPTSGSHFPGQLPGLPGQMAQGIGGIQAGQAPLTPEMEKMLVQQVLGMSAEQINMLPPEQRQQVLQLRDMLRQ*

>Sbi:Sb10g021940.1|Sb10g021940.1

MALKQAAAGEALAVQINGISRSEMYDMMSKMKSMIDHDQETVRRMLVDNPEVTRALFRAQVVLGMVKTPKTAQSSDLVPPAAVPTAPSSVKTTAPDHVSLPPPPLSSNQQSVAQLSAPFPSGLSNVGLKMDIPTISANPPQPTQAKGYSIHQMPSSAPQPSQHPNMALPHAPPQYSNLPSHIPIVHSQPQQPLQSPAIYNQQLQRPLPQLSRPPSMQSFAHQMHPQVPNSFGLTHANAPQHMLQQPMFHPGANPQTNFLPGQPPLPSQPPPQQLYQGKCLQAQGAGGGPNNRRTVVTQMGEGAKNSLVSCTHKLFCCNVTSTCVSVLLKINLVVSLPSFSRNVHTH*

>Smo:425016|425016

MPGGNVDATLYVSNLDDRVDERVLYDIMVQAGPLVEVYIPRDKETKRHRGYGFAEYESEESASYALRLFSGLVTLHNRPVNFAFSGGAKKVPQQQQQQQSSLFDPTLDQTPPSSKFERFLSASSSSDRESFCSNNAYFFLETRPLLMAKFQSKLHDEGLVCVRHQQGGVERMANVPHSQPVGALLPLFSLALLHQWLRHPCPSHTNKPAGGASKNYWLYQESYFNIWHCSLSPDNQSLEQVVYNEAKREVILYDSGDGALEAHKSKLDSKELELLSDVCLDRNNNKAYHLYCCGELATYDLESHELSSKLEILEGYVSDASLDLNISVVACKNNAFMVGYLLVECGKYDHIGIWKLDMEGKRWELVSKVPHSDGYVEDEEGNGSYDEDVIAYRITRCCSDGVDKIWIRSCGCIVWMPTHGESFQSIHRLKRLDIVINCISFL*

>Smo:421400|421400

MPGGNVDATLYVSNLDDRVDERVLYDIMVQAGPLVEVYIPRDKETKRHRGYGFAEYESEESASYALRLFSGLVTLHNRPVNFAFSGGAKKVPQQQQQQQQSSLFDPTLDQTPPSSKFERFLSASSSSDRDPKVIMESSSSFIVLNSSDEPGLFVFNTNKEEWSEWPMCHTHNQSELSCPCSRLLCFTNGSGIHVHHTQTNQREELANITGCIREGYFNIWHCSLSPDNQSLEQVVYNEAKREVILYDSGDGALEAHKSKLDSKELELLSDVCLDRNNNKAYLLYCCGELATYDLESHELSFYKLEILESYVSDASLDLNTSVVACKNNAFMVGYLLVECDEYDHVGIWKLDMEGKRWELVSKVPHSDGYVEDEEGNGSYDEDVIAYRITRCCSNGVDKIWIAIDKCEFMWMYSVDADTWRKLPSYSQAKKA*

>Aly:476521|476521

MAGKQIGGDGGLPANLAGMTKSQLYDIMSQMKTLIDQNHQQAREILIRNPLLTKALFQAQIMLGMVQPPQLTPKVEPQAMQQPQQSHQPIPLKPNVQAHMSSIQGGGSVHEPANTMQPQAPIRKHPTPQPMPMPPPPSVSATNTALPQPRFSHPQRQGHLNPTVTSMSHQQSSQVQNAPPPAPHHPTSQPPPFHHLDIPASSTQLQQQPMHSGGGPHLPQQQPRPYHHQYGQAQTGPNTGFQHHGAPPQHLSQPMFHSGNRPPASGVPQFPQGQQHLPSQPTYQGGGQYRGDYNNNQLAGLMAQDRGPSWMAGHSESSNITHLPGLGPVPPPSQVGPGGGQPPRPAPISAEMEKALLQQVMSLTPEQINLLPPEQRNQVLQLQQILRQ*

>Aly:877263|908776

MASSSQRRCVFVGNIPYDATEEQLREICGEVGPVVSFRLVTDRETGKPKGYGFCEYKDEETALSARRNLQSYEINGRQLRVDFAENDKGTDKTRDQGQGGPGLPSTSVMTESQKQIGGPVDTNMHQPVGLNVAITAASVMAGALGGPQVGSQFTQSNLQVPASDPLTLHLAKMSRSQLTEIISSIKLMATQNKEQARQLLVSRPQLLKAVFLAQIMLGIVSPQVLQSPNIVQAPSHMTGSSIQDTQLSGQNLLPPLAQRSQQLSRAPQSQYPVQQSSKQPFSQIPQLVAQPGPSSVNPPPRSQVKGETAPFQRQQGVPASTNIGYSSQTSVPNNAIQPSQVPHPALTNSVMQQGGQTVSLNYGKRINEGPPHQSMNRPSKMMKVDDRRTTSHPGGHASNSMLPNQVQAPQTRISPDVQPTLLQQVMNLTPEQLRLLTPEQQQEVLKLQQALKQDHMMQLS*

>Gma:Glyma05g35080.1

MAGKHVGGEGLAANLAGMSKNQLYDIMSQMKNLIEQNQQQARQILIQNPMLTKALFQAQIMLGMVQAPQVVSKVRPMVSQSNQQSVQLIQKPNIQPAPLLPGHGGAQDQAGVSQTQIPLRKHQNQPSVPISSAVPAMSHQSQPMAAHSLPMPQQHKGHPTPQMAPVSLPQSSQLPNVPLPSLHQTQMATASSQLQQPLQTSGFPPLQPPLPPQIRPTALPTFHPQYPPQVGANMGFQHAGTSHNLPQSMIHPGTKPSASVGSTFPQGQTPLPGQPSSQPPYQVGNMPLGPDFGNQAGNAMQVDRGSSWLPGPSENLAHLSGPPGQMSAAANQLLRPPGLTPEMEKALLQQVMSLTPEQINLLPPEQRNQVLQLQQMLRQ*

>Gma:Glyma08g04620.1

MAGKQVGEGLPANLAGMSKNQLYDIMSQMKNLIEQNQQQARQILIQNPMLTKALFQAQIMLGMVQAPQVVPKVQPMVSQNNQQPVQPTQQPNIQPAPLLPGHGGAQDQAGVSQTQIPLRKHQNQPSVPVSSAVPAARHQSQPMAAHSLPMPQHPKGHPTPQMALVSLPQSSQLPNIPPPSLQSSSQPLHPTQMATASSQLQQPMQTSGFPPLQPPLPPQIRPTALPTFHPQYPPQMGANMGFQHAGASHNLPQSMFHPGTKPSASVGSTFPQGQTPLPGQPSSQPPYQVGNMPLGPDFGNQAGNAMQVDRGSSWMLGPSENLAHLSGPPGPPSVVSGQMGAAAKQPLRPPGLTPEMEKALLQQVMSLTPEQINLLPPEQRNQVLQLQQMLRQ*

>Gma:Glyma10g04650.1

MAGKQVGGEGLSVNLAGMSKNQLYDIMSQMKSLIEQNQQQAKQILIQNPMLTKALFQAQIMLGMVQAPQTVPKVQPMVPQNNLQSVQPTQKPNIQPAPLLPGLGGAQDQAGVSQTQIPLRKHQNQPSVPVSSAVPALSHQSQPMAAQSLTMPQQPKGHLAPQVALASLPQSSQLPNIPSPSLHSLSQPLHPTQMSTASSHLQHPLLTPGFPHMPLPPQIRQPAMPTFHPQYPPQMGANLGFQHAGASHNLSQSMFHPGTKPPASVGSTFPQGLPSQKSSQPPYQVGNVPSGPEFGNQAGNAMQVDRGASLMPGPSDNLAHLSGPPGPPYVVSGQMGAANQPLRPPALTPDMEKALLQQVMSLTPEQINLLPPEQRNQVLQLQQMLRQ*

>Gma:Glyma10g36350.1

MATSQSQHRCVFVGNIPYDATEEQLIEICQEVGPVVSFRLVIDRETGKPKGYGFCEYKDEETALSARRNLQGYEINGRQLRVDFAENDKGNDRNREQGRGGPGMTTNVDHQKQVGIPAVLGEAVQHQPIGLHIAITAAAVMTAALGGAQFGIQSNQNSLQSQSALAHDPLTLHLAKMSRSQLTEIISELKGMATQNKELARQLLLSRPQLPKALFQAQIMLGMVTSQVLQMPNLRLVSDQTSQSLMNEGQLGQPSLVQTLSGLPPPGQGKLQPGLTPYAQEGQVNTIPHNPLAPNQLTAHPKPPVQPRIPLQQHPNNLVLPGTLSGQSNLMLPSARPPGLGSLSVRPLIQPGTSTALNQQMHPSLLQHSVHVGNSTVGHNIQMHCPSMSSANSQLLSKGDKSSKVIEDLNWAKRANTHSKSNIPLGVEKTNMVHDSSESFTRPSKVMKLDEGRSAPLSSGISDMPFTDGSSHILGRSSLPVHAAPKAEGQYSEQQFSQLPPDVESVLLQQVLNLTPEQLSSLPPDQQQQVIQLQQALRRDQIQPS*

>Gma:Glyma20g31220.1

MATSQSQHRCVFVGNIPYDATEEQLIEICQEVGPVVSFRLVIDRETGKPKGYGFCEYKDEETALSARRNLQGYEINGRQLRVDFAENDKGNDRNREQGRGGPGMTTNVDHQKQVGVPAVHGEAVQHQPIGLHIAITAAAVMTAALGGAQFGIQSNQNSLQSQSALAHDPLTLHLAKMSRSQLTEIISELKGMATQNKELARQLLLSRPQLPKALFQAQIMLGMVTSQVLQMPNLRLVSDQTSQSSMNEGQLGQASLVQTLSGLPHGQDKLQPGLTPYAQEGQVNTIPHNPLVPSQLTAHPKPPVQPRIPLQQNPNNLVLPGTLSGQSNLMLPSARSPGLGSLSVRPPIQLATSTALNQQMHASLLQHSVHVGNSTVGHNVQKVRPDANFQPGPSMSTTNSQLLSKGDKSSKVIEDLNWAKRTNTYSKSNIPLGVEKTNMVRDSSESFTRPSKVMKLDEGRSTPLSAGILDMPVTDGSSHILGRSSLPVHAAPKAEGQYSEQQSSQLPPDVESVLLQQVLNLTPEQLSSLPPDQQQQVLQLQQALRRDQIQPS*

>:GSVIVT01008293001

MSDKPIAADALPSNSDEFPQMSRSELYDMMYQMKLLTDENPQQARQILAENPRLARFVFQAQIMLGMMGTPRETPSTQSSVSQHTQQPIALATQQMSGTSGSQNQVPVTKQMENPMQIASAAAQMAPSQIPNLLSHPISSASQPPLARPQMPTVSSQLQQPGQTPILPHMPLQPPLPPHPRLPSMPAFQHQHPSQMGSSMGFQQPGGQLHHSQPVFHSGTQPNASIGPSFTQAQPPLPSQLPSQSFNQIGGSHLRTEFSNPAGSSMQADRGSSAWMPPRHESTMGTQIPGPPPLVPGFGPSNPPPRPPMLNPEMEKALLQQVMSLTPEQINCLPPEQRHQILQLQQMLRQ*

>:GSVIVT01008294001

MSDRPIGADAAPSNSDEFPQMSRAELYDIMYKMKILTDEKPQQARQILTDNPQLTRFLFQAQIMLGMMGSPRETPTTQLPTLQHPQQAQTWISNPSACNETNGNPTCNGHLNIQMAPSQILNTPPLPTSSASQPPLVRPQMPTASNQFQQPLQAPIIPHMPLQPPLPPHPRLPLPPTFQHQHPSQMGSNMAFQQSGSQLHHSQPVFHSGSKPNAGIGPSFTQAQPQIPNQPPSQSFNQMGGSLLRTEFSNAAGSSMQVDRGSSSWMPPRHENTMGTQLPGPPPLVPGQMGSTNPSPRPQLLNPEMEKALLQQVMSLTPEQINRLPPEQRHQILQLQQMLRQ*

>:GSVIVT01013670001

MASSQHRCVFVGNIPYDATEEQLIQICEEVGPVVSFRLVIDRETGKPKGYGFCEYKDEETALSARRNLQGYEINGRQLRVDFAENDKGADRNREQGRGGPGMVANVEPQKQVGGPAILGDAALHQPVGLPLAMAASSVMAGALGGAQAGSKSNQNGFQSQAMLGSDPLTLHLAKMSRNQLNEVISDLKVMATKNKELARQLLLTSPQLPKALFQAQIMLGMVTPQVLQMPNIRQASVPPTQPVLQDGQQGQQLAVQTLSGLPPLAQSKMQLGLMPKGQEGQVSAMPHNSLVHSQFSALPQQPAQPQIQLPPQGHNQALQQATFAGQSGVTALPSVRPQPPAKLPVRPQIQVASSSSLKHQMQPPLLQQPGQFGSAKSGNNSQLVIPNATLRPTLMTRPSFPDSGFQPGSSTFSSIPETTNNNADRSSQVTNNTYSNMPSGLSMKKSMARDTSEPMNHPSKMVKLDDGRRAPFSTVGLNLSTSSASASASGPSQVLGIGSVSANQISKAEEVQHMDKKAPQLQLPPEIESALLQQVLNLTPEQLSSLPPEQQQEVIQLQQMLR*

>Ppa:Pp1s36_103V6.1

MSSTQHRASRCVFVGNIPYDATEEQLVHICEEVGPVVNFRLVLDRETGKPKGYGFCEFRDEETALSARRNLQGYEINGRQLRVDFAENEKGGGGDRNREQRQGGQVQQQAGYSTDTQRQAGGRQAGSNVEIDRSGEPPMGQASAVAAAQFLTGTLGGNQQQQQQYGVSMNGQSMPVANGTPVSVGNDPLTSHLAGMSKQQLYEVMVQMKTMIQQNQQQARQILMANPQLTKAIFQAQIMLGMVRAPPMAMLPTQQQQQQQQQQQQQQQQRAAVPAAPTNLMPQPPLPPQPRPVQTPVISGQVQQPNPLQPISYHPQQMQQPLPQATFQPQGGMVQQSMGLSMSYVQQGVPPPPSQPSPQQQYQMLQQSSRMDGSSGPSVGQAGMVPGSEPVLPTGQVMGLGGIPQMGRNMPMAQAGQVSGMVPGASMNQPSPPPSVPSSIPGQVMPGPFMGAGPPVGSGSQATLNTGVPVSSGSSLIGAPLVPDNQSRPSYGSQAGIPDSSASAMQSHAYGQQMPIQGQVSQQQPLQMTGELEQQKALLQQVLSLTPEQINSLPPEQRQQVLQLQQALRS*

>Ptr:POPTR_0012s05270.1

MTGKPIAGEGLPANLAGMTKNQLYDIMFQMKTLIEQNKQQAKEILIQNPLLTKALFQAQIMLGMVQPPQAIPNIQPAASQQPQLSAQPSRQSNIQAAQTLPGQGALQDQTSVSQSQPPMRKQHQSQPAMSISAPPGPPVNLQSQPLPSHPLHMPQQPKGHVNPQVTPMSVTQPSQLPNLPPASSHSVSQPLPIHQTQMSSVSSQLLLPLQKTGIPHLPLQQPFPSQPRTASVPSFHHQYGQQMGPNMGYQHAGAPHHPSQPMFHSSNKPQSSMGPSFPQGQPPLSIQQPPQSYQAGGSHLGAEYNSQVPTSMQVDRGSSWMSGPPDSSTMTQLAGPPQFNPGQMAQGNQPSRTAPMSSEMEKALLQQVMSLTPEQINLLPPEQRNQVLQLQQMLRQ*

>Ptr:POPTR_0002s06200.1

MGNPDTPSSSCKRWVWNGYQPCPEGHGASLGSGSAFASVLSSLKKALKYRYLGLVIDRETGKPKGYGFCEYKDEETALSARRNLQGYEINGRQLRVDFAENDKNADRNREQGRGGPRLAANNDPQKQAGGPAILEEPAQHQPIGLHIAITAATVMAGTLGGAQTVMQSNQNGLQSQPALASDPLTLHLAKMSRNQLNEIMSELKGMATQNREAAHQLLLGKPQLSKALFQAQIMLGMVTPQVLQLPNIRQSTGQPALSSLQDSQQVQRPAVPNLPGLPPTAQRMQLGLGQFSAGPQPSVQPQKSLVHNQFSATPQPSVQAQTQIPHHVNNHVPHHATLLGQSAPLPAVLPSVRPPVQMANSAPLNQQMQPSLVQHTRQVGNTNARHNPQVVLPNKAMQSSLLSRPPATGSFQQSGLSVSSGLSDAANADRSTLRSNAYLNMQTSTAHDSKEPVNRPSKVLKLDDGRSMSVPMGGSNLFSATGSGPSQAPAVNSVPPNPLPRPEDLQHSGKQAPQLPADIESALLQQVLNLTPEQLSSLPPDQQQQVIQLQQALLRDQMQPS*

>Ath:AT1G71800.1

MASSSSQRRCVFVGNIPYDATEEQLREICGEVGPVVSFRLVTDRETGKPKGYGFCEYKDEETALSARRNLQSYEINGRQLRVDFAENDKGTDKTRDQSQGGPGLPSTTTVTESQKQIGGPVDSNMHQPVGLHLATTAASVIAGALGGPQVGSQFTQSNLQVPASDPLALHLAKMSRSQLTEIISSIKLMATQNKEHARQLLVSRPQLLKAVFLAQVMLGIVSPQVLQSPNIVQAPSHMTGSSIQDAQLSGQNLLPPLAQRSQQLSRAPHSQYPVQQSSKQPFSQIPQLVAQPGPSSVNPPPRSQVKVENAPFQRQQVVPASTNIGYSSQNSVPNNAIQPSQVPHQALPNSVMQQGGQTVSLNFGKRINEGPPHQSMNRPSKMMKVEDRRTTSLPGGHVSNSMLPNQAQAPQTHISPDVQSTLLQQVMNLTPEQLRLLTPEQQQEVLKLQQALKQDHMMQPS*

>Ath:AT1G73840.1

MAGKQIGGDGGLPANLAGMTKSQLYDIMSQMKTLIDQNHQQAREILIRNPLLTKALFQAQIMLGMVQPPQVTPKVEPQAVQQPQQSHQSIPPKPNVQAHMSSFQGGGSVHEPANTMQPQAPIRKHPTPQPMPMPPPPPSVSANSAQSQPRFSHPQRQGHLNPAVTSMSHPQSSQVQNAPPPASHHPTSQQPPFHHLDIPASSTQLQQQPMHSGGGPHVAQQQSRPYHHQYGQAQTGPNTGFQHHGAPTQHLSQPMYHSGNRPPASGGPQFPQGQPHLPSQPTYQGGGQYRGDYNNNQLAGLMAQDRGPSWMAGQSESSNITHLPGLGPVPPPSQVGPGGGPPPRPAPISAEMEKALLQQVMSLTPEQINLLPPEQRNQVLQLQQILRQ*

>Bdi:Bradi2g20030.1

MATANQPAGEALAAHISSMSRPEMHDLMAQMKVMIGHDQERVRRMLVENPDVTRALFRAQIVLGMVKTPKPAHSSDLAQPSVAQITPTSVNAPVQDHVNLSQTQPPARQHNLQPSGPFQSGVPNLPSSLDLPTMPANPPQSAQAKGYPMHQMLPASAPQSSQHPSVTMPPHAPPHYSNVPSHMPTVHSQPQQSLQNPGMFNQQLQPPLPQLPRPPSMQPFTHQMHQQVPSSFGQQMLQQPMFHPGGNQQNSFFTGQQQLPSQPPSLPNQPPPQLYQANSHVSSHYNSQSMQVDRSAPWGRVNQETSSAGSHFPGQFPGLPGQMTQGIGGIQTGRSEAPLTPDMEKMLVQQVLSMSPEQINMLPAEQRQQVLQLRDMLRS*

>Bdi:Bradi4g24180.1

MAAAPAGPQNRCVFVGNIPYDATEEQLVQICEEVGPVVSFRLVVDKETGKPKGYGFCEYKDEETALSARRNLQGYEVNGRQLRVDFAENGRNTDRNREKGRGGPGMASCVDAPKQPTATPVVGDTSLHQPVGLPPAIHAASVMAGVLGAAQTANVQNGLPVQYGLGNDPLTHYLARMSRHQLYEFMSELKTLTTQNKDRAKALLQGIPQLPKALFQAQIMLGMVTPQMMQMAKNQQLSSSLTQSSAHLNESFPQPDAVIPVVPKQEPTAPLHNFPQYQHSSQPPIKIFPHGHQYQLSSQPFSTSSSVPTQPLATSGGLISQVQPPFLPQHPRPQVMPTNVQQLPLTNPHLSQVAAAPEIPMKEIRLPDRTSYQAELAHPSKLRKLEDGTSASGNVNNNAAVYPAPSQVVVPCGPSGSCSSGAVNFQQPENEAPQLTPDDESVLLQQVLQLTPEQLSSLPIEQQQQVIQLQKMLSAGK*

>Osa:LOC_Os05g43780.1

MAANQPAGEALAANISAMSRPEMYDLMSQMKVMIDHDQERVRRMLVDNPDVTRALFRAQVVLGMVKAPKTAQSSDKAQPAAVQATPSSSVKPTVQDHSSFPQPQLPSSQQNIQPSGPFSSGPSNPASSLDLPAMSANPQQSAQAKGYPIHQMPPTSTTQTSQHQSATLPPHVSSQYSNIPSHMPIVHSQPQQPLQNPGMFNQQLQPPLPQLPRPPNMQPFVHQMHPQVPSSFGLSHTNAPQHMLQQSMFHPGGNPQTSFLTGQPPLPNQPPPLPNQPPPQLYQGSSHAASHYNSQSMQMDRSTPWGRGNAEASSAGTHFPGHLPGLPGQMTQGIGGIHSARPEAPLTPEMEKMLVQQVLSMSPDQINMLPPEQRQQVLQLRDMLRQ*

>Osa:LOC_Os11g07490.1

MAAAAPAGAQNRCVFVGNIPYDATEEQLVQICEEVGPVVSFRLVIDKETGKPKGYGFCEYKDEETALSARRNLQGYEINGRQLRVDFAENGRNADRNREKGRGGPGMASSVDTQKQLAGTPVVGDTGLHQPVGLPSAIHAASVMAGILGGSQTANVQNGLPVQYGLGNDPLTHYLARMSKHQLYEIMSELKSLTSQNKDVANKLLQGIPQLSKALFQSQIMLGMVTPQMMQMAKSQQPSSSLAQSSSHISEPFPQPDAMISSVPRPSASLPNPNVLQDPSAQLHNFPQYPHSSQPAGTIFPHGSQSGVGIHPPIVSQLLGASSSVPPLPLATSGSLISQVQPPFMPHHPRPPAMPAGMQQLPLTHPHVPQVPAIPDIAQKEMRFPEQANRSTEFAHHPKLRKLEDGTSTPGIVNNNPAVYPAPSQGMLPGGPSGSYNSAAVSFQQPENEVPQLTPDVESALLQQVLQLTPEQLSSLPVEQQQQVIQLQKMLSAGK*

>Cre01.g024200.t1.2

MDRPPTCTVFIGNIPYDVTEQMLQDMFSQVGAIKSLRMVTDKDTGKPKGYGFCEYHDVGTAQSAVRNLNKYEVNGRMLRVDFAEEHSVDGRGKREKDMRGGGRGGGGPSDSLVPAAPPGTRPIGRDAANAAATQANSLLGNAPYTGPAQDKISTVIAGMTPLQLYEILSQMRSLAQQNSATARSILVSNPQLTKALFQAQVLLGMVKGSAPPSTLPAMPQPQPPQQPPPVAPPAPQPVAPPPMSLPPQGVAPAMQPYPGAPYAPGPGAPPPAPVMGGMPGPAMLIDPTTGMPYTAPPGAVIVTAGQQQGAPQPQPQQMMPPPQAPPPQAVGPGEGPPASVTLPPNLAPQQAQMLQRVLSLTVEQVNALPPAQRQQVMLVRQQLGLPQ

>cstf-2[Homosapiens]

MAGLTVRDPAVDRSLRSVFVGNIPYEATEEQLKDIFSEVGPVVSFRLVYDRETGKPKGYGFCEYQDQETALSAMRNLNGREFSGRALRVDNAASEKNKEELKSLGTGAPVIESPYGETISPEDAPESISKAVASLPPEQMFELMKQMKLCVQNSPQEARNMLLQNPQLAYALLQAQVVMRIVDPEIALKILHRQTNIPTLIAGNPQPVHGAGPGSGSNVSMNQQNPQAPQAQSLGGMHVNGAPPLMQASMQGGVPAPGQMPAAVTGPGPGSLAPGGGMQAQVGMPGSGPVSMERGQVPMQDPRAAMQRGSLPANVPTPRGLLGDAPNDPRGGTLLSVTGEVEPRGYLGPPHQGPPMHHVPGHESRGPPPHELRGGPLPEPRPLMAEPRGPMLDQRGPPLDGRGGRDPRGIDARGMEARAMEARGLDARGLEARAMEARAMEARAMEARAMEARAMEVRGMEARGMDTRGPVPGPRGPIPSGMQGPSPINMGAVVPQGSRQVPVMQGTGMQGASIQGGSQPGGFSPGQNQVTPQDHEKAALIMQVLQLTADQIAMLPPEQRQSILILKEQIQKSTGAP

>cstf-2,tau[Homosapiens]

MSSLAVRDPAMDRSLRSVFVGNIPYEATEEQLKDIFSEVGSVVSFRLVYDRETGKPKGYGFCEYQDQETALSAMRNLNGREFSGRALRVDNAASEKNKEELKSLGPAAPIIDSPYGDPIDPEDAPESITRAVASLPPEQMFELMKQMKLCVQNSHQEARNMLLQNPQLAYALLQAQVVMRIMDPEIALKILHRKIHVTPLIPGKSQSVSVSGPGPGPGPGLCPGPNVLLNQQNPPAPQPQHLARRPVKDIPPLMQTPIQGGIPAPGPIPAAVPGAGPGSLTPGGAMQPQLGMPGVGPVPLERGQVQMSDPRAPIPRGPVTPGGLPPRGLLGDAPNDPRGGTLLSVTGEVEPRGYLGPPHQGPPMHHASGHDTRGPSSHEMRGGPLGDPRLLIGEPRGPMIDQRGLPMDGRGGRDSRAMETRAMETEVLETRVMERRGMETCAMETRGMEARGMDARGLEMRGPVPSSRGPMTGGIQGPGPINIGAGGPPQGPRQVPGISGVGNPGAGMQGTGIQGTGMQGAGIQGGGMQGAGIQGVSIQGGGIQGGGIQGASKQGGSQPSSFSPGQSQVTPQDQEKAALIMQVLQLTADQIAMLPPEQRQSILILKEQIQKSTGAS

>cstf-2[Musmusculus]

MAGLPVRDPAVDRSLRSVFVGNIPYEATEEQLKDIFSEVGPVVSFRLVYDRETGKPKGYGFCEYQDQETALSAMRNLNGREFSGRALRVDNAASEKNKEELKSLGTGAPVIESPYGESISPEDAPESISKAVASLPPEQMFELMKQMKLCVQNSPQEARNMLLQNPQLAYALLQAQVVMRIVDPEIALKILHRQTNIPTLISGNPQPVHVAGPGSGPNVSMNQQNPQAPQAQSLGGMHVNGAPPMMQASMPGGVPAPVQMAAAVGGPGPGSLAPAGVMQAQVGMQGAGPVPMERGQVPMQDPRAAMQRGALPTNVPTPRGLLGDAPNDPRGGTLMTVTGDVEPRAYLGPPPPPHQGPPMHHVPGHEGRGPPPHDMRGGPLAEPRPLMAEPRGPMLDQRGPPLDARGGRDPRGLDARGMEARAMEARGLDARGLEARAMEARAMEARAMEARAMEARAMEARAMEARGMDTRGPVPGPRGPMPSGIQGPNPMNMGAVVPQGSRQVPVMQGAGMQGASMQGGSQPGGFSPGQSQVTPQDHEKAALIMQVLQLTADQIAMLPPEQRQSILILKEQIQKSTGAP

>cstf-2,tau[Musmusculus]

MSSLAVRDPAMDRSLRSVFVGNIPYEATEEQLKDIFSEVGSVVSFRLVYDRETGKPKGYGFCEYQDQETALSAMRNLNGREFSGRALRVDNAASEKNKEELKSLGPAAPIIDSPYGDPIDPEDAPESITRAVASLPPEQMFELMKQMKLCVQNSHQEARNMLLQNPQLAYALLQAQVVMRIMDPEIALKILHRKIHVTPLIPGKSQPVSGPGPGGPGPSGPGGPGPGPAPGLCPGPNVMLNQQNPPAPQPQHLPRRPVKDIPPLMQTSIQGGIPAPGPIPAAVPGPGPGSLTPGGAMQPQVGMPVVGPVPLERGQMQISDPRPPMPRGPMPSGGIPPRGLLGDAPNDPRGGTLLSVTGEVEPRGYMGPPHQGPPMHHGHDNRGPASHDMRGGPLAADPRMLIGEPRGPMIDQRGLPMDGRGGRESRGMETRPMETEVLEPRGMERRMETCAMETRGMDARGLEMRGPGPSSRGPMTGGIQGPGPINMGAGGPQGPRQVPNIAGVGNPGGTMQGAGIQGGGMQGAGMQGGGMQGAGMQGGGMQGAGMQAGMQGASMQGGMQGAGMQGASKQGGGQPSSFSPGQSQVTPQDQEKAALIMQVLQLTADQIAMLPPEQRQSILILKEQIQKSTGAS

>cstf-2[Drosophilamelanogaster]

MADKAQEQSIMDKSMRSVFVGNIPYEATEEKLKEIFSEVGPVLSLKLVFDRESGKPKGFGFCEYKDQETALSAMRNLNGYEIGGRTLRVDNACTEKSRMEMQQLLQGPQVENPYGEPCEPEDAPELITKTVASLPPEQMYELMKQMKLCIVSNPSEARQMLMLNPQLAYALLQAMVVMRIVDPQQALGMLFKANQMPPVLGGNPHQGPGNHTMMGQQQVPQQQVQIPQQQQQAPQPPMPVPGPGFPANVHPNDIDLRMVPGGPMPMDPRMMGRGMDQDLRASLPNPVPPPLMDPRARAQMPPQQQQGVPQAPPAPYPSDPRQRPMDPRLRAGPGPQQQAPPQGIPQAPPPTQQQQAAAQQLQSRLGAHGVLPSDASDQEKAALIMQVLQLSDEQIAQLPSEQRVSIVMLKEQIAKSTQR

>cstf-2[Caenorhabditiselegans]

MMSGGYKSSGVGNDRSQRSVFVGNISYDVSEDTIRSIFSKAGNVLSIKMVHDRETGKPKGYGFIEFPDIQTAEVAIRNLNGYELSGRILRVDSAAGGMNMEEFGSSSNAPAPVEENPYGPECDAGKAPERISQTVASLAPEKMFELMKQLQESLKNNPSELHKFLVEHPQIAYAVLQAAVVMRIVDPQTALGLLHRNKAATLTPFHNTPQGAPPMVQQQQMPMPPKPTFAHPGPSMGPPMGPPMGPPMGHPQAPQYGQNYGQPVAPQQYKPPPQQQPPVQMRPPVQQPQQNHQEEQQNAELLMQVMQLSEHDLQMLPAGDREKIIELRQQLKRNVK

>Rna15p

MNRQSGVNAGVQNNPPSRVVYLGSIPYDQTEEQILDLCSNVGPVINLKMMFDPQTGRSKGYAFIEFRDLESSASAVRNLNGYQLGSRFLKCGYSSNSDISGVSQQQQQQYNNINGNNNNNGNNNNNSNGPDFQNSGNANFLSQKFPELPSGIDVNINMTTPAMMISSELAKKPKEVQLKFLQKFQEWTRAHPEDAVSLLELCPQLSFVTAELLLTNGICKVDDLIPLASRPQEEASATNNNSVNEVVDPAVLNKQKELLKQVLQLNDSQISILPDDERMAIWDLKQKALRGEFGAF

Figure S4.

>Sbi:Sb06g033360

MVSSSSPVVNVYPLANYTFGTKEPKMEKDTSVADRLARMKVNYMKEGMRTSVEAILLVQEHNHPHILLLQIGNTFCKLPGGRLKPGENEIEGLKRKLCSKLAVNSPSFPPNWQIGECVAVWWRPNFETVMYPYCPPHITKPKECKKLFIVHLSEREYFAVPRNLKLLAVPLFELYDNVQRYGPVISTIPQQLSRFQFNMVSS

>Aly:492272

MAMSQVVNTYPLSNYSFGTKEPKLEKDTSVADRLARMKINYMKEGMRTSVEGILLVQEHNHPHILLLQIGNTFCKLPGGRLKPGENEVDGLKRKLTSKLGGNSAALVPDWKVGECVATWWRPNFETMMYPYCPPHITKPKECKRLFIVHLSEKEYFAVPKNLKLLAVPLFELYDNVQRYGPVISTIPQQLSRFHFNMISS

>Gma:Glyma10g38120

MVSSQVVNTYPLSSYTFGTKEPKMEKDTSVADRLARMKLYEGGNEDQCGRNFTGTRAQSSSYTSSPNWKHILQTPGGRLKPGENDIWTLERHVKFGIGDYPHVFCCSFALIHGKLQISECVAIWWRPNFETIMYPYCPPHITKPKECKKLFLVHLSEREYFAVPKNLKLLAVPLFELYDNVQRYGPVISTIPQQLSRFQFKMITN

>Gma:Glyma20g29700

MVSSQVVNTYPLSSYTFGTKEPKMEKDTSVADRLARMKVNYMKEGMRTSVEGILLVQEHNHPHILLLQIGNTFCKLPGGRLKPGENENEGLKRKLTSKLGANSPALVPDWQIGECVAIWWRPNFETIMYPYCPPHITKPKECKKLFLVHLSEREYFAVPKNLKLLAVPLFELYDNVQRYGPVISTIPQQLSRFQFKMITN

>:GSVIVT01026851001

MVTSPVVNTYPLSSYTFGTKEPKMEKDTSVADRLARMKVNYMKEGMRTSVEAILLVQEHNHPHILLLQIGNTFCKLPGGRLKPGENEIEGLKRKLSSKLAANSLALQPDWQIGECVAIWWRPNFETIMYPYCPPHITKPKECKKLFIVHLSEREYFAVPKNLKLLAVPLFELYDNVQRYGPVISTIPQQLSRFQFNMMTV

>:GSVIVT01028985001

MLTSPVVNTYPLSSYTFGTKEPKMEKDTSVADRLARMRVNYMKEGMRTTVDAILLVQEHTHPHILLLQIGNTFCKLPGGRLKPGENEIEGLKRKLSSKLAANSPGLQPDWQIGECVAIWWRPNFETVMYPYCPPHITKPKECKKLFIVHLSEREYFAVPKNLKLLAVPLFELYDNVQRYGPVISTIPLHLSRFQFNMIAA

>Ppa:Pp1s35_259V6

MSAGFGVTRLIASLQLRSCLSVCLSALLLSWGFEAEANMVSGPVVNVYPLSSYTFGSKEAKMEKDTSVADRLARMKQNYMKEGMRTSVEGILLVQEHNHPHVLLLQIGNTFFKLPGGRLKTGENEIEGLKRKLTSKLAPTASSIQPEWQIGECAAMWWRPNFETLLYPYQPPHITKPKECKKLFVVCLSERQYFAVPKNLKLLAVPLFELYDNVQRYGPVISAIPQQLSRFNFNAINN

>Ptr:POPTR_0012s13280

MVGAGSAVVNTYPLSSYTFGTKEPKMEKDTSVADRLARMKVNYMKEGMRTSVEAILLVQEHNHPHILLLQIGNTFCKLPGGRLKPGENENEGLKRKLTSKLGANSPALVPDWQDFSSPYFGHMSFACRLVNVWLWWRPNFETIMYPYCPPHITKPKECKKLFLFHLSEREYFAVLKNLKLLAVPLFELYDNVQRYGPVISTIPPQLSRFQFNMITTT

>Ptr:POPTR_0015s13200

MVGAGSAVVNTYPLSSYTFGTKEPKMEKDTSVADRLARMKVNYMKEGMRTSVEAILLVQEHNHPHILLLQIGNTFCKLPGGRLKPGENEIEGLKRKLTSKLGANSPALVPDWQIGECVATWWRPNFETIMYPYCPPHITKPKECKKLYLVHLSEREYFAVPKNLKLLAVPLFELYDNVQRYGPVISTIPQQLSRFQFNMITT

>Ath:AT4G25550

MAMSQVVNTYPLSNYSFGTKEPKLEKDTSVADRLARMKINYMKEGMRTSVEGILLVQEHNHPHILLLQIGNTFCKLPGGRLKPGENEADGLKRKLTSKLGGNSAALVPDWTVGECVATWWRPNFETMMYPYCPPHITKPKECKRLYIVHLSEKEYFAVPKNLKLLAVPLFELYDNVQRYGPVISTIPQQLSRFHFNMISS

>Cre:Cre10.g462700.t1.1

MATKGQPVYNVYPLTNYTFGNKQPKLEKDTSVQERLARLRSSYEQHGMRRSVEAVLVVQEHNTPHVLLLQLGLNHFKLPGGRLRPGEEEVEGLRRKLTNTLAPANPSLHITWDVGEVLGVFYRPNFDTVFYPYVPPHITRPKESRKLFVVQLPERCVFAVPKNMRLVAVPLFDLHDNLPRYGPVITALPAVLSRLRLNLQFVLPAAPPHGHGGQQQQQQGQHGQQDGHFPP

>Bdi:Bradi5g26806

MVSGSSSSVVNVYPLANYTFGTKEPKMEKDTSVADRLARMKVNYMKEGMRTSVEAILLVQEHNHPHILLLQIGNTFCKLPGGRLKPGENEIDGLKRKLCSKLAVNSPSFPPNWQVGECVAVWWRPNFETVMYPYCPPHITKPKECKKLFIVHLTEREYFAVPRNLKLLAVPLFELYDNVQRYGPVISTIPQQLSRFQFNMVSS

>Osa:LOC_Os04g58640

MVSSSSPVVNVYPLANYTFGTKEPKMEKDTSVADRLARMKVNYMKEGMRTSVEAILLVQEHNHPHILLLQIGNTFCKLPGGRLKPGENEIEGLKRKLCSKLAVNSPSFPPNWQVGECVAVWWRPNFETVMYPYCPPHITKPKECKKLFIVHLSEREYFAVPRNLKLLAVPLFELYDNVQRYGPVISTIPQQLSRFQFNMVSS

>Sbi:Sb07g019840

MGLEMEVMSEAAAPTTAVVPAAAAREPGVDVDIYPLTRYYFGARDAAAAVPRGLETAADRALRLKANFAARGLRTSVHGVLLVELFDHPHLLLLQVRNSSFLLPGGRLRPGEEEVQGLKRKLSSKLSFVDADDDQTIEEEDDWQIGECIGMWWRSEFEAIPFPYMPPSFRAPKECIKLFLVRLPMSRQFIVPRNMKLLAVPLSQVHGNAQVYGPIISGIPNLLSKFSFNVISD

>Aly:491776

MGEEARALDMEETSEENTTRRNHVVHDLMVELYPLSSYYFSSRDALRVKDEIISDRVIRLKSNYAAHGLRTCVEAVLLVELLKHPHVLLLQYRNSIFKLPGGRLRPGESDIEGVKRKLASKLSVNENVVVPGLEVGECIGMWWRPNFETLMYPFLPPNVKHPKECTKLFLVRLPVNQQFVVPKNFKLLAVPLCQLHENEKTYGPIISQIPKLLSKFSFNMMEI

>Gma:Glyma09g07670

MGEEEEEEVSVTALHHPNSGNDDQSLEFDIYPLSSYYFGSKDAVPSKDLTLVDRVLRMKSNYAASGIRTCVEAVLLVELFKHPHLLLLQIRNSIYKLPGGRLRPGESDTDGLKRKLARKLSVNEDGDGSEWEVGECLGMWWRPDFETLMYPFIPPNVKKPKECTKVFLVKLPESRKFIVPKNMRVLAVPLCQVHENHKTYGQIISGVPQLLSKFSFNMIES

>Gma:Glyma15g18870

MRRYGGGMLYAALPLHMQRGLGFTCSGVTYHTISSPLYDFLYMFNFIMPTVNLNYWAFDFVAEVELFKHPHLLLLQIRNSIYKLPGGRLRPGESDTDGLKRKLARKLSIIEDGDGSEWEVGECLEMWWRPDFETLVFPCLPPNVKQTKECIKVFLVKLPESRKFIVPKNMRLLAVPLCQVHENHKTYGKIISGVPQLLSKFSFNMIES

>:GSVIVT01015153001

MRDIWVNMSNPSPLLPVHLGHITHIPSWTATSKVTQHSDIVLRHRPVCYFASPQRFFLSRLFSAFPQALSLIFRMNYEMGSSADGDRSSSGDSSNHVLDIYPLSCYYFGSKDPLLLKEETLADRILRMKSNYSRYGSRTCVVAVILVELFKHPHLLLLQVKNSFFKLPGGRLRPGESEINGLKRKLSRKLSVNEDGDGSDWEVGECLGMWWRPDFETLLYPYLPPNVKNPKECTKLFLVKLPPSRKFIVPKNLKLLAIPLCQLHENDKTYGPIIAGVPQLLSKFSFNIIDS

>Ptr:POPTR_0006s14840

MSDLRLGLTTTPDLSYSGLAIMFGSDMVARPKRGSLWFCVGVSGAELKMGDHSVTVTDNNNHQSTVIEIYPLGSYYFGSKDPIAFRDETIADRVQRMKSNFSARGLRTSVQAVMLVELFKHPHLLLLQVRNAFFKLPGGRLRPGESDIDGLQRKLSRMLSVNEDETDHWEVGDCLGMWWRSDFETLLYPYLPPNLKVPKECTKLYVVKLPASRKFIVPKNLKLLAVPLCQVHENHKTYGPVISGVPQLLSKFSFNINY

>Ptr:POPTR_0018s05430.1

MALFDFIPIVVFSHGLIEVYIFLDFSDNIDGLKRKLSSSVNGDGTDHWEVGDCLGMWWRSDFETMLLPYLPHNVKVPKECMKLYLVRFPESRKFIVPKNLKLLAVPLCQVHENHKLLSKVSFNINS

>Ath:AT4G29820

MGEEARALDMEEISDNTTRRNDVVHDLMVDLYPLSSYYFGSKEALRVKDEIISDRVIRLKSNYAAHGLRTCVEAVLLVELFKHPHVLLLQYRNSIFKLPGGRLRPGESDIEGLKRKLASKLSVNENVGVSGYEVGECIGMWWRPNFETLMYPFLPPNIKHPKECTKLFLVRLPVHQQFVVPKNFKLLAVPLCQLHENEKTYGPIMSQIPKLLSKFSFNMMEI

>Bdi:Bradi3g35340

MGLEMETLLQVGAERETAAAAAERAREQQAVEIYPLSRYYFGARDPASPARAETAADRALRLKANFAAHGLRTCVHGVLLVELLGRPHLLLLQARNSSFLLPGGRLRPGEQDVQGLRRKLSSKLSADGHQQEDYGWQIGECIGMCWRSDFESGPFPYLPPNTRAPKECTKMFLIRLPMSRRFIVPRNLKLLAVPLSQIHDNAQVYGPIISGIPNLLSKFSLNVVRD

>Osa:LOC_Os08g30820

MGLEIMTDEAAAAPSPAAAAARVEIYPLCRYYFGARDVAAGGAGAGLETAADRALRLKANFAAHGLRTSVHGVLLVELFDHPHVLLLQVRNSSFLLPGGRLRPGEQDVQGLKRKLSTKLSVAGHQDDEDGDGDDEWQIGECIGMWWRSEFDAAPFPYLLPNARAPKECIKLFLIKLPVSRQFVVPRNMKLLAVPLSQIHDNAQVYGSIIAGIPNLLSKFSMNIISD

>gi|5901926|[Homosapiens]

MSVVPPNRSQTGWPRGVTQFGNKYIQQTKPLTLERTINLYPLTNYTFGTKEPLYEKDSSVAARFQRMREEFDKIGMRRTVEGVLIVHEHRLPHVLLLQLGTTFFKLPGGELNPGEDEVEGLKRLMTEILGRQDGVLQDWVIDDCIGNWWRPNFEPPQYPYIPAHITKPKEHKKLFLVQLQEKALFAVPKNYKLVAAPLFELYDNAPGYGPIISSLPQLLSRFNFIYN

>gi|13386106|[Musmusculus]

MSVVPPNRSQTGWPRGVNQFGNKYIQQTKPLTLERTINLYPLTNYTFGTKEPLYEKDSSVAARFQRMREEFDKIGMRRTVEGVLIVHEHRLPHVLLLQLGTTFFKLPGGELNPGEDEVEGLKRLMTEILGRQDGVLQDWVIDDCIGNWWRPNFEPPQYPYIPAHITKPKEHKKLFLVQLQEKALFAVPKNYKLVAAPLFELYDNAPGYGPIISSLPQLLSRFNFIYN

>gi|116007798|[Drosophilamelanogaster]

MASSQVSNKSGSGWPRRGSQGQADAASSNNNGTQKYTNQALTINRTINLYPLTNYTFGTKEPLFEKDPSVPSRFQRMREEFDRIGMRRSVEGVLLVHEHGLPHVLLLQLGTTFFKLPGGELNAGEDEVEGLKRLLSETLGRQDGVKQEWIVEDTIGNWWRPNFEPPQYPYIPPHITKPKEHKRLFLVQLHEKALFAVPKNYKLVAAPLFELYDNSQGYGPIISSLPQALCRFNFIYM

>gi|17507315|[Caenorhabditiselegans]

MEDIWPTIERTTISASVPEAPANFDEKPPFNRTINVYPLTNYTFGTKDAQAEKDKSVPERFKRMKDEYEVMGMRRSVEAVLIVHEHSLPHILLLQIGTTFYKLPGGELELGEDEISGVTRLLNETLGRTDGETNEWTIEDEIGNWWRPNFDPPRYPYIPAHVTKPKEHTKLLLVQLPSKSTFCVPKNFKLVAAPLFELYDNAAAYGPLISSLPTTLSRFNFIFNDSN

Figure S5.

>Sbi:Sb06g019410

MDGDDAGGQFLPASASPRTEEAIAAPQNDVRIPLTPSAGEDYDDLYGDVNVGFIPLLPLSPSPAPTSPPKTPSPGRSIQFPPQSPHRLPPPEPLPAPAPAPQREPEPPRSLPPVPRQHMTPTQRQRRPRGGGASYSSPSRCTPLYISELHWWTTDAEVEAALAPAPHGAAAALYGLHFYAEKFNGKSRGICRADFLHPAAAASAAIALHGHAFHGRHCVASLDRPPFLHRLGDDSDSYAEAARSPNPTGGLGNGGRGASNATAIRGNAAPVLGDRPPRAPPQRSVVPRSSPSPQFPGILGCVGGYGGFQSMAQYNAVMGNGMMSSALAHHMNPPFFAASGMGMQGSGVWHGQGMSGGLWGAQQEYNFRSCQMPWQQLRAPRQHQQAHQQFGNGNYGKQGRCLRREGLSNKNEERSIGNVRYPDRRQADRDGDDYYKENDCEKGRHHEHIVDKEREQEKHWNQIDRHGGGNRRYQEYTERADQDRRVRARSRSQSRDDGDDDHPRRRR

>Sbi:Sb07g028800

MDPDGDPAFHRNEAISAVQDVDQYYGDDDDFDDLYNDVNVGDGFLHASHQAPPPPTQQAPPPQQHQHQQQQLPPPPQQPQPQQQLHPPAHTLPPPPPQVPPPQQQVRIPGVAVPGPGIPPGQTSLPPPPQPPAAPAPPPPQHHQIHQGDGINRPGGNFAGGPIVVGNGGPAGGGDGPGGTTLFVGELHWWTTDADLESELSKYGPVKEVRFFDEKASASPRLLPGAIGPGAGDGAGGDGGNWGRGGGGMGNRGPVGNMRNRMGPAGGDRGIMGNGGMVAPPPPMMPPGGMLGQGFDPAGYGAMGRMGGGFGGFPGGPGAMPFPGLMQPFPPVVAPHVNPAFFGRGGMGAGGMWPDPNMGAWGGEEQSSYGDDAASDQQYGEGGSHGKERPPEREWSGAPERRREREKDAPPAQEWPERRHRDERDMDRERNRDYDRDRERDRDRERDRDRDRERDRDRERERERERERHRDDRDRYGDYHRHRDRDSERNEDWDRGRSSGIRSRSREADHSKRRRMTPQ

>Sbi:Sb07g028810

MDPDGDPAFHRNEAISAVQDVDQYYGEDDDFDELYNDVNVGDGFLRNSHPPPQHAPPPQQNNHQQQQQQQQQLPPPPAQLNHHHQPQQQQLPPPPLPQPQQLPPHPLPPHAPPQQQGYAPAVAAPSPSQPPPQPNLPPPPGPAPAPPQHHQIQQRDGFHRPGGNYSGGPVVVANGAGVGGGDGPGGTTLFVGDLHWWTTDADLEAELSKYGPVKEVRFFDEKASGKSKGYCQVDFYDPAAAAACKEGMNGYSFHGRPCVVAFASPNTVRRMGEAQVKTQQALAAQTSSVQPKGGSRGGGGGAMPHAGGNYSGGRGGGAVSGGGGGGNWGRGGGGGRGPVGNMRNNRMGPAVGRGIGNGMVAPPPPMLPQGGMLGQGFDPGFGAMGRMGGGFGNFPVGPGAGPFPGMMQPFPPVVAPHVNPAFFGRGGMGAGGVGMWPDPSMGAWGAEEQSNYGDDAASDHQYGEGGSHGKERPPEREWSGASERRRDREKDMPPEQEWSERRHRNEHDMGRERDRDYGRDRERDRDRERDRDRERDRDRERHRDDRDRYGDYHKHRDRDSERNENWDRGRSSGLRSRSREADHSKRRRMTPQ

>Aly:471472

MTEENDYGGNQKILHQGSGTIPALADEELMGDDDEYDDLYSDVNVGESFFQAHNQPQPPAQVGGTGNASLQAQTSHVAAEPRMGIVSGGTVEGKYRNDGGHNGISGPDTRSDVYPQASSFGAKGLNIDIQSNKIGQQGSTSVVLNNHGFSGNAVNVPELPVHNPYGAPPQGAQQIPVSQMSVNPNVMMNKSPTQPFVVDNGNTMLFVGELHWWTTDAEIESVLSQYGRVKEIKFFDERVSGKSKGYCQVEFYDSAAAASCKEGMNGYIFNGKACVVAFASPETLKQMGANFTGRNQGQNQIQNRRPLNEGMGRGNNNNNMNTQNGDGGRNYGRGGFARGGQGMSNRGGPWGGGMRGRGPNNMASGSGTGPYGPGLAGPAFGGMMHPQGMMGAGGFDPTFMGRGGGFGGYSGIAYPGMPHSYPGVNAMGMVGVAPHVNPAFFGTGMGTMGSAGMNGVHAAAMWSEANGGGGEEGGSEYGGYEDETQEKEEKPSRDKERATTERDWSENSGDRRHKSHREEKDSHREYKQQRDRDSDEFDRGQSSVKSRSRSRMSEDDHRSRSRDADYGKRRRGD

>Aly:495654

RWERSDGRDQMDQFHQNEAISAVADDGFLAEEEDDDYEDLYNDVNVGEGFLQSIQKNDEAGSRNEEKDKVSIEEEDRVEPVLGASEAEVSIPGLVGESVAKEEGGGGSGTDVVVASSGYGAQEVKVSDVSREISGGIGTGTGGGLRVELGQAANRANDLEASRGNNMSQGLLPPPHVLGNNENLMRPVMGNANGGTPPGPGSIMVGNGANIAMPGVVGGGTGGGGGGGGGAILFVGELHWWTTDAELEAELCKYGAVKEVKFFDEKASGKSKGFCQVEFYDPMAASACKDGMNGYAFNGRPCVVDYASPYSVKRMGEAQVNRTQQAQSVIAQAKRGGPADPPSKPVVTNNNNNNNAIGGNFQGGENRGFGRGNWRGNAQGMGGRGPGGPMRNRPGGMGGRGLMGNGGGGFGQGMGTGPPMNMMHQPMMGQGFEQAFGGPMARMGGYGGFPGAPGPPFPGLLSSFPPVGGVGLPGVAPHVNPAFFGRGMPMNGMGMMPNAGVDGGHNMGMWDPNSGGWGGGEDLGSGRAAESSYGEEAASDHQYGEVNHDRGARPNPVKEKERVSEREWSGSSDRRNREDKDAGYERDIPREKDVGHGYDMPERRHRDDRDTGREREREHHHKDRERSREHVRDRERDRERDRHREERERYGGDHRTRHRDEPEHDEEWNRGRSSRGHSKSRLSREDNHRSRSRDTDYGKRRRLTTE

>Gma:Glyma08g44150

MDEAEGGYGESGDPMDQFHRNEAISAVADDGFLGEEEDDDYEDLYNDVNVGEGFLQSLRKNDDSGFRNDDVEEKKPPPPPPAVPDATGVSIPGDGGGGEGVGGGGSGVVESRVSGSVDGFQNQGFRGNDVGSKGGIRVELGNQSGKLTEIEDQGGGGGSGGGGNCECCGGGGGAAGGSGGTILFVGDLHWWTTDAELETELSKYGPVKEVKFFDEKASGKSKGYCQVEFFDPSAATACKEGMNGHVFNGRPCVVAFASPFTVKKMGEAQINRNQQMNPSAVPQGRRGPADAGAKPGGSNISTVGNYQGGEGNRGYGRGGGNWGRGNNPGMGNRGPVNPMRNRGGGMGGRGIMGHGGNGFGQGMGGTPPMLHPQSMMNQGFDPAFGGPMGRMGGYGGFPGAPTPPFSGILPSFPGVGGVGLPGVAPHVNPAFFGRGMPVNGMGMMPGSGMDGPNMGMWSDPNMGGWGGEEPGGGKAGESSYGEEAASDHQYGEVSHDRAGWPMREKDRGSERDWSGTSERRYRDDRDQGYERDAPKEEDMGHDHEWSERRHRDDRETGRERSRDRDRERSRDRDRDRERDHRERDRHREDRDKYADHHRYRDREAEHDDEWERGRSSRMHSKSRLSQEEEHHSRPRDADYGKRRRLTSE

>Gma:Glyma08g44170

MDEGEGGDPMDQFHRNEAISAVADDGFLGEEDDDYEDLYNDVNVGEGFLQSLRKNEDSGFRNDVVEDKKPLQQPDQDPVVGVSIPGVGGGGGSGVVEGRVSGNVDGLQNQGFRGGNSGGGGQPSGKLSEIEEQGGNDGTDGLVRQGQGGGGGAGNVNRVGGNGVGNSVSTVNSVNTGGGGGVVVVGGGAPPGGGGGTILFVGDLHWWTTDAELEAELSKYGSVKEVKFFDEKASGKSKGYCQVEFYEAFAATACKEGMNGHVFNGRPCVVAFASPFTVKKMGEAQINRNQQMNQTAVPQGRRGPADAGAKPGGSNISTGGNYQGGDGNRSYGRGGGGNWGRGNNPGMGNRGPINPMRNRGGGMGGRGIMGNGGNGFGQGMGATPPLLHPQSMMNQGFDPAFGPMGRMGSYGGFPGAPTPPFSGILPSFPGVGGVGLPGVAPHVNPAFFGRGMPVNGMGMMPGSGMDGPNMGMWSDPNMGGWGGEEPGGGKAGESSYGEEAASDHQYGEVSHDRAGWPMREKDRGSERDWSGTSERRYRDDRDQGYDRDAPKEKDMGHDHEWSERRHRDDRETGRERSRDRDRERSRDRDRDRERDYRERDRHKEDRDRYADHHRYRDREAEHDDEWERGRSSKPHSKSQLSQEEEHHSRPRDADYGKRRRLTSE

>Gma:Glyma18g08590

MDEGEGGYGESGDPMDQFHRNEAISAVADEGFLGEEEDDDYEDLYNDVNVGEGSKKRSLRRRRRFRMPPGFRFRVSVAVEEAGLWNLGFRGGLMGFRIKGNQSGNLSEIEDQGGNDGAAMQGIGQQPHGGVVGIVGNEGLVRQGQGGVGGGGGGGNVNRVGGNGVGNSVSAVTSVNTGGVGGAGGGGAAAGSGGTILFVGDLHWWTTDAELETELSRYGPVKEVKFFDEKASGKSKGYCQVEFFDPSAATACKEGMNGHVFNGRPCVVAFASPFTVKKMGEAQINRNQQMNQSAVPQGRRGPADAGAKPGGSNISTGGNYQGGEGNRGYGRGGGNWGRGNNPGMGNRGPVNPMRNRGGGMGGRGIMGHGGNGFGQGMGGTPPMLHPQSMMNQGFDPAFGGPMGRMGGYGGFPGAPAPPFSGILPSFPGVGGVGLPGVAPHVNPAFFGRGMPVNGMGMMPGSVMDGPNMGMWSDPNMGGWGGEEPGGGKAGESSYGEEAASDQQYGEVSHDRAGWPMREKDRGSERDWSGSSERRYRDDRDQGYERDASREKDMGHDHEWSERRHRDDRETGRERSRDRDRDRERSRDRDRDREKDHRERDRHREDRERYADHHRYRDREAEHDDEWERGRSSRTHSKSRLSQEEEHHSRPRDADYVKRRRLTSE

>Gma:Glyma18g08610

MDEGEGGYGESGDPMDQFHRNEAISAVADEGFLGEEEDDDYEDLYNDVNVGEGFLQSLRKNDDSGFRNDEVEEKKPPPPPPVPDAAGVSIPGVGGGGGSGVVESRVSGRVDGFQNQGYRGNEVGAKGGIRIELGNQSGNLSEIEDQGGNDGAAMQGIGQQPHGGVVGIVGNEGLVRQGQGGVGGGGGGGNVNRVGGNGVGNSVSAVTSVNTGGVGGAGGGGGAAGSGGTILFVGDLHWWTTDAELETELSRYGPVKEVKFFDEKASGKSKGYCQVEFFDPSAATACKEGMNGHVFNGRPCVVAFASPFTVKKMGEAQINRNQQMNQSAVPQGRRGPADAGAKPGGSNISTGGNYQGGEGNRGYGRGGGNWGRGNNPGMGNRGPVNPMRNRGGGMGGRGIMGHGGNGFGQGMGGTPPMLHPQSMMNQGFDPAFGGPMGRMGGYGGFPGAPAPPFSGILPSFPGVGGVGLPGVAPHVNPAFFGRGMPVNGMGMMPGSVMDGPNMGMWSDPNMGGWGGEEPGGGKAGESSYGEEAASDQQYGEVSHDRAGWPMREKDRGSERDWSGSSERRYRDDRDQGYERDASREKDMGHDHEWSERRHRDDRETGRERSRDRERSRDRDRDRDREKDLRERDRHREDRDGYADHHRFRDREAEHDDEWERGRSSRTHSKSRLSQEEEHHSRPRDADYGKRRRLTSE

>:GSVIVT01000588001

MPFQGGGAISALADDELMGEDDEYDDLYNDVNVGEGFLQMHRSEAPAPSGVMAGGPFQAHKTDVPPQKLEAGTSQGLIIPGVSIEGKYSNPHFHEKKEGPMAVKGPEMGSTSHLDGPSVSQKGRVLEMTHDTQVRNLGFQGSTPIPQKTGAEPSDVHGKIANESTPVLNSGTGGPRAVPQMLSNQMGMNVNVNRPMVNENQIRPAVDNGATMLFVGELHWWTTDAELESVLSQYGRVKEIKFFDERASGKSKGYCQVEFYDASAAAAFSGKEGILNRGPGGLAGPTFGGPAGGLMHPQGMMGSGFDPTYMGRGGAYGGFSGSAFPGMVPSFPAVNTMGLAGVAPHVNPAFFGRGMAANGMGMMGEEHGRRTRESSYGGDDGASDYGYGEVNHEKVGRSNTASREKERGSERDWSGNSERRHQETLAIRVMLCCSSFFGSSYETPHLCSHFSVSLHQSATVDILYFLISVGPTYGVRLPDGKRSRVGYSLLS

>:GSVIVT01014675001

MSVEEGDGGGFGDGGDPGDQFHRNEAISAVADEGFIGEEDDDYEDLYNDVNVGEGFLQSLRKNEDLGFRNEVEENKIDPAVPSVPPTLPPAAGMSIPGVGGDVGGGAERKFEKDVAVGVSEGGARVSVRLEGYQSVGYRGNEMGVKGPGGASQGGATVGGGGMRVQLGQSSIKTSDFEEQTVNNTVGVQGIVQQQPHGGGVGECWKRGFSKARRRKCEMEIQVGMWLATMALVAVFTLVEFYDPAAATACKEGMNGHLFNGRPCVVAFASPYSVKRMGEAQVNRNQQMAQTNISQGRRGQGDGGGKPGGSNVQQGGNFQGGDNNRGYGRGNWGRGNAQGMGGRGPVGPMRNRSGGMGGRGIMGNGGNGFGQGIAATPPLMGGYGGFPAGPTPPFSGILSSFPPVGGVGLPGVAPHVNPAFFGRGMPMNGMGMMPATSVEGPNMGMWSDPNMAGWAGDEHGGRAGESSYGEEAGSDHQYGEVSHDRGGWQNTMKEKDRGSERDWEKDLVHENDWPERKHREDRDIGRDRDRERDREREHRDRYADHHRYRDRESEHDDEWDRGRSSRTHSKSRLSQEEEQRSRSRDADYGKRRRLTSE

>Ppa:Pp1s42_200V6

MCCGQAGNAPTVANNSGTDFSAKHTGQGPDSGGLMLFVGELQWWTTDAELEAALSEYGRIKNLKFFEEKASGKSKGYCQVEFFDSQAARLCKEKMDGRVFNGRACVVAFASPQTIKQMGAAQVGKNQANPQGQSQTGQGKKAAEGRNAGAAGRGGEGRGGRGQPGMERGRGNQAPGRGRGRGMAPFGPQGPGMGGPPGGMMPPRGMMGQGFEPGFGPPGGGYGMGPGGGGFGPRGPMFGPSFPPMGPGLPGVPAHVNPAFFGRGNANGSGMGPGMEGPPGGWGEVGMPGWDEHQRHMRDGGYAGEDMGGAEFGYGGEMGRVGPTRERERGAGEGEWGPERRRREERSGERVEDWERSDRHRMGERDGYRDQRGDRSEREKDKDWDRADRPSRGPRDKEDERARSREDDHGKRRRMTSDGDRKSER

>Ppa:Pp1s138_94V6

MVAGSAFGREESGAVVFVVVVVVMAFEGNVLEEDLDYENDGNGSRLNEVELLDEEDDFEEDLYGDVNVGVYGAQASGRIAPQNGYGEMDSGNEDEDLLGESKGALGVEARDARLMSVRHAMAASASPPQESVGYQNDYIAQVKEENSHEDTEADPLLSKPKDEFEQGIQLPGRPASVPKGGKNTEILNGSGGAEGGAGRGPRSEGFHPGSWPAGSGPAGAQAGNSLAVANNVGNDFPAKHAGQGSESGGLMLFVGELQWWTTDAELEAALSEYGRIKNLKFFEEKASGKSKGYCQVEFFDSQAARVCKEKMDGRVFNGRACVVAFASPQTIKQMGAAQVGKNQANPQNQSQGGQGKKAAEGRNAGAAGRGGEGRGGRGQPSMERGRGNQAPGRGRGRGMVPFGPQGPGMGIPPGGMMAPRGMMGQGFEPGFGPPGGGFGMGPGGAGFGPRGPMFGPPFPPMGPGLPGVPAHVNPAFFGRGNGNGPGIGPGIEGPPGGWGEVGMPGWEEHQRHMREGGYAGEDMGGAEFGYGSEMGRVGPARERERGAGEGEWGPERRRRDERSGDRGEDWERGERHRTGERDGYREQRGERGDREKDKEWDRAERPASRAPRDKEEDRTRSREEDHGKRRRMTSDRDRKTER

>Ptr:POPTR_0010s06150

MDFEEEEKMQYQGSGAIPALAEEELGEDDEYDDLYNDVNVGENFLQMHGSEAPAPPATAGNGGFQTRNAHESRVETGGSQVLATSGAGVAVEGKYSNAGAHFPEQKQAGIGVEANDVGSIGYGDGSSVAQKGSAGPRGVPQMQVNQMNMNADVNRPVVNENQVRPPIENGPTTLYVGELHWWTTDAELESVASQYGRVKEIKFFDERASGKSKGYCQVDFYEAAAAAACKEGMNEHVFNGRPCVVAFASAQTLKQMGASYMSKTQGQPQPQSQGRGSMNDGMGRGGNANYQSGDGGRNYGRGGWGRGGQGVLNRGPGGGPMRGRGGMGPKNMAGNVAGVGSGANGGGYGQGIAGPAFGGPAGGMMHHQGMMGAGFDPLYMGRGGGYGGFPGHGFPGMLPSFPAVNSMGLAGVAPHVNPAFFARGMAPNGMGMMASSGMEGPNPGKWPDTSMGGWGEEPGRRTRESSYDGDEGASEYGYGEGNHEKGARSSGASREKERVSERDWSGNSDRRHRDEREQDWDRSEREPKYREEKDTYRGHRQRERDSGYEDDRDRGHSSSRARSRSRAAPEEDYRSRSRDVDYGKRRRPPSE

>Ptr:POPTR_0008s18280

MDYEEEEKMQYQGSGAIPALAEEEMGEDDEYDDLYNDVNVGENFLQMHGSEAPAPPATVGNGGFQTRNAHESRIETGGSQALAITGGGPAVEGIYSNAKAHFPEQKQVAVAVEAQDVGPVDGSSVAQKGRVIEMSHDVQVRNMGFQKSTPVPPGIGVDPSDMSRKNAIEPEPLPITGSAGPRGAPQMQVNQMHMSADVNRPVVNENQVRPPIENGSTTLYVGELHWWTTDAELESFASQFGRVKEIKFFDERASGKSKGYCQVDFYEAAAAAACKEGMNGHVFNGRPCVVAFASPQTLKQMGASYMNKTQGQPQTQSQGRGSMNDGAGRGGNANFQSGDGGRNYGRGAWGRGGQGILNRGPGGGPMRGRGAMGPKNMAGNVAGVGSGANGGGYGQGLAGPAFGGPAGGMMPPQGMMGAGFDPLYMGRGGGYGGFAGPGFPGMLPSFPAVNSMGLAGVAPHVNPAFFARGMAPNGMGMMVSSGMDGPNPGMWESSYDGDEGASEYGYGEGNHEKGARSSGASREKERGSERDWSGNSDRRHRDEREQDWDRPEREHRYKEEKDSYRGHRQRERDSGYEDDRDRGHSSSRARSRSRAAPEEDYRSRTRDVDYGKRRRLPSE

>Ath:AT1G13190

MTEENDYGGNQKILHQGSGTIPALADEELMGDDDEYDDLYSDVNVGESFFQAHNQPQPPAQVNTSNASLQAQNSHVAAEPRMGIVSGGTVEGKYRNDGGHNGISGPDTRSDVYPQASSFGAKGLNIDIQSNKIAQQGSTTVVLNNHGFSGNAVNVPEMPVHNSYGAPPQGAQQIPVSQMSVNPNVMMNKSPTQSFVVDNGNTMLFVGELHWWTTDAEIESVLSQYGRVKEIKFFDERVSGKSKGYCQVEFYDSAAAAACKEGMNGFIFNGKACVVAFASPETLKQMGANFTGRNQGQNQIQNRRPLNEGMGRGNNNNNNMNTQNGDGGRNYGRGGFARGGQGMGNRGGAWGGAMRGRGVNNMASGSGAGPYGPGLAGPAFGGMMHPQGMMGAGGFDPTFMGRGAGYGGYSGIAYPGMPHSYPGVNAMGMVGIAPHVNPAFFGTGMGTMGSSGMNGVHAAAMWSEANGGGGEEGGSEYGGYEDETQEKEEKPSRDKERATTERDWSENSGDRRHKSHREEKDSHREYKQQRDRDSDEFDRGQSSLKSRSRSRMSEDDHRSRSRDADYGKRRRGD

>Ath:AT5G55670

MDEGDGRDQMDQFHQNEAISAVADDGFMAEEEDDDYEDLYNDVNVGEGFLQSMKKNDEAGSRNEEKEKVNMEEEDRVEPVLGEAEVSISIPGLVGESVEKEAEAESGGGGSGSGTDVVVASSGYGAQEVKVSDVSQEIPGGIGTGTGGGLRVELGQASNRANDLEAPRGNNISQGLLPPPPVLGNNENLMRPVMGNVNGGIPPGPGSNMVGNGANIAMPGVVGGGTGGGGGGGAFLFVGDLHWWTTDAELEAELCKYGAVKEVKFFDEKASGKSKGYCQVEFYDPVAASACKDALNGYPFNGRPCVVEYASPYSVKRMGEAQVNRTQQAQSVIAQAKRGGPADPPSKPLVANNNNNNNNNAIGGNFQGGENRGFGRGNWGRGNAQGMGGRGPGGPMRNRPNGMGGRGLMGNGGFGQGMGTGPPMNMMHQPMMGQGFEQAFGGPMARMGGYGGFPGAPGPQFPGLLSSFPPVGGVGLPGVAPHVNPAFFGRGMPMNGMGMMPNAGVDGGHNMGMWDPNSGGWGAGEDLGSGRAAESSYGEEAASDHQYGEVNHERGARPNPVKEKERASEREWSGSSDRRNREDKDAGYERDIPREKDVGHGYDMPERRHRDDRDTGREREREHHHKDRERSREHVRDRERERERDRHREERERYGGDHRTRHRDEPEHDEEWNRGRSSRGHNKSRLSREDNHRSKSRDTDYGKRRRLTTE

>Cre:Cre08.g384950.t1.1

MPEQQFLQPQSDLRGAMHINLGGANQGQQQQQQQQQQDGDDHMQQQQPYDGGHLHPPPDGGAGPQDPSQHDDTLFGDQQQQQQQQQYVGPSAASVREAREAGGRVYIANLAWWTTDADVEAAAGEFGRLGGGGVQFLEERGCGRSKGAALVEFEEPEAAAACKEQLAGRPINGRPCVVVFATSSATAALERGDRGAGAAAAAGGSKAWGSHRTGGAPTPPPPAIAVAGSGSGGAVDVGGGMGGGMGSGGGMGSGGGGMGPGMGPGGGMGGGGGGRGFGGRGGRGGRGGRGRGGGGMGGPGGGFGGPMDGGPMGPGPMGMGHMGPGGPMGPMGPGPMGPGGPMVPGGMMAAGLMAAMMGGGPMGPGGPMMGPGGPMMMGGRHPMGGPGHF

>Bdi:Bradi4g33020

MDPDGDGSFHRKEAISAVQDVDQYYGDDDDYDDLYNDVNVGDGFLHASQPPAQPQPPAMPPKQHQPPPQQALPQQQQVMPSPSLPLPPPPPPMGQPEKVHIPGVAAVPAPVPDRPNPSHLPPPPQPPLAAAPPSLPHHQIQAGGGGGGGGGGGGGDGFHRQGGGNYGGGPIVVGNGGGGDGPGATTLFVGDLHWWTTDADLEAELVKYGHVKEVRFFDEKASGKSKGYCQVDFFDPGAAAACKEGMNGHLFNGRPCVVAFASPNTVRRMGEAQMKNNQSMGQQNSGMQKGGRGGGPPGGPAGPQVGGNYGGGRGGGGAAGGGGGGGGGGAGGGNWGRGGGGMGGRGPGGNMRNRMGPVGGRGIMGNGGMVAPPPPMMHPGGMMGQGFDPTGYGAAMGRMGGGFGGFPGGPGAAPFPGLMQPFPPVVAPHVNPAFFGRGGMGAAGVGMWPDPSMGGWGGEEQSSYGDDAASDQHYGEGGSHGKERPPEREWSGAPERRREREKELPPPPDLPERRHRDERDMGRERERERDRDRERERDRDRERDRDRDRHRDDRDRHGDYHRHRERETDRNEDWDRGRSSGRRSRSREVDHSKRRRMSHE

>Bdi:Bradi5g12840

MDAVAGGQLNAVVEFPQAEAIAAVLADCVLTPSTDDDCDDLYGDVNLGFLPLLPLSPSPSSPPKTPSPGISISFSPSPSPPPRRSPTPQPQPEPQPKPPTTPLTAPKPHTPWNQLLLSAPKPPTLQHEPLLLAPKPLSPRHQRALLAPKPPSEWHQPPHHIALGASLPTTTLALYIADLPWWTTDEEVEAALEPHGALQDLHFFADKFSGRSRGFCRTDFLHPSAAASAAAALHGRAFHGYHCVASLSCPPALHRPAPHLTPAFMAANRMAMSGTGTGVWHNQGVAGPGGLWGGQQQWNFGGYEMPWQQPRLQQHHRQQYRNGDYGKMSGTGRERPSGRNEDRDGGNVRGNTERRQFGRGDGERLRQHNRGEGNRHQEHVLEKERERDRNFDENDRRGGEKRRHSEYTEHDDWERRGRARSRSQSRDSDDDDYRRRRR

>Bdi:Bradi3g38990

MAMDPDGDPAFHRSEAISAVQDVDQYYGDDDDFDDLYNDVNVGDGFFNSSSHQPPPLPLNTLPPPQQNQPHQLPPPPQPHQNHLPPPPQPHQNHLPPPPQPQQQHMPVPHSLPTPPRQAPPPQQKVHVPIPNLPPPPQPPVASAPPPPHQQIQQGGDGFHRQGGNFSGGPIVVGNGGAGGGGDGPGGMTLFVGDLHWWTTDADLEAELSKYGLVKEVRFFDEKASGKSKGYCQVDFFDHVAVSACKEGMNGHLFHGRPCVVAYASPSNVRRMGESQMKNQQPVAPQPLPMQPKSGRGNGNASAPQAGGNYGAGRGGPTGTGGGGNWGRGNAGLGNRGPVGGMRNRMGQTSGRGIMGNGGMAPPHPPPMLPQGIMGQGFDPAFGPMGRMGNGFGFPGPGPAFPGMMQPFPPVVAPHLNPAFFGRGGMGAGSVGMWPDPNMGSWGGEEQSSYGDDAASDQQYGEGGSHGKERLPERDWSGPPDRRQEREKDMPPAQPERRHRDERDMGRERDRDYEREREKDRDRERERDREREKERDRHKDDRDRHSDHHRHRDRDPERNGDWNRGRSSGMHSRSRDVDHSKRRRMTPE

>Osa:LOC_Os08g38240

MEPDGDAAFHRNEAISAVQDVDQYYGDDDDFDDLYNDVNVGDGFLHNSSSSQPPPSQQLPPPSLPPPLPQKQPPSQQLPPPPQQQQPPPQHSLPPPPPLPQAPPPQQQKVHIPGVAAPAPNHPPSQPNLPPPAAPAPLPPQQHQIQQGGGDGFHRPGGNYGGGPIVVGNGGPAVVGGDGPGGTTLFVGELHWWTTDADLEAELIKYGPVKEVRFFDEKASGKSKGYCQVDFYDPAVATACKEAMNGHLFNGRPCVVAFASPNSVRRMGEAQVKNQQSMSAQTSSMQPKGGRGGGGAGSPQVGGNYGGGRGGGPGGGAGGGGGNWGRGGGGMGRGPAGNMRNRMGGPAGGRGIMGNGGMVAPPPPMLPPGGMMGQAFDPTGYGAMGRMGAGFGGFPGAPGAGPFPGLMQPFPPVVAPHVNPAFFGRGGMGAGGVGMWPDPNMGGWGGEEQSSYGDDAASDQQYGEGGSHGKERAERPPDREWSGASERRREREKDIPPAQEWPDRKHRDERDMGRERDRDYDRERERDRDRERERDRDRERDRERDRERERDRHRDDRDRYGDYHRHRDRDSERNEDWDRGRSSGVRSRSREVDHSKRRRMTPE

>Osa:LOC_Os04g39250

MDAGGELTAVDAAFPPAEAIAAVHDSHAPSPTTEDDCDDLYGDVDLGFLPLSPPSHYPTSPPKTPSPGHSALSPSPPPPPRRGPLPDPTAKAEPEPPKPTPQQQPQPLLPAAKPAPPRASPPTTAVFIGELPYWTTDAEVEGALAPHGALHGLHFFTDKLTGKSRGFCRADFLSPDAAASAAAALHGRTFDGRHCVASLSCPPTLLPLGGGGGGDDPHVHAPRAARGRGRGRGGGHGGNSTTARGNVGPPLGDPPALAPPPRPQLPFGGGMLGGGGGAGYGGFAPMIGQCNAAIGTSMMPSVMSPHVNPAFLAASGMAMGGTGMWYDQRMTGMWVGQQPWNFGGYGMPRHQQKPPMQQPNRNGDYGTVRGTARRGRPAGGRNEGDTGNANGNERGYPDRRQCGRGRDGFDLSRKHGHEERGRYRPRVLEEEREHERNWDESDRYGGDRRRYQEYPERDFERRGRVRSRSSSRDGDDDDHPGRHC

>Osa:LOC_Os09g29980

MDPDGDGSFHRNEAISAVQDVDQYYGDDDDYDDLYNDVNVGDGFLQSSHPPPQPPPPPQQQQQPPPISQQPPPLQAPPPPPQQQQQQQQLQAPPSLPPPPPQRQPEKVHIPGVAAVPPAPVPDRPNPVHLPPQPQPPVAAAPPPPPHNQIQPGGGDGFHRQGGGNYGGGPIVVGNGGGGDGPGGTTLFVGELHWWTTDADLEAELSKYGQVKEVRFFDEKASGKSKGYCQVDFYDPGAAASCKEGMNGHLFNGRPCVVAFASPHTVRRMGEAQVKNQQSMAQQNSGVQKGGRGGGAAGGPGGAQVGGNYGGGRGGGGGGPGGGGGGGGGGNWGRGGGGMGGRGQAGNMRNRMGPVGGRGLMGNGGMVAPPPPMLHPGGMLGQGFDPTGYGAAMGRMGGGFGGFPGGPGAAPFPGLMQPFPPVVAPHVNPAFFGRGGGMGAGGVGMWPDPSMGGWGGEEQSSYGDDAASDQQYGEGGSHGKERPPEREWSGASDRRREREKDLPPPPDWPERRHRDERDAGRERERERDRDRERERDRDRERERERDRDRERERYRDDRDRHGDYHRHGKRESDRNEDWDRGRSSGRRSRSREVDHSKRRRMSPE

>HsCPSF6

MADGVDHIDIYADVGEEFNQEAEYGGHDQIDLYDDVISPSANNGDAPEDRDYMDTLPPTVGDDVGKGAAPNVVYTYTGKRIALYIGNLTWWTTDEDLTEAVHSLGVNDILEIKFFENRANGQSKGFALVGVGSEASSKKLMDLLPKRELHGQNPVVTPCNKQFLSQFEMQSRKTTQSGQMSGEGKAGPPGGSSRAAFPQGGRGRGRFPGAVPGGDRFPGPAGPGGPPPPFPAGQTPPRPPLGPPGPPGPPGPPPPGQVLPPPLAGPPNRGDRPPPPVLFPGQPFGQPPLGPLPPGPPPPVPGYGPPPGPPPPQQGPPPPPGPFPPRPPGPLGPPLTLAPPPHLPGPPPGAPPPAPHVNPAFFPPPTNSGMPTSDSRGPPPTDPYGRPPPYDRGDYGPPGREMDTARTPLSEAEFEEIMNRNRAISSSAISRAVSDASAGDYGSAIETLVTAISLIKQSKVSADDRCKVLISSLQDCLHGIESKSYGSGSRRERSRERDHSRSREKSRRHKSRSRDRHDDYYRERSRERERHRDRDRDRDRERDREREYRHR

>MmCPSF6

MADGVDHIDIYADVGEEFNQEAEYGGHDQIDLYDDVISPSANNGDAPEDRDYMDTLPPTVGDDVGKGAAPNVVYTYTGKRIALYIGNLTWWTTDEDLTEAVHSLGVNDILEIKFFENRANGQSKGFALVGVGSEASSKKLMDLLPKRELHGQSPVVTPCNKQFLSQFEMQSRKTTQSGQMSGEGKAGPPGGGSRAAFPQGGRGRGRFPGAVPGGDRFPGPAGPGGPPPPFPAGQTPPRPPLGPPGPPGPPGPPPPGQVLPPPLAGPPNRGDRPPPPVLFPGQPFGQPPLGPLPPGPPPPVPGYGPPPGPPPPQQGPPPPPGPFPPRPPGPLGPPLTLAPPPHLPGPPPGAPPPAPHVNPAFFPPPTNSGMPTSDSRGPPPTDPYGRPPPYDRGDYGPPGREMDTARTPLSEAEFEEIMNRNRAISSSAISRAVSDASAGDYGSAIETLVTAISLIKQSKVSADDRCKVLISSLQDCLHGIESKSYGSGSRRERSRERDHSRSREKSRRHKSRSRDRHDDYYRERSRERERHRDRDRDRDRERDREREYRHR

>CeCPSF6

MAELDEAALLGDGNEQHDGPIDENALLDGKELKEEDIDDLYDEAIAPTNSTESAKPVSPTITTVTAPTAGIGAKPATSSEGRKYCCYVGNLLWYTTDADLLKALQSTGLARSQFADMKFFENRTNGQSKGYALLVLNSDAAVKQTMEILPTKTIHGQSPTVLSYNKTNQAKLEDAQAKNQTRPDVKKKGFEEGCVNMGTIRIGAGGQTGRTGTSVSGRSGPPPLMMQQVRPTPLMSQPTSLPSNLNQAPQMRLQINGQQVPLMNRAPVPQQTMLGNGPLGGMNQQIQQQPQMMMGQQVRPMMQTSMGIQPMMGMNAPPPMNNQFQNRPPQLGSMGVQPLMQMNTAMRPPINGLPPVHVNPQMFPGLQGTVLSDAEFEDVMTRNQTVSSSAIARAITDASVGDIKGASETILTAIQLIKNSRIGHDERCRQLVYGLEHTLKGLESKGYSSRSKSHRDRSRSRERDRKRRRRSRTRSRSYSRSPSPRRRRY

>DmCPSF6

MADVVLDLYAEDLDKDFAGQAQDEFGGDGVDLYDDIGGPTESAASGGGGGGTPSADGAAGPGSGEPGERNSGGPNGVYHQSSGSLTPTMNRRYQLYVGNLTWWTTDQDIANSLRDIGVSDLQEVKFFENRANGQSKGFSVISLGSESSLRAVLDQLPKKEMHGQAPVVTYPSKQALTQFESLQKTRPVPPPQQNGPPRGPAPPSMGGGPMPTGHPGGPQGGGPPGHPPRGMNSIMQPGQYRPQHMSQVPQVGGPNSGPPRMQPPMHPQGGLMGNQQPPPRYPSAQGQWPGQRPGGPRPGPPNGPPQRPMFQGGPMGMPVRGPAGPDWRRPPMHGGFPPQGPPRGLPPAPGPGGPHGAPAPHVNPAFFNQPGGPAQHPGMGGPPHGAPGPQPGMNMPPQQGMNMTPQHGPPPQFAQHGPRGPWPPPQGKPPGPFPDPQQMGPQLTEVEFEEVMSRNRTVSSSAIARAVSDAAAGEYSSAIETLVTAISLIKQSKVAHDERCKILISSLQDTLHGIEAKSYNRRERSRSRERSHRSRQRRERSTSRYRERSRERERDRDRERERDGGSYRERSRSRERERQAPDHYRDDSRSVRPRKSPEPVVAEAAEAPSSKRYYEDRERYRSSDRERRDRDRDRDRERERDRDRREEHRSRH

>HsCPSF7

MGRPESAGGGSRGPFEGGGRARRAGGIFLTLSILRTRDLPSGAMSEGVDLIDIYADEEFNQDPEFNNTDQIDLYDDVLTATSQPSDDRSSSTEPPPPVRQEPSPKPNNKTPAILYTYSGLRNRRAAVYVGSFSWWTTDQQLIQVIRSIGVYDVVELKFAENRANGQSKGYAEVVVASENSVHKLLELLPGKVLNGEKVDVRPATRQNLSQFEAQARKRECVRVPRGGIPPRAHSRDSSDSADGRATPSENLVPSSARVDKPPSVLPYFNRPPSALPLMGLPPPPIPPPPPLSSSFGVPPPPPGIHYQHLMPPPPRLPPHLAVPPPGAIPPALHLNPAFFPPPNATVGPPPDTYMKASAPYNHHGSRDSGPPPSTVSEAEFEDIMKRNRAISSSAISKAVSGASAGDYSDAIETLLTAIAVIKQSRVANDERCRVLISSLKDCLHGIEAKSYSVGASGSSSRKRHRSRERSPSRSRESSRRHRDLLHNEDRHDDYFQERNREHERHRDRERDRHH

>MmCPSF7

MSEGVDLIDIYADEEFNQDSEFNNTDQIDLYDDVLTAASQPSDDRSSSTEPPPPVRQEPAPKPNNKTPAILYTYSGLRSRRAAVYVGSFSWWTTDQQLIQVIRSIGVYDVVELKFAENRANGQSKGYAEVVVASENSVHKLLELLPGKVLNGEKVDVRPATRQNLSQFEAQARKRECVRVPRGGIPPRAHSRDSSDSADGRATPSENLVPSSARVDKPPSVLPYFNRPPSALPLMGLPPPPIPPPPPLSSSFGVPPPPPGIHYQHLMPPPPRLPPHLAVPPPGAIPPALHLNPAFFPPPNATVGPPPDTYMKASTPYNHHGSRDSGPPPSTVSEAEFEEIMKRNRAISSSAISKAVSGASAGDYSDAIETLLTAIAVIKQSRVANDERCRVLISSLKDCLHGIEAKSYSVGASGSSSRKRHRSRERSPSRSRESSRRHRDLLHNEDRHDDYFQERNREHERHRDRERDRHH

>Hrp1p

MSSDEEDFNDIYGDDKPTTTEEVKKEEEQNKAGSGTSQLDQLAALQALSSSLNKLNNPNSNNSSSNNSNQDTSSSKQDGTANDKEGSNEDTKNEKKQESATSANANANASSAGPSGLPWEQLQQTMSQFQQPSSQSPPQQQVTQTKEERSKADLSKESCKMFIGGLNWDTTEDNLREYFGKYGTVTDLKIMKDPATGRSRGFGFLSFEKPSSVDEVVKTQHILDGKVIDPKRAIPRDEQDKTGKIFVGGIGPDVRPKEFEEFFSQWGTIIDAQLMLDKDTGQSRGFGFVTYDSADAVDRVCQNKFIDFKDRKIEIKRAEPRHMQQKSSNNGGNNGGNNMNRRGGNFGNQGDFNQMYQNPMMGGYNPMMNPQAMTDYYQKMQEYYQQMQKQTGMDYTQMYQQQMQQMAMMMPGFAMPPNAMTLNQPQQDSNATQGSPAPSDSDNNKSNDVQTIGNTSNTDSGSPPLNLPNGPKGPSQYNDDHNSGYGYNRDRGDRDRNDRDRDYNHRSGGNHRRNGRGGRGGYNRRNNGYHPYNR

Figure S6A.

>Sbi:Sb02g011680

MEASASTRRSAAGPDPGGAKKPRLAQPPPSRDPRSYAASNGAASAAEQALVDELLGQYRTALGELTFNSKPIITNLTIIAGENLQAAKPIAALICANILEVPSDQKLPSLYLLDSIVKNIGKDYVKHFSARLPEVFCKAYKQVDPSIHHSMRHLFGTWKGVFPLPPLQMIEKELGFQSSANGSSSAAPSRTDSQSPRPSNSIHGILGSGAKTSVIADTGDDIERANRLGTDRSAGRRLEAPDARPNIQRTQRDPFSNSVHEKQAGRDVRGLGFSKIPQQAVVGTGQVRSKPKGQDAIGGPYYASGVGSSGEQFDRRNNFYASKDVRPSGPVRLDSALLPTPSINADRVGRPSSNKSWKHSEEEEYVWDDVHSKAAEYGSSNNVIKGEWMSDDGNAKFASLQRAKWAEAGPVERIDPNTHKLDNLSRFGLAAGQERRISAYMDQEEYLLGKREVEARIDREIRSEGQQFPPPRGSSLWVSQEKTLPDIGLDPRMPRFSNQPAERSTIYTGTMSASITSSVPVGLSGHYAGRSSLDTANSVPIRSTEAFGQQHRYWSSSPPQAHSPSTAPFARQGSPNPAESDFYPSRPLSQLAQNPQEEYSQRALPVLAKDSHEPSLQATQQTQKYPTFQSKSHIKPSDPLQASFSCENSPSLFRPSHLGEVSLPSDSTPISSDLTSASNLLAGLIKSGFKPNNQSDAKLGPSGSLPVASLSLQNTAGENTTLHTQAPDTSRPPLPPGLPPPSSTQSADKAAPLSSLLSSLVAKGLISSPASDSSNAVLSQPNKASSMNAKVVTASAVPLPALKPSVGKVSSNSDSSAPTNASLPKAIEIKMGDLIGLEFKPEKLRKYHEHVISSLFDDQSHLCKTCGNRFRLEEELSLHTSSCGPREPETIYTGIAPKKWYPSKNFYIDGSHEIEDSTEASDGDLGSTEEVCEFMVPADERQSICALCGEPFDDIYSFEKGNWMYKDAVFLDYPKEESSCGNNVEPEEHVPIVHVRCMPRGSNDGMEVN

>Sbi:Sb07g005190

MAGAAASPVGGHVVERFRSRLREEAGGGEPGAAAVVRVYAEALRELTFNCKPVITELTIIAGQHAALAARGIADAVCARVAEVPPDQILPSLYLLDSIVKNIGGEYVEHFATRLQSVFVDAYYRVHPNQYTSMRRLFRTWWPVFPSSVLHGIEDDLQFSPSETNRPTTSTNQTESLSPRLSHGIHVNPKYLEAQHKFKQANVAHQSAARDTTQMTDVQEDLINGLPSNSSRGRPSMFQKSSLQYADNPDQQETFRPLAGTIRVTSPHLLSTHSSDVNLDGPLVNSRRNMSRSPPLDVFPRNVSPKRALERLPPSHSVLGPDPRKLPDRNGRLRLVFDDGVQRSTISMLDEEYRKQSARELIDAYGNCQGRDADERLPKVQRLDPNGMASRSSARNWLTSEEEEYSWEDMSPTLTDRVRSSMPSFPPGTMRAGFPGANAGLLESDVGRHNFPSQITRSSVDGPPYNLEDRITTASHVDISTRRYPSNFGVQNGALLEYQNSEDTLNHGRIDTMPAPPWQKPTGLPLRIQAPQHPSVLDRIPQPANGEMAVKRLDISGTYNGLNVDNIPLVEKHRSSPSAPIEWLPLHHTRSQTLPLIPPDTKHVRSAPNSLEISSFVSQGASSSVFVPRHQYDALDRKTVTGNLAQPPYQHQDLLPSSQQNQGTILGNQAHPHRPTQLHPHPHSHSHHQETFRSFASGMSVSPFQGQGGNATMTPVSVLPTSFSVPPAVPPYGVPPLPPGPPPVPLQMGSSSSQVGGPQPFVSGLLSNLMRHGVISLEPPSQSQDSVGVDFNVDLKLRNESVINALYQDLSRQCKTCGLRFKCQEEHRAHMDWHVTKNRNSKNRKQSSRKYFVTVGEWLRAAETVGNDGVPAFVPSDPVPDRKEEKEIAVPADEEQTACALCQEPFEDFYSDETDEWMYRGAVYMNAPDGNIDGLERSQLGPIVHAKCRSGPSNTS

>Aly:902758

MLEEDDSVRIPSSSSQFKNTNMRELPDASQVEIFDSKLAAENAPGLTIASEATGQPNMSDLLEAVMKSGILSNNSTHGAIKEEISQDEVNPGALTLSAASKPKNLPSALPISLAGDNLLARLKVEQSSAPLVSCAASLTGITSVQTSKENSKASDPLSCLLSSLVSKGLISASKTEQPSAPSITQEYSPDHSTNSSMSVSVVPSDAQPSVLVKKGPSTAPKVKGLTASETSKSEPEDLIGLKFRADKIRELHPSVISSLFDDLPHLCTSCGVRLKQKEELDRHMELHDKSKGELSGKNSKCRVWFPKVENWIAAKAGELEPEDEEVLN

>Aly:338779

MASNGFFSAQRNAHRNANAGTTLKRRIDNNRGYGIGDLQEERNRYVPPQKRFRSQTQPQTNFRSGHIPLYHHHGRNNNNMSRVSSQSYNNYGVDVIASNSSFPLRNNDSNINNYQKPFIVGYGNPNPQIVPLPLPYRKLDDDDSLPEWVPNSRTLTPNFVPNTYVQNPMNHSNMVSVVSQTMSHHQPIVLSKELSDLLSVLRNEKEKETSEASKSDSLPVGLSFDNPSSLNVRHESVIKSLYSDMPRQCSSCGVRFKCQEEHSKHMDWHVRKNRMAKTTMRLGQQPQKSRGWLVSASLWLSAATGEGTVEAAKPSFGGETQKKKEEEKEQQQIVPADENQKNCALCEEPFEEFFSHEADDWMYKDAVYLTKNGRIVHAKCMPAPRPANDTREPSRVMSVTVPSVAKAICLDETEEDIHENLSRSASSVELNGDCVFNPSLSSSAGENDRKLGFRVVSEIRRFTIGN

>Aly:490122

MDSEKILNPRLLSINSSTSSRKGMSVELPQKPPPPPSLLDRFKALLNQREDEFGGSEEVLPPSMDEIVQLYEVVLGELTFNSKPIITDLTIIAGEQREHGEGIANAICTRILEAPVEQKLPSLYLLDSIVKNIGRDYVRYFSSRLPEVFCLAYRQAHPSLHPSMRHLFGTWSSVFPPPVLRKIEMQLQLSSAAHQSSLGASEPSQPTRGIHVNPKYLRRLEPSAAENNLRGINSTARVYGQNSGGYDDIEDRLESPSSLSSTAEGFPRRFNDGANPSNQAFNYGMGRATSRDDEHMEWRRKENLGQGNDHDRPRALIDAYGVDTSKHVTINKPIRDMNGIHSKMVTPWQNTEEEEFDWEDMSPTLDRSRAGEFLRSSVPALGSVRARPRVGNTSDFHLDSDIKNGVSHQLRESWNLSQNFPHSSNHVNTRAGKDLKVMASSVGLVSSNSDFGPPPFDSIQDVNSRFGRALPDGTWSHLNVRGPNSLPVPSAHLHHLANPGNAMSNRLHGKPLYRPENQVSQSHHNDLTQQNQMLVNYLPSSSAMAPRPIQSLLNHVSHGYPPHGSTIRPSLSIQGGEAMHPLSSGVLSQIGSSNQPPGGAFSGLIGSLMAQGLISLNNQPAGQGPLGLEFDADMLKIRNESAISALYGDLPRQCTTCGLRFKCQEEHSKHMDWHVTKNRMSKNHKQNPSRKWFVSASMWLSGAEALGAEAVPGFLPAEPTTEKKDEEEMAVPADEDQTSCALCGEPFEDFYSDETEEWMYKGAVYMNAPDESTTDMDKSQLGPIVHAKCRPESNGGDMEEGSQRKKMRS

>Aly:482627

MQVPSDQKLPTLYLLDSIVKNIGRDYIKYFGARLPEVFVKAYRQVDPPMHSNMRHLFGTWKGVFHPQTLQLIEKELGFNAKSDGSAAVISTARAEPQSQRPPHSIHVNPKYLERQRLQQSGRTKGMVTDTPEIAPNLTRDSDRLERVSSIASGGSWVGPAKTIRRPQRDLLSEPLYEKDIESIAGEYDYASDLPHNSRSVIKKVGSAMITDDGCEKQWYGAMSRSPDLISDQRDGLHTKSRTSNYAMARLANLESSGPSRNIGAPCDSWKNSEEEEFMWDMHSRLSETDVATINPKNELHAPDESERLETENHHLKRPRFSALDPRFDPANSSEQQDPSIFGHWTSSPRSLHDSEVFSSTNATSTAARKGIQPQPRVASSGILPSSGSGSDRQSPLHDSTSKQNVTKQDVRRAHSLPQRDPRASRFPAKNVPRDDSVRIPSSSSQFKNTNMRELPDASQVEIFDSKLAAENAPGLTIASEATGQPNMSDLLEAVMKSGILSNNSTHGAIKEEISQDEVNPGALTLSAASKPKNLPSALPISVAGDNLLARLKVEQSSAPLVSCAASLTGITSVQTSNENSKASDPLSCLLSSLVSKGLISASKTEQPSAPSITQEHSPDHSTNSSMSVSVVPSDAQPSVLVKKGPSTAPKVKGLTASETSKSEPEDLIGLKFRADKIRELHPSVISSLFDDLPHLCTSCGVRLKQKEELDRHMELHDKSKGELSGKNSKCRVWFPKVENWIAAKAGELEPEDEEVLSEPESAIEDGPAVAADETQCACVLCGEVFEDYFSQEMAQWMFKGASYLTNPPANSEASGPIVHTGCLTTSSLQSLEVGNAIKEEIVE

>Gma:Glyma03g34910

MTVVWYFDWVPSDQKLPSLYLLDSIVKNIGRDYIKYFASRLPEVFCKAYRQVDPCVHSSMKHLFGTWKGVFPPQSLQMIEKELGFAPAVNGSASVSATVRSDLQSQRPPHSIHVNPKYLERQRLQQSSRSKGVVSDMTGAVLNSNEDSERPDRAAARPWLDPRINMLNNQHTHRDAFNDSVTEKSIDGSYGSSEYSSVISSNLVSGAGRTGSKLIDLGHDKTWFKTDGGDADTTSGQRNGFGLKRSFSNQEAPKSMNLDAHRQPRQSITNLRNNVMSGNWKTSEEEFTRDEMNNGSGLPTNASSLAVKKGNQSFMSNAVVGMTKFVGQQFDSGQIISQHIRDHSPTLRPIVKVGNLRRSQEKDMQGPLSSMTSLRPKLQQKQLNPSQTEVTATTKLPQSKVSLTRETSEQLTTNNLSAAPVKTGIIPKKSITRSAVASPSSLDPLHNDSSTLPKKPKGKAGQPPQRLSTQPPASSNVSSSSAPILNAAKNNKPNPIANLLSTLVAKGLISAETESPTTVPSVAPKGSKDQTEIITTSCSLPVTSISGSAAVPVSSSDDEVDAATKTCLASPQSTSTEIRNLIGFDFRPNVIREFHPSVIRELWDDIPHHCKVCGIKLKQEELFNRHLEWHATREHGPIKASRSWYAKSSDWIAGKAEYSSESEFNDSVDVHDEKTGSSQLDTMVLADENQCLCVLCGELFEDVYCHERNEWMFKGTIYMNYSDVNSEMESGNVGPIIHAKCLSENSIITNLDND

>Gma:Glyma10g07660

VPSDQKLPSLYLLDSIVKNIGRDYIKYFAARLPEVFIKAYRQVDPSVHQSMRHLFGTWKGVFPPQTLQVIEKELGFTPAVNGSSSASATLRSDSQSQRPPHSIHVNPKYLERQRLQQSSRSQLLIPVTVLSVLLSIQCWPCKDDNLTVIPIDILSCYLGVVDDITGAISNTNDDPEMPGRTLGVGRPWVDPSVTVNIHSRENEQCAHRGAFNDSVLEKSIGASYGSNEYGSNISRNLGLGIRRPGGRVTESGHDKSWYSKSGVVAGTMSGQGNGLGLKYSFLNTEAPKSMILDVHHQPTQNISSTRTSVISASWKNSEEEEYTWDEMNSGLTVHGASTVSNLSKNSWTADDENLVSLVEFGDLGIRLMQNRTGTILKTKIYTLLIYYVYIFLTCRKLKIASKSEILFGLMLIEKSNANSSAARMVNQPFLSNATIGLPGIAGQFHSLGAENPSGQSPLQQRSPSPPVSVHPSHPLQNLAEQDLPQNHKASQFLGGLQSQYIKDSSPAPPPSIQVGNLQSSQLKNLQGPFSSTTFQARHQHQLGSSHNEVTVKTEKPPMSEVPLARETKSNLDTGNLPSRKAGPPRASTLPPASSNVSSASAQTSSDTNNTLNPIANLLSSLVAKGLISAETESPAKVPSELLTRLEEQSDSITTTSSLPVASVSGSATVPVPSTKDEVDDTARTPISLSESTSPGIRNLIGLEFKPDVIREFHSSVVSGLFDNFPHQCSICGHKLRFQEQFNRHLKWHATRESEENGLISASRWYLKSNDWILGKAEYPSENEFTDSVDTYGKEADKSQEDAMVLADEKQCLCVLCGELFEDFYCQETGEWMFKGAVYLANSDSKSEMGIRDVSTGRGPIIHASCLSDNSVSSVLETFKFKASFPPLFAYFELKDFHC

>Gma:Glyma10g39710

MFSQNMILPPENPRPAGFASKPMGNEIAKPPPSILVGRFKALLKQRDDELRATSVPVPPPSTDEIVQIYELLLSELTCNLKPIITDLTIIAEQQREHAKGIADAICARILEVPVDQKLPSLYLLDSIVKNFGQEYIRYFSLRLPEVFCEAYRQVQPSLHSAMRHLFGTWSKVFPPSVLHKIEAELQFSQAVNTQSSTPNPVRASESSRPSHGIHVNPKYLRQLERSTVDSSMFASWFRLNGSMSIRSYYIRIVFSSHILCLGANEASKTHQFLSSSSRLGISSSSPLRIGVDRPLSASIDEYAVDNPGVDYGVAKALGRDVDLTEWQRKLLSNGHQRQSSRALIDAYGSDKSQETSSSKSLLVERLDRNGIDKVLSTSWQNTEEEEFDWENMSPTLIDHSRNNSLLPSTFGFSRERPGVAANATLSEQDTRKGWSSGSQLPPVDDSSAIAEDAFASSTFCRAPPGQVPGSQNQINHSLGSSQPHDAWKISHHPSNIFSNRGRARNLMIPPIDNIRNTDNNPYWVRPAVSRMEAHPSVLPAPFEMRPSGHGVSISTAMSNPLPVLPFPLPFQSISNNPLHLQGGAHPPLPPGRPPAPSQMIPHPNAGAFMPSQQPTVGYTNLISSLMSQGVISLANQLPAQDSVGTEFNPDILKIRHESAVNALYGDLPRQCTTCALRFKCQEEHSSHMDWHVTKNRMSKSRKQKPSRKWFVSDRMWLSGAEALGTESAPGFLPTETIEEMKDHEELAVPAEEDQNTCALCGEPFDEFYSDEMEEWMYRGAVYLNAPLGITAGMDRSQLGPIIHAKCRSESNMATSEDLGLDEKGADEEGSQRKRMRS

>Gma:Glyma19g37600

MKHLFGTWKGVFPPQSLQMIEKELGFAPAVNSSASVSATVRSDLQSQRPPHSIHVNPKYLERQRLQQSSRSKGVVNDMTEAVLNSNEDLERPDRALSAARPWLDPRINMLNNQHTHRDAFNDSVPEKSMDGSSYGGSEYSSVISSNLGSGAGRTGSKLIDLGHDRTWFKTDGGDADTTSGQRNGFNLKHSFSNHEAPKSMNLDAHCQPRQSITNKQNDVMSCNWKTSEEEEFMWDEIDNGLIDHGPNVSKTLSTDTWMADVENLEGEDHLQITRPFGAKVNREISTVKNQLPGLGGHPPSSWQLQNLKPGYSEGFVSSHSALPANASSLTVKKGNQSFMSNSVVGRVKFVGQQFDSGETESPSWQSPLRQQSPSLPGTVHHPHSMQNFADQELPQNLKTSQFLGGPITQHIRDHSLTLRPIVQVGNLRRSQEKDMQGPLSSMTSFRPKLQQKQLDPSQSKVSLTRETSAQLTTNNLSAAPVKSGVIPKKSITCDPDPRKHPSQTGVQPTQSGRPTTLDPLHNDSSTLPKNTQGKAGQPPQRLSTQPPASSNISSSSAPTLNTAKNNKLNPISNLLSSLVAKGLISAETESPTMVPSEVPKGSKDQTEIITTSCSLPVTSISGSAAVPVSSSGDEVDSATKTSLASPQSTSTEIRNLVGFDFRPNVIREFHPSVIRELWDDFPHNCKVCGIKLKQEELFNRHLEWHATREHGPIKASRSWYAESSDWIAGKAEYSSESGFNDSVDVHEQKTDSSQLDTMVLADENQCLCVLCGELFEDAYCHERNEWMFKGAVYMNYSDVNCEMESRNVGPIIHAKCLSENSIVTNLVRPII

>Gma:Glyma19g37610

MERSLDRSREPGPKKPRLIEELNARQLPQRPTAVTTLPSTRFRAYGRDSEISDLGRGGGGGYQPQPPPHQELVTQYKTALAELTFNSKPIITNLTIIAGENLSAAKAIAAAVYDNILEVPSDQKLPSLYLLDSIVKNIGRDYIKYFAYRLPEVFCKAYKQVDPCVHSSMQHLFGTWKGVFPPQSLQMIEKELGFAPAVNSSASVSATVRSDLQSQRPPHSIHVNPKYLERQRLQQSSRSKGVVNDMTGAVLNSNEDSERPDRALSAARPWLDPRINMLNNQHTHRDVFNDSVPEKSMDGSSYGGSEYSSVISSNLVSGAGRTGSKLIDLGHDKTWFKTDGGDPDTTSGQRNGFNLKRSYSNREAPKLTNLDAHRQPRQSTTDIRNNLMSGNWKTSEEEEFMWGEMNIGLTDHGANVSSNLSTDTWMADDENLEGEDHLQITRPFGAKVDREISTAKKQPPGFGGHPPSSWQLQKHHSIDKLNLKPGYSEGFVSTLSGLPANPSSLAVKKGNQSFTSNAVVGMAKFVGQQFDSGETESPSGQSPLRQQSPSLPGAERDMHGPLSSMTSFRPKLQQKQLNPSQTEVTAKTKLPQSKVSLTRETSEQLSTNNLSAVPVKSGIIPKKSISSNLDSREDPSQTGVQPTQSGRPTTLISSGSAVASPSSLDPLHNDSSTLPKKPQGKAGQPPQRLSTQPPASSSVSSSSAPTLNAAKNNKLNPIANLLSSLVAKGLISAETESPTTVPSEAPKGSKDQTEIITTSCSLPVTSISGSAAIPVSSSGDKVDAATKISHASPQSTSTEIRNLIGFDFRPNVIREFHPSVIRELWDDFPHNCKVCGIKLKQELFNRHLEWHAAREHGPIKASRSWYAKSIDWIAGRTEYSSESEFTDSVDLQDKKIDSSQLDTMVLADENQCLCVLCGELFEDVCCHDRNEWMFKGAVYMNFSDVNCEMESRNVGPIIHAKCLSENSVITNSDND

>Gma:Glyma20g28050

MFSQNVILPPENPRPTAFASKPMSNEIAKPLPSILVGRFKALLKQRDDELRVAAGDPVPPASTDEIVQIYELLLSELTCNLKPIITDLTIIAEQQREHAKGIADAICARILEVPVDQKLPSLYLLDSIVKNFGQEYIRYFSLRLPEVFCEAYRQIQPTLHSAMRHLFGTWSKVFPPSVLRKIETELQFSQAVNTQSSTLNPVRASESSRPSHAIHVNPKYLRQLERSTVDSASKTHQFLSSSSSLGISSSSPSRIGVDRPLSASMDEYAVDNSAVRLIERNSPHPAVDYGVAKALGRDVDLTEWQQKHLSNGHQRQSPRALIDAYGSDKSQETSSSKPLLVERLDRNGIDKVLSTSWQNTEEEEFDWENMSPTLTDHSRNNSLLPSTFGFSRERPDTRKGWSSGSQLPPVDDSSAIAEDAFASSTGLDSFVWVGLIRCMKFVHTCLAIGLFSNLFRRTPPGQVPGSQNQINHSLGSSQPHDAWKISHHPSNIFSNRGRARNLMIPPMDNIRNTDNNPYWVRPSMSRMEARPSVLPAPFEMRPSVNVNGHGASISTAMSNPLPVIPFPLPFQSIANNPLHLQGGAHPSLPPGRPPAPSQMIPHPNVGAYMSSQQPTVGYTNLISSLMSQGVISLANQLPAQDSVGTEFNPDILKVRHESAVNALYGDLPRQCTTCGLRFKCQEEHSSHMDWHVTKNRMSKTRKQKPSRKWFVSDRMWLSGAEALGTESAPGFLPTETIEERKDDEELAVPAEEDQNTCALCGEPFDEFYSDEMEEWMYRGAVYLNAPTGTTAGMDRTQLGPIIHAKCRSESNMATSEDLGPDEKGADEEGSQRKRMRS

>:GSVIVT01012646001

MITQIQKMGLSLLNQDASYIKYLNILSVPRDCDLLILNPAMDGDRFVVSARENPRTLGFAPERGPGGSATATAKPMSNEISQKPLVPIVDRFKALLKQREDELRVLSGDDVPPPTTEEIVRLYEIVLSELIFNSKPIITDLTIIAGDHKEHADGIADAICARIVEVSVEQKLPSLYLLDSIVKNIGRDYIKHFSSRLPEVFCEAYRQVHPNLYTAMRHLFGTWSAVFPPSVLRKIEAQLQFSPTLNNQSSGMASLRASESPRPTHSIHVNPKYLEARHQFEHSPVDSNMQHSRGTSSTLKVYGQKPAIGYDEYDSGHTEVISSQARAQRLNSTGSVGRTPFALGADKLLPSSTARVAKSTSPRIGTAGSSSPPAEKFSMDNSPRRVVERASPSHRGFEYGLVRSMGRDEETSDRQRKHWSNDRFETSAAHNLSNGRERQGLRALIDAYGNDRGQRTLNDKPPKVGHLDMNGTDNKVPKKAWQNTEEEEYDWEDMNPTLANRRQCNNILQSSVSPFGSFRTRPGSGALGAAPLESDFNRSKWSGQAQLSMVDDSPVIAEDVVPTTSLGRGSISKPGFGNETKFHGSHYPQESWNLVHRVPQSSQHNRNAKGRGKNFNTPFLGSGISSSAAETISPLISNIPDADAQLRRLPTVASRMGSSSLNSMNVESLFLPELDSKLPQMANRQAGSIPLNGKNQTQVTRLQPQFLPQETHGNFVPSTTAPVSSYSVAPPLNPGYTPQGHAAATSTILLNPVPGVHSSIPIHNISNSSNTGPIVSNQQPGSALSGLISSLMAQGLISLAKQPTVQDSVGIEFNVDLLKVRHESAISALYGDMSRQCTTCGLRFKCQEEHSSHMDWHVTKNRISKNRKQKPSRKWFVSASMWLSSAEALGTDAVPGFLPTETIAEKKDDEELAVPADEDQNVCALCGEPFDDFYSDETEEWMYKGAVYLNAPEGSAAGMDRSQLGPIVHAKCRSESNVVSPEDFGQDEGGNMEEGSKRKRMRR

>:GSVIVT01033783001

MHPIINNRQLSSSETKGQTEKTPLFCFSTPALHSSFQSYPIVPTLKTVEFGVCFISFSHQFSMYIPTRVRVLISRVSNFHLLFDSRRSFDRSREPGFKKPRLAEEAERGPNPNGRPFPQRPGAAPAASRLKTNERDVDRDDLGRGLYQQQHQELVTQYKTALAELTFNSKPIITNLTIIAGENLHAAKAIAATVCTNILEVPSEQKLPSLYLLDSIVKNIGRDYIKYFAARLPEVFCKAYRQVDPSIHPGMRHLFGTWKGVFPLAPLQMIEKELGFPPAINGSSPGIATSRSDSQSQRPPHSIHVNPKYLEARQRLQQSSRTKGAANDVTGTMVNSTEDADRLDRTAGINAGRPWDDLPAKSIQHSHREAIGELVEKKIGAPYGDYEYGTDLSRNPGLGIGRPSEQGHDKPWYKAGGRVVETFSSQRNGFDIKHGFPNYPAPRSANADAHLQPTQSTVNRSNSGMSRSWKNSEEEEYMWDDMNSKMTEHSAANHSKKDRWTPDDSEKLDFENQLQKPQSIYDVGSSVDRETSTDSMSSEQREQGAFGHRMSSLWPLQEPHSTDGLKHSGTSTLILGHSEGYPTQFTLDALPKLIQKAQLGDLQKLLPHNLQSLSPAVPSVPIRHHAPFSPQLQPDPLQPEPSGQAQKTSLPQTSIFEAPSTIENPVLEHSNYPAAESTGKLSTSNLLAAVMKSGILSNSSVSGSIPKTSFQDTGAVLQSVIQPPLPSGPPPAHKSASNLSQRKVERPPLPPGPPPPSSLAGSGLPQSSNVTSNASNPIANLLSSLVAKGLISASKTESSTHVPTQMPARLQNQSAGISTISPIPVSSVSVASSVPLSSTMDAVSHTEPAAKASVAVTQSTSVEVKNLIGFEFKSDIIRESHPSVISELFDDLPHQCSICGLRLKLRERLDRHLEWHALKKSEPNGLNRASRSWFVNSGEWIAEVAGFPTEAKSTSPAGESGKPLETSEQMVPADENQCVCVLCGEVFEDFYSQEMDKWMFRGAVKMTVPSQGGELGTKNQGPIVHADCITESSVHDLGLACDIKVEKDP

>Ppa:Pp1s341_36V6.1

MPRDRHNGDVIGSGPPGNIRMARGSVPERERELMKFGNYPARQLIVERDMLDGDPRRVGHLTMTGREMETDDGPVDRGPGGNREEIASAAEELLELVTQYKSALVELTFNSKPIITNLTIIAGENAHAAHGITKTICDHIITVPKDQKLPSLYLLDSIVKNIGGEYVKYFAARLPEVFCKAYRQVDPSLYTAMQHLFWTWRGVFPQAPLRTIETELQLGPKPGAPASITQPPRAVDPAVRPGHGIHVNPKYLEQRQLIQQTRPSRVDSMSPAETNGDAKLRQDKSTLRENPKTWPETQRAIIGHFNRERLAEPGYNKELTLDYSDYELGRRGVPRPEIGKLRKLERLDQGESGSWLREGDAEKGNGVPDERSQRHPRRNGCRDDPRQSLGARPVGVDGQDYSRTSGPGRGGAFLASQSVDLGSRGRGEVRSRRMDPRIPVGADGRGRRIGLGSEVEEGRGYRIDSRTRSLEAGGRGNRLDTRGMGLESRGLVPDGSGLRPNVRGSVLDNRALRSETVGLGADSRGLRLDRGLGSEVVGRGNWQNEEEEEFQWEDMHPQVQEPDRKAGDSKADEWLSVDRDMIRAGNSNRPGIEPVGGLDDWRRVGSGSQSEQFSGPAAIRGTLRRESEERSRLAMSQSSHGSRLKVEPDMDVMNSGQTRLPPVQVTLPNFSMALSTSNVQPRPGTPPILSPGGRDSVQGGRGAYGLRQNYGSNLGQGPGYNSSSGVPFGQGQNDGQANGVHSSQAVPCSVGLPQVSAGGMLQLPLFGPSQQAGNSSVQVAVISQQSGLVSSEGTQNQYPQQSGSGVVTMQAQLNQFQQATQGGSSLGGNQGGVLPLNTAAISELLKLVQQLPASQNPMQQQQQALPSSQTQSQIVTTGQVLQSVSGAPPLPNGSPPSFLYNSVAQQGGQYTTAQGQVLAPPMLYPSQAGGVPNQPPLPPGPPPASALTGNSGGQGSGGAGVNQFDNLFKSLLAQGLISAPGATAATSVQAAVVDVRGGMNAASTSSSFFNPNVGSVQASSVQAFALSSGLGGSVGPGADSSSRYAGDAAAGSQGVAVKDDPIGTEFKPEVLRERHEVVIDALYNDFPRQCKTCGLRFLEQEAHSKHMDWHVSRNRRQKSQKKVSRKWFVSEKEWLSGTVASSAEAAPSFFAAEVGAGAAKADEGESLAVPADYNQSVCALCGEPFDDFYSDERDEWMYKGAVYMNVPAGGSIEGIDSVNLGPIVHAKCQTESAATADLTEDSEEVQPELMAADSVRMEVDVGGGDDTAMPLLDNFEALESKDENIEMENRKKRVRY

>Ptr:POPTR_0006s11660

MRHLFGTWKGVFPPQPLQMIEKELGLAPAVNGSSAGAAASRSESQSQRPPNSIHVNPKYLERQRIQQSSRAKGVSNVLTVPVANSIEDVEGPDRAVSIDTRRPWVDPPVKTQTLQRSHREALNEPVHEKKKIGAIYEDFEYGSDVSRKSGLGIGRASGRVAEQGQGQENPCYGTSSNAAELISGQRNGFNMKHGFPNYPASKSSMVDLHLQPTQRIGRSETGISANWKNSEEEEYIWDMHSRLSDHNAAGLSNNSRKDHWIPDDSDKMDLERLDGETSSDSLSTEQKEHATIGSRLSSPWKLPESHSTDGLILSGTSTTNTGHVEGYSATVGGVATSSRSSLGRMAVRPRLGSSHIGKAGLASSTNTSLLSTETLGQQKFQSQGAASPSGQSPIRQRPSSPAFQACYPQLQNSGEQDYHQSQSMTQPDYRAQFSGNLLPSNVQLGSLPKLHSEDLQAPSLPSFQLSHQHRLSQRRQPDSKESEAFGQIQRPHLPPVSNFGTSSTSVSSAADHLNPFTAGTSGQSSTSSLLAAVMKTGILSKINSGVVPDRNFQDIGKMPSQSIIQPPLPSGPPPQFSFSEARIESASSAPAQSQDKLPTVSNISQRKDERPPPPLGSPPSSEQTTDAVNKAPNPISNLLSSLVAKGLISTSKSETSSPLPTQVPSQLQKKNPSITSPSSEPISSATLHSSTVGEASIPEPDTKCSVALSQTTKVEIDDLIGLEFKPEVIRELHPPVISSLFEDLPHRCSLCGLQLKLKERLHRHLEWHNQRKPESDGINGPTRGWYADLGHWLTVNDGLPLGVESSCPMDDFEETTECDDKTVLAHEDHCVCVLCGKLFEDYYCEERNKWMFKGAVRMTLPSGDGQMGTAKESAKGPTVHVNCISESSLCDLVLASGIKMEKDVYAST

>Ptr:POPTR_0004s01970

MQPTKLLNPKAATKAAAAAAVTTTMPNELLAQKPSASSVLDKFRSLLKQRQGSAVEDDGGGDGASLRLEDVVEIYETVLNELTFNSKPIITDLTIIAGEQREHGEGIADVLCARIVEAPVDQKLPSLYLLDSIVKNIGREYIRHFSSRLPEVFCEAYRQVDPSLYPSMRHLFGTWSSVFPSSVLHKIETQLHFSPQVNDQSSSLTSFRASESPRPPHGIHVNPKYLRQLDHSTADNHAKGTSSNLKIYGKKPTVGYDEYESDQAEAISSQVGVGRNSPRRFVEALSPSHPLFDYVHSRAIVRDEEANELRRNNYSDDNHNRFEPSARYRLSNGLEHQGPRALIDAYGDDRGKRITSSKPLHIEQLAVNGVHNKVASRSWQNTEEEEFDWEDMSPTLSERGRSNDFLPSSIPPFGSVVPRPAFGRLSAIHAESDIRSNRSTWNFPPHIHQSAHLLNSKGRGRDFQMPLSGSGVSSLGGENYSPLAEKLPDIDAQLNRPPAIASRWGSNIDSTSSGTWSSVAPPSSGVWPPVNARKSLPPPHAALNQQNQAHVNPFQPQQLPSHEARENFHPSGVTSMPPRPLAPPLNHGYNTHGHSTAISMVPSNALPAVQLPLPVNNIPNISGVPGQPSGSAFSGLFNSLMAQGLISLTKQTPVQDSVGLEFNADLLKLRYESAISALYGDLPRQCTTCGLRFKCQEEHSTHMDWHVTKNRMSKNRKQKSSRNWFVSASMWLSGAEALGTDAAPGFLPTETAVEKKDDDEMAVPADEEQSTCALCGEPFDDFYSDETEEWMYRGAVYLNSSNGSTAGMDRSQLGPIVHAKCRSDSSVVPPEDFGHDEGGNSEEGNQRKRMRS

>Ptr:POPTR_0011s00540

MQSTKLLNPKTATKAAEAVTNTMPNELLPQKSPASSIMDKFRYLLKQRQQSAVEEGGGLSTEDMVEIYETVLNELTFNSKPIITDLTIIAGELREHGEGIADALCGRIVEVPVDLKLPSLYLLDSIVKNIGREYIGYFSSRLPEVFCEAYGQVDPRLYPSMRHLFGTWSSVFPSSVLRKIETQLQLSSQINNQSSSLTSLKASESPRPSHGIHVNPKYLRQMDSSRDNNVQHTKGTSNLKMYGHKPAVGYDEYETDQAEVISSQVGVDRASLTLGSNKLQPSSTSRLARRLSPSTTGAERPSSSEIDDFAAGNSPRRFVEGLSPSHPPFDYGHGRVVVRDDETNELRRKHYSDDNHYRFEASARSLSNGHEQQGPRALIDAYGDDRGKRIPNSKPLHIEQLAVIGMHNKVAPRSWQNTEEEEFDWEDMSPTLLDRGRSNDFLPPSVPPFGSVVPRPGFGRLNAIRADSDIRSNGSSLTPMALVDDSSNMGGDAVSILGSGRGSTSKMPGLLTERNQISGSRYSQEARNLPPHIRQPSRLLNAKGRGRDFQMPLSGSGVSSLGGENFNPLVEKLPDMDAKLVRPPAIASRLGSSIDSNSSGTWSSAVLPLSGAWPPVNVHKSLPPPVHSTFPPEKQSRSQFDPVNTSSTVTNQALQKASVMPEQSFNSFESKDYVLMKPTPLPNQHAALNQQNQAHFNPFQPKFLPSHEARENFHPSGIALLPPRPLARPMNHGYTTHGHGSSNALPSVQLPLAVSNVPNTLHSQVGVRPPLPQGPPQTMPFPQNASSGAPAQPSGIAFSGLINSLMAQGLITMTKQTPVQDSVGLEFNADLLKLRYESAISALYSDLPRQCTTCGLRLKCQEEHSSHMDWHVTKNRMSKNRKQNPSRKWFVSASMWLSGAEALGTDAVPGFLPTETIVEKKDDDEMAVPADEEQSTCALCGEPFDDFYSDETEEWMYKGAVYLNAPDGSTADMDRSQLGPIVHAKCRSDSSGVPSEDFGHEEGLAAKLNHGNTSDFGVGNTEEGSRKRMRS

>Ath:AT2G36480

MENPRRPFDRSRDPGPMKKPRLSEESIRPVNSNARQFLSQRTLGTATAVTVPPASSRFRVSGRETESSIVSDPSREAYQPQPVHPHYELVNQYKSALAELTFNSKPIITNLTIIAGENVHAAKAVVTAICNNILEVPSDQKLPTLYLLDSIVKNIGRDYIKYFGARLPEVFVKAYRQVDPPMHSNMRHLFGTWKGVFHPQTLQLIEKELGFNAKSDGSAAVVSTARAEPQSQRPPHSIHVNPKYLERQRLQQSGRTKGMVTDVPETAPNLTRDSDRLERVSSIASGGSWVGPAKVNNIRRPQRDLLSEPLYEKDIESIAGEYDYASDLPHNSRSVIKNVGSRITDDGCEKQWYGATNRDPDLISDQRDGLHSKSRTSNYATARVENLESSGPSRNIGVPYDSWKNSEEEEFMWDMHSRLSETDVATINPKNELHAPDESERLESENHLLKRPRFSALDPRFDPANSTNSYSSEQKDPSSIGHWAFSSTNATSTATRKGIQPQPRVASSGILPSSGSGSDRQSPLHDSTSKQNVTKQDVRRAHSLPQRDPRASRFPAKQNVPRDDSVRLPSSSSQFKNTNMRELPVEIFDSKSAAENAPGLTLASEATGQPNMSDLLEAVMKSGILSNNSTCGAIKEESHDEVNPGALTLPAASKPKTLPISLATDNLLARLKVEQSSAPLVSCAASLTGITSVQTSKEKSKASDPLSCLLSSLVSKGLISASKTELPSAPSITQEHSPDHSTNSSMSVSVVPADAQPSVLVKGPSTAPKVKGLAAPSETSKSEPKDLIGLKFRADKIRELHPSVISSLFDDLPHLCTSCSVRLKQKEELDRHMELHDKKKLELSGTNSKCRVWFPKVDNWIAAKAGELEPEYEEVLSEPESAIEDCQAVAADETQCACILCGEVFEDYFSQEMAQWMFKGASYLTNPPANSEASGPIVHTGCLTTSSLQSLEVGIAIKQEIVEAKRPSQYRTFPYSMPFFRVSQKNMRVSYESETQICHVNIGERAKLTFIGGRKMQKV

>Ath:AT4G04885

MDSEKILNPRLVSINSTSRKGMSVELPQKPPPPPSLLDRFKALLNQREDEFGGGEEVLPPSMDEIVQLYEVVLGELTFNSKPIITDLTIIAGEQREHGEGIANAICTRILEAPVEQKLPSLYLLDSIVKNIGRDYGRYFSSRLPEVFCLAYRQAHPSLHPSMRHLFGTWSSVFPPPVLRKIDMQLQLSSAANQSSVGASEPSQPTRGIHVNPKYLRRLEPSAAENNLRGINSSARVYGQNSLGGYNDFEDQLESPSSLSSTPDGFTRRSNDGANPSNQAFNYGMGRATSRDDEHMEWRRKENLGQGNDHERPRALIDAYGVDTSKHVTINKPIRDMNGMHSKMVTPWQNTEEEEFDWEDMSPTLDRSRAGEFLRSSVPALGSVRARPRVGNTSDFHLDSDIKNGVSHQLRENWSLSQNYPHTSNRVDTRAGKDLKVLASSVGLVSSNSEFGAPPFDSIQDVNSRFGRALPDGTWPHLSARGPNSLPVPSAHLHHLANPGNAMSNRLQGKPLYRPENQVSQSHLNDMTQQNQMLVNYLPSSSAMAPRPMQSLLTHVSHGYPPHGSTIRPSLSIQGGEAMHPLSSGVLSQIGASNQPPGGAFSGLIGSLMAQGLISLNNQPAGQGPLGLEFDADMLKIRNESAISALYGDLPRQCTTCGLRFKCQEEHSKHMDWHVTKNRMSKNHKQNPSRKWFVSASMWLSGAEALGAEAVPGFLPTEPTTEKKDDEDMAVPADEDQTSCALCGEPFEDFYSDETEEWMYKGAVYMNAPEESTTDMDKSQLGPIVHAKCRPESNGGDMEEGSQRKKMRS

>Bdi:Bradi4g38387

MRASMEAAPSSSSAARRSAAPDPAAANKKQRLAQAPRDPRSYATNGAAAAASAEQQVQVDELVAQYRTALGELTFNSKPIITNLTIIAGENLHAAKPIAALICANILEVPSEQKLPSLYLLDSIVKNIGKDYIKHFSARLPEVFCKAYRQVDPPVHTSMRHLFGTWKGVFSQASLQMIEKELGFQSPANGSSGAASSKPDSQSQRPSHSIHIPFSPQFGQGLLGAGAKTSVMSDASDDIERVNRVAIDKGAGRRLDVVNSRPRAQRDPFSNPIHEKPDRDVRALGFSNISQQPVVGAGQFRSKSKGQDGPVGPYYTGGLSSSEEQFDRRNNLYANRDARPSGSVRLDNALLPTPVSNSDRIIKPSSNKSWKNSEEEEYMWDDVRSQGADYGGASSARKGERMVDDGSIIGFQGAKWADPGDQLDPDFHKPDIIPRFGHATGQDRRIAAYMDPEEYIHGKREVEPRIDREMWPEGQKFLEPRSSLWLSQEKMHPDVGRDPRISRFSNQSASITSSPPIGLSGAYAGRSSLESATSGPTTFGEQKHKYWQSSSPPVHSASPTASFARQGSPSPAEHDIYTSRSFLPLGQNLQEEHNQRAHALSQNAAHSQGRPSMKATVSQASQQTQKHPSVQPKPHLKPSDQLQTHLPHENSSSLFRSSVHLPLSSGMGHHQPEVSSPSDSTHVNSDQMSASNLLAGLIKSGFKPKPNDHASLRAQPPLPSAPHQHGSTSLPVASASENATSKPHALNSVRPPLPPGPPPTQNAEKAAPLSSLLSSLVAKGLISSPSTNLSAAVPQKPSKSSLSASDVNATPPLLPISQPSVDKDAPTKTLLPQHVEIKMADLIGLEFKPEVLRKYHAHVVNGLFDDQSHQCKTCGLRFSLEEELSAHTVGCGSGLSETRNTGIAPERWYPSKNNWIDGSHEAENIFLDSDVDASDSESGPAEVCEFMVPADESQIICLLCGEQFDDIYSIDRGDWMYKDAVYFDYSKVEGSCGGSVESKGSAPIVHARCMPRIANDGMDVD

>Bdi:Bradi3g15320

MASAAAAPPVAGKVVERFRARLREEAGEEAGAAAAAVVGVYGEALAELTFNCKPIITDLTIIADQHAALAARGIADAICARIVEVPVEQKLPSLYLLDSIVKNIGREYIGHFAARLQKVFCYAYRNVHPNQHAAMRHLFRTWSQVFPSSVLRGIEDELQFSPSENKFPARATNLRQSESLSPRPSHGIHVNPKYLEAQQQFKHASKADQLAPRARQMTDVGEDHVNGLTSRSLLGFPATSSKLQRSTILYADDPDQQETFRSRTGMTTRDMSRSPPLDILPRNASPKRKLERLPLSHSVSGHDPRRLPNRNGWFERQWAFEDGAQRPSMSTLDEEHRKQSARELIDAYGNSQGNDADERLPKMQRLESNGMASRSSAQQWLNSEEEEYSWEDMNPAFRNRSSMPSLPPSETLRTGFPGLNTGLLDSDIGMRSWESQATRPSADRPSLHLEDRITATGHVDMATSRRYPSNLGPQNGTISEYHTSENTLDPGRLLAMSAPSWQHTNGLPLRVQAPQPSSTLDRLSLPADGEMPVKRLPAGGTYALIEKHRPSPAPAPIEWPPLVHGQPPSFTNHARRAKDSLEIRPFINQGVNSSVFVPRHQYDASDQNTVSTGNLAQPPYQQPDLLSSSQQNQGTMGNQSQTHHARQFHPHSLSHPQEAFRSFAPNMPVGMSQNPFQGQGGSAATPPVPTLPNSFSLPPAVPPYGVPSVPNFAPPPLHCGLPPASLQMGPSSSQVGGPTTYFSGILSNLMHQGVISLEPPSQPQDSIGVDFNVDLKVRNESVINALYQDLSRQCKTCGLRFKCQEEHRAHMDWHVTKNRNSKNRKQSSRKYFVTVREWLRAAETVGNDGVPSFEPSEPVPDKNEEKEMAVPADEDQTSCALCQEQFEDFYSDETEEWMYKGAVYMNAPDGNILGLERSHLGPIVHAKCRSGPNNTS

>Osa:LOC_Os08g08830

MAAAAAVAGAGGVGHVVERFRARLREEQEEEEGGGGEVAAAAVVRVYVEVLSELTFNCKPIITELTIIAGQHAALAARGIADAICARIAEVSADQKLPSLYLLDSIVKNIGREYVGHFAARLQKVFCDAYRKVHRNQHAAMRHLFGTWSQVFPSSVLRGIEDELQFSPLENKRSATATDIRQSESISPRLSHAIHVNPKYLEAQQQFKQSTSVHQPITRGNRQMNDVEEDQINGLTSKSSRGWPATNSKLQKSTMLYADDLDQQEAFCSHTGLIRPSSPHLLSKHPSILNTEGPLANSRRTMSRSPPLDVLPRNASPKRALERPPLSHSVLGPDPRRLPDRNGWFERKWAFEDGAQRPSMSILDEEYRKQSARELIDAYGNSQGKDVDERLPKMQRLDSNGMAGRPSAQKWLTSEEEEYTWEDMSPTLTDRNRTSVPSLPPLGTLRAGFLGPNSGLLESDIVRHSWPGQAPRPAIDGPPLNLEDRIPTNGPVDRTNNRRYPGNFGVQNGAFLDYQSSEHTLDPGRTTMPVPPWQQTIGQPLRVQAPQPASILNRMPLPTDSEVPVKRLATGGTYDALNVDIPLLEKQRSSPAHAPMEWPLNTQSLTIQPIPPDTKHPRGASDGLDSRPFISQGSSSSVFVPQHHALDRRTMNADDLAQPSYQHPDLLSLSQQNQGTVLGNQGQPHHPPQFHPHPHSHLQETIRSFAPSMSVAPPQNIFHGQGGSAAALLPSSFPVPPAVPPYGLQSMPGFPLPSLPSGPPPPSQIGPSSSQVGGPPLVSGLLSNLMQHGIISLQPPSQPQDSVGVDFNVDLKVRNESVINALYQDLPRQCKTCGLRFKCQEEHRAHMDWHVTKNRNSKNRKQTSRKYFVTVGEWLRAAETVGNDGVPSFEPAEPVADAKEEKELAVPADEDQTTCALCQEPFEDFYSDETEEWMYKGAVYMNAPDGNIGGLERSQLGPIVHAKCLSGPNNT

>Osa:LOC_Os09g39270.

MRASAAPMETSASARRSAAPDPKKPRLAQHPAPRDPRSYPAAANGNAPAVDQAQVDELVAQYRTALGELTFNSKPIITNLTIIAGENLHAAKPIASLICANILEVPSEQKLPSLYLLDSIVKNIGKDYVKHFSARLPEVFCKAYKQVDSSIHNSMRHLFGTWKGVFSPTSLQVIEKELGFQSSTNGSSGAAPSKPDSQSNRPSHSIHVNPKYLEARQQLQQPNKGQGILGAGAKTTTISDSGDDIERTSRTAVDRGAGRRLDALNPRTNVQRAQRDPFSNPIHEKQDRDMRVLGFSNISQQAVVGTGLVRAKPKGQDGSGGPYYTAGVGSSEEQFDRRSNFYANKDVRPSGSVRLDGALLPTPVSNSDRIGRPSSNKSWKNSEEEEYMWDDVRSQGADYGGTSSTRKREWIPDDGNVGSFQRVKWAEAGGPLDPDQHKLDSFQRFGNATGQDRRITPYMDHEEYLHGKHEVEPRIDREMLPEGQPFSSSRGSSLWLSHEKPLPDIVSDPRISAFSNQPADRPTIYAGTLSTSITSSVPVGLSAAYAGRSSLESAATRSTETIGQQKNRYWSTSSPPVQSPSASFARQSSPSPVELDYSSKPFSQLGQNSLEDYNQRTHALAQNLALSQGRPNLLGAPSHASQQIEKHPSLLQSKPHLRTLDQPQANFSPENSSSVFKSSIQLPISVGVGHRQPEEVSLSSDSTLMSSDHLSASSLLAGLIKSGFKPNDPNDLASLRAQPPLPSGPPPHVSTSFSAASSSLHLPASDTLKSQAPNSLRPPLPPGLPLSSPFVCPTTQTSEKAAPLSSLLSSLVAKGLISSPSADSTVAIPQQPCKSELNTPDDTASAPSLPFVQPSVKKETSNQNSSAPSKVLVHQPVEIKKEPAEIKMVDLIGFDFKPEMLRKYHAHVISTLFDDQSHQCNTCGLRFSLEEELSVHTACHGSKQSETRKTGIAPEKWYPSKNNWVDRSHEVQNSALESASSVADLSSEEEVCEFMVPADESQIICALCGESFDDIYSIEKDNWMYKDAVYFDSSKTEGSSGDSAESKERVPIVHARCMSISSNDGMEVD

>Ptr:POPTR_0017s04910

MATTLIARKDHHRPRAVPNLHNPVLDSEKRSRTSYMAQQQQGGDYYHRNQLGLQAKKPRVDRFNNNEAVNFGPKIQSFPQFEGKKVEINYGVSGFDARVQAREPELSNNGVLTPNFRPLPQLQDQKAEGNNGFFMPSLQAWKPEGNNFVVSRPYPPLSQPFQGKKLGFYNGFSVPNYQVSRHFQDSSLKVDNAVFNPNHAMQQDQEKKSEVNKGGFMPNLNLFSSKSLGGPLYSMPNPAIVKPMLNQNVTNMCLLNSQSHSSLASSVNKNGGFTPNPPVMQLNLVNLSATNMPLNSQAQVASCYSDLLTTLVGNGVVSLAKHEVQSNNANSCKFNCAPKLLETSNQLVANGCPGLRNSLMSSLIKKEDDFRGGVVFDANQLKVRHESVIRSLYADMPRQCSTCGIRFKFQEDHSKHMDWHVIKKRTIKISKQRSISRMWLDGVDMWLAARADVAAVPGFAKADAPVEKEKEEDWMSSTDENKVCALCREPFEEFYSHEADDWIFRGAVYLNAEKKSAAESMDRSRLGPAVHAKCRPASK

>Ptr:POPTR_0007s01480

MAAIFTTGTDHHYNPVLDSHKRSRTIYMNQQQQGGDYYHSNPRWFQVKKPRVDHYNSNNGVFNFGPKIQPLPQFEAKKVEINNGVSGFNARVQDQKPRLNYNGVSTLMSQALPEFQTKRSEANNGVFVPNLQAWKPEANNSVFARPYSTVSKQFQGMKQRFYDGVSVPNPQVSRQFQGRSLEADIVVFKQNLPALQQIQANKPDFNNGVPLPNPVLYNSKSLRSPVYSIPNPAFVKPMLNRNVTNMCLLNSPSQNSLVSSGLLTNPSFLQSNSLNDVKSISTNSEYLAAPMHSGFFTKANFFNNNGGFTPNPPVMQPNLVNQNAINLPLNSQDQVACVYSDLQTSMVGNGVISLAEHEVQSNNPDTARKFNCAPRLQETSNQLVANECSGLINSLLNSLMKEDDFSGGVVFDANQLKVRHESAIRSLYADMPRQCSTCGTRFKCQEDHREHMDWHVIRNRKARISKHQMQKQNQRIRIWKKKMKIG

>Ath:AT1G66500

MASNGSFSAQRNANARTTMKRRSDNRGYGGGIGGYQEETNRYAPPQKRFRSQAQQQFRSGHNPLYHHHGSNNNNVSRVSSQSYNNCGVDVIASNSSFALRNNDSNTNNYQKPFVAGYGNPNPQIVPLPLPYRKLDDNLSLDSLPDWVPNSRTLTPNYPVRSSNFVPNTPVFTNVQNPMNHSNMVSVVSQSMHQPIVLSKELTDLLSLLNNEKEKKTLEASNSDSLPVGLSFDNPSSLNVRHESVIKSLYSDMPRQCSSCGLRFKCQEEHSKHMDWHVRKNRSVKTTTRLGQQPKKSRGWLASASLWLCAATGGETVEVASFGGEMQKKKGKDEEPKQLMVPADEDQKNCALCVEPFEEFFSHEDDDWMYKDAVYLTKNGRIVHVKCMPEPRPAKDLREPSRVMSVTVPSVAKAIAC

>Ath:AT5G43620

MASNGSFSAQRNANAGTTMKRRNDNRGYGGGIGCYQEERNRYAPPQKRFRSQLQQQFRSGHNPLYHYGSNTNNNVSRVSSQSYNNYGVDVIASNSSFALPNNDSNTNNYQKPFVVYGNPNPQIVPLPLPYRKLDPLDSLPQWVPNSTPNYPVRSSNFVPNTPDFTNVQNPMNHSNMVSVVSQSMHQPIVLSKELTDLLSLLNNEKEKKTSEASNNDSLPVGLSFDNPSSLNVRHESVIKSLYSDMPRQCTSCGVRFKCQEEHSKHMDWHVRKNRSVKTTTRLGQQPKKSRGWLASASLWLCAPTGGGTVEVASFGGGEMQKKNEKDQVQKQHMVPADEDQKNCALCVEPFEEFFSHEADDWMYKDAVYLTKNGRIVHVKCMPEPRPAKDLREPSRVMSVTVPSVAKAILC

>Bra:Bra004171

MVSVVSQSLPQQPVVSKELADLLSVLNNNEKEKECDLGLDFDKINLNVRHESVVKSLYSDMPRQCSSCGVRFKCQEEHSKHMDWHVRKNRMAKDATKAATTTRASQKPKKSRDWFASLSLWLSAATGAAIEGAKPLFGETQKTKEEEKQQQRYVPADENQKMCALCLESLEEFFSHEEDDWMYRDAAYLNMNGSGPIVHVNCMPEPRKGPAKGLTKPVASAVAC

>Homosapiens

MSEQTPAEAGAAGAREDACRDYQSSLEDLTFNSKPHINMLTILAEENLPFAKEIVSLIEAQTAKAPSSEKLPVMYLMDSIVKNVGREYLTAFTKNLVATFICVFEKVDENTRKSLFKLRSTWDEIFPLKKLYALDVRVNSLDPAWPIKPLPPNVNTSSIHVNPKFLNKSPEEPSTPGTVVSSPSISTPPIVPDIQKNLTQEQLIRQQLLAKQKQLLELQQKKLELELEQAKAQLAVSLSVQQETSNLGPGSAPSKLHVSQIPPMAVKAPHQVPVQSEKSRPGPSLQIQDLKGTNRDPRLNRISQHSHGKDQSHRKEFLMNTLNQSDTKTSKTIPSEKLNSSKQEKSKSGEKITKKELDQLDSKSKSKSKSPSPLKNKLSHTKDLKNQESESMRLSDMNKRDPRLKKHLQDKTDGKDDDVKEKRKTAEKKDKDEHMKSSEHRLAGSRNKIINGIVQKQDTITEESEKQGTKPGRSSTRKRSRSRSPKSRSPIIHSPKRRDRRSPKRRQRSMSPTSTPKAGKIRQSGAKQSHMEEFTPPSREDRNAKRSTKQDIRDPRRMKKTEEERPQETTNQHSTKSGTEPKENVENWQSSKSAKRWKSGWEENKSLQQVDEHSKPPHLRHRESWSSTKGILSPRAPKQQQHRLSVDANLQIPKELTLASKRELLQKTSERLASGEITQDDFLVVVHQIRQLFQYQEGVREEQRSPFNDRFPLKRPRYEDSDKPFVDSPASRFAGLDTNQRLTALAEDRPLFDGPSRPSVARDGPTKMIFEGPNKLSPRIDGPPTPASLRFDGSPGQMGGGGPLRFEGPQGQLGGGCPLRFEGPPGPVGTPLRFEGPIGQAGGGGFRFEGSPGLRFEGSPGGLRFEGPGGQPVGGLRFEGHRGQPVGGLRFEGPHGQPVGGLRFDNPRGQPVGGLRFEGGHGPSGAAIRFDGPHGQPGGGIRFEGPLLQQGVGMRFEGPHGQSVAGLRFEGQHNQLGGNLRFEGPHGQPGVGIRFEGPLVQQGGGMRFEGPSVPGGGLRIEGPLGQGGPRFEGCHALRFDGQPGQPSLLPRFDGLHGQPGPRFERTPGQPGPQRFDGPPGQQVQPRFDGVPQRFDGPQHQQASRFDIPLGLQGTRFDNHPSQRLESVSFNQTGPYNDPPGNAFNAPSQGLQFQRHEQIFDSPQGPNFNGPHGPGNQSFSNPLNRASGHYFDEKNLQSSQFGNFGNIPAPMTVGNIQASQQVLSGVAQPVAFGQGQQFLPVHPQNPGFVQNPSGALPKAYPDNHLSQVDVNELFSKLLKTGILKLSQTDSATTQVSEVTAQPPPEEEEDQNEDQDVPDLTNFTVEELKQRYDSVINRLYTGIQCYSCGMRFTTSQTDVYADHLDWHYRQNRTEKDVSRKVTHRRWYYSLTDWIEFEEIADLEERAKSQFFEKVHEEVVLKTQEAAKEKEFQSVPAGPAGAVESCEICQEQFEQYWDEEEEEWHLKNAIRVDGKIYHPSCYEDYQNTSSFDCTPSPSKTPVENPLNIMLNIVKNELQEPCDSPKVKEERIDTPPACTEESIATPSEIKTENDTVESV

>Caenorhabditis elegans

MESVESAARDYRETLAELRNNNKTQINLLTILADDFKKAAPQIVEVIERHLTTCSPSQKLLVMYVCDSILKNVKKPNDYDALFARKIVSMFEHAFRQGDERIRTSLYRIRVTWASTTLFMPSKLYELDMKINKLDPNWPISNPQTGRALRDDPQVMAPSQSRPAGNATSPAASTSTNRVFVNPKFIGSSTPGAASASKTVVEKTKSPGTVNKEKQVKKEPKQDPLDKLLPSSSASKTSSSPAGLKRKSAPSEHPNAPIRKKPQQPPKPQTAIDEDLRSISLTKKPPVPSAQDQDFRPKTLKPTSVIGSHAFAPVAAPIRPMIPVPPPVSVAPFVPAPPLSSAPPFQHPQQHHPQLPPPPVHQGMGRGYHHNSPPQDPAPIVPVQAPPPQQHLPAPENVYSSEQPKLDVPANNRIFVDGKAYEVMFVDDTAVIERGGAPHRIYFAGPPRNLVIDGIPHLLQFDTPTQIDILGSKHMVKFGAPSRELYIGGHPFKGQFGGPPIIATINGRRHEIRLTGSAPEVRIEPEPAYHLTHFLHKMREEKKIEIASEKPEKKEDWLSYLKNLRTRNILPAPARSPSSPRNQTPPPANLPIPGMNNAGQRGGYHNRHQPQQNARWGGANKQQNIPPPPSDPSPIGSGVEKRSAPPAAITDFNIRLLQIRYDSVVDALITKRADACKFCGMRLDDSQGKSKEWQDHMDWHVKQNLARHGSNNSAAVPYRQWYPSTSTWLTPRASDETNEQEADKPEEPLPGVASSGVKTKECSVCGEKFDEYYDDDEETWRLRDTVNVHGKIVHSACASDAARSLDNSSFFNDSDIKKEEPFD

>Mus musculus

MSEQTPAEAGAAGAREDACRDYQSSLEDLTFNSKPHINMLTILAEENLPFAKEIVSLIEAQTAKAPSSEKLPVMYLMDSIVKNVGREYLTAFTKNLVATFICVFEKVDENTRKSLFKLRSTWDEIFPLKKLYALDVRVNSLDPAWPIKPLPPNVNTSSIHVNPKFLNKSPDEPSTPGTVVSSPSISTPPIVPDIQKNLTQEQLIRQQLLAKQKQLLELQQKKLELELEQAKAQLAVSLSVQQETANLGPGSVPSKLHVPQIPTMAVKTPHQVPVQPDKSRAGPSLQMQDLKGTNRDPRLNRMSQHSSHGKEQSHRKEFVMNTINQSDIKTSKNVPSEKLNSSKQEKSKSGERITKKELDQLDSKSKSKSKSPSPLKNKLSHTKDLKNQDSESMRLSDMSKRDPRLKKHLQDKAEGKDEDVKEKRKTAEKKEKDEHMKSSEHRVIGSRSKIINGIVQKQDMVTEELEKQGTKPGRSSTRKRSRSRSPKSRSPIIHSPKRRDRRSPKRRQRSMSPNLAPKAGKMRQSGLKQSHMEEFPPPSREERNIKRSAKQDVRDPRRLKKMDEDRPQETAGQHSMKSGGDPKENIENWQSSKSAKRWKSGWEENKSLQQGDEHSKPPHLRHRESWSSTKGILSPRAPKQQHRLSVDANLQIPKELTLASKRELLQKTSERLASGEITQDEFLVVVHQIRQLFQYQEGVREEQRSPFNDRFPLKRPRYEDSDKPFVDGPASRFAGLDTNQRLTALAEDRPLFDGPGRPSVTRDGPAKMIFEGPNKLSPRIDGPPTPGSLRFDGSPGQMGGGGPMRFEGPQGQLGGGCPLRFEGPPGPVGTPLRFEGPIGQGGGGGFRFEGSPSLRFEGSTGGLRFEGPGGQPVGGLRFEGHRGQPVGGLRFEGPHGQPVGSLRFDNPRGQPVGGLRFEGGHGPSGAAIRFDGPHGQPGGGGGIRFEGPLLQQGVGMRFEGPHGQSVAGLRFEGHNQLGGNLRFEGPHGQPGVGIRFEGPIVQQGGGMRFEGPVPGGGLRIEGPLGQGGPRFEGCHSLRFDGQPGQPSLLPRFDGLHGQPGPRFERTGQPGPQRFDGPPGQQVQPRFDGVPQRFDGPQHQQASRFDIPLGLQGTRFDNHPSQRIESFNHSGPYNDPPGNTFNVPSQGLQFQRHEQIFDTPQGPNFNGPHGPGNQNFPNPINRASGHYFDEKNLQSSQFGNFGNLPTPISVGNIQASQQVLTGVAQPVAFGQGQQFLPVHPQNPGAFIQNPSGGLPKAYPDNHLSQVDVNELFSKLLKTGILKLSQPDSATAQVTEAVAQPPPEEDEDQNEDQDVPDLTNFTIEELKQRYDSVINRLYTGIQCYSCGMRFTTSQTDVYADHLDWHYRQNRTEKDVSRKVTHRRWYYSLTDWIEFEEIADLEERAKSQFFEKVHEEVVLKTQEAAKEKEFQSVPAGPAGAVESCEICQEQFEQYWDEEEEEWHLKNAIRVDGKIYHPSCYEDYQNTSSFDCTPSPSKTPVENPLNIMLNIVKNELQEPCESPKVKEEQIDAPPACSEESVATPTEIKTESDTVESV

>Drosophila melanogaster

MEQLFQNYRDDERRIGEEYLSSLQDLNCNSKPLINMLTMLAEENINYAHIIVKVVEYYISQVAPEFKLPILYLIDSIVKNVKSSYVQLFGQCIVNIFLHAFESVQHSQSQVLEKVRERMYALRQTWNEVFPPSKMYALDVKVKRLDNNWPITAKQPTNKIHVNPAIHVNPDFLKPGLVPGMPGNPTITSDMEEILQAKTRELLELKKRKLELELEQTKKHLEEQERQLTQTTDAMVGAPIIMPAPTPAAICPPITDPGIAVHNQRAPGVMGNVGPVMHNMPVAPQLFANKPKVHPVNPALLNSVRQRDPRLARQMHAASSHPAAPARNDPRLEAKSSSSQKSSRSRSKSPVRNSSSRSGKSGSSSHSSSSSRKRSESKSSTTSSSSSSSDVRHKGVTASSSNQPTVKRSGKITSKDSDRYVRNGSPLGSAKRKSSSPSSSPSKSKRSTSHKSSSSRGKTSSLRSRSRSPVFMDVDLRTGVRSKSPESRAVAPSSLPLAGASLPASAKAPKDLEKPTFQILEPGHPPAPPSPPIIDVSSMDIDLRRPKTPPPPTFDSVDSLDQKDTTSGHISSTIIQASGASNASPSGASTSKLPAKVSFKIQKQFNKLEKSTANLSTVSMLNSNPTTSGTPAASLVRQSSPSPSSSSISSQGNVSDALQLSINKLLQVQGGAGEKRPADALPPHIDGEEQPPQQKRSKSAKLDALFGSEDVDLRREILVVKPGVIVVEDDSMDECDVDKPTKKSGSPPKKATLEELRAKLANSARIQNKQGKSDKSRDQAVSQRLKQLAELKVNDDSQEAHDEKVRTILSQAQEMYENNGMNQEQYKDLVQKVVAINENSKIKESRRRDNDLERNAARDAVLRKRIPKLKGNENHSAGSPHTDGSPRYDDQPAAAPEKPSNKRDKTKREMKRRKPTKWGEQVDAGAAQRTAWQLANVNNNNNNNNNKRGGIQLPVQPQAGFRGMPWQQPPAMVLGQSAVPPPPQPPSMISMPPVPPVTMTKAINSLDNPMADVVRSITIDGGSKEIRFYNQVAIIFMDGDQPHEIGFQPGQRVIFIDHNEPLPLCFNDDYKPFHLDGQLHRIRFGFPSRELYIDEHWYEIYFGGPAVSVPIGNKLHIIKAEGPPPNVDIGRVRRDLVVGKINMIADAHTIVPLFLDARQQTFQLGAEQHSLQFVDSFQYALLDGQLQKMEYGGLPKGMMLNGGRSCFIRFGTLPKGVIAGKTHVADMVYIKTDAPAEPPKPPPIIVKPPPVVEQPKPAPVAVAPISLPAAAAALESLNINDLFQKLVSSGIIGGATAAPTLPAADSSAAKEPTASATEPSTASATVAPATLPAGPPAEPIKRIDLRKPESIKTRQAAVVAVLYLGMQCSSCGVRFPPEQTIKYSQHLDWHFRQNRRERDSTRKATSRKWYYDLNDWRQYEEIEDVEEREKNFLEAQGQPGGVEALDELSQQRSLDSPVPTCAAGRDDVDHCCDMCHEKFEQFYNEELEEWHLRSAIRVEDKIYHPLCYEDYKASLNPPTEVKSDQDVDMNNTDDNAMDTLIKVEDDEDEGTPNSSQPIFEDDDDDVIVLPNEEPSVTEIVDDDDEDEYVPGNVTRADMGNESQDKTESNSETKEPKKDQVELGEQQQNESANESDVEIQEPNIPFTDLDTYVEKEMDEATRAALLNVKIKEEPKDEYEEDEDDGFEDVGTVVSLLPLPEDEISIHSSETQTHTIGSSASPATIERPASVTSLSLPANEADELEQGDANTDTDAELNGEKQEATHNLSAVGPALPLASIVNKIKINITKNTSSNSHNSASNTTTTDSQVSAISVIGGSGAAGGASGAGDPVQQVNAIQTISTIPVLCGGNTFVPKIATSTPSNVISSISVIGSTYGASSSSNGSRSTTSTASSAPAASLLAETGSLKSPTPPLATVDPDPEPVVEQKPALRNATLKRTKKVQNGIETSGLCSIM

>Pcf11p

MDHDTEVIVKDFNSILEELTFNSRPIITTLTKLAEENISCAQYFVDAIESRIEKCMPKQKLYAFYALDSICKNVGSPYTIYFSRNLFNLYKRTYLLVDNTTRTKLINMFKLWLNPNDTGLPLFEGSALEKIEQFLIKASALHQKNLQAMLPTPTVPLLLRDIDKLTCLTSERLKNQPNDEKLKMKLLVLSQLKQELKREKLTLNALKQVQMQLRQVFSQDQQVLQERMRYHELQQQQQQQQQQQQQQQQQQQQYHETKDMVGSYTQNSNSAIPLFGNNSDTTNQQNSLSSSLFGNISGVESFQEIEKKKSLNKINNLYASLKAEGLIYTPPKESIVTLYKKLNGHSNYSLDSHEKQLMKNLPKIPLLNDILSDCKAYFATVNIDVLNNPSLQLSEQTLLQENPIVQNNLIHLLYRSKPNKCSVCGKRFGNSESEKLLQNEHLDWHFRINTRIKGSQNTANTGISNSNLNTTTTRKNIQSRNWYLSDSQWAAFKDDEITSTKHKNDYTDPHANKNIDKSALNIHADENDEGSVDNTLGSDRSNELEIRGKYVVVPETSQDMAFKCPICKETVTGVYDEESGEWVWKNTIEVNGKYFHSTCYHETSQNSSKSNSGKVGLDDLKKLVTK

Figure S7.

>Sbi:Sb02g044000

MLAARNPPPPPHDIAARLHHLRDLRRVPLPNRVARIADLHTDEASPVRKLVAEIIGELGSKHMAYLPDMIPCLLHLLNDETPAVVRQAVKTGTNLFSKVLQKLVIQGLFSTGGIDDALKLSWEWLLKFKSVVSHMAFQLSHVEFQCQATGNEGVRLLAVKFVEKTVLMYTPDPNVPSDPPTETTEVMGFNVAWLRAGGHPLLNVGDLAMEASQSLGLLLEQLKYPKVKSLSTSMIIVFITSLSAIAQTRPSFYGRILPVLLSLDPASSIIKLRVPGAFHALKSAFSACLKCTHSSAEPWRERLLEAQNIVNQGDSREDAANAAKNFGDTSNREESWLLMERSTDGSNKRSLAEDMNHMTEDDLHSPKRARQSFDANEHSEGANKRNVESTSVDISSSQPSSIRTGNSEAVYQLIGMFAALAAQGDRAAGSLQILSSSIASDLLAEVVMVNMQHLPISRPEVDQQQLPSTSSGDGLPLSSFFSLLGTLLKRANQIDQDEVSPTKESAVVSSVADDIMTVPASSPVPSSVNLPIEENTNSPTVPLCIETTEAKLVVNNIDLSDEAKDLLQKEAFVRILESDKQEASGGSIARLPLLAHLGVEFPLELDPWEILQKHVLSDYANNEGHELTICILNRLYREAEQDQDFLSSTTATSVYESFLLNIAENLRDMFPASDKSLGKLLCEIPYLPEGVLKLLEGLCSPGSNEKQDKDIQSGDRVTQGLSAVWNLIMLRPSNRDRCLEIALQSSTHHLEEVRMKAIRLVANKLFPMASISKKIEDFANEKLNSVLEVIPSGDSATTEMATPQAHKDVGLENLTAPVADAQTLMSLYFALCTKKHSLLRHVFAIYGNLPQAAKQAVHRQVPILIRTIGSSSDLLGIISDPPADCWDLLMQVLQTLTDAAVPSKDLISSIKILYSKTKDVEVLFAILAHLPKDEVLPVFPSIVNLPIDKFQGAISRILQGSPRTGPSLDPSEILIAIHVIDPDKEGIPLKKVMDACSSCFEQRTIFTQQVLAKALNQLVEQIPLPLLFMRTVMQAIGVFPALVDFVMEIMSRLVSKQIWKYPKLWVGFLKCAILTKPQSYGVLLQLPAPQLENALSKNPTLKAPLAEHAEQPNIRSTLPRSTLVVLGLAEDQPQQPAAAQAQSSQNQAVETSSSAADTATEVTQESSAAS

>Sbi:Sb03g031920

MGEVILTKEVEGLMTEEAEVLVTEGGMEECIVMELYLVLRLVEMDVEMVKALLLIMLTLLMLLLLVRLLRELGRNVTEDLVVLMPNLLSFLKHDDPAVVKQSIASGTTLCAAVLEEMTLQVNKCGKLETWLEDVWAWMKQFKDAVCGVMNEPGPIATKLLALKFIETWILCWTPQANSDQTQPTEGTIFQINGNKYMSNIFLRENGFHIDKYTFHTGKNWRFHTSRLSQFHRSLDPAVLEADAHRALQLLLDIIRTAYAHRGSFLVGTINSLAAVVKIRPIYYDRVLPVLLDFDPGLETAKGAHSASLWYAVRAAFWGFLRSPHQAMIESKDILVRRLRVLSPGEAMEQNIRQVEKMSRNIERASRAIKDESTSWEMPYGDINWKKPAARSSDILTTSDGIAKRARFDMSATSNLPVLGSSDYSDMQADDGCSVGHSSDPSILNNNVSPVEKMIEMIGALLAEGERGAESLGILVSTVEADVMADIVIETMKHLLEASFHLATNNGVQQLNLKYSSGLLTQNLPANSDSALSAAQSTPTADGVSMSPSEAFVMTSVHDAKRDPRRDPRRLDPWRIVSPSALNSIQVKMETNSVHQTDNLSNTLYSNSGKSENYSDYSGDLQKNEDEQNSASQPNQTIAKDKLELLDVATEPEPTSEVEADIRIHSSDVEDMVKPMSSEVISLDESDSMDLEVDPFLPAPEASTPEDTNHDLPVITSHLELSEKGKISINKLAIGQILDDYKKNSLNARFSLLAHLIAQSAADDNIMDLIQRNIIFHSHDQKGYELAMHVLYQLQSISVANSPESSTATSKHYEKFFISLARSLIDSLPASDKSFSKLLCDAPYLPESIFRLLEDLCLSEDNSQQLKDGDGDRVTQGLGTVWSLILVRPPLRHVCLDIALKCAAHSQDEVRGRAVRLVSKRLYDLPYATKKIEQFAIESLVGVADEHTVDTDINLKSLKESTAEIEVGGQGTSVTGSQIPDTEFSENEPFKTSSISPKQSAVSVSEARRRTSLFFALCTKTLQTLTEDSNPSADLVAAVKQLYNTKLKDASILIPLLPSFPKEEVLAIFPRLVDLPLQRFQDALARILQGTAHIGPALTPAEVLIAIHDINPEKDKVALKKVMDACTACFEQRTVFTQQVLEKSLNQLVDRIPIPLLFMRTVIQALDAFPALVDFVMGILSRLVDKQIWKMPKLWVGFLKLSFQTQPRSFDVLLQVYLVMQLKHSCISIIQFY

>Smo:442688

MAVPPEARERAVALLHDAQLHPDAAAKLDSLRELRELALHREPRLLPELLPVLLELRNDRASAIRNFLVERGSQCRVIFLAPRIIEEVGLRQVEYVPVMMPVLLTLLRGEPAFARRAITAGTNLFRHTLEYVLLKGIYTRQVDKFMQDSWISVLQFKEFVYPFAQQHGNDGVRLHAVKFIETVILLFTPDPSGPSQPPLPDRLDGTSKGINVSWIVSGHPLLDATAMGQEASKSLGLLLNQLRQPDVSALPGPVAIVIVNSGFLFGKGMSSLAAIAKRRPSFYGRILPVLLSLGPSCEAIKGGQVASVVTALKNAFVQLLKCTYPAAAPWRDRLTSALRQMNAGDVADLAVERSSRGGERESRESSSRSNREERQDPRNKRPPPQENGNDEESYGKRLRTTPPPPVQQHFHPDASGYANGAGLPPIQQMIQMFEALAAKGEIASIDLLMSRLPPDTLADVVIVNMQNLPPSVPADFMDKPQLLPPPPPPPPIEPVAMDGRRDPRRDPRRMDPRMDPRRLPSPGPPMVMPKQEDLQQPGVPPVFRTVKAEPVSPPVMNSRDPRASLKATSVKMEPTFTHVKSEPDEDMGIRTISTMYGALYPSSDALDDRQEAGTSGAGLSSFLPVSAEALNTLQKHILADYANHMGHELALHVFYELYLMHIAGGDSIEKYENFFVSLSQGLKDSLPPSDKSLSKLLCEIPLFPEGAFALLESLCNPINEPNGDRVTQGLSALWSLILQRPAVRERCLNMALECAVHEVDDARTKAIRLVANKLYPVTFISQKIEEFATKMLLSVVNVGGHNVDSEAQERSTLQEVAPGDQEANAADGGGTKAEAQRCMSLYFALCTKKHALLGELFNVYDRAPKPVKQAIHRQIPVLVRTVGQSSPELLSIISAPPQGCENLLLQVLHVLTEGTTPSPELIKTIKQLHETRIKDAVFLIPILSSLSKEEVLPIFPRLVDLPPDKFQVALARILQGSAHTGPALTPAEVLIALHGIDQQRDAVPLKKVTEACSACLQQRNVFNQDVLAKVLNQLVEQTPLPKLFMRTVIQAVGTFRSLVGFVMDILLRLVNKQIWKTPQLWVGFLKCASETTPHSFRVLLQLPTAQLENALVKHPTLKTPLAAHASQPSVRSSVPRSSLVLLGVIQDDVTAVEAASAPSSSGNAPQQ

>Aly:486936

MASYSRERLEGLASSAKSATELPPKLQRLRYLRRDLRKDESVFPTELLPHLFDLLSDQFGAVRKFVAEILGEVGLKYVELLPEIVPLLIKSLEDETPAVARQVIACGVDLFRSTLERVAVQGLHSSELNDLLESSWTWVIKFKDEICSLAFKQGNSGVKLCAMKFVEALILLYTPHEGIEADFNISILRGGHPVLKIGDLSIEASQKLGLLLDQLRHPAAKSLNSSTIIVLINSLSSVAKKRPAYCGRILPVLLSLDPLSFLKGVHAAAANLALKTVFLSCLKCTHPAAAPDRLISALKEIEGGGRAAKAKDLFYKTNGSIQDKDSVEDTKVSMEENPLCASSDVAESNLSRKRSGSEYNIDLNGDASDGKRARITPSVSEESIDGLNGNDGGSLPRVASTLTGPSDSRGVSDTGPTQQLVGLFGTLVSQGEKAIGSLEILISSISADLLTDVVMANMHNIPPNGSSYADGTDELVMNMCIVGSDAQIKYPPSFVAGVLSLSTAFPPIAALINPHNEDEEVYSVHVDQQMFPAEDARTPPGLLASSFPENEESNTVSLQNVHYIRKRESGIPGLESSAQHDVSGALVTNVLSSTNMEAASKNQNASFSGKLLVDVIPSMSVDKSEEFSPKAVGTGSTSLVLSTATSVASAPQFVLPKISAPVVDLSDEEKDSLQKLVFLRIVEAYKQISMSGGSQLRFSLLAHLGVEFPSELDPWKILQEHVLSDYLNHEGHELTVRVLYRLYGEAEAEQDFFSSTTAASAYESFLLTVAEALRDSFPPSDKSLSKLLGDSPHLPKSVLKLLESFCCPGSSEEVEKDLQYGDRVTQGLSAVWSLILMRPGIRNDCLNIALQSAVHHLEEIRMKAIRLVANKLYSLSFITQQIEEFAKDRLFSVVSCISSERGDAETRIDDCNKKDLDLKSPPNKPQHVISGTGMETPSEATSSTSVTEAQRCLSLYFALCTKVLGIFTILRLMINLAFIIYKNASDPVKQAIHLQIPILVRTMGSSSELLKIIADPPTGSENLLMQVLQTLTEGPTPSSELILTIRKLFDTRIKDVEILFPILPFLPRDNVLRIFPHMVNLPMEKFQVALSRVLQGSSQSGPVLSPSEVLIAIHSIDPARDGIPLKQVTDACNTCFAQRQTFTQQVLAGVLNQLVQQIPLPMLFMRTVLQAIGAFPALSDFILKILSRLVSKQIWKNPKLWVGFLKCTQTTQPQSYKVLLQLPPPQLGNALTKIPALRAPLTAHASQPEIQSSLPRSTLAVLGLVPDSQGTQTSQVQANETQTSQEQEQQQASESQQTSQSQQVYVPLSDSQVDHQEPSQVVASQSQSNPIGPGLPEMSQSQNSPMDTGRSEMSQPQNSPIDTGRPEMSQPQNSPIDMGRSEMSESQSSPIGAGRSEMGQSQSSPIGTGQSEMSQTPQVSDSSVPAPTSHTQTSDPQASSQTQGDGDDDDDEKIDDTATSGNEVTEIEKSKESSEEDEEEE

>Aly:313480

MAAPAATTARAQALSLLAAANNHGDLAVKLSSLRQVKEILLSLEPSLSAEIFPYLAELHLSREILVRKSLIEIIEEVGLRMLDHSYVLVSVLLVLLRDEDPTVAKKSISAGTTFFCNILEEMAMQFHHRGKVDRWCGELWTWMVKFKDIVFATALEPGCVGVKVLALKFMETFILLFTPDASDPEKVSSEGSRQMFNISWLAGGHPILNPATLMSEANRTFGILVDFIQSANRLPGALTISVISWYVSESIPLNHDSLSFSLAVVARKRPVHYNTVLSVLLDFHPNLETVKGCHAASVQYSIRTAFLGFLRCTFSPIIESRDKLLRAFRAMNAADVADQVLRQVDKLVRNNERAARENWSGKNNQVNSHQNSWDLSKKRIMPQGEDDTINGEVAPKRVRHNTNMNLTQHVQTNEFLQGSVSINGISSGNHPSDSELTPVEQMVSMIGALLAEGDRGAASLEILISKLHPDMLADIVITSMKHLPSTPPTLTSSVATPADIVVSSSINTIHSPTPPAQLPFDPILPTGSSFSEVPSLNSSVADPRRDPRRDPRRMDPRRINSPVGPSSLPVGEGKEPAPTQKDISTLLSKPVSVPAVTPGATGSVHSTAVERSQNKMMGSSGIRIINQPDCREDLLTVPNECSYPSKEISSLDVPLSPCRDDEGIRETKYSGSETMYDLDMSSVPDFDQHSPSASVPDFDQDPPAASDITAPEESYRELAPVPSYVELTTEQSKTVGKLAIERIIESNRHVFGFDCNKIRMALIARLIARIDAGNDVATILREHISVDHRDFKGHDLVLHVLYHLHSMAMLDTDDSSPYATIYENFLISVARSFLDALPASDKSFSRLFGEAPHLPDSAIKLLDELCSTRHDPVGREVCDSERVTQGLGAVWSLILVRPNERKACLAIALKCSVHSEEDVRAKAIRLVRKLVPDSNMVTNKLYHLTYIAEHVEQFATDMLLTAVNSETDLSQTGSITEGIKTEAKIQITSTSDSLWSGNSDIHSQQDLQTSRDVSVISISEAQRLISLFFALCKKKPSLLRLVFEVYGRAPKMVNQDVSILIPLLSSLTKDEVLPIFPPLLNLPPEKFQLALAHILQGSAHTGPALTPAEVLIAIHDIVPEKDGPPLKKITDACSACFEQRTVFTQQVLAKALGQMVDRTPLPLLFMRTVIQAIDAFPTLVDFVMEILSKLVRKQIWRLPKLWPGFLKCVSQTKPHSFPVLLELPMPQLESIMKKFPDLRPSLTAYANQPTIRASLPNSALSVLGLDNGQDSRSQMHPSDATSSIHGAALT

>Gma:Glyma01g30320

MVGKTMCMAATSREKLTSLVNAAKLAIDIPSKLESLRQLRHELPPEDPVLLTEFLPSLFLFHSDRFGPVRKFLTEMLGEIGLKNTEFLSDIVPVLIDLLDDDTPAVVRQALLCGIDLFRATLEKIAVQGLYSSDLDGALESAWAWMLKFKDKVYSIAFQHGSGGAKLLALKFVEAVICLYTHDPNGSSEPTSHQGRPVEFNISWLGRGHPVLNIGDLSIEASHRLGLLLDLLRFPTVKSLGNSVIIVLIKSLSAIAIDRPAFYGRILPVLLSLEPSSSVVNGVSVSATHFALKNAFLTCSKCTHPSAAPIKAIMAALCIAALCHIMGVASQAQHSGKIYRCVVDIESGEWVGDRRWCLGFSIRKEKRNQVMCGFRYRPCGGCVQNLHGFLKNSCAYGLVVPCETSKLLLLLFFTLFSLNMERSFGRGLEGIAVRRKGRSVAPIIGYNLNNILFSNFLHNGIKQLKKLFTCLHKIRAINRLQKHGTSLRVNQKINDASLFPISPCIGHNSTTILDPLLPMAFHQIFSIFNFLLQEWNSDVMIARNGQMWPILTNGCNHVLKAFQFNEEEEPATNSGDSVQNTLARKRSGSQIGGDLSEDEETPGKRVRTTIVALEEPKKELDVCTTAYSQDEAPSKGVVDNGPVRQLVATFGALIAQGEKAVGHLEILISSISADLLAEVVMANMQNLPTYYPNAEGNDEQLQDISMIGSDDKAKYPASFVAAVMSLSKSSFEQHFDVHKYCRSHKWRKKLLKLLQILVLIPGVENGCTTVPPDPDIHDVGNSESGIPGLDSFGRSDAVSQTFAPSLLVSTEICQEDGSQEQDTSLDQRSPLNLAPSISTDRSEELSPKAAVRDANSLVSSTATSVVPPRLVLPKMIAPVVDLEDEQKDRLQQSCFMRIIDAYKQIAVAGGSNVRFSILAYLGVEFPLDLDPWKLLQKHILIDYTGHEGHELTLRVLYRLFGEAEEEPDFFSSTTAASVYEKFLLTVAEALRDSFPPSDKSLSKLLGESPYLPKSVLKILENMCSPGNGDKGEKELHSLNADRVTQGLSTVWSLILLRPPIRDTCLQIALQSAVHHLEEVRMKAIRLVANKLYPLSSISKQIEDFSKEMLFSVMSGDATEATDVEGSFADSQKGPDVEKVPNEQSSLSGSTKDVPSDNRQSCTSESVSPDSVSEAQRCMSLYFALCTKKHSLFRQIFVIYRSTSKAVKQAVRCQIPILVRTMGSSSDLLEIISDPPNGSENLLMQVLQTLTDGTVPSKDLICTVKRLHDSKLKDAEVLIPILPFLSHDEVMPIFPHIVNLPLEKFQAALGRILQGSSQSGPVLTPAEVLIAIHGIDPEKDGIPLKKVTDACNACFEQWQTFTQEVLARVLNQLVEQIPPPLLFMRTVLQAIGAFPTLVDFIMGILSRLVMKQIWKYPKLWVGFLKCVQLTKPQSFGILLQLPPAQLENTLNRIAALKAPLIAHASQPDIQSKLPRAMLVVLGLASDSQVLSQAQTTQTQTSQTQTSQTQTTQTQTSQTQTSQTQTGETSNSDKDTATEKSKESSTAS

>Gma:Glyma03g07790

MAATSREKLASLVNAAKLAIDIPSKLESLRQLRHELPPEDPVLLTEFLPSLFLFHSDRFGPVRKFLTEMLGEIGLKNTEFLSNIVPVLIDLLDDDTPAVVRQVLLCGTDLFRATLEKIVVQGLYSSDLDGALESAWAWMLKFKDKVYSIAFQHGSGGAKLLALKFVEAVIRLYTPDPNGSSEPTSHQGRPVEFNILWLRRGHPVLNIGDLKIEASHRLGLLLDQLRFPTVKSLSNSVIIVLIKSLSAIAFDRPAFYGRILPVLLSLEPSSSVVNGVCVSATHFALKNAFVTCSKCTHPSAAPWRDRLAEALKEMQSEGKADRVFHLISASNGTIEREKDDQPVIKEEEPATNSGDSVQNNLARKRSGSQIGGDLAEDEETPGKRVRTTVVALEEPKELDECTTTYSQDETPTVPTSSKGDVDNGPVRQLVATFGALIAQGERAVGHLEILISSISADLLAEVVMANMQNLPPNYPNAEGNDEQLQDISMIGSDDKAKYPPSFVAAVMSLSSTFPPIASLLDAHQSVSNEKSQVEEEISATAANSGAVDSGMNIESENIPSPIDFPSSDASIPGVENGCTTMPPDIHDVGNSESGIPGLDSFGRSDSVSQTSAPSLLVSTETCLEDGSQEQVTSLDQRSPLNVAPSISTDRSEELSPKAAVRDVNSLVSSTATSVVPPRLVLPKMIAPVVDLEDEQKDHLQKSCFMRIIDAYKQIAVAGGTNIRFSILAYLGVEFPLELDPWKLLQKHILIDYISHEGHELTLRVLYRLFGEAEEEPDFFSSTTAASVYENFLLTVAEALRDSFPPSDKSLSKLLGESPYLPKSVLKILENMCSPGNGDKGEKELHSLNADRVTQGLSTVWSLILLRPPIRDTCLQIALQSAVHHLEEVRMKAIRLVANKLYPLSSISKQIEDFAKEMLFSVMSGDASEATDIEGSIADSEKGPDVEKVPNEQSSLSGSTKDVTSDNRQSCTSESVSPDSVSEAQRCMSLYFALCTKKHSLFRQIFVIYRSTSKAVKQAVHRQIPILVRTMGSSSDLLEIISDPPNGSENLLMQVLQTLTDGTIPSKDLICTVKRLHDSKLKDAEFLIPILPFLSNDEVMPIFSHIVNLPLEKFQAALGRILQGSSQSGPVLTPAEVLIAIHGIDPEKDGIALKKVTDACNACFEQRQTFTQEVLARVLNQLVEQIPPPLLFMRTVLQAIGAFPTLVDFIMGILSRLVTKQIWKYPKLWVGFLKCVQLTKPQSFGILLQLPPAQLENALNRIAALKAPLIAHASQPDIQSKLPRAVLVVLGLASDSQVSSQAQTSQTQTSQTQTSQTQTTQTQTSQTQTGETSSSDKDTATEKSKESSTAS

>Gma:Glyma04g09480

MDRKRPVPLDNEQLANGHDTISKRIRSGSDSHSTLPAQINDSRQDLSSVNGVSANVPVLDSELTAVEQMIAVIGALLAEGERGAESLEILISKIHPDLLADIVITNMKHLPKTPPPLARIANLPVTRQLSSQLHSLHLRPLSLLLLPLHCHLTPLTSVTSLLILNDPRRLDPRRVVVTPGGATVSIADDTGATKEFDEPVSSIKPVSLPVMTADDNTLSDLTLPPYIELSEEQGSKVKNMAVRRIIDSYKHLHGTDCQQFCMPLLARLVAQIFFCYQMIIMVPVNFHYIFEADHLIKFDFCGSFCRYIIDYKKGWHSAQGSPIVGFGERVDLCITPVSGERGHELVLHVLYHLHSLMILDSVGNASSSAVLYEKFLLGLAKTLLDSFPASDKSFSRLLGEVPLLPESSLKILNDLCYSDVIGHDGKIIRDIERVTQGLGAIWSLILGRPQNRQACLGIALKCAVHPQDEIRAKAIRLVTNKLFQLSYISGDVEKFATKMLLSAVDHEVSDTVLIYWIELFDSDLVATVSQNNLVSHKFYLTLVESHEISCTSQVSESTISENDTAIFAKPSIQSVPSILFSEAQRLISLFFALCTKKPSLLQIVFNVYGQAPKIVKQAFHRHVPVVVRALGQSYSELLHIISDPPQGSENLLTLVLQILTQDTTPSSDLISTVKHLYETKFRDVTILVPLLSSLSKQEVLPIFPRLVDLPLEKFQRALAHILQGSAHTGPALTPVEVLVAIHGIVPEKDGLALKKAFNVFLKNVLVQIIKLFWYIVFMALFMITDACSACFEQRTVFTQQVLAKALNQMVDQTPLPLLFMRTVIQAIDAFPAMVDFVMEILSKLVSRQVWRMPKLWVGFLKCVYQTQPRSFHVLLQLPPQQLESALNRHANLRGPLASYASQPTVKSSLSRSTLAVLGLANETHEQHLSSSLHSSDTSSSVHGATLT

>Gma:Glyma06g09580

VPLDNEQLANGHDTISKRIRSGSGSDSHSTLPTQINDSGQDVNSVNGVSANVPVLDSELTAVEQMIAVIGALLAEGERGAESLEILISKIHPDLLADIVITNMKHLPNTPPPLARIGNLPVTRQLSSQVSQSQSLSGTAQASFPSTSTTVTATATTSLPSDTSSFSNQPADSKRDPRRDPRRLDPRRVVPVSLPVGTADDNTPSDLTVKIINDDIVSEEDLERLGDIHQITEADTSLDLPLSSTYLRDEDPSTVKLPDDTETIGTDSSIFEFDQFSLDVQVESTLEDTCLELPQLPPYIELSKEQESKVKNMAVMRIIDSYKHLHGTDYNVAYCLDTTFVWILESILMLIVADYDSIYRCVSKNCFCSLISFGHELVLHVLYHLHSLMIVDSVGNASSSAVLYEKFLLGVAKTLLDSFPASDKSFSRLLGEVPLLPESSLKILNDLCYSDVIGHDGKIIRDIERVTQGLGAIWSLILGRPQNRQACLGIALKCAVHPQDDIRAKAIRLVTNKLFQLNYISGDVEKFATKMLLSAVEHEVSDTGLLQSGHTEQRAEAELPCPKIIFVPSISFSEAQRLISLFFALCTKKSGLLQIVFSVYGQAPKTVKQAFHRHIPIVVRALGQSYSELLRIISDPPQGSENLLTLVLQILTQDTTPSSDLISTVKRLYETKFKDVTILVPLLSSLSKQEVLPIFPRLVDLPLEKFQRALAHILQGSAHTGPALTPVEVLVAIHGIVPEKDGLALKKAYLITDACSACFEQRTVFTQQVLAKALNQMVDQTPLPLLFMRTVIQAIDAFPALVDFVMEILSKLVSRQVWRMPKLWVGFLKCVYQTQPRSFHVLLQLPPQQLESALNRHANLRGPLASYASQPT

>:GSVIVT01009244001

MFVIQSHCSESNVEHVQMIMRVLYIGFSLLLSIYIFFWEILCYIFNFWGWFVSSNAMGFLAAIARKRPHHYNTVLSALLDFDSSIEMVKGHSASVQYSLRTAFLGFLRCTCPTIMESRDRLLRALRSMNAGDAADQVIRQVDKMMKNNERASRDARLGRDDPPSSQLSVPGDLFRKRSMHQDNEEPTNGHGMTSKRIRYGHNMHSASHVQMSDSGQDSTAAPTITMQSSVLPAQVPFSTAAATSMAHSEMSTVINLPPDSKRDPRRDPRRLDPRRVGVPVGLQSVHMVEDTGAIQAEFDGSISLSKPPSLPVVTSVENTSTSLVSKTEGDDKILKNALISETDQPISREELLDGAKEVDHIPEIGATSDAALSPARTIDEDSAAPESLDIAVADGADTSPLIETDQHSPARSNTYVSEETSIDLPLPPPYVELTEDQKIRLKKLALERIIDSYVYSRETDCSHTRMALLARLVAQIDGDEDVVVMLQKHVLLDYQGQKGHELVLHILYHLHALMISDSVEHSSFAAVVYEKFLLAVVKSLLEKLPASDKSFSKLLGEVPLLPDSALKLLDDLCSSDVTDQHGKVLRDRERVTQGLGAVWSLILGRPLNRQACLNIALKCAVHSQDDIRTKAIRLVANKLYLLSYISENIQQYATDMLLSAVNQHISDPELSQSGSSDQRLEAETGSLETSVSGSQISEPGTSENDPMKGSQSVQNISTVEFHQAQRLISLFFALCTKKPNLLQLVFNIYGRAPKAVKQAIHRHIPIIIGALGPLYPELLSIISDPPEGSENLLTQVLKILTEEKTPTPHLIAIVKHLYETKLKDATILIPMLSLLSRNEVLPIFPRLIDLPLDKFQDALANILQGSAHTGPALTPAEVLVAIHDISPEKDGIALKKITEACSACFEQRTVFTPQVLAKALNQMVDHTPLPLLFMRTVIQAIDAYPTLVDFVMEILSKLVSKQVWRMPKLWVGFLKCVSQTQPHSFRVLLQLPAPQLESALNKHANLRGPLSAYASQPSIKSSLPRSILIVLGLVNEPHMQQSHPPSSLHSSDTSSSVHGATLT

>:GSVIVT01011157001

MVGMMTASLINSAKLALDVPSKLEHLRQLKEDLLHEGPVLLSQFLPRILDLHTDRLSPVRKFIAQMIGEIGSKHLDLLPEIIPVLISLLKDGTPAVARQAITCAIDLFRCTLEKVAIQGLYSSELDVSLESSWEWMLKFKDKIYSIAFQPGSDGRRLLALKFVESVILLYTPDPNGSSDPPSNQPSEGKFVEFNISWLRGGHPVLNVGDLSIQASQSLGLLLDQLRFPTVKSISNSMIIVLINSLSVIARKRPSFYGRILPVLLGLDPSSSVIEGVHISGAHHALRNAFLSCLKCTHPGAAPWRDRLVDALNEMKVGGLAEQALREVCKINGSVLEGKDDSSIVKEEKPSVKSCDAVHVTLGRKRSGVHDIGDLVEDDDVSGKRVRTASTVAEEPSKESSRDLTSVQNVSPIGLKSSRGDEDTGPVQQLVAMFGALVAQGEKAVGSLGILISSISTDLLAEVVMANMRHIPPERPKDEGEEESLLNMGSNASTVGSDTQAKRLPPFLARFPQIVALLDAQQSASNDIVKSQGEEEHHVATVADSDLACGDMDCGTEQGMDSAGVPISSNVLPSAIENFSATSYEIHDVGNLESIPGLDSTAHDDRFVETLAASSLASADLEEGSQEQVTSLGRRSQLDLLPSMSTDRSEELSPKSSLTDANSIISSTETSAGLSSQFVLPKLLAPVIDLTDEQKDLIQKLAYARIVDAYKQIAVAGGSHVRFSLLAYLGVQFPLELDPWEDLKQHIMSDYLNHEGHELTLRALYRLYGEAEEERDFFSSTNATSVYDMFLLTVAETLRDSFPASDKSLSRLLAEVPYLPKSVFKLLDCLCSPGNSSKDEKELLSGDRVTQGLSAVWNLILLRPPIRDACLKIALQSAVHHSEEVRMKAIRLVANKLYPLSSVAQQIEDFANEMLLSVINGAHATDRTETEGSSTELQKDSNLEKSSDEHSSGSAIAKEIASDTQQSCTSQTISSSSISEAQRCMSLYFALCTKKHSLFRQIFVIYKSTSKAVKQAVHRHIPILVRTIGSSPELLEIISDPPPGSKNLLTQVLRTLTDGAVPSPELIFTIRKLYDSKVKDIEILIPILSFLPKDEVFLIFPHLVNLPLEKFQAILVHTLQGSSHSGPVLTPAEVLIAIHGIDPDRDGIPLKKVTDACNTCFEQRQIFTQQVLAKVLNQLVEQIPLPLLFMRTVLQAIGAFPALVEFIMEILSRLVSKQIWKYPKLWVGFLKCALLTKPQSFSVLLQLPPAQLENALNRTAALKAPLVAHAIQPNIRSSLPKSVLVVLGITPDSQTSSQTQTTQAQIAPPQTGDTTNLDKEVVTEKAKESSSAS

>Ppa:Pp1s84_220V6

MAGIREQAVALLNEVKMTGEASAKVDLLKTLMELVLYRDPLSLLPEFVPYLMEFQTEPGSPIRKYLAEMIEGIGMKHVDFVPVIVPVLLSLLQDATPAVARRAITSGSNIFRSVLEQVALQGVYSGQVERRLVDCWNWMTCFKDAVFSLAFQHGNDGVRLLALKFVETTILLFTPDPNGLLPNQQSSDGTSRGFNMTWIAGGHPVLDATVLGQEASKNLGLLLNQLRTPEASTLPCLVAIVIINSLAGIAKKRPSMFGRILPVLLALAPNCEPIQGSQIASVIHELKSAFLNLLKSNQPGALPWRDRLVAALRSMNVGDIADQTIRHLERAVRVAERNRTAKDTRVSTKERISPFVSTPQVSTIQVIAPAPVDMSLRKRSLPQENGNVQSIDFNEVSGKRSRHDVAPITTQGEATPVIPNGTGNVMGNTAAYGDHSVAPLISAFATLLAQGERGAASVQILIESLTPDMLAEIVIANMVHLPSTPPFTPEVNFGANWGLGPIPPSGPPEIPVNPVTSLPIESAPALQPYSSTADLSQDLRKDPRRLDPRPAVPSDGVTVLLTPKLEVEVNAVPPPVANAQTGNSNLENTLNVSTNSRDPRAMFHVNANHRSIGQSHIQVKQELKQEPLQEPKQEPIELENRVVSDPSLPSSPPPQSGVSTVPVPSPVITLLPLSAEQQAALSTASLVRIMENHKTIAAAGGADLRIALLARLVAQSYEDTEVLKVLQKHVLADYQAHKGHELVLHVLYQLFAEHACSDVEQPMSTSLAYEQFLLSLAQGLRDKISAGDRSLSRLLGEAPVVPMSTLKLVEELCNPPSGAESSKGEHVTAGLTALWSLILQRPPTRDTCLKMALKCTVHESDDTRAKAIRLVANKLYPLKFVAQNIEDFATASLLSVVDHQSGDTAERDILEGDNTVKTDLMNEDQRVANGVAPMDVAGDEGAQQPTTAAKNTEVLSLSEAQRCMSLFFALCTKKHALLRQLFQVYGRSSKVVKQAVHRHIPILFRTIGSSSQELLQLIADPPAGSENLLLQVLHSLTDGTEPSEELIATVKKLYDVKFQDATYLIPVLSSLSKDEVTPIFPRLVDLSNEKFQAALARILQGSAHTGPALTPAEVLISLHGIDPHRDSVPLKKVMDACATCLQQRTVFTQQVLAKVLNQLVEQTPLPLLFMRTVIQAVHSFPSLESFVMEILSRLVSKQIWKLPKLWTGFLKCAHQTKPKSFHVLLQLPTAQLEDALKGFPSLKEPLAAHADQPSVRPTVPRSSLVLLGLCQDTPAASNSTSAGQESNTEVSKQ

>Ptr:POPTR_0002s10820

DVHLLPSQFSISWLPYKLLRVLTLLNPANVNLSTFSYGGCLILISGILFLSYLNKISFFLQCLAAVARKRPLHYETILSALLDFDPKVEKGCHAASIQYSLRTAFLGFLRCTYPTILESRDKLLRALRAMNAGDAAEQAIRQVDKMIKNKERTSREVRFSRDDQPTSQLPVSGDQLRKRSVPMDNEEQANGHEMAQKRSRYGPNILSTTPIQINESGPDSVFDNGVSANVHLSDSDLTPAEQMIAMIGALLAEGERGAESLELLISNIHPDLLADIVITNMKHLPKSSPPLTRLGSLPVTLQNCSSSSPAQAVAPSAPVSSAQGPIPVVTAGNLSLSDAPIVNNFPVDSKRDPRRDPRRLDPRRTATSVGVPSVAIVDDHGGMQPEMDSSVSLSKASPLPVVTSVENPPEPYISNSKIEDKSLEGLLVSKTDQVSMGEEVICRPEEIVPILEAKASSDQAFSPPHTSEEGDVVLKLSDFEVASGADTLSVMEPEQLSPDVSNISVPEEICQVDLPQLPPYVELTEEQQKTVRLLAVERIIESYKHLSGTECSQTRMALLARLVAQIDADDDVVVMLQKHVLVDYRQHKGQELVLHFLYHLHSLTILDSVGSASYAAVLYEKFLLVVARSLLDAFPASDKSFSKLLGEVPFLPESAFKLLDDLCHCDIFDSHGKEVRDGERVTQGLGAVWGLILGRPNNRQAFLDIALKCAVHSQDDIRSKAIRLVANKLYQLNYISQNIEQFATNMLLSVVEQHASDIKPSQSVSTDQREGEPLFVLYNFNLPFGTQAVHRHIPVLIRALGSSYSELLRIISDPPEGCENLLMLVLQILTQETTPSANLITTVKHLYETKLEDATILIPILSSLSKNEVLPIFPRLVGLPIEKFQMALAHILQGSAHTGPALTPAEVLVAIHDINPNKDGLPLKKITDACSACFEQRTVFTQQVLAKALNQMVDQTPLPLLFMRTVIQAIDAFPSLVNLHVLSNLFATLPFKLYHPVCNQIVC

>Ptr:POPTR_0016s12390

MVAMTKSSSRERLASLINSAKSASDIPSKLQTLRQLNQILQQQENANSLSEFLPRIFEFQSDQHSPVRKFATEMIGEIGLKHLEFVPEIVPVLMLVLEDLVPAVARQAITCGISLFRATLEKLAIQGLYTSELDDLLKSSWSSMLEFKEKIYSIAFQLGSGGVRLLALKFVEEVILLYTPDPYGTSEPPSHEGNDTRSLTFTSCPGCITRQKILICYCTSISMDSIWLCRVVTYFAGSSVEFNISWLRGGHPVLNVGDLSIEASRKLSLLLDQLRMPTVKSISNLMIIVLVNSLATIAKKRPPCYGRILPVLLGLDPSNSVIEGMHGYGAHHALKNAFLTCLKCNHLGAAPWRDRLVGVLKEMKAGELAEEALQVLRSNGSVEEAKEDFLVAQEEKLLIKSSDGIPNNSARKRSGPEDSIDLADLAKDDDVSGKRVKSSPSVSEESSKELDHRANKKDDDNGPVQQLVAMFGALVAQGEKAVGSLEILISSISADLLAEVVMANMRYLPTGHPQAEGDDESLLNMTIVGSDTRAKYPSSFLTNVLSLSSSFPPIAAQLNAGHSVSKDIPTTDEEELQTTTDEEELQTTKDEEELHVAAADVADVYTGKAHSAEDELMPAGLPASSNVDLSGMQMDGLAISSNIHDFENLDSEIPGLDSSARNDVFSETMGASSLVSTDIEDASQEQGTSLGTRSNQEVLPSISNDRSEELSPKAAATDSNSLISSTATSVCLHQPLVLPKMSAPVVNLVDEQKDQLHNLAFIRIIEAYKQIAVAGSSQFRLSLLASLGVEFPSELDPWELLKKHILSDYVVHEHLTILAGCLQGHELTLHVLYRLFGEVEEEHDFLSSTTAASVYEMFLLTVAEMLRDSFPPSDKSLSRLLGEAPYLPNSIFSLLESLCSPGNIDKAEELQSGDRVTQGLSTVWSLILLRPPIRESCLKIALQSAVHHLEEVRMKALRLVANKLYPLSSIAQQIEDFAKEKLLSVVNSDATESMDAEGSFTESQKDSILEKPSNEHQSMSAISKDISSETHQSCTSESVSSLSISEAQRCLSLYFALCTKKHSLFRQIFIVYKSASKAVKQAVNRHIPILVRTMGSSSDLLEIISDPPIGSENLLMQVLQTLTEGAVPSPELLFTIRKLYDSKIKDAEILIPILPFLPRDEILLIFPHLVNLPLDKFQIALARTLQGSSHSGTMLSPAEVLIAIHGIDPDRDGIPLKKVTDACNACFEQRQIFTQQVLAKVLNQLVEQIPLPLLFMRTVLQAIGAFPALVEFIMEILSRLVSKQIWKYPKLWVGFLKCALLTKPQSFNVLLQLPPPQLENALNRTAALKAPLVAYASQPNIKSSLPRSVLVVLGIAPDPQTSSQAQTSLAQTGDTNNSDKDVTVENSKTGETSNSVKEVLTEKSKESSVAS

>Ath:AT1G27595

MPQGEDDTINGEVAPKRVRHNTNMHLTQQVQTNESLQGPVSINGISSGNHLSDSELTPVEQMVSMIGALLAEGDRGAASLEILISKLHPDMLADIVITSMKHLPSTPPTLASSVATPADIVVSSSTNTVHSPTPPAQLPFDPILPAGSSFSEVPSLSSSVADPRRDPRRDPRRMDPRRLNSSVGPTSLPVGEGKESVPVQKDISTLLSKPVSVSAVTPGATGSVHSTAVELSQNKMMGSSGIRIIDPPECREDLLTVPNECSYPSKEISSLDVPLSPCRDDEGIRETKYSVPDLDMLSVPDFDQHSPSASVPDFDQDPPAASDITAPEESYRELDPVPSYVELTTEQSKTVGKLAIERIIESNRHVFGFDCNKIRMALIARLIARIDAGSDVATILRELISVDHREFKGHDLVLHVLYHLHSMAILDTDESSFYATVYENFLISVARSFLDALPASDKSFSRLFGEAPHLPDSAINLLDELCSTRHDPVGKEVCDSERVTQGLGAVWSLILVRPNERKACLAIALKCSVHSEEEVRAKAIRLVTNKLYHLTYIAEHVEQFATDMLLTAVNSETNLSQTASTAEGIKMEAKSQITLTTESLGSGNSDIPSQQDLQTSREVSVISISEAQRLISLFFALCKKKPSLLRLVFEVYGRAPKMVNQAFHRHIPILIRELGSSYTELLQIISDPPKGSENLLTYVLQILTQELAPSLDLIATVKHLYETKLKDVSILIPLLSSLTKDEVLPIFPPLLNLPPEKFQLALAHILQGSAHTGPALTPAEVLIAIHDIVPEKDGPPLKKITDACSACFEQRTVFTQQVLAKALGQMVDRTPLPLLFMRTVIQAIDAFPTLVDFVMEILSKLVRKQIWRLPKLWPGFLKCVSQTKPHSFPVLLELPVPQLESIMKKFPDLRPSLTAYANQPTIRSSLPNSALSVLGLDNGQDSRSQMHPSDATSSIHGAALT

>Ath:AT1G27570

MAAPADATTKGQALALLAAAKNHGDLAVKLSSLKEVKEILLSLEPSLSAEIFPYLRELCLSPEVLVRRSLIEIIEEVGLRMLEHSYVLVSVLIHLVGDNDPTVAEKSISTGTTFFRSILEKMETQFHHRGKVDRWCVNLWTLMLMFKDAVFNIALDLEPGRVVGVKVLALKFMETFILLITPHASDPEKVSTSSEGSRQMINISSLAAGLPMLNLTGLMSEVNQTLVRLGSFLQAPTLIQDALPIAVIDCSLSFSLAVVARKRPVHYDTVLSVLGFLKCTSSPIVESRDLLFRAFPAMDPADISDQVVREVDELFRVNEHAANENRSSQILEVFPTLSSLPMQLRDHLQQMIHWLDEAIIGDMLPQYERRNDMRQVLDRDRRQRCLFKRFSDEDRARNEAIAYLLDHPEDGHRSQSEQIYGFSRVRRAVWVRCRIKNQMKMGVLIEFLESSVTVQSLGTVYSELPDDVGAEEIHKIVVLDIRFGNIDRNLGNLLVQAEPRNGSAAHLVPIDHELSFFNDAHPYITCGACWIKWLEQIDKDFSSQLVNYVAALDPDRDLEFLRHCGWEPNQRYIENFTVFATFLKKAVSQGLTALQIGLLASYKWEEDLDYNLHCIVASVQREDNNFVESVGTRIEQRLREFHENLHGNA

>Ath:AT5G01400

MASYSRARLKDLANSAKSATELPPKLQRLRYMRRDLQKDDSVFPTELLPHLFDLLSDQFGAVRKFVAEILGEIGLKYVELIPEIVPLLIKSLEDETPAVARQVIACGADLFRSTLERVAVQGLHSSELNDLLESSWTWLIKFKDEICSVAFKQGNSGVKLCAMKFVEALILLYTPHEGIEADFNISILRGGHPVLKIGDLSIEASQKLGLLLDQLRHPAAKSLNSSTIIVLINSLSSVAKKRPAYCGRILPVLLSLDPLSFLKGVYAAATNLALKTVFLSCLKCTHPAAAPDRLTSALKEIEGGGQAAKAKDLFYKTNGSIQDKDSVEDTKVSVEENPLCASSDVAESNLSRKRSGSEYNIDLNGDASDGKRARITPSVSEESTDGLNGNDGVSLPRVASTSTGPSDSRGVSDSGPAQQLVGLFGTLVSQGEKAIGSLEILISSISADLLTDVVMANMHNIPPNCSSYADGTDELVMNMCIVGSDAQIKYPPSFVAGVLSLSTAFPPIAALINPHNEDEEVYSVHVDQQMFPAEDARTPPGLLATCDTSFPENEESNTVSPQNVHYIGNRESGIPGLESSAQHDGSGALVTNVLSSTNVEAASKNQNASFSGKLLVDVIPSMSVDKLEEFSPKAVGTVASASQFVLPKISAPVVDLSDEEKDSLQKLVFLRIVEAYKQISMSGGSQLRFSLLAHLGVEFPSELDPWKILQEHVLSDYLNHEGHELTVRVLYRLYGEAEAEQDFFSSTTAASAYESFLLTVAEALRDSFPPSDKSLSKLLGDSPHLPKSVLMLLESFCCPGSGEVEKDLQHGDRVTQGLSAVWSLILMRPGIRNDCLNIALQSAVHHLEEIRMKAIRLVANKLYSLSFITEQIEEFAKDRLFSVVSDDCDKMDLDLKSPPNKPQHSISGMSMETPSEATSSSTSVTEAQRCLSLYFALCTKVLRIFTILRLMTNLVFNIYKNASDPVKQAIHLQIPILVRTMGSSSELLKIIADPPSGSDNLLIQVLQTLTEGPTPSSELILTIRKLFDTRIKDVEILFPILPFLPRDDVLRIFPHMVNLPMEKFQVALSRVLQGSSQSGPVLSPSEALIAIHSIDPARDGIPLKQVTDACNTCFAQRQTFTQQVLAGVLNQLVQQIPLPMLFMRTVLQAIGAFPALSDFILEILSRLVSKQIWKYPKLWVGFLKCTQTTQPQSYKVLLQLPPLQLGNALTKIPALRAPLTAHASQPEIQSSLPRSTLAVLGLVPDSQGTQTSQVQANETQTSQEQQQQQASEPQQTSQSQQVSVPLSHSQVDHQEPSQVVASQSQSSPIGTVQSAMSQSQNSPIDTGRSEMSQSQNSPIDTGRSEMSQSQNSPIDTGRSEMSQSQNSPIDTGRSEMSESQSSPIGQSQSSPIGTGQSDMSQTPQVSDSSAPEPTSHTRTSDPQASSQTLRDDDEKIDDTATSENEVTEIEKSKESSEEEEEEEEEEE

>Cre:Cre02.g099200.t1.2

MAALTVKLPPLIEQLRGAVGEDAKIAALAALRNALDSSQTGEAPCPNDFVATWLPGLLQQMQGDASSYVRRELAAFTGQLAVNHTGTAALAAACACLGALLRDGVPGVVKEAVLAFACVLRGALALASLKPNQTDVRDAWDGVRRLQGEVVATSQGHAANCVRIAATKLAEHVVLLFTHDAAPTVPGVPETLVNKLRLPDAARLAGEAEGLLRWLLEPLRPAAALDQPPVRLIAHVSAAWSVAIARPNLMGKLLPVLMALAKEGGFLASSAPGAAGRSSGDVSVGNALRSGLAAVLKSRVPSSVPWRARLVAALESLGAGDTASHMMKYIERQEWKERRATRDKRPAEDALSGHDAKRAAAEAAAAAEEAARVSQAPPAAPAPGGGYGSQQGYAPGGVVPSAGPGGYAYIMPAPANGAAAGPGYATYPPYQPPGASQPVAVQPMPQQLQAAAAQQQQQQQQQQQQQQALSEATQQAMQQAMQQAAAAQAAAIITAAAQVAAGLPPVTAPPSEKSDLDQILAYADAALRSRDDALVESLLGSLVAGQPALLADLVLAHLPLLPPQLPPQFWPPPMPMPPPMPAPAPAPLVAALQAQPVAQQQPQLPAPPPAAAAAAAAAVAAAQAVAVAAAAVAAGGPPAAPQAAVPIGAAGYPPGVGALMPPPPQVVMVGPPGLQQPGLPMAAPAVPVAGGPAGFPARMTPPPPPPPPMVAVVPVGAAAAVAAAPAGPAPGRQPEGPTVVMPGRRPRGPLPPLQGPARPPLAQPQFALSADGAGAPGRRGAAAAAGAAAAPPKPPPVRPATLLPAAAAALRQGALRRLMALPPAAPPGRRLRQVVLARFGAHVLPSDPTAEHLLEYSLRHYHAGGSELALAWLNSLFVAVCPLPQRVVAAAAAAAAAGAGASAGGGGGAGGSGQGRTGGLHGMEEEEGKDGGQEEGKEDEKEEGKHWVAGQGLATGQAAADDGDAIMAEAGCGGAGDGAGTAGEGVGEEDKKAEPANSTPEPKADADADMSEAAAAAGTAAVLQGEGVPAAADADADADAMAKAYDAYADADDHGLMGGGASGQHNLAVGDGLPGPGPGEAAEGGPGQAAGAAGSGEASGQTSAGGQVGAGQEAQAEAPDLSGTPYETVLLALLERARNHAVMWHAHQLRRYSRSLRDTATILQQQRAALLPLQPPAARLAAGLEATLAAARLLQTNVAWAAAQGAWLQAEASGLDLGAAGGGGVEGEELQAVPELADEVVAEVEGVQASVQESMMQLRQWQQELGHPLPPPPPPPAQPPRPLVGLEVKELLRAAPSLPLGGVLGFLRELLGGGGKWATLALSTAWDAIEGRPPARAAMLDLVLEAAEDPHEEVRSAAVRLLTSKLYPRPNLRPTILTTAALRLTSLMPAPPPAAAPAPAPAPADTITTTTATATTNTAEAGAGGAEGAAAAAEPPQPPLPPPPPVEPCSVSEATRRVALYMALVAKQPDLLPGLVAAYAGGGPNLRAAVGGHAGSLAAALGPSHPALLAQLRAPVPGSEELLLTMLHALTDKDLPPGRLVDSCKAWYAASSDPRVLVPVAFTLSRREVVGLLPVMLRRLAPAVLKRLYRSLASKHGEVEPLFSASELLVALHRDLDPVRDELPLKSLMASVDLALHSPDIFPQPVMLAAIRAMEGLSPLPRMFMRTVIQALKAAPRLRTDVVQLLERLVAKQIWMDRDQWRGFLLCADNMRSDAYGPLLQLPEAVLEVTMLGAAASDAARAQAGRMGLPPGCQLPLGSGARLAAYVLHPAPNQPQMANVMVTTGIRSVLQRLLALQKAQEEEAAAAAAAVKAEAAVKAEAAAAAVKAEAEAGAVKVEEGAAAGAPSGRAAGREEPGAAAGNGGVKVEPGSHAGALGPQPGHQQGTNVPEAPSPAPALTHVHSIRAGREASAAAAAAAAAAAAAARTVPRRESAGAAALGAGGPGATGFQPGAAGAGDAAATGPDDLLEEEGEEGGGAGGEHEAAEAGAAGDGGTGNEFEVDFEDDL

>Bdi:Bradi2g47267

MEFQVDGALWPESRGGAAEAPPPPPPKERGEAPPPRFDSSRALRLLRDLGTNVTEDLVVLLPNLLSFLKHDDPAVVKQSIASGTNLFAAVLEEMALQINECGRVDAWLEQMWASMNQFKDAVCGMMHEPGPIASKLLALKFIETWILCLTPQSNSERMQSTEGKNRRFDASRLPKFHPSLDPVVLEADTNRAFTILVDIVQSAYAHRGSLLVGTINSLAAIGKNRPGYYDRALSVLFGFDPNLETSKGAHSASLRYSLKTAFIGFLRSPCQAMIESKDILVRRLRALSPGEATEQIIRQVEKMSRNIERTSRASKEELPAWEVPHGDLNRKNPAARSGDTLAMAEGIAKRARFDSSAGSNLLVQGMPDYSDMQIENDANVGHSSDPPSLLSTDVSPVEKMIEMIGALLAEGERGAESLGILISSVESDVMADIVIETMKHLPEAPFPLDEVGISPPDALPTPGVSDSKRDPRRDPRRLDPRRTVAPAATSPIQVKVETTSVHQTDNFSNIPSPISGKVENHADYSGDLPENEDEEHTSSQPDETIGKEISESLENGTEPETNFEVQAPVEARFNSSDVDGEKTNPLSQEAISNDEFDSMDLDVDPFSPVSKASTPEETNHELPLLPSHLELSDSEKLSLHKLTVRRIIDDYKNNSLNTRFSLLAHLVAQSTADDNIMELIQKHIIFHYHDQKGHELAMHVLYQLQCVNVADSPESSTPASKHYEKFFISLARSLIDSLPASDKSFSKLLCDAPCLPESLFRLLESLCMSQGNSQQTKDSDGDRVTQGLGTVWSLILVRPPLRQACLDIVLKCAIHSQDEVRGKAVRLVSKKLYDLTYAAEKVEQFATESLLAVANKHGVETDVNFTTSKDCTTEFEVGSQETSVSGSQISDAGSSENGSAKTPLASPKQSAVSVSEAKRHSSLFFALCTKRPTLLGHLFNVYGMSPKVVKQCIHWHIPTLVRNLGSSCSEMLDIIHNPPEGSEELVIMILQTLTEESNPSAKLVVAVKHLYETKLKDASILIPLLSSFPKEEVLPIFPRLVDLPPDKFQDALARILQGSAHTGPALTPAEVLIAIHDINPEKDQVPLRKVIDACTACFEQRTVFTQQVLEKSLNKLVDNVPVPLLFMRTVIQALDAFPALVDFVMEILSRLVNKQIWKMPKLWVGFLKLAFQTQPRSFDVLLQLPPAQLEIALNKYPNLRTHLSSFVNQRNAHGILPRQTLKVLGFINEPQQASVPFVPASLQTADTTSSLPGATLM

>Bdi:Bradi1g16570

MAVAAAPRAFLPQGRTQPAAGEMGPRLRQLRELRRVPLQELVARLADLRSDEASPVRKVVAEMIGEVGSKKMVYIPDMMPYLLDLLDDETPAVARQAVKTGTNLFAKVLQQLVIQGLFSSGGIDDSLKLSWEWMLKLKSAVSLLAFQSTSNEGVRLLAIKFVEKTVLMHTPDPNITSDPPNQATEDMGFNIAWLRGGHPLLNVGDLAMEASQSLGQLLEQLKSPKISSLSTSMIIVFVSSLSAIAQRRPSFYGRILPVLLSLDPTNAIIKVQVPGAFHALKSAFDACLKCTHSSAEPWRARLLEAQNIINQGDSIEHNANAGRSVEETSNKAESLPLTETSTDNSNKRSLADDMNNILEDDGHSSKRVRQSHDAEEHSEEARNIEVASIDSSSNPPAPARTGNSEAVYQLISMFAALAAQGDRAAGSLQILSSSIAADLLAEVVMVNMQHLPVSRPEVDKQQPPSTSQSSQSSNPISGRFPLLESLLKTIKEADQDEVPPVNDSTLVTSSAGDVAPVIASSAVPTATNPPKEENSDSSAVPLDMEIVEAKVPSADATGLSIEIQESSETSHASTEPQGTQEHSGSFISSLPADNSSVGVSLAQSSETRSPTSSTIEGSQSQFSSLNSLTSQHVLPKLVVTNIDLTDEAKDLLQKEAFLRILERDKQEESSGSNTRLPLLSHLGVEFPLELDPWELLQKHVLSDYVNNEGHELTLCILNRLYREAEQDQDFLSSRTATSVYESFVLTVAENLRDMFPASDKSLGKLLCEMPYLSDGVLKLLESLCSPGNNEKQDKDLQSGDRVTQGLSAVWNLIMLRPSNRDRCLEIALQSSINRLDEVRMKAIRLVANKLFPMASISKRIEDFANEKLDSVLEVIPATESASAAEMATSEVHEDGGLENSASVAEAQTLMSLYFALCTKKHSLLRRVFAIYGSLPQSAKQAVHRQVPILIRTIRSSPDLLGIISDPPADSRDLLMQVLQTLTDGAVPSQDLISSIKNLYSKTKDAEFLFSVMAHLTKDEVMSVFSNIVNLPMDKFQVALSRILQGSPQHGPSLDPSEILIAIHVIDPEKEGIPLKKVMDACAACFEQRTIFTQQVLAKALNQLVEQIPLPLLFMRTVMQAIGAFPALVDFVMEIMSRLVSKQIWKYPKLWVGFLKCAILTKPQSYGVLLQLPAPQLENALNKNPVLKAPLVEHASQPNVRSTLPRSSLVVLGLAEDPQPEPAPEAQSSQNQAAETSSSAADTTTEVTQESSAVS

>Osa:LOC_Os07g49320

MAVAAAQDLPPTFHPNPSPLPSSMEPRLRQLRRVPLLDFVARIADLHADQASPVRKLVAEMIGEVGSKHMAYLPNVMPCLLHLLNDDTPAVARQAIKTGTTLFAKVLRQLVIQGLFSSGGIDDSLKLSWEALLKLKSAVSHMAFQPMSNEGARLLAIKFVEKTVLLYTPDLDTPPDPPIEVTEDMGFNVAWLRGGHPLLNVGDLAMEASQNLGLLLEQLKPPKVKSLSTSMIIVFVTSLSAIAQRRPSFYGRILPVLLSLDPASSIIKVQVPGAFHALKSAFAACLKCTHSSAEPWRARLLEAQNIINQADSIEHSSNRVESLPLETTSTDNSNKRNLIDDIDNAPEDGDRSNKRIRQSHHDQEHTENVKNNVELTSADTPSSPSNSASTGNSEAVYQLVSMFAALAAQGDRAAGSLQILSSSIAADLLAEVVMVNMQHLPVSHPEVDQQQSPSAGQPSGAPSSSLLSACFPLLESLLKRINQNDREVDEAPQTIDSAVVPSAAGETAAIPAIPGPTSRNVPMEENSNSSSIPSDMETIEAKEPTADAARLSIEIQESSEASHASTELQGTQEHGGSFISSLPADNSSAGLSLAQSSETRSPSSSMVEASQTQFSYSSTLTSQHVLPKLVVTNIDLSDEAKDLLQKEAFLRILDCDKQDASGGSIARLPLLAHLGVEFPLELDPWELLQKHVLSDYVNNEGHELTLCILNRLYREAEQDQDFLSSRTATSVYESFLLTVAENLRDMFPASDKSLGKLLCEIPYLPEGVLKLLEGLCSPGSNEKQDKDLQSGDRVTQGLSAVWNLIMLRPSNRDRCLEIALQSSIHHLDEVRMKAIRLVANKLFPMASISKRIEDFANEKLNSVLEVVPADESAASEMSTPEAPKDGGSENLSSSVADSQTLMSLYFALCTKKHSLLRHVFAIYGSLPQAAKQAVHRQVPILIRTIGSSPSLLGIISDPPADSRDLLMQVLQTLTDGAMPSQDLISSVKNLYSKTKDIEVLFAVLAHLPKDEVLPVFPSIVNLPLDKFQVALSRILQGSPQNGPSLDPSEILIAIHVIDPEKEGIPLKKVIDACAACFEQRTIFTQQVLAKALNQLVEQIPLPLLFMRTVMQAIGAFPALVDFVMDIMSRLVSKQIWKYPKLWVGFLKCAILTKPQSYGVLLQLPAPQLENALNKNPVLKAPLVEHANQPNVRSTLPRSTLVVLGLAEDQQQPAPQAQSSQNQAAETSSSAADTTTEVTQESSAVS

>Osa:LOC_Os01g49940

MEFEADGARWPEPRGDAAGAPPLERGDAPSPRFDSSRALRLLRELGSNVTEDLVVLMPNLLSFLKHDDPVVVNQSIASGTNLFAAVLEEMTLQINKCGRVDAWLEEMWAWTKQFKDAVHNLIHESVPVATKLFAVKFIETWILCFAPQSKSDRMQPTEGRNRRLFDSSRLSQFHPSLNPAVLEADANRALILLVDILQSACAHQGSFLVGTINSLAAIAKNRPVYYERILPVLLGFDPSLEVAKGAHPASLRYSLKTAFLGFLRSPCQAMIESKDTLVRQLRVLSPGEATEQIIRQVEKMTRNIERASRASKDEPSTLDMPYGDVSRKYPAARSSDAFATADGVAKRARFDTSAALNPPFQGASDYSNMQVDNEANVDHSSDPALLNCDMSPVEKMIEMIGALLAEGERGAESLGILISTVEADVMADIVIETMKHLPETSILLATSNNGQQKKIQSSSSPLTENLPANSHSMPYSTQFALPADGVSMSMSDVPVVSGVHDSKRDPRRDPRRLDPRRTVAPAATSSIHVKGETTGVHQTNNLSNVPYPVSGKVENSLDYSGDLSKNEDVQQTSCQPNQSLPKENSEILDDALELEPKFEVQALADVGFHSSGVDKEMVNPLSPEATSNNELDSVELEVDPFSPVLKASTLEDTTNHDLPVLPSHLELSDDEKILLHKLAIRRIIDDYKKNSVNTRFSLLAHLIAQSTADDNIMDLIQRHIIYHYHDQGHELAMHVLYQLHSVSVADSPESTLPASKNYENFFISLARSLIHSLPASDKSFSKFLCDAPYLPESMLKLLEDICVSQGNSQQTKDSDGDRVTQGLGTVWSLILARPPLRQDCLDIALKCAIHSQDEVRGKAVRLVTKKLYELTYASERVEQFAIDSLLAIANKHGVETDINFTSLKESSPEFEAGSQETSVSGSHISDAEPSESTCNKTDLVSPKQSAVSVSEAKRHTSLFFALCTKRPILLQHLFNVYGRSPKVVKQCIHWHIPNLVRNLGSSCSEMLAIIHNPPEGSEELVTLILQTLTEDSTPSAELVAAVKHLYKTKLKDASILIPLLSSFPKEEVLPIFPRLVDLPPDRFQDALARILQGSAHTGPALTPAEVLIAIHDINPEKDRVALKKVTDACTACFEQRTVFTQQVLEKSLNKLVDNVPIPLLFMRTVIQALDAFPALVDFVMEILSRLVNKQIWKMPKLWVGFLKLAYQTQPRSFDVILQLPPPQLEIALNKYPNLRTPLCSFVNQRNMHSILPRQILKVLGFINEPHQAPIPFVPAAMQTADATSSLPGATLM

>gi|124028529|[Homosapiens]

MASGSGDSVTRRSVASQFFTQEEGPGIDGMTTSERVVDLLNQAALITNDSKITVLKQVQELIINKDPTLLDNFLDEIIAFQADKSIEVRKFVIGFIEEACKRDIELLLKLIANLNMLLRDENVNVVKKAILTMTQLYKVALQWMVKSRVISELQEACWDMVSAMAGDIILLLDSDNDGIRTHAIKFVEGLIVTLSPRMADSEIPRRQEHDISLDRIPRDHPYIQYNVLWEEGKAALEQLLKFMVHPAISSINLTTALGSLANIARQRPMFMSEVIQAYETLHANLPPTLAKSQVSSVRKNLKLHLLSVLKHPASLEFQAQITTLLVDLGTPQAEIARNMPSSKDTRKRPRDDSDSTLKKMKLEPNLGEDDEDKDLEPGPSGTSKASAQISGQSDTDITAEFLQPLLTPDNVANLVLISMVYLPEAMPASFQAIYTPVESAGTEAQIKHLARLMATQMTAAGLGPGVEQTKQCKEEPKEEKVVKTESVLIKRRLSAQGQAISVVGSLSSMSPLEEEAPQAKRRPEPIIPVTQPRLAGAGGRKKIFRLSDVLKPLTDAQVEAMKLGAVKRILRAEKAVACSGAAQVRIKILASLVTQFNSGLKAEVLSFILEDVRARLDLAFAWLYQEYNAYLAAGASGSLDKYEDCLIRLLSGLQEKPDQKDGIFTKVVLEAPLITESALEVVRKYCEDESRTYLGMSTLRDLIFKRPSRQFQYLHVLLDLSSHEKDKVRSQALLFIKRMYEKEQLREYVEKFALNYLQLLVHPNPPSVLFGADKDTEVAAPWTEETVKQCLYLYLALLPQNHKLIHELAAVYTEAIADIKRTVLRVIEQPIRGMGMNSPELLLLVENCPKGAETLVTRCLHSLTDKVPPSPELVKRVRDLYHKRLPDVRFLIPVLNGLEKKEVIQALPKLIKLNPIVVKEVFNRLLGTQHGEGNSALSPLNPGELLIALHNIDSVKCDMKSIIKATNLCFAERNVYTSEVLAVVMQQLMEQSPLPMLLMRTVIQSLTMYPRLGGFVMNILSRLIMKQVWKYPKVWEGFIKCCQRTKPQSFQVILQLPPQQLGAVFDKCPELREPLLAHVRSFTPHQQAHIPNSIMTILEASGKQEPEAKEAPAGPLEEDDLEPLTLAPAPAPRPPQDLIGLRLAQEKALKRQLEEEQKLKPGGVGAPSSSSPSPSPSARPGPPPSEEAMDFREEGPECETPGIFISMDDDSGLTEAALLDSSLEGPLPKETAAGGLTLKEERSPQTLAPVGEDAMKTPSPAAEDAREPEAKGNS

>gi|226437613|[Musmusculus]

MASSSGDSVTRRSVASQFFTQEEGPSIDGMTTSERVVDLLNQAALITNDSKITVLKQVQELIINKDPTLLDNFLDEIIAFQADKSIEVRKFVIGFIEEACKRDIELLLKLIANLNMLLRDENVNVVKKAILTMTQLYKVALQWMVKSRVISDLQEACWDMVSSMAGEIILLLDSDNDGIRTHAIKFVEGLIVTLSPRMADSEVPRRQEHDISLDRIPRDHPYIQYNVLWEEGKAAVEQLLKFMVHPAISSINLTTALGSLANIARQRPMFMSEVIQAYETLHANLPPTLAKSQVSSVRKNLKLHLLSVLKHPASLEFQAQITTLLVDLGTPQAEIARNMPSSKDSRKRPRDDTDSTLKKMKLEPNLGEDDEDKDLEPGPSGTSKASAQISGQSDTDITAEFLQPLLTPDNVANLVLISMVYLPETMPASFQAIYTPVESAGTEAQIKHLARLMATQMTAAGLGPGVEQTKQCKEEPKEEKVVKPESVLIKRRLSVQGQAISVVGSQSTMSPLEEEVPQAKRRPEPIIPVTQPRLAGAGGRKKIFRLSDVLKPLTDAQVEAMKLGAVKRILRAEKAVACSGAAQVRIKILASLVTQFDSGFKAEVLSFILEDVRARLDLAFAWLYQEYNAYLAAGTSGTLDKYEDCLICLLSGLQEKPDQKDGIFTKVVLEAPLITESALEVIRKYCEDESRAYLGMSTLGDLIFKRPSRQFQYLHVLLDLSSHEKDRVRSQALLFIKRMYEKEQLREYVEKFALNYLQLLVHPNPPSVLFGADKDTEVAAPWTEETVKQCLYLYLALLPQNHKLIHELAAVYTEAIADIKRTVLRVIEQPIRGMGMNSPELLLLVENCPKGAETLVTRCLHSLTDKVPPSPELVKRVRDLYHKRLPDVRFLIPVLNGLEKKEVIQALPKLIKLNPIVVKEVFNRLLGTQHGEGNSALSPLNPGELLIALHNIDSVKCDMKSIIKATNLCFAERNVYTSEVLAVVMQQLMEQSPLPMLLMRTVIQSLTMYPRLGGFVMNILARLIMKQVWKYPKVWEGFIKCCQRTKPQSFQVILQLPPQQLGAVFDKCPELREPLLAHVRSFTPHQQAHIPNSIMTILEATGKQEPEVKEAPSGPLEEDDLEPLALALAPAPAPAPAPAPAPAPAPRPPQDLIGLRLAQEKALKRQLEEEQKQKPTGIGAPAACVSSTPSVPAAARAGPTPAEEVMEYREEGPECETPAIFISMDDDSGLAETTLLDSSLEGPLPKEAAAVGSSSKDERSPQNLSHAVEEALKTSSPETREPESKGNS

>gi|24644386|[Drosophilamelanogaster]

MDSIIGRSQFVSETANLFTDEKTATARAKVVDWCNELVIASPSTKCELLAKVQETVLGSCAELAEEFLESVLSLAHDSNMEVRKQVVAFVEQVCKVKVELLPHVINVVSMLLRDNSAQVIKRVIQACGSIYKNGLQYLCSLMEPGDSAEQAWNILSLIKAQILDMIDNENDGIRTNAIKFLEGVVVLQSFADEDSLKRDGDFSLADVPDHCTLFRREKLQEEGNNILDILLQFHGTTHISSVNLIACTSSLCTIAKMRPIFMGAVVEAFKQLNANLPPTLTDSQVSSVRKSLKMQLQTLLKNRGAFEFASTIRGMLVDLGSSTNEIQKLIPKMDKQEMARRQKRILENAAQSLAKRARLACEQQDQQQREMELDTEELERQKQKSTRVNEKFLAEHFRNPETVVTLVLEFLPSLPTEVPQKFLQEYTPIREMSIQQQVTNISRFFGEQLSEKRLGPGAATFSREPPMRVKKVQAIESTLTAMEVDEDAVQKLSEEEFQRKEEATKKLRETMERAKGEQTVIEKMKERAKTLKLQEITKPLPRNLKEKFLTDAVRRILNSERQCIKGGVSSKRRKLVTVIAATFPDNVRYGIMEFILEDIKQRIDLAFSWLFEEYSLLQGFTRHTYVKTENRPDHAYNELLNKLIFGIGERCDHKDKIILIRRVYLEAPILPEVSIGHLVQLSLDDEFSQHGLELIKDLAVLRPPRKNRFVRVLLNFSVHERLDLRDLAQAHLVSLYHVHKILPARIDEFALEWLKFIEQESPPAAVFSQDFGRPTEEPDWREDTTKVCFGLAFTLLPYKPEVYLQQICQVFVSTSAELKRTILRSLDIPIKKMGVESPTLLQLIEDCPKGMETLVIRIIYILTERVPSPHEELVRRVRDLYQNKVKDVRVMIPVLSGLTRSELISVLPKLIKLNPAVVKEVFNRLLGIGAEFAHQTMAMTPTDILVALHTIDTSVCDIKAIVKATSLCLAERDLYTQEVLMAVLQQLVEVTPLPTLMMRTTIQSLTLYPRLANFVMNLLQRLIIKQVWRQKVIWEGFLKTVQRLKPQSMPILLHLPPAQLVDALQQCPDLRPALSEYAESMQDEPMNGSGITQQVLDIISGKSVDVFVTDESGGYISAEHIKKEAPDPSEISVISTVPVLTSLVPLPVPPPIGSDLNQPLPPGED

>gi|25149975|[Caenorhabditiselegans]

MDYIQGLNEENETASERIGEALKEARDAETIEKKLLSLSTAMHLLIDPSLSISILDNFLTEMLEFAELNDSRILCLLVDFLLKASAKDFTLCNKTVERYSFYLIPNKSIKRYESVIKRVVVASTNLYPIVLEFAIMDKNDNAESCWDAFNLLKNRICMLVSDDHEGVRTVTVKFLEALILCQSPKPRELATGSNISWAREANTRFNRISLSDVPRSHRFLSYHKTQLEAEENFSALLKQTTVAEATSQNLITVIESLCMITRCRPQWENALPRVFDVIKALHSNVPPMLSKGQVKFLRKSFKYNLLRFLKLPASVPLQQKITTMLTNYLGASPREVQQSIPPELIQKIAPPRPPQHPAEPVAKRPKIQNQIFEDDDDDDDEAGPSTSTVNAKDARTEAIDMTAKFIMECLNHETVMNLVKISLYTLPSEMPAAFASSYTPIANAGTEPNRQELSELMAVQMTNKEIGPGYEWLQQQRKKEYEARNKARSEGMAIAQTPIHEPNMSNRVPAQIVKQSLQEINTLPVIQKAKKAFNLVEEAVVFDDKEAAEMFELAYESVLQAERRVVAGGARLMYQKLVVRLTTRFWEDCTPFEEKLIEFVLADHKKRNDLALLWLCELYAQYQGYSNCALFMKEMIAGQEGLTQAQRLDRYDQAMCKMLDAMLERNMEKEALFYKVLLETPLLTPNAIERLKQVCLAKENEHGMAMLRELIMTRNRQRPQLLQFLFGLFFMERPELRSSCLEVVKELCYLPFIRSSLSDQARMQIHDCLQESPPMYMRSSEDSDQWTDEMYKNSLAVYSTLMPSDPLLLIPLASVYAQSTNVFKRVVLRSLEPVFRQLSQEMVISLIEDCPYGAETLVARLVVLLTERITPSTDLIQKLKILHDERKMDIRALLPIIGGLEREEVVRLIPTFIFRAEYQKSVNVLFRKLYTVRDPQTGNLVFDPIEVIKEYHKIEPKNDNEAELLVNNLEFLFDPALLKPDTASQAIEAVFKWENVPFLFLHSLYTLFHKFKTFESFVANLFYKVTEKKMYQQSDRWKQAFFKCIKELKTKAYPAVITFLSFEEYEELKEVLGDGIVAEFKIIYSTLATQQQKNMDEKIKEELHDKERENRERDKRLRREEKKEKEREKERTRESGKERSSRR

>Pta1p

MSSAEMEQLLQAKTLAMHNNPTEMLPKVLETTASMYHNGNLSKLKLPLAKFFTQLVLDVVSMDSPIANTERPFIAAQYLPLLLAMAQSTADVLVYKNIVLIMCASYPLVLDLVAKTSNQEMFDQLCMLKKFVLSHWRTAYPLRATVDDETDVEQWLAQIDQNIGVKLATIKFISEVVLSQTKSPSGNEINSSTIPDNHPVLNKPALESEAKRLLDMLLNYLIEEQYMVSSVFIGIINSLSFVIKRRPQTTIRILSGLLRFNVDAKFPLEGKSDLNYKLSKRFVERAYKNFVQFGLKNQIITKSLSSGSGSSIYSKLTKISQTLHVIGEETKSKGILNFDPSKGNSKKTLSRQDKLKYISLWKRQLSALLSTLGVSTKTPTPVSAPATGSSTENMLDQLKILQKYTLNKASHQGNTFFNNSPKPISNTYSSVYSLMNSSNSNQDVTQLPNDILIKLSTEAILQMDSTKLITGLSIVASRYTDLMNTYINSVPSSSSSKRKSDDDDDGNDNEEVGNDGPTANSKKIKMETEPLAEEPEEPEDDDRMQKMLQEEESAQEISGDANKSTSAIKEIAPPFEPDSLTQDEKLKYLSKLTKKLFELSGRQDTTRAKSSSSSSILLDDDDSSSWLHVLIRLVTRGIEAQEASDLIREELLGFFIQDFEQRVSLIIEWLNEEWFFQTSLHQDPSNYKKWSLRVLESLGPFLENKHRRFFIRLMSELPSLQSDHLEALKPICLDPARSSLGFQTLKFLIMFRPPVQDTVRDLLHQLKQEDEGLHKQCDSLLDRLK

Figure S8.

>Sbi:Sb01g012650

MSKAKTNNGYLGVTEPISLSGPTDKDLMQTTEVEKYLSDAGLYESQDEAVLREEVLGKLDQTVKAWIKKATRISGYGEQFVHEANAKIFTFGSYRLGVHGPGADIDTLCVGPRHATRNEYFFRWLHDMLAEMPEVSELHPVPDAHVPVLGFKINGVSIDLLYANLAHAVIPEDLDLSQDSILNNVDEQTVRSLNGCRVTDQILRLVPNILSFRTTLRFIRYWGKRRGVYSNVMGFLGGINWAILVGRICQLYPNASPSMLISRFFRVYSKWKWPNPVMLCHIEEGYLGLPVWDPRRNYRDRGHQMPIITPAYPCMNSSYNVSVSTRYVMTQEFTRAFEICQAIDEGKADWDALFEPYPFFESYKNYLEVNITARNEDELRSWKGWVESRLRTLVLKIERYSHEMILAHPYPKDFSDKSRPLHCFYFMGLWRKQTTQTQEAEQFDIRGIVNEFKNTICAYQQWKEGMDIEVSHVKRKEIPLFVFPGGVRPSRSSRTAHKNSRTVPTCDVSADDQVGNLLGVASCSDAQPVSCKGSYMKQPEPDCAGGFQLPGSTSVLPPSLPNKVALNGSANFHAESVEHEHPEHYQESKFATVQNAVRNVVKQPNSLLPNSNNAWQLYGSDSSLNNSQRECAGSAANNLLNLSPAILATPDELDELVSHHQVKVNQKDVNADRRPSLEIGSENNLEQVSSLRPQDSNNNLKRKANQELEPLELAAPSTGAAPQSTASAPRKPLRLRLTTLGKPKPAEGTS

>Sbi:Sb01g037200

MALDLAVGGSAARRAETQTLAPVLLMGPPPPPPIPPTTGVYLPGPPPPGALLSRPIPMALPREVIVYMDECRSRSLLKFISDAGIVPSLEDERRRERVVRELGKIVMEWAKRVAYEQGKQHWITSATVLTFGSYALGDPHAEGGAGQGSGPGLHMKKVHSFWNPRSSCTMLAWATAYGVAVWVKSGFWAFSRACVVEAPIDNASGKAIPWDQAYGPESDIDVLCIGPYIATLQHHFFVVLRQMLEGRPEVSELQSIEGAKVPLMRFKFNGILVDFPYVQLPFINAAEAMHAFDPHVLENVDGPSWRCLSGVRANRQIIQLVPNMKLLGFFAGIHLAILAAYVCRRHPNASINTLLSLFFDIFAHWPWPLPVSLLDPPVLCRGPDGCSLMPIMLPCNPPEFCSSSTTESTFSKIKEELRRGYALTKDTRSTDFDWSWLFASFPYGARYKCFLRIVLSAPLDEELRDWVGWVKSRFRNLLLKLESLGVYCDPDSSEQVDHTITEPNVVFFWGLVFTRNIQICTSSLKEDFMKSVCNNIYGKEKCAHSDITMAIVGPPQLPKSIFDLSVYSEKLPQHMMGHQLMKQRYNAVS

>Sbi:Sb02g043400

MAYMAAVAPVPWWPPPPELAPVGFPDASSPAGYPKPQTLPFLLAPTPPPPPPPPPLPAGYPLLPPPAPIIIQLQPDPSFVAEVDQRRSSSLVQFLKDEGAVPSPEDEKKREKVIRELKKIVMHWANAVAYEQSVPQGLATATVLTYGSYTLGAHGPESDIDVLCVGPCIATLQYHFFVVLRQLLEGRPEVSELQTIEKAKVPLMRFRFTGIAVDFTYAQLPVIDALKKFQALLRCIKLWARKRGLHCHYLGFFAGIHLAILAAYVCRKFPDASVNGLFAVFFQTFAHWPWQVPDSLRHDFQWTWLFEPFPYDKKYQQFLRIALCAPTFAELRDWAGWVKSRFRLLILKLERAGIECDPCPSEEVDHTDNDPNVVFYWGLIPERIIQVDTSSLKEDFMESITNDVYGTVKCTHSDVTISVVGLPQLPKSMRSHVHWQYMQRCMMAYEGTDEGQSAGWLGLG*

>Sbi:Sb04g008100

MAGSVGKGRAALRSSPKRYSGTDPPLSLAGPTLADLQRTAELEKFLVEAGLYEGKEESAKREDVLCEIGQIVKEWVKQLTSKKGYADQLVEQANAVLFTFGSYRLGVHGPEADIDTLCVGPSYVNREEDFFVTLHGILAEKEEVTELQPVPDAHVPVLKFKFRGISIDLLYASLSLSVIPADFDISQGSVLCDVDEATVRSLNGCRVADQILRLVPNVENFRTTLRCLKYWAKRRGVYSNITGFLGGVNWALLVARVCQLYPNAVPSMLVSRFFRVFTQWQWPNPVMLCAIENNDLGFSIWDPRKNPRDRNHLMPIITPAYPCMNSSYNVSSSTLRVIMEQFQFGNKICQEIELNKANWNALFEPFHFFEAYRKFLVVDIVAENDDDLRLWKGWIESRLRQLTLKIDRDTKGILQCHPYPCEYSDPTIECAHCAFYMGLSRKEGSKKRGQQFDIRGTVDEFMREIGMYSLWMPGMDLAVTHVQREQVPSYVFEQGYKKPCPTMHANQQEQSDGDVTLSPYLDSQLKRKYDSDGDGHVELHKSVKWASVSPPGVGTPPHGNSVSNVVCDSPVKFVSSVVCSRAQTSPSHDDINLEQAQLTTSPYGSEDTSASGTSFAAVGAVVLADESSKLGLKSYAINCLLLDGNLVSCLWELVKIRMVQVLQPKYRNCCEG*

>Sbi:Sb06g026810

MAMASSQPKQQMFGEPISLVGPTPADLEATAELEKVLREAGMYESPQESAVRAEVLRDLQGIVDRWVKQLTLKHGYPDAMVDEATALLLPFGSYRLGVHGGGSDIDALVVGPSFVDRDQDFFGVLAGALAEATEAVTDLQPVPGAHVPVMKLRFRGVQVDLVYASVNLPVVPRDLDLSDRAVLRGLDHVTARSMNGVRVADEILRLVPDAAAFRTALRCVKLWAKARGVYSNVSGFLGGVAWAILVARVCQLYPNAAPSMLVSRFFKVLSQWKWPTPVMLCDIEHDDELGLPVWDGRRNPRDRTHLMPVITPAYPCMNCTYNVSQATQRIIKEQIQAGHVACQEIAAGGDRGWGALFQPFPFFRTHKSYLQVDATVAGGEEELREWKGWVESRLRQLVAKVERDTFGELLCHQNPRAYDAEPHGLRCASSFFVGLSKPQQQRQQPSPPQGQQPQFDLRATADEFLQDVYTYRFWRPGLELAVKHVRRKDLPPYVMHKIRGPNIHELKRKRDDDDSSPSSPTLCSSSSASSCDDDSVRRPSSRARLHPT*

>Sbi:Sb10g022090

MASGSDPPKQYGITKPLSLLGPVEADLQRTAELEKFLVEAGLYESPDESARREEVLGKLDQIVKDWVKQLTSQRGYTDQMVEEANAVLFTFGSYRLGVHGPGADIDTLCVGPSYVNREEDFFIVLHGILAQTEDVTELQPVPDAHVPVMKFKFHGISIDLLYASVSLLVVPADLDISQGSVLYDVDEATVRSLNGCRVADQIIRLVPNIENFRTTLRCLKYWAKRRGVYSNVTGFLGGVNWALLVARVCQLYPNAVPSMLVSRFFRVFTQWQWPNPVMLCSIEEDEVGFPVWDPRKNPRDRCHHMPIITPAYPCMNSSYNVSTSTLRVMVEQFQFGNKICQEIEMNKASWSALFEPFQFFEAYKNYLQVDIIAEDDEDLRLWKGWVESRLRQLTLKIERDTYGMLQCHPYPHEYADPSRQCAHCAFFMGLSRKEGVKIQEGHSSIFVELWMSLGMTSICICSGSLEWS*

>Smo:169728

MEPLSTAGPTPADLGRTRDLEKLLSNAGLNESREEAMKREGVLGRLDQIVKSWVRQICVNKGFSNEVVQEANAKIFTFGSYRLGVHGPGSDIDTLCVGPSHATREEDFFVELHNILAETENVTELNPVPEAHVPVMKFKFDGISIDLLYARLSSWTIPEDLDISDESIMQNLDEQSVLSLNGCRVTDQILRLVPNIQHFRTTLRCMKYWAKRRGVYSNVTGFLGGINWALLVARICQLYPNAVPSTLVSRFFRVYTQWRWPNPVMLCPIEERSSLGLLQVWDPRKNPRDKSHLMPIITPAYPCMNSSYNVSTSTLRIMTQEFNRGNEVCEQLEMSRATWDLLFESFSFFEAYRNYLQIDVVAIDDCDHRCWKGWVESRLRQLTLKVEKDTYGMLQCHPHPSDFVDMARDGYHCAYFMGLQRKLGAPLHEGQQFDIRTTVEQFKLNVAAYTSWKPGMEIYVSHVRRKQIPLFVYPGGVKPARPARQHSGSPSSDSKQGDSRKRLTSADPGGHCSKKIASSSSSNQVVAAEEVEVS*

>Smo:231918

MAQSNGKKYLGVTEPISTAGPTEADFARTRDLEKVLTEAGLYESPEEAVQREEVLGRLDQLVKEWVKNICLRKGYSEQLTQEANAKIFTFGSYRLGVHGPGTDIDTLCVGPRHVSREEDFFGVFHGMLEAMSEVTELHPVPDAHVPVMRFKFSGISIDLLYAPLAVWTIPEDLDISQESILRNLDEPSVRSLNGCRVTDQILRLVPNIEHFRTTLRCMKLWARKRGVYSNVTGFLGGVNWALLVARICQLYPNALPSMLVSRFFRVYTQWRWPNPVMLCEIEEGSLGLSVWDPRKNPRDRTHQMPIITPAYPCMNSSYNVSSSTLRVMVEEFSRANGICEVIEMNKAEWSALFEPYAFFDAYKNYLQIDVFAADNDDLRRWKGWVESRLRQLTLKVGLDREIEKHTYGMLQCHPHPCDFVDESKEGKHCAFFMGLQKRQGLPSQEGQQFDIRLTVEEFRQSVTGYQLWKEGMDIAVSHVRRRQIPAYVFPGGTKPARPPKLSSSRKAREAGSIRGCNTSYVIEAGCERSKFWISRNPTEWTGLGSCTG*

>Aly:472012

MASVQQNGQRFGITEPISLGGPTELDVIKTRELEKHLQDVGLYESKEEAVRREEVLGILDQIVKTWIKTISRTKGLNDELLHLANAKIFTFGSYRLGVHGPGADIDTLCVGPRHATREGDFFGELQRMLSEMPEVTELHPVPDAHVPLMGFKLNGVSIDLLYAQLPLWVIPEDLDISQDSILQNADEQTVRSLNGCRVTDQILRLVPNIQNFRTTLRCMRFWAKRRGVYSNVSGFLGGINWALLVARICQLYPNALPNMLASRFFRVYNQWRWPNPVLLCSMDEGSFGLQVWDPRRNPKDRLHIMPIITPAYPCMNSSYNVSASTLRIMTGEFQRGKEICEAMEANKADWDTLFEPFAFFEAYKNYLQIDISAANVNDLRKWKGWVESRLRQLTLKIERHTYDMLQCHPHPHDFQDASRPLHCSYFMGLQRKQGVPAAEGEQFDIRRTVEEFKHTVNAYTLWIPGMEISVSHIKRRSLPNYVFPGGVRPSHTSKGTWDSNRRSEHRNSSTSSALAAATTTTEMSSESKAGSNSPVDGKKRKWGDNETLTDQLRKSKHIAVSVPVENCEGGSPNPSVGSICSSPMKDYCTNGKSEPISKDPPENVVAFSKEPAESLPIEKIATPQAPSQETEELEESFDFGNQVVEQISHKVAILTATATIPPFEATSNGSAFPNEAVEELEVLPMRQPDAAHRPSVQQRKPIIKLSFTSLGKTNGK*

>Aly:491411

MVGTQNLGGSLPPLNSPKSYGITKPLSLAGPSSADIKRNVELEKYLVDEGLYESKDDTMRREEVLGRIDQIVKHWVKQLTQQRGYTDQMVEDANAVIFTFGSYRLGVHGPGADIDTLCVGPSYVNREEDFFIILHDILAEMEEVTELHPVPDAHVPVMKFKFQGIPIDLLYASISLLVVPQDLDISSSSVLCDVDEPTVRSLNGCRVADQILKLVPNFEHFRTTLRCLKYWAKKRGVYSNVTGFLGGVNWALLVARVCQLYPNAIPSMLVSRFFRVYTQWRWPNPVMLCAIEEDELGFPVWDPRKNHRDRYHLMPIITPAYPCMNSSYNVSQSTLRVMTEQFQFGNNILQEIELNKQHWSSLFEQYMFFEAYKNYLQVDIVAGDAEDLLAWKGWVESRFRQLTLKIERDTNGMLMCHPQPNEYVDTARQFLHCAFFMGLQRAEGVGGQECQQFDIRGTVDEFRQEVNMYMFWKPGMDVFVSHVRRRQLPPFVFPNGYRRPRQSRHQNQPGGKSGEDGTVSHSGSVVERHAKRKNDSEMMDARPEKPEKRASLSPQSLDIVSPESSAITTGWTPPVCNLRRPPSEEIEAENLNTECTELTNFARNECNSGSEQVLEVDSMALVQECSDPAEPLGKCVTPDSSDVVACVSGQEENLDRNLRSVSISGTDSQPLPRLLDTKVFNPADLVGRTVIMRLGFGFPAANSDPLGKENLYSQSGMSEDLQSNSLVSGMEKSEDRASTSRLSLKSTV*

>Aly:477964

MKKGGGRNKGFPPSMEDGSSISLRQLMVNEGLIPSLEDQEKRRVVINKLRKIVVRWVKNVAWQHRLPQNQIDATNATILPYGSYGLGVYGSESDIDALCIGPFFASISEDFFISLRDMLKSRREVSEVHCVKDAKVPLIRFKFDGILVDLPYAQLRVLSIPNNVDVLNPFFLRDIDETSWKSLSGVRANKCILQLVPSLELFQSLLRCVKLWAKRRGVYGNLNGFLGGVHMAILAAFVCGYESNATLSSLLANFFYIFAHWQWPTPVVLLEDTYPATGAPPGLMPIQLPCGSHQYCNSNITRSTFYKIMAEFLRGHNLTKDYLKPNFSWKNLFELYPYENTYTWFTKIHLSAANQEDLSDWVGWVKSRFRCLLSKIEEVYGICDPNPTEYVETYTKQPNIVFYWGLQLRTINVSDIESVETEFLKNLNSGSFQGPVGRIQLSVVKESQLPKNGECASNNRSKKVTKTCWRIRENKQCNNVPVYSHHLPGYVVGYQKMANREADGMAG*

>Aly:320377

MVSSTQQRTDDGSSPPVVKALKTYGITKPLSIAGPCAADVKRNLELEKFLVDEGLYESKEETMRREEVLVRIDQIVKHWVKQLTRQRGYTDQMVDDANAVIFTFGSYRLGVHGPRADIDTLCVGPSYVNREEDFFIILRDMLAEMEEVTELQPVTDAHVPVMKFKFQGISIDLLYASISLLVIPQDLDISNSSVLCDVDEQTVRSLNGCRVADQILKLVPNSEHFRTTLRCLKYWAKKRGVYSNVTGFLGGVNWALLVARLCQLYPNAIPSMLVSRFFRVYIQWRWPNPVMLCTIEEDELSFPVWDPRKNHRDRYHLMPIITPAYPCMNSSYNVSQSTLRVMTEQFQFGNTICQEIELNKQQWSSLFEQYMFFEAYKNYLQVDVLALDAEDLLAWKGWVESRFRQLTLKIERDTNGMLMCHPQPNEYVDTSKKFRHCAFFMGLQRAEGFGGQECQQFDIRGTVDEFRQEVNMYMFWRPGMDVYVSHVRRRQLPSFVFPKGYKRPRQSRHQNQQCREPGEGVVGSLSDSVDGYAKRKNDNETMDTRPEKREKRASRSPHSLDAVSPVSSGITTGETPQIGIAPGPRAECLVTGDLVCNVRSLPNVEVEAEKFIRKSTELIKFSQYEHNSGCEQILEVDSRALVQGYHGLAEPVGKHVRPDPRAVLACEGWQNKEIGRDMGSESINDTATQHLPRRLNVKEDVDEVEREAKSGEIADGVLWNGYCGRNLDHEGFLTPANLDSAVEHRNLHADRLFKSGLSEELQSNPLLSGMGKLDDGASSRRLSLKSIV*

>Gma:Glyma04g09170

MAVSDSPSGGSTPPQQEQQPNKYAFTKPLSLAGPTDADLQRNNELDKFLLDSGLYESNEESAARKEVLHRLDQIVKNWVKQLTRQRGYTDQMVEDANAVIFTFGSYRLGVHGPGVDIDTLCIGPSYVNREEDFFVILHNILAEMEEVSELQPVPDAHVPVMKFKFQGISIDLLYASISLLVVPEDLDISHGSVLYDVDEPTVRSLNGCRVADQILKLVPNVEHFRTALRCLKFWAKRRGVYSNVTGFLGGVNWAILVARICQLYPNAIPSMLVSRFFRVYTQWRWPNPVMLCSIEENELGFPIWDPRRNPRDRFHTMPIITPAYPCMNSSYNVSASTLRVMMEQFRYGNKICDEIELNKAQWSALFQPYIFFEAYKNYLQVDIIASDADDLLAWRGWVESRLRLLTLKIERDTNGMLQCHPYPNEYVDTSKLCAHSAFFMGLQRKEGVRGQEGQQFDIRGTVDEFRQEINMYMYWKPGMDIFVSHVRRKQLPAFVFPGGYKRTRMPRHISHQAEKTGDDATKCYSGSGSGSSERCIKRKSCPEMVDKKPGKPDKRASISPQRLECVSPESCTSKSGGTTQMSIECIEVVRLAGSTTKDANDNCEVKSSDALPGSGLSTEVADMQISEPGFVDTTHDMLKSRSVEIPNENGVLNGDKAQDLALDCLESAETESTNSLSNYKEGDNDTDQRLDKECNFIPRAECSDYVPNASSQNLNCEPDVRLGSAV*

>Gma:Glyma05g34210

FSIVETKYQQRLGITEPISLGGPTEYDVIKTRELEKLLQLLPHTCKMLACMRISRRQLGSAWQARPDPCKGTKQATGARSKCQDFYFWLLLTRGMNPGANIDTLCVGPRHASRDEDFFGDLHRMLSKMPEVTELHPVPDAHVPVMRFKFNGVYIDLLYAKLSLRDLDISQELILQNADEETVCSLNGCRVTDKVLLQNFCTTLRCMKFWAKHCGLFPNALPNMLVSRFFRVYTQWCWPNPVMLCAIEEGSVGLQIWDPRRYPNDRFHLMPIITPAYACMNSSYNVSSITLCIMTEEFQRGNEICENLMAIYLLSNRVFVSPCTSMTSGHQDSVFPSPSYGFDFVVSVVSLSISNPLSSNSYKNYLQIDISGENADDLRKWKGWIERHIYGMLQCHPHPGDSSDKSRPFHCSNFMALQRKQGVPVNDGGQFDIRLTVEEFKHSVNIYTLWIPGMDVHVPHVKHRNIPTFVFPGSVRPSHPSKLRVSGHAQAEKSQEGKMAVLGANDDRKRKQAEDSVDSLRNSKSMASLPPSSREVHEDENPISPASSCFVKSNDSEINSTSEQQNTSSSKEAEKLAIEKIMCGPYDAQQVFSGEPDELEDDLEFRNQVKDFGGKMKNSIGLFKLRLQWPRNQLFLRRKPLPQLFYTQMGAQKSSRTSAIIFHIYRLFLIAVIVC*

>Gma:Glyma06g09310

MAVSDSPSGGSTPPHQEQQANSKYGFTKPLSLAGPTDADLQRNNDLDKFLLDSGLYESNEESAARKEVLHRLDQIVKSWVKQLTRQRGYTDQMVEDANAVIFTFGSYRLGVHGPGVDIDTLCIGPSYVNREEDFFIILHNILAEMEEVSELQPVPDAHVPVMKFKFQGISIDLLYASISLLVVPEDLDISHGSVLYDVDEPTVRSLNGCRVADQILKLVPNVEHFRTTLRCLKFWAKRRGVYSNVTGFLGGVNWAILVARICQLYPNAIPSMLVSRFFRVYTQWRWPNPVMLCSIEENELGFPIWDPRRNPRDRFHTMPIITPAYPCMNSSYNVSASTLRVMVDQFCYGNKICDEIELNKAQWSALFQPYIFFEAYKNYLQVDIIASDTDDLLAWRGWVESRLRMLTLKIERDTNGMLQCHPYPHEYVDTSKPCAHSAFFMGLQRKEGVRGQEGQQFDIRGTVDEFRQEINMYMYWKPGMDIFVSHVRRKQLPAFVFPDGYKRTRMPRHISHQAEITGDDATNCYSGPGLSERCIKRKNYSEMVDKKPDKPDKRASISPQRLECVSPESCTGKSGGTAQMSIECIKGVRLAGSTTKDANSNCKIKSSDALPGSGLRTEVADMQISEAGFVNTTHDMLKSRSVEVPNENEVVNGDKAQDLALDCLESAETESTNSLSNYEEGDIDMDQRLDKACNFITRAECSDYVPNASSQNLNCEMSVSEVLYKLKMGLGG*

>Gma:Glyma07g01780

MGGSEGVGASSKQFGVTKPISMAGPSATDLQRTRELEKFLAASGLYESKDEAAKREEVLHRLGEIVKSWVKQLTRLRGYTGQMVEDANAIILTFGSYRLGVHGPGADLDTLCIGPSYVTREEDFFYTLHDILANVEEVTELQPIPDAHVPVMKFKFDGISIDLLYASISRLVLPEDLDISDVSVLHNVDEPTVRSLNGCRVADQILKLVPNIEHFRTTLRCLKFWAKRRGIYSNVTGFLGGVNLALLVARVCQFYPNAVPSMLVSRFFRVYTQWRWPTPVMLCPIEENELGFPVWHPGKNPRDRSHHMPIITPAYPCMNSSYNVSTSTLRVMMDQFQYGNKICGEIELSRACWKALFEQYSFFESYKNYLQVDVVAADADDLRSWKGWVESRLRQLTLMIERDTFGKLQCHPYHHEFVDTSRLCAHCAFFMGLQRKQGEVVQEGQQFDIRGTVEEFRHSVNMYMFWKPGMEIYVSHVRRRQIPFYVVPDGYKRSRLSRPTSQVENCKSFHNEVSGTEHVERIRKRKNNDGVD

>Gma:Glyma07g11140

MGIPGLSNQNNGQQRLGITEPISLAGPTDDDAIKTLELEKYLQGVGLYESQEEAVVREEVLGRLDQIVKIWVKNISRAKGFNEQLVHEANAKIFTSGSYRLGVHGPGADIDTLCVGPRHASRDEDFFGGLQKMLSEMQEVTELHPVPDAHVPVVKFKFNGVSVDLLYARLALWVIPEDLDISQESILQSVDEQTVLSLNGCRVTDQVLRLVPNIQTFRTTLRCMRFWAKRRGVYSNVAGFLGGINLALLVARICQLYPNALPDMLVSRFFRVYTQWRWPNPVMLCAIEEGSLGLPVWDPRRNPKDRYHLMPIITPVYPCMNSTYNVTSSTLRVMSDEFRRGSEICEAMEASKADWDTLFEPYPFFESYKNYLQIDITAENADDLRQWKGWVGSRLRQLTLKIERHTYGMLQCHPHPGEFSDNSRPFHHCYFMGLQRKQGVPVNEGEQFDIRLTVEEFKHSVNAYTLWKPGMDIHVSHVKRSNIPNYIFPGGVRPTFPSKPQGGKAVAVGADDVRKRKRSEDNMDNNPRNSRSPVSLPPPSREVHEDISPISASSSCSMKFDESEVNSIGGQKSEKLCLKSPGEIPSGDSGTNGSVTSNQQVNPVLAAADTSNSKEEEKLAIEKIMSGLYDAHQAFQEEPKELEDNTQYKNQDKDSSANMKNNMESLDSKPAVPEEPVISKEITCSTHLCSNESLEELEYMCMHNVWLSLLVVFSFLSIYFRQNLVEIFFNGVQYKYDHMTLSHKSDRFLYEFLARVLHWHMFLLFNPSYNIISWIIFLCFCFLVPSHPEYAFVIT*

>Gma:Glyma08g21440

MGGSEGVGASSKQFGVTKPISMAGPTVTDLQRTRELEKFLAVSGLYESKEEAAKREEVLHRLGEIVKNWVKQLTRLRGYTDQMVEDANAVILTFGSYRLGVHGPGADLDTLCIGPSYVNREEDFFYTLHDILANIEEVTELQPIPDAHVPVMKFKFDGISIDLLYASISRLIVPEDLDISDVSVLHNVDEPTVRSLNGCRVADQILKLVPNVEHFRTTLRCLKFWAKRRGIYSNVTGFLGGVNLALLVARVCQFYPKAVPSMLVSRFFRVYTQWRWPTPVMLCPIEENDLGFPVWHPGKNPRDRSHHMPIITPAYPCMNSSYNVSMSTLRVMMEQFQYGNKICGEIELSKDCWKALFEKYSFFESYKNYLQVDVVAADADDLRSWKGWVESRLRQLTLMIERDTFGKLQCHPYHHEFVDTSRQCAHCAFFMGLHRKEGEVVQEGQQFDIRGTVEEFRHSVNMYMFWKPGMEIYVSHVRRRQIPCYVFPDGYKRSRPSRPTSQFENCKSFHNNEVSVTEHVERIRKRKNNDGVDVREDAIVKRQYACPPGDRLARRNSLGTGGLSVEAVSDSQELRSVECNHLSNSGQDDLDRTESPEAASNSSVITSVGSSEDINSRGVEMMNNGRFEDTTYGNDSVTLVKNIVASGNEVFQHELQQQLQPNAMLGMVLDSAEKVLSEAVQEPVIRRLSLPSTV*

>Gma:Glyma09g31080

MGIPGLSNQNNGQQRLGITEPISLAGPTEDDVIKTRELEKYLQGVGLYESQEEAVGREEVLGRLDQIVKIWVKNISRAKGFNEQLVHEANAKIFTFGSYRLGVHGPGADIDTLCVGPRHASRDEDFFGELQKMLSEMQEVTELHPVPDAHVPVMKFKFNGVSVDLLYARLALWVIPDDLDISQESILQNVDEQTVLSLNGCRVTDQVLRLVPNIQTFRTTLRCMRFWAKRRGVYSNVAGFLGGINLALLVARICQLYPNALPNMLVSRFFRVYTQWRWPNPVMLCAIEEGSLGLSVWDPRRNPKDRYHLMPIITPAYPCMNSTYNVTSSTLRVMSDEFRRGSEICEAMEASKADWDTLFEPYPFFESYKNYLQIDITAENADDLRQWKGWVESRLRQLTLKIERHTYGMLQCHPHPGEFSDNSRPFHHCYFMGLQRKQGVPVNEGEQFDIRLTVEEFKHSVNAYTLWKPGMNIHVSHVKRRNIPNYIFPGGVRPTFPSKVTAENKQSSKSRVPGHGQAEKPQGGKTVVVGADDVRKRKRSEDIMDNNPRNSKSPVSLAPPSREVNEDISPISASSSCSMKFDESEVNSIGGQKSEKPCLNSPGEIPSGDSGTNGSVTNNQQVNPVLAAADTSNSKEEEKLAIEKIMSGPYDAHQAFPEEPEELEDDTQYKNQDKDSGGNMKNNMESLLSKPAVAEEPVISKEITCSTHLFSNEILEELEPAELSAPLLSGPPAPLPMKKPLIRLNFTSLGKAADKSA*

>:GSVIVT01008352001

MSNLGLNNRNNSGQRLGITEPISLGGPNELDVTKTQELEKFLAAAGLYESQEEAVSREEVLGRLDQIVKIWVKAISRAKGLNEQLVQEANAKIFTFGSYRLGVHGPGADIDTLCVGPRHATREEDFFGELHKMLSEMPEVTELHPVPDAHVPVMRFKFSGVSIDLLYAKLSLWVIPEDLDVSQDSILQNADEQTVRSLNGCRVTDQILRLVPNIQNFRTTLRFMRFWAKRRGVYSNVAGFLGGINWALLVARICQLYPNALPSMLVSRFFRVYTQWRWPNPVMLCAIEEGTLGLQVWDPRKYPKDRFHLMPIITPAYPCMNSSYNVSSSTLRIMSEEFKRGNEISEVMEANKADWATLCEPYPFFEAYKNYLQIEIAAENADDLRKWKGWVESRLRQLTLKIERHTYNMLQCHPHPGDFSDKSRPFHCCYFMGLQRKQGVPASEGEQFDIRLTVDEFKHSVGMYTLWKPGMEIHVIHVRRRNIPNFVFPGGYGYKYVGKDKERKIDGSVRCSHPIKTLSSSGGSPSSTEAEKIAIEKIMSGPYVSHQAFPGELDELEDDVEYKNQVKDFTGSTKGSSAESSKANVAEEPLTTTSGTVPCTILSPNGGLEELEPAELMPPLSYGNRPSSTEQKKPIIRLSFTSLAKATGKST*

>:GSVIVT01011814001

MASVSASNQVNTLCLGVSEPISTAGPTEFDLIKTRELEKFLADSGLYETREEAIRREEVLGRVDQIVKVWVKTVSRAKGFNEQLVHEANAKIFTFGSYRLGVHGPGADIDTLCVGPRHATRDEDFFGELHRMLAETPEVQELHPVPDAHVPVMKFKFNGVSIDLLYARLSLWVIPEDLDISQETILQNVDEQTVRSLNGCRVTDQILRLVPNIQNFRTTLRCMRFWAKRRGVYSNVSGFLGGINWALLVARICQLYPNAVPSTLVSRFFRVYTQWRWPNPVMLCPIEEKCLGLPVWDPRRNIKDRNHLMPIITPAYPSMNSSYNVSWSTLRIMEEELQRGNEIVKEMETENTGWITLFEPFLFFEAYKNYLQIDITAENDVDLRNWKGWVESRLRLLTRKIERDTGGMLQCHPHPAEFSDTSRSFHYCYFMGLRRKEGSAAQEGEQFDIRLTVDDFKNTVWMYSSWKRGMWIHVCHIRRKNIPNFVFPGGVRPPQPMKVAGEQGQVSKPMGFNSQKRKRDEIDAGSHPRESTSLVENVESDLQGEVKLEQVEVNVGDSLTKVICSSENSSHNSIELEGYARCSQPLGAASSASGSLSLGRALVIETTSEPLVHQCSSKEVGESQVGLVDQVQNFDVGKRVNCVQSSTAERGMAAAAAGQGFSPCSSLFQNGGLEELEDAELTAPFYHGNPGASSTPQKHLIRLSVNSLMGLQSQLNDVSSDSIPLLLVAIIANCVAYIRSLLLGLFQSMGLSRFDADEVEDGLLGFPAYVFFPLVSKFQAKQKNHCSLEMNPHVGMSLMSPP*

>:GSVIVT01015292001

MENVKVRAKQFGLTKPISYVKPTDFHIRRSFELEKVLWDGGVYQVEEEARKREEIIEKLRVVVKSWVKQVTRWKGYTDKMVENANALIVTFGSYRLGVHGPGSDIDTLCIGPSYVNREEDFFIRLHNILIGMEGVSELLPIPHAHVPVMKFKFEGVSIDLLYASVSHLVVPDDLDISNESILYEADEPTVRSLSGCRVADQILRLVPNVEHFRTTLRCLKFWAKRRGVYSNVTGFLGGVNLALLVARVCQLYPNANPSMLVSRFFRVYTQWHWPNPVMLCPIEDKELGFPVWDPRRNPLDRNHHMPIITPAYPNMNSSYSVSTSTLEAMMKQFHTANKICNDIELNKSSWGALFEPFLFFRSYQNYLQVDITATDADDLRAWKGWVESRLRQLTLKVERCTIGKLLCVPCPREYVDTSRQCCHCTYFMGLRKKPGVEVGEVIDIRVATQEFKEEIVNMFSFWTPGMEIHVSHVLKNQLPSYVFPDEYRKRSQSSKSINQQHQNKRKINSEMVDGKPSASGCMSGGMSNSSSQLKADMGSCKTGYKRRRLTPEEGSCEESKGLSVSESESESDGLAKEKGSSLLQNALLKEVEVSENFL*

>:GSVIVT01016969001

MVSSKGLGDSPPRQSVKQYGVTKPISVAGPTEVDIQRSLELEKFLVDAGLYESKEEAIKRAEVLDRLGQIVKDWVKQLTRLRGYTDQMVEDANAVLFTFGSYRLGVHGPGTDIDTLCIGPSYVSREEDFFFILHNILADMEEVTELQPVPDAHVPVMKFKFDGISIDLLYASISLLVVPEDLDISDLSVLYNIDEPTVRSLNGCRVADQILKLVPNVEHFCTTLRCLKFWAKRRGVYSNVTGFLGGVNWALLVARVCQLYPNAVPSMLVSRFFRVYTQWRWPNPVMLCAIEEDELGFSVWDPRKNPRDRTHHMPIITPAYPCMNSSYNVSISTLRVMMEQFQYGNKICEGIELSNAQWGALFEPYLFFESYKNYLQVDIVAVDIDDLRAWKGWVESRLRQLTLMIERDTFGKLQCHPYPHEYVDTSKQCSHCAFFMGLQRKQGEIIQEGQQFDIRGTVDEFRHSINMYMFWKPGMEIYVSHVRRKQIPSYVFPEGYKRSRPQRPVNQQQGDEASAKRLTISPQRQDSVSPEIISHRWMKADEKGNIEPDKSDKPIPCTGNAEAGSVSNSSVVTSITSEVSSSGDVGFESVGGSSDGNTGSVEGSNILGISQGDSCEADSELLLENGCVNAKEGFQDGLHEELEPNAALGIVLKSRGGVDSEPVPKSVLSRLSLTSTA*

>:GSVIVT01034960001

MAHAHRSQNSVALVHSQPLIHPQPPVVNAPVGFVPRPVGVINPSFGPVPPFDPASLPQPGFVLNPAVLVRMEHRRSISLLQFMSNEGLIPSPEEELKRKNVIEKLKEIVLTWVKRVAWQRQRPKQQIAVTSATILTYGSYGLGVHGPESDIDALCVGPFFASMADDFFIVLRNMLESRPEVSEIHCVKDAKVPLMRFKFDGISVDLPYAQLKLLYVPENLNVLNPYFLRNIDETSWKSLSGVRANECILQLVPNVENFQSILRCIKLWAKRRGVYGNLFGYFGGVHLAILAAFVCQKNPHANLNVLMSSFFKTFSGWPWPTPVALEDGRLPTGGTRETRALMPIQLPCSPYGYCHSNITKSTFYRITTELTLGHALTRDLLRLDFDWNDIFEPFCYSKKYSRFIKIYLSSSNQDELGDWVGWVKSRFRFLLAKVEEVQGLCDPNPTEFIDPDAGGPNVVFFWGVQPGRINFSDIDVVEDDFMQNINNGGYQGPPGKMNLSVIPTSQLPGYAQLDTGSRNRTKACWRMFNYHQPRVPVFSQHLPLYFVGYAATDKDAKRLDAWS*

>Ppa:Pp1s3_426V6.1

MEGSRYLGVTEPISTADPTEVDYVNTKQLEECVKGLALQGSREEEVRREEVLGRLDELVNVWVKSMSRKKGLNDEYVREARCKIFTFGSYRLGVHGPGADIDTLCVGPCYVTREEDFFVELHDLLQKTEGVTELHTVPDAHVPVMSFEFNGIPIDLLYARLPLWVIPEELDILQDTILQNVDEQSVRSLNGCRVTDRILRLVPNMEHFRTTLRYVKLWAKRRGVYSNVIGFLGGVNWALLVARICQLYPNAVPSVLLSRFFRVYKQWRWPNPVMLCAIEEGPLGLPVWDPRRNPRDRSHLMPIITPTYPCQNSSFNVSNSTLRVMTEEFKRGDSICDSLDSKVADWSKLFEPYPFFESYKNYLQIDISAGDEEDLRIWKGWVESRLRQLILKVEKDTFGALQCHPHPSAFHDTSKRVQVCSFFVALQRKQGAPPSSTPFDMCHTIAEFKHSVNQYLLWKPTMKIHVSHVRPKQIPTYVFPNGIRPVRPPRPTIHKPGTNPDNVPTSQSPEASTRSVVTPPSEEPELPIPGTKRGAESLGVLGKRVRVELRSES*

>Ppa:Pp1s475_11V6.1

MDGDRYLGVTEPISTADPTEVDYANTKQLEEFVKGLALQGSREEEVRREEVLGLLDELVNVWVKMVSRMKGLNDEYVREARCKIFTFGSYRLGVHGPGADIDTLCVGPCYVTREEDFFVELHDLLQKTDGVTELHTVPDAHVPVMSFEFNGIPIDLLYARLPLWVIPEELDILQDSILQNVDEQSVRSLNGCRVTDRILRLVPNIEHFRTTLRYVKLWAKRRGVYSNVIGFLGGVNWALLVARICQLYPNAVPSVLLSRFFRVYKQWRWPNPVMLCAIEEGSLGLPIWDPRKNPRDRSHLMPIITPTYPCQNSSFNVSNSTLRVMTEEFKRGDGVCDSLDSKVADWSKLFEPYPFFESYKNYLQIEITAGDEEDLRIWKGWVESRLRQLILKVEKDTFGMLQCHPHPNAFHDTSKKVQVCSFFVALQRKQGAQHSSTPFDMCHTIAEFKHSVNQYLLWKPTMKIGVSHVRPKQIPTYVFPNGIRPVRPPRPPLLSGRPGSGPNMDNVPTSLSPEASTRAVVTPPSDGKVSDGKESKEPELSIPDTKRGAESLGVLGGPHSKRVRVELRSES*

>Ptr:POPTR_0006s25200

MVGSQSPNGTAAKRYGIMKPISVAGPTEPDLHRNAELEKQRRGYEERRCSGPYRSDWVKRLTRQRGYTDQMVEEANAVIFTFGSYRLGVHGPGADIDTLCVGPSYVNRELAEMEEVTELQPVPDAHVPDLDISNGSVLYEVDEQTVRSLNGCRVADQILKLVPNVEYASLSVLQSLYTMALAESCDAMLNRRRCTWVSCVGSSQKSSRPKIELNKEQWSALFEPYLFFEAYKNYLQVDIVAADAVDLLAWKGWVESRLRQLTLKIERDTDGMLQCHPYPNEYIDPSKQCAHCAFFMGLQRKEGVTGQEGQQFDIRGTVDEFRQDINMYLPWKPGMDIYVSHVRRRQLPGFVFPDGYKRSRPSRHVNQQTNRTSEDVARSQSGSAERHVKRKNDCEMADLKPVKPEKRASTSPQRLQSVSPSSSAGRSGMTSLASSCEGVILGCSTIGDIVSNCEDVASNSEVRSTSWQLESEKSDLGDARQLGVTVYQESPSNRQTSMDVHDSPIVRNELEPADHMNGSEPMGLMFDRITKQELVSSHEVPNFETGEKHEVGVNENIEDLGSNFLENGSSRKLMNWVGGASRGMEVDQELVKPCSQTAVVEFAESVISSHSGSQNLNYEGNVCAVDADSLLESGCLNVSGAEYYCRTACQKN*

>Ptr:POPTR_0018s04870

MVGSQSSNGTAAKRYGITKPISVAGPTEPDLHRNAELEKFLVDSGLNESKDETIKREEVLGRIDQIVKDWVKQLTRQRGYTDQMVEEANAVIFTFGSYRLGVHGPGADIDTLCVGPSYVNREEDFFITLHDKLAETEEVTELQPVPDAHVPVMKFKFQGISIDLLYASISLLVVPEDLDISNGSVLYEVDEQTVRSLNGCRVADQILKLVPNVEHFRTTLRCLKFWAKRRGVYSNVTGFLGGVNWALLVARVCQLYPNAIPSMLVSRFFRVYTQWRWPNPVMLCSIEEDDLGFPVWDPRKNPRDRFHLMPIITPAYPCMNSSYNVSTSTLRVMTEQFQSGNRICEEIELNKAQWSALFEPYLFFEAYKNYLQVDIVAAVAADLLAWKGWVESRLRQLTLKIERDTNGMLQCHPYPNEYIDASKQCPHCAFFMGLQRKEGVTGQEGQQFDIRGTVDEFRQEINMYMFWKPGMEIYVSHVRRRQLPGFVFPDGYKRSRSSRHINQHTSKTGGMEIYVSHACYSPVRPQSVSPSSSVSRSGVAPVKRKNDCEMEDLKPEKQACYSPVRPQSVSPSSSVSRSGVTSLASSWEGVKLGCSTIRDIGSNCKDVASNSEVRSSSGQLESEKDGLGDSMQLGETVYQDSPLNRQISMDVHDSPIVRNELEPANHMNGIEPMESMVNTITKQEMLSPQEIPNFETGEKHETGVNDKIAGLGSNLMENGSSRKLLNWVAGTSQAMEVDQELVKPCCQTAVVEYAESVIRSHSGTQNLNCEGNVCAVDADVVLESGCLNMSRVLPKGLPEELEPKTAIGKVVNSQDGARSESLQKPMIRLSLKSTA*

>Ptr:POPTR_0015s04100

MGSPGLINRNNGQQQQQRLGITEPISLGGPTEYDVTKTRELEKFLQDAGLYESQEEAVSREEVLGRLDQIVKNWVKVISRAKRLNEQLVQEANAKIFTFGSYRLGVHGPGADIDTLCVGPRHATREEDFFGELHRMLSEMPEVTELHPVPDAHVPVMRFKFKGVSIDLLYAKLSLWVIPEDLDVSQDSMLHNADEQTVRSLNGCRVTDQILRLVPNIQAMQNFRTTLRCMRFWAKRRGVYSNVSGFLGGINWALLVARICQLFPNALPNMLVSRFFRVYTQWRWPNPVMLCAIEEGSLGLSVWDPRRNPKDRYHLMPIITPAYPSMNSSYNVSSSTLRIMTEEFQRGNEICEAMEVSKAEWDTLFEPFSFFEAYKNYLQIDISAENEDDLRQWKGWVESRLRQLTLKIERHTYNMLQCHPHPGEFSDKSRPLHCSYFMGLQRKQGVPVNEGEQFDIRITVDEFKNSVNMYTLWKPGMEIRVTHVKKRNIPNFVFPSGVRPSRPSKATWDGRRSSEAKVANNSSADKIEGKGVLDGSDEGKKRKRIDEDTENNLRNPKGYAAMPPSGGEVHEGSPPVGNVSSCSTQSDLVITNSLGELKGEKADNNETESLSNSQNLAGIFAQNGELDGILRCNLPDKGLPANNDTSSSKEAEKLAIDKIMSGPYVAHQALPQELDELEDDFVYTNQGKGSEWAAKGSPVESSLSNTAVEQTNESIAAVACSNGAGPSAYLYPNGGSEELEPAELMAPLFNGISSAPPVAQPKPLIRLNFTSLGKAAGKST*

>Ptr:POPTR_0008s10210

MEDERSLSLLKLMVNEGLFPSPEEDEKRKIIVEKLKTIVVAWAKKVAWQRCLPKQQIAATSATILTYGSYGLGFHDPESDIDALCVGPFFATIAEDFFIVLHNILKSRPEISEIHCVKDSKVPLMRFTFDGISVDLPYAQLKVLNVPENVDILNLSLLTNIDETSWKSLSGVRANQRILLLVPNLMNFQSMLRCLKLWAKRRGVYGNLNGFLGGVHLAVLAAFVCQNQPNASVIALISNFFSTYAMWPWPTPVMLQDGMSSNVEDVIETRFYMPIRLPCSPYEYCHSNVTKSTFTKIRAEFLRGHSMTRDLLKLKLDSDVGRIFEPFPYSTNYTRFVKIYLSAPDQDELGDWVGWVKSHFRCLLLKLEAVQGFCDPNPMEYVDMDASEPNVVFYWGLNRSRCNFVYIEPVEEDFSRSIYCGYYGIRGKMELSIVQASELPKNARFDSGNGKKMKACWKMLDYNQRRTPAYSQHLPSYFVGYVESNGDTEYPSTGG*

>Ath:AT2G25850

MVSTQQRTDDDSSQPVKASLKSYGITEPLSIAGPSAADVKRNLELEKFLVDEGLYESKEETMRREEVVVRIDQIVKHWVKQLTRQRGYTDQMVEDANAVIFTFGSYRLGVHGPMADIDTLCVGPSYVNREEDFFIFFRDILAEMEEVTELQPVTDAHVPVMKFKFQGISIDLLYASISLLVIPQDLDISNSSVLCDVDEQTVRSLNGCRVADQILKLVPNSEHFRTTLRCLKYWAKKRGVYSNVTGFLGGVNWALLVARLCQFYPNAIPSMLVSRFFRVYTQWRWPNPVMLCAIEEDDLSFPVWDPRKNHRDRYHLMPIITPAYPCMNSSYNVSQSTLRVMTEQFQFGNTICQEIELNKQHWSSLFQQYMFFEAYKNYLQVDVLAADAEDLLAWKGWVESRFRQLTLKIERDTNGMLMCHPQPNEYVDTSKQFRHCAFFMGLQRADGFGGQECQQFDIRGTVDEFRQEVNMYMFWRPGMDVHVSHVRRRQLPSFVFPNGYKRSRQSRHQSQQCREPGDEGVGSLSDSVERYAKRKNDDEIMNSRPEKREKRASCSLHTLDAASPDSSGITTSGTPQIGIVPGPRAECLVTGDLVCNVTSLPNVEVEAEKFISKITELRKFSQYEHTSGSEQILEVDSRALVQSYHDLAEPVAKHVRPDLSALLACEGGQNKEIGHDMGSESINDTDTQHLPRRLNVNEDVDEVEREAKLGEIAGGVLWNGHCGRNLDHEGFVTPANLDSAVENRNLHSDGLFKSGLPEELQSNSLLSGTGKLDDGARSESLQNEMMRHVFLQPIIGLCKS*

>Ath:AT4G32850

MMVGTQNLGGSLPPLNSPKSYGITKPLSLAGPSSADIKRNVELEKYLVDEGLYESKDDTMRREEVLGRIDQIVKHWVKQLTQQRGYTDQMVEDANAVIFTFGSYRLGVHGPGADIDTLCVGPSYVNREEDFFIILHDILAEMEEVTELHPVPDAHVPVMKFKFQGIPIDLLYASISLLVVPQDLDISSSSVLCEVDEPTVRSLNGCRVADQILKLVPNFEHFRTTLRCLKYWAKKRGVYSNVTGFLGGVNWALLVARVCQLYPNAIPSMLVSRFFRVYTQWRWPNPVMLCAIEEDELGFPVWDRRKNHRDRYHLMPIITPAYPCMNSSYNVSQSTLRVMTEQFQFGNNILQEIELNKQHWSSLFEQYMFFEAYKNYLQVDIVAADAEDLLAWKGWVESRFRQLTLKIERDTNGMLMCHPQPNEYVDTARQFLHCAFFMGLQRAEGVGGQECQQFDIRGTVDEFRQEVNMYMFWKPGMDVFVSHVRRRQLPPFVFPNGYRRPRQSRHQNLPGGKSGEDGSVSHSGSVVERHAKRKNDSEMMDVRPEKPEKRASLSPQSLDIVSPENSAITTGWTPPVCNLRRPPSEEIEADNLNTECTELTDLARNECNSGSEQVLEVDSMAVVQECSDPAEPLGKCVTPDSVDVVACVSGQEENLDRNLRSVSISGTDSPLLPSRSCGQNRDYEGFGFPAANSDPMGKKNLYSQSGMSEDLQSNSLVSGMEKSEDRARSESFQKSQIRHAIDVKKTGDFNSNCCVGQTTESRSFW*

>Ath:AT1G17980

MASVQQNGQRFGVSEPISMGGPTEFDVIKTRELEKHLQDVGLYESKEEAVRREEVLGILDQIVKTWIKTISRAKGLNDQLLHEANAKIFTFGSYRLGVHGPGADIDTLCVGPRHATREGDFFGELQRMLSEMPEVTELHPVPDAHVPLMGFKLNGVSIDLLYAQLPLWVIPEDLDLSQDSILQNADEQTVRSLNGCRVTDQILRLVPNIQNFRTTLRCMRFWAKRRGVYSNVSGFLGGINWALLVARICQLYPNALPNILVSRFFRVFYQWNWPNAIFLCSPDEGSLGLQVWDPRINPKDRLHIMPIITPAYPCMNSSYNVSESTLRIMKGEFQRGNEICEAMESNKADWDTLFEPFAFFEAYKNYLQIDISAANVDDLRKWKGWVESRLRQLTLKIERHFKMLHCHPHPHDFQDTSRPLHCSYFMGLQRKQGVPAAEGEQFDIRRTVEEFKHTVNAYTLWIPGMEISVGHIKRRSLPNFVFPGGVRPSHTSKGTWDSNRRSEHRNSSTSSAPAATTTTTEMSSESKAGSNSPVDGKKRKWGDSETLTDQPRNSKHIAVSVPVENCEGGSPNPSVGSICSSPMKDYCTNGKSEPISKDPPENVVAFSKDPPESLPIEKIATPQAHETEELEESFDFGNQVIEQISHKVAVLSATATIPPFEATSNGSPFPYEAVEELEVLPTRQPDAAHRPSVQQRKPIIKLSFTSLGKTNGK*

>Ath:AT3G06560

MKKGGGRNKGFPQDDESSISLRQLMVNEGLIPSLEDEVKRRGVINQLRKIVVRWVKNVAWQHRLPQNQIDATNATILPYGSYGLGVYGSESDIDALCIGPFFASIAEDFFISLRDMLKSRREVSELHCVKDAKVPLIRFKFDGILVDLPYAQLRVLSIPNNVDVLNPFFLRDIDETSWKILSGVRANKCILQLVPSLELFQSLLRCVKLWAKRRGVYGNLNGFLGGVHMAILAAFVCGYQPNATLSSLLANFFYTFAHWQWPTPVVLLEDTYPSTGAPPGLMPIQLPCGSHQYCNSTITRSTFYKIVAEFLLGHNLTKDYLKLNFSWKDLFELYPYANTYTWFTKIHLSAANQEDLSDWVGWVKSRFRCLLIKIEEVYGICDPNPTEYVETYTKQPNIVFYWGLQLRTINVSDIESVKIDFLKNVNSGSFRGTVGRIQLTLVKASQLPKNGECGSNNRSKKVTKTCWRIREDKQCNNVPVYSKHLPGYVVGYQKMVNREADGMEVKC*

>Cre:Cre10.g433750.t1.2

MAVADPNDVYLLRPLNNSLPSAEDKRHSAELEQFLRDAGLYEPDEDAYLRQEVLGLFYELTQTWVKGVCRKKNLNVEDARAHVYTFGSYRLGVHGPGADMDTLVVGPRYVLRDSDFFGSEKHCLEYMLSQTPDITDIQPVPDAFVPMIGIKYKGVQIDILYASLAMQTLPEQLDLSNHAVLRGCDEPTVRALNGCRVTDTMLKLVPRQEVFRTALRAVKHWASLRGISSNVTGYLGGVNLAIMVAKICQLYPRAEASTVLLKFFILLKAWPWPRAIHLRIPEEHSLGLPVWDPRPGTRDSLALMPVITPAYPAMNSLYNVQRSTLEVMTEEFAAAADVCTSFLHCPPGKPIEWSRLFTPVPFFTQHSFYIQLEVSADSEGDLVLWDGWVSSRIRRLVRNLEDHVRVRPYPKAQKPPVEPAKEADPDGNDAKPVTEEASKDVVKAEPKDADKDPDATAPNRPRLCYYIGVDKRAAAALTAQQLLPNGQVAQLPAHALTGPAGAPAGKVDLKQPCIIFVNEVSAWPAKRPGMALKVNVLKRALLPSWLPGVPQRPQTQALPAPPPATAGGKAGIKRAPSEDPAHQDASAAPAAALPAQQPAKRPRVAQAAPAAAGPKRAAVQDASPSTAAVAAPAGAPPAKRSKVAQATSRSQAQPPALTSEPQAGATTAAAPTVPAPVQAACEVAAAAAEPPSGAGPVSAGAGGESGVAVASGSAAPQSADATSLSGAATDDATADTAAAAGGGGVGLSPEAAQQLAERASQADERAAVQESQLHNTGRDMGDWLGVDHGVALTGVQVRQRRRWWQWRWQWWLGG*

>Bdi:Bradi1g17160

MATYLPIGTPSSSSSSPAAAAGAPEPQTLPFFLSPLPLPPPPLPAGHRVLSSLPPPPPRWPIAVRVPPAFLFEADFRRSHSLVQFLENEGAVPTVEEDKTREQVIRKLKEIVMDWAKVVAYEQRVPPRRVTATVLTYGSYTLGAHGPESDIDALCVGPCIATLQAINTSSPQFLRRLDSRSWRSLSGVRVNEQIVQLVPNAKKFQVLLRSIKLWARRRGLHCHLLGFFAGIHLAILAAYICQRYPNATANGLFTMFFEIFAHWDWQIPVNLHGQPTNCKRSDGYYMPIVMPCTPPELCMSNMTKCTFKKIREELMRGYALTKDLWRHDFEWTWLFAPFPYATRYQQFLRIALCSPTSEELRDWTGWVKSRFRQLILKLERNGLECDPYSSEEVDHRVIKPNIMYHWGLISEANTYLDISSLREDFMKDIINDVYGKVKCRRSELTLSVVGSSQLPKSMYSDSAYLGYMPQYMVGYQPAPDCWSAAG*

>Bdi:Bradi1g37940

MTTSNKPLSLAGPVDADIQRTAELNKFLVEAGLYESAHESARREEVLGELDKIVKDWVKQLTSQRGYTDQMVEDANAVLFTFGSYRLGVHGPGADVDTLCVGPSYVNREEDFFIVLHDILAQTEEVTELQPVPDAHVPVMKFKFHGISIDLLYASVSLLVVPSDLDISQESVLYDIDEATVRSLNGCRVADQILRLVPNIENFRTTLRCLKHWAKRRGVYSNVTGFLGGVNWALLVARVCQLYPNAVPSMLVSRFFRVFTQWRWPNPVMLCAIEEEELGFPVWDPRKNPRDRTHHMPIITPAYPCMNSSYNVSTSTLRVMIEQFQFGNKICQEIELNKANWPALFESFQFFEAYKNYLQVDIIAEDDEDLRLWKGWVESRLRQLTLKIERDTYGKLQCHPYPYEYADPSRQCAHCAFFMGLSRNVGVKIEEGQQFDIRGTVDEFRHDINMYMFWKPGMELAVSHVRRKEIPAYVFPEGYRRPRPQRHVSHQQQSDKNDTENGNMTGSPDGQLKRKHDSAGTDDTEPCRSVKRASVSPVHPKHDSPGTDDTEPCRSVKRASVSPVHPTTSSPSSGNISDEATCNNQKKMSSNASGGSQNSHGSGNLEQANCSGSSPASEKSTDSIASDSKCVIGETVCSGDVTNNLVTCISPVNNSTPTVAVCTTLKRVAEKVVSELVGSESLGGNNPELLEIAEKDMGSVLVENVHFGGNGVTQSGLHEELEPNNGIEVLSKAHAGVNSDGSQKPSMRVSLTSTA*

>Bdi:Bradi1g64220

MVLDPVVGGSASRKVEPLTLAPPRARGPPPPPPIPPTPRMYLGGPPPLPPPGTIPPRPIILRLDPATTVHMDVVRSVSLLKFIAGAGVVPSQEEEQRREEVVRELDKIVMDWAKQVAYDQRDKHWITTGTVLTFGSYALGAYGPESDIDAVCVGPCVASLQHHFFVVLRQMLEERPEVSDLHSIESARVPLMRFKFNGVSVDFPYVQLPVINAAEAIRAFDPRLLEKVDGASWRCLSGVRVNRQIMQLVPNMKRFQVLLRCLKLWARKRGIYCHLVGYFAGIHLAVLGAYVCRRHPNASVNTLFSMFFDIFSHWPWPLPVALHDQAPLWGPDGCSLMPIVMPCFPPEFCASSITKSTFNKIKEELRRGFALTKDIRNVDIDWTWIFAPYPYTVKYEHFLHMVLSAPTTEELRDWVGWVKSRFRNLILKLESLDVDCDPDPSEKVNHTIAAPNVVFFWGLIYRSTKISASSLKNDFMKSVINNIYGKEKCARSDITMSIIETSQLPKSISGDSVYKKLQNLPPSMLGYQPMKQGCPVV*

>Bdi:Bradi5g19810

MESRPLPPPPQQNQPISLLGPTPADLESTSRMEKHLRDAGLYESPEELAAREDVLRDLRGIVDRWVKRLTAQRGYPDGMVSQATALVLPFGSYRLGFHGRGSDIDALVVGPSYVDRDHDFFGVLGGVLSEMTDDAVTELQPVPGAHVPVIKMRFRGVQVDLLYASVCLAVVPRDLDLRDRSVLRGMDLATVRSVNGVRVADEILRLVPDAGAFRTTLRCVKLWAKARGVYSNVMGFPGGVAWAILVAFVCQLYHNAAPSVLVSRFFKVLAPWKWPNPVKLRDIEHDDDELRLPVWDPRRNPRDRTHLMPVVTPAYPCMNSCYNVSHATLRTITQQLQIGNAVCQKIVASGGEWGALFEPFHFFGWTLLVMRVETATAGMLLCHPNPQAYAAKPTELQHTTSFFVDLSKPQPQEKEQQQPQVQFDLRETAEEFKREVYMYVSWRPGMEVEVSHNRRKDLPSYVLEQILPAGHLKRKRPQEDDEPSPSSSPASDD*

>Bdi:Bradi5g19750

MESSRPLQQQQPISLLGPTPADLESTSRLEKHLRDAGLYESPEELAAREDVLCELRAIVDRWVKRLTAQRGYPDGMVSQATAPVLPFGSYRLGVHGRGSDIDALVVGPSYVDRDHDFFGVLGGVLSEMTDDAVTELQPVPGAHVPVIKMRFRGVQVDLLFASVCLAVVPEDLDLRDRSVLRGMDLATVRSVNGVRVADEILRLVPDAGAFRTTLRCVKLWAKARGVYSNVMGFPGGVAWAILVAFVCQLYPNAAPSVLVSRFFKVLAPWKWPNPVKLRDIEHDDDDLRLLVWDPRRNPRDRTHLMPVVTPAYPCMNSCYNVSHATLRTITQQLQIGNAVCQKIVASGAGWGALFEPFHFFKEYKSYLRVDVKVAGGGGEGDLREWKGWVESRLRQLVMRVETATAGMLLCHPNPQAYAAKPTDLQRTTSFFVGLSKPQPQPQEKEQQQPQVPFDLRETAEEFKRKVYMYVSWRPGMEVEVSHNRRKDLPSYVLEQILPAGHLKRKRPQDDDPSPSSSPASDDSESSSRHTKRVEAASTTGSLSDGTIEQRLV*

>Bdi:Bradi3g08637

MAGSVATGRVTPQSSLKRSSGMDPPLSLAGPTMVDLQKTSELEKFLVEAGLYEGEEQSAKREEVLREIGRIVKEWVKQLTSQKGYADQMVEKANAVLFTFGSYRLGVHGPEADIDIVCVGPSYVNREDDFFVTLHDILAQMEEVTQLQPVPDAHVPVMKFKFQGIPIDLLYARVSLSVIPPDFDISQGSVICDVDEATVRSLNGCRVADQILRLVPNTENFRTTLRCLRYWAKRRGVYSNVTGLLGGVNWAILVARVCQLYPNAVPSMLVSRFFRVFTQWQWPNPVMLRAIENDDLGFSAWDPRKNPRDRSHVMPIITPVNPRMNSSYNVSTSTLRVIMEQFKLGNKILQEIELNKASWTALFEPFQFFEAYTKYLVVDIVADDDDDLRLWKGWVESRLRQLTLKIERDTKGMLQCHPYPLEYANPLAQCAHSSFYMGLSRKEGMKMHGQKFDIRGTVDEFMHEMGLYTSWKSGMDLAVTHVHKKQIPYYVFEQGYKKHCPPMLPNEQEQSDRNDNEDGTLTGSLEGQLKRKHDIDEAGHIESCKSVKRSSVSPGYKETPPEYGSNISKIVCENPVKSVSSALCSGLQNSLLHGDVSLEPSNCSSSPHGSEESTASGSSCAAVETVGLVDETVDPESSMPCIVNGTVQTMAVHTPIKCVAEKDEMKFEGINSLANRAEFLDKPEMLTGNILPENLH*

>Bdi:Bradi3g03930

MAKSNTGYLGVSEPISLSGPTEKDVMQTTEVEKFLADAGLYESQEEAVSREEVLGKLDQTVKTWIKKATRASGYGEQFVQEANAKIFTFGSYRLGVHGPGADIDTLCVGPRHATRNDYFFRCLHDMLAEMPEVSELHPVPDAHVPVLGFKLCGVSIDLLYANLAHVVIPEDLDLSQDSILHNVDEQAVRSLNGCRVTDQILRLVPNIPSFRTTLRFMRYWGKRRGVYSNVMGFLGGINWAILVARICQLYPNASPSMLISRFFRVYSQWKWPNPVTLCHIEEGPLGLPVWDPRRNFRDRGHQMPIITPAYPCMNSSYNVSVSTRYVMVQEFTRGYEICQAIDENRATWDDLFEPYPFFELYRNYLEVVISARNEDDLRNWKGWVESRLRTLVLKFERYTHEMLLAHPHPRDFSDGSRPLHSFYFMGLWRKQTVQPQEAEQFDIRGIVNEFKSSVCAYAHRGEGMDIEVSHVKRKDIPLFVFPGGVRPPRSSRTAGRNGHAVSRNDVSADGQTGVRNNVSADGQVGTRNDVSADGQVGNPLVTEGWSDPPTQGPSAGYQLPENTSLLANLASRLSNNETQHILNGDSNLHTESVEHEHPVRFLGSTSAPVDNAVLDVIKPPNSIPSTSSNGGPTNGLGISFNSSHKESKGIHVNNLVTSSPTTTDGLDELVWPQAKSDKIHANTAHVSPLEGCSGTSLGQAVNLSSHGNNHVKRKAEEELEPLELAAPSVRAAAPSTSTVKRKPLRLRLSTVPQPKPAEGSS*

>Osa:LOC_Os04g49870

MASQSPQSRGVAEPISLVGPTPADLESTARLERLLREEGLYESAEETAAREEVLRGLRGVVDRWVKRLTRQRGYPDGMADRATALVLPFGSYRLGVHGRGSDIDALVVGPSYVDCDRDFFGALATALAETAAVAELQPVPGAHVPVIKMRFHGVQVDLVYAGVCLPVVPGDLDLSGRSVLRGLDLATARSLNGVRVADEILRLVPDATAFRTTLRCVKHWAKARGVYSNVAGFLGGVGWAILVACVCLLYPNASPSMLLPRFFRVFARWKWPSPVMLRAIEHDDGELGLSLPVWDPRRNPRDKIHLMPIVTPAYPCMNSGYNVSHATLRVITEQLAVGDAVCQEIVKAGSGGGGWDKLFQPFNFFGAYKSYLQVDVTVTGGEEDDLREWKGWVESRLRLLSARVEADTSGMLLCHLHPQPYAAEPHNEPRRRRRTSSFFVGLSKPPAQPQQQQHQLFDLRATTEGFKEEVYMYDYWRPGMEVAVAHVRRKDLPSYVLRQLLRSPGRHDQLKRKRADDDPSSSPAASDHSASSSSSRDAKRPAAAPGRIGSSFEKKT*

>Osa:LOC_Os07g48890

MASARATDPLASRDPPTLPSYLPPPPPPPLPSPSPHHRLLPAPMAPILLHLHPAFLAQMDSRRTTSLLQFLKDEGGIPSPEADKKREQVIRKLNKIVMDWAKVVAYEQRVPPRRATATVLTYGSYTLGAHGPESDIDALCVGPCIATLQYHFFIVLRQILEDRPEVSELQTVESAKVPLMRFRFSGISVDFTYAQLPVIDASEAIITSNPHLLQKLDSLSWRSLSGVRVNEQIVQLVPNAQKFQILLRCIKLWAKRRGIHCHLLGFFAGIHLAILAAYVCQRYPYGTINGLFTIFFDIFAHWNWQIPVSLHGQPTNCRRPDGSFMPILLPCTPPEFCTSNMTKGTFKKIREELMRGYALTKEPWRHDFEWVWLFAPFPYATKYEEFLRIALCAPTSEELRDWAGWVKSRFNLILKLESIGVECDPDSTEEVDHTVFEPSIVCHWGLIYKTSTHIDISSLGEDFMKDVINDVYGKVKGTHSKLTMSIVRSSQLPKSLYSHSVYTPYIPQYMLGYQTPTDYSGAAG*

>Osa:LOC_Os03g19920

MSRSSRGGRQSASSATRMASRARPGFPVAPPPPMGPPPPPPMPPVPVMYLRGVPPPPPWLPQHLIICGLDPAAAERTDAFRSKSLLNFISRTGVLPSPEEELKRQVVVRELDKIVMGWAKRVAYDQREQYWNTTATVLTFGSYALGAYGPESDIDAVCVGPCIASLQHHFFIVLRQMLEERPEVSDLHSIENAKVPLMRFKFNGMLVDFPYVQLPVINAAEAIHAFDPRLLAAVNEPSWRCLSGVRVNRQIMQLLPNIKKFQILLRCLKLWARKRGLHCHLLGFFAGIHLAILAAFVCIMHPHATLSSLFNSFFDIFSHWHWPLPVSLLDQPTPWRPHCCSFMPIVMPCSPPEFCASSITRSTFNKIKEELQRGFALTKGDRNGDINWTELFAPFPYTVRYKHFLRIVLSAPVAEELRDWVGWVKSRFRNLLLKLESIGVDCDPDPSEQADHSMIEPNVVFFWGLMYRTSTNICIDSVKEDFMKSVTNDIYGKEKCTHSDITMSIVWPTHLPKCVYAHSVYSQNRQNPRQFMMGNQLMNQDCNAVR*

>Osa:LOC_Os02g13400

MAGSFVAVRGKPRSSPKRSGGGGGADPPLSLAMPTVADLHKTAELEKFLVEAGLYEGEEESAKREEVLREIDQIVKEWVKKVTIQKGYSEQMVKEANAVLFTFGSYRLGVHGPGADIDALCIGPSYVKREEEFFVMLYGALSEMEEVTELQPVPDAHVPVMKFKFRGLPIDLLYASVSLPVIPPDFDISQGSVLCDVDEATVRSLNGCRVADQILRLVPNAEIFRKTLRCLKYWAQRRGVYSNVTGLLGGVSWALLVARVCQLYPNAVPSMLVSRFFRVFTQWQWPNPVMLCAIENDDNLGFAVWDPRKNPRDRSHVMPIITPAYPCMNSSYNVSTSTLRVIMEQFQFGNKICQEIELNKASWSSLFEPFQFFEAYTRYLVVDIVADDDDDLRLWKGWIESRLRQLTLKIERDTKGMLQCHPNPCEYADPSIQCAHCAFYMGLSRKEGMKIRGQKFDIRGTVDEFMHEIGMYTQWKSGMDLAVTHVRKKEIPLYVFEQGCQKTRPPTPICAEQQDQSDKNDSEVCTTTASLVGQLKRKYHSVGGADVDSFKSVRRASVSPACEEASIQLHDDANFGLTNCSTSPHGSEGSTVSGNSCAAVGTIGLVDETS*

>Osa:LOC_Os06g36360

MAACNAAAAAAVAEQPQKQYGITKPISLAEPAEVDLQKTAELEKFLVEAGLYESPEESARREEVLGELDKIVKDWVKQLTSQRGYTDQMVEEANAVLFTFGSYRLGVHGPGADIDTLCVGPSYVNREEDFFIVLHDILAQTEEVTELQPVPDAHVPVMKFKFHGISIDLLYASVSLLVVPPDLDISQGSVLYDVDEVTVRSLNGCRVADQILRLVPNVENFRTTLRCLKYWAKKRGVYSNVTGFLGGVNWALLVARVCQLYPNAVPSMLVSRFFRVFTQWQWPNPVMLCAIEEDELGFPVWDPRKYHRDRSHHMPIITPAYPCMNSSYNVSTSTLRVMMEQFQFGNKICQEIDISKANWSALFEPFQFFEAYKNYLQVDIIAEDGEDLRLWKGWVESRLRQLTLKIERDTYGMLQCHPYPHEYADPSRQCAHCAFFMGLSRKEGAKIQEGQQFDIRGTVDEFRHDIGMYGYWRPGMELAVSHVRRKQIPSYVFPEGYKRPRPSRHINHPQQSNKNDVEDGTANRSPDGQPKRKHDTAGVYDSEPGRSVKRASISPSISPVHQKTSSPPSGNIADASGASGGSPVSLANGNLEQANCLNSPLASEKSLDSVTSGSKCVGVEAVCPSDATKEHDNCGSNMKNCTTTTVAVSLKRVAEKVVSELVGSESLGGNKSGELLERAEDMGSALVENVHFGGNGVVQTGLPEELEV*

>Osa:LOC_Os06g21470

MAKSNNGNGYLGVTEPISLSGPTEKDVVRTQEVEKCLADAGLYESQEEAVSREEVLGKLDQIVKAWIKKATRASGFGDQFVQEANAKIFTFGSYRLGVHGPGADIDTLCVGPRHATRTEYFFQALYDMLVDMPEVTELHPVPDAHVPVLKFKLNGVSIDLLYANLTHVVIPEDLDLSHDSILHNVDEQTVRSLNGCRVTDKILRLVPNILTFRTTLRFMRFWAKRRGVYSNVIGFLGGINWALLVARICQLYPNASPSMLISRFFKVYSKWKWPNPVMLCHIEEGSLGLLVWDPRRNFRDRGHHMPIITPAYPSMNSSYNVSISTRHVMVQEFTRASDICQAIDERKADWDALFEPYPFFESYRNYLKIEITARNEDDLRNWKGWVESRLRTLVLKIERFTREMLLSHPNPRDFIDSSRPLHCFYFMGLWKKQISQAQEAEQYDIRAIVNEFKSNIHAYQHWREGMEIEVSHVKRKDIPSFVFPGGIRPSRPSRTVGKEARAVSRSNISANVQERNVPSMAQPMPYKSSEVNKIPSDPHGGYQSQERNNAVVSSLPCEETGHMFNGYANLHTESVELEHLRSYKGSTSVPENHVVHDLVKPPESMPPNSIHVYPSPTNGLGHLLDSSCKKPADIIVNKTTNFSSAVLAVPDELDELDSHQVKVNQKDLTAVDQGLSLEHKVGSNGGKAGTTGSPDNNHLKRKAEEELEPLELAAPLVRPPAPTSMTQRRPLRLRLSTVVQPKPAEGTS*

>gi|16306568|ref|NP_075045.2| poly(A) polymerase gamma [Homo sapiens]

MKEMSANTVLDSQRQQKHYGITSPISLASPKEIDHIYTQKLIDAMKPFGVFEDEEELNHRLVVLGKLNNLVKEWISDVSESKNLPPSVVATVGGKIFTFGSYRLGVHTKGADIDALCVAPRHVERSDFFQSFFEKLKHQDGIRNLRAVEDAFVPVIKFEFDGIEIDLVFARLAIQTISDNLDLRDDSRLRSLDIRCIRSLNGCRVTDEILHLVPNKETFRLTLRAVKLWAKRRGIYSNMLGFLGGVSWAMLVARTCQLYPNAAASTLVHKFFLVFSKWEWPNPVLLKQPEESNLNLPVWDPRVNPSDRYHLMPIITPAYPQQNSTYNVSTSTRTVMVEEFKQGLAVTDEILQGKSDWSKLLEPPNFFQKYRHYIVLTASASTEENHLEWVGLVESKIRVLVGNLERNEFITLAHVNPQSFPGNKEHHKDNNYVSMWFLGIIFRRVENAESVNIDLTYDIQSFTDTVYRQANNINMLKEGMKIEATHVKKKQLHHYLPAEILQKKKKQSLSDVNRSSGGLQSKRLSLDSSCLDSSRDTDNGTPFNSPASKSDSPSVGETERNSAEPAAVIVEKPLSVPPAQGLSIPVIGAKVDSTVKTVSPPTVCTIPTVVGRNVIPRITTPHNPAQGQPHLNGMSNITKTVTPKRSHSPSIDGTPKRLKDVEKFIRLESTFKDPRTAEERKRKSVDAIGGESMPIPTIDTSRKKRLPSKELPDSSSPVPANNIRVIKNSIRLTLNR

>gi|32490557|ref|NP_116021.2| poly(A) polymerase alpha isoform 1 [Homo sapiens]

MPFPVTTQGSQQTQPPQKHYGITSPISLAAPKETDCVLTQKLIETLKPFGVFEEEEELQRRILILGKLNNLVKEWIREISESKNLPQSVIENVGGKIFTFGSYRLGVHTKGADIDALCVAPRHVDRSDFFTSFYDKLKLQEEVKDLRAVEEAFVPVIKLCFDGIEIDILFARLALQTIPEDLDLRDDSLLKNLDIRCIRSLNGCRVTDEILHLVPNIDNFRLTLRAIKLWAKRHNIYSNILGFLGGVSWAMLVARTCQLYPNAIASTLVHKFFLVFSKWEWPNPVLLKQPEECNLNLPVWDPRVNPSDRYHLMPIITPAYPQQNSTYNVSVSTRMVMVEEFKQGLAITDEILLSKAEWSKLFEAPNFFQKYKHYIVLLASAPTEKQRLEWVGLVESKIRILVGSLEKNEFITLAHVNPQSFPAPKENPDKEEFRTMWVIGLVFKKTENSENLSVDLTYDIQSFTDTVYRQAINSKMFEVDMKIAAMHVKRKQLHQLLPNHVLQKKKKHSTEGVKLTALNDSSLDLSMDSDNSMSVPSPTSATKTSPLNSSGSSQGRNSPAPAVTAASVTNIQATEVSVPQVNSSESSGGTSSESIPQTATQPAISPPPKPTVSRVVSSTRLVNPPPRSSGNAATSGNAATKIPTPIVGVKRTSSPHKEESPKKTKTEEDETSEDANCLALSGHDKTEAKEQLDTETSTTQSETIQTAASLLASQKTSSTDLSDIPALPANPIPVIKNSIKLRLNR

>gi|77874206|ref|NP_064529.4| poly(A) polymerase beta [Homo sapiens]

MMPFPVTTQGPPQPAPPPNRYGVSSPISLAVPKETDCLLTQRLIETLRPFGVFEEEEELQRRILVLEKLNNLVKEWIREISESKSLPQSVIENVGGKIFTFGSYRLGVHTKGADIDALCVAPSHVDRSDFFTSFYAKLKLQEEVKDLRAVEEAFVPVIKLCFDGIEIDILFARLALQTIPEDLDLRDDSLLKNLDIRCIRSLNGCRVTDEILHLVPNIDNFRLTLRAIKLWAKCHNIYSNILGFLGGVSWAMLVARTCQLYPNAVASTLVRKFFLVFSEWEWPNPVLLKEPEERNLNLPVWDPRVNPSDRYHLMPIITPAYPQQNSTYNVSISTRMVMIEEFKQGLAITHEILLSKAEWSKLFEAPSFFQKYKHYIVLLASASTEKQHLEWVGLVESKIRILVGSLEKNEFITLAHVNPQSFPAPKENPDMEEFRTMWVIGLGLKKPDNSEILSIDLTYDIQSFTDTVYRQAVNSKMFEMGMKITAMHLRRKELHQLLPHHVLQDKKAHSTEGRRLTDLNDSSFDLSAGCENSMSVPSSTSTMKTGPLISSSQGRNSPALAVMTASVANIQATEFSLQQVNTNESSGVALNESIPHAVSQPAISPSPKAMVARVVSSTCLISHPDLQETQQQTYLIL

>gi|226494207|ref|NP_766143.2| poly(A) polymerase gamma [Mus musculus]

MKEMSANTMLDSQRQQKHYGITSPISLACPKEIDHIYTQKLIDAMKPFGVFEDEEELNHRLVVLGKLNNLVKEWISDISESKNLPPSVVATVGGKIFTFGSYRLGVHTKGADIDALCVAPRHVERSDFFQSFFEKLKHQDGIRNLRAVEDAFVPVIKFEFDGIEIDLVFARLAIQTISDNLDLRDDSRLRSLDIRCIRSLNGCRVTDEILHLVPNKETFRLTLRAVKLWAKRRGIYSNMLGFLGGVSWAMLVARTCQLYPNAAASTLVHKFFLVFSKWEWPNPVLLKQPEESNLNLPVWDPRVNPSDRYHLMPIITPAYPQQNSTYNVSTSTRTVMVEEFKQGLAVTDEILQGKSDWSKLLEPPNFFQKYRHYIVLTASASTEENHLEWVGLVESKIRVLVGNLERNEFITLAHVNPQSFPGNKEHHKANNYVSMWFLGIIFRRVENAESVNIDLTYDIQSFTDTVYRQANNINMLKDGMKIEATHVKKKQLHHYLPAEILQKKKKSLSDVSRSSGGLQSKRSSLDSTCLDSSRDTDSGTPFNSPVSANKPSNPDSPTGEIERSSAEPVAVVVEKLPSVPPAQGLSIPVIGAKVDPTAKAVSSPAVCTIPTVVGRNVIPRITTPHNPVQGQPHLNGISNITKNVTPKRSHSPPTDGTSKRLKDIEKFIRLESAFKESRAAEDRKRKPMDSIGGESMPIPTIDTARKKRLPSKELPDSSSPVPANNIRVIKNSIRLTLNR

>gi|9910588|ref|NP_064327.1| poly(A) polymerase beta [Mus musculus]

MMPFAVTTQGAQQPAPAPKQFGISSPISLAAPKDTDRELTQKLIETLQPFGVFEEEEELQRRILILQKLNNLVKEWIREISESRNLPQAVIENVGGKIFTFGSYRLGVHTKGADIDALCVAPRHVDRNDFFTSFYDKLKLQEEVKDLRAVEEAFVPVIKLCFDGIEIDILFARLALQTIPEDLDLRDDSLLKNLDIRCIRSLNGCRVTDEILHLVPNIDSFRLTLRAIKLWAKCHNIYSNILGFLGGVSWAMLVARTCQLYPNAIASTLVRKFFLVFSEWEWPNPVLLKEPEERNLNLPVWDPRVNPSDRYHLMPIITPAYPQQNSTYNVSVSTRMVMIEEFKQGLAITHEILLNKAEWSKLFEAPSFFQKYKHYIVLLASAPTEKQHLEWVGLVESKIRILVGSLEKNEFITLAHVNPQSFPAPKETADKEEFRTMWVIGLVLKKPENSEILSIDLTYDIQSFTDTVYRQAINSKMFEMDMKIAAMHLRRKELHQLLPNHVLQKKETHLTESVRLTAVTDSSLLLSIDSENSMTAPSPTGTMKTGPLTGNPQGRNSPALAVMAASVTNIQFPDVSLQHVNPIESSGIALSESIPQIPSQPTISPPPKPTMTRVVSSTHLVNHPSRPSGNTATNIPNPILGV

>gi|21914853|ref|NP_035242.1| poly(A) polymerase alpha [Mus musculus]

MPFPVTTQGSQQTQPPQRHYGITSPISLAAPKETDCLLTQKLIETLKPFGVFEEEEELQRRILILGKLNNLVKEWIREISESKNLPQSVIENVGGKIFTFGSYRLGVHTKGADIDALCVAPRHVDRSDFFTSFYDKLKLQEEVKDLRAVEEAFVPVIKLCFDGIEIDILFARLALQTIPEDLDLRDDSLLKNLDIRCIRSLNGCRVTDEILHLVPNIDNFRLTLRAIKLWAKRHNIYSNILGFLGGVSWAMLVARTCQLYPNAIASTLVHKFFLVFSKWEWPNPVLLKQPEECNLNLPVWDPRVNPSDRYHLMPIITPAYPQQNSTYNVSVSTRMVMVEEFKQGLAITDEILLSKAEWSKLFEAPNFFQKYKHYIVLLASAPTEKQRLEWVGLVESKIRILVGSLEKNEFITLAHVNPQSFPAPKESPDREEFRTMWVIGLVFKKTENSENLSVDLTYDIQSFTDTVYRQAINSKMFELDMKIAAMHVKRKQLHQLLPSHVLQKRKKHSTEGVKLTALNDSSLDLSMDSDNSMSVPSPTSAMKTSPLNSSGSSQGRNSPAPAVTAASVTSIQASEVSVPQANSSESPGGPSSESIPQTATQPAISPPPKPTVSRVVSSTRLVNPSPRPSGNTATKVPNPIVGVKRTSSPNKEESPKKTKTEEDETSEDANCLALSGHDKTETKEQVDLETSAVQSETVPASASLLASQKTSSTDLSDIPALPANPIPVIKNSIKLRLNR

>gi|17985991|ref|NP_536790.1| hiiragi, isoform A [Drosophila melanogaster]

MWNSEPTHRQHHQHNGNSTSGGPPAKQLGMTSAISLAEPRPEDLQRTDELRGSLEPYNVFESQDELNHRMEILAKLNTLVKQWVKEISVSKNMPESAAEKLGGKIYTFGSYRLGVHHKGADIDALCVAPRNIERTDYFQSFFEVLKKQPEVTECRSVEEAFVPVIKMNFDGIEIDLLFARLSLKEIPDDFDLRDDNLLRNLDHRSVRSLNGCRVTDEILALVPNIENFRLALRTIKLWAKKHGIYSNSLGYFGGVTWAMLVARTCQLYPNAAAATLVHKFFLVFSRWKWPNPVLLKHPDNVNLRFQVWDPRVNASDRYHLMPIITPAYPQQNSTFNVSESTKKVILTEFNRGMNITDEIMLGRIPWERLFEAPSFFYRYRHFIVLLVNSQTADDHLEWCGLVESKVRLLIGNLERNPHIALAHVNPKCFEFKKGQSANNSQNNSGNEDDLKQSQGNQSAVTSAPFCSMWFIGLEFERSENLNVDLTESIQNFTEHVMMHGVNIKMLKEGMTIDARHVKRKQLSLYLDSDFLKRERKSMESHNNFNNTLLANRKRLSTELAQSQDPLPPGQQPSSGNRGRDSGAKIQRLSDSLTEENSNASSDMGAGTPTTPTTAQLSAPSFKSSGKNGSEIDVVEQEPTQPHNNGNASSNTTTTEVACS

>gi|17565368|ref|NP_505683.1| Poly-A Polymerase family member (pap-1) [Caenorhabditis elegans]

MSATEKDKTPLLGVSQPISLAHPDSKDIAQTTLLIETLKKFGSYEPKEETEQRMEVLRNLNRLVKEWVKNVTAMKIPNGEGVNAGGKLFTFGSYRLGVHSSGADIDTLAVVPRHIDRSDFFTSFKEMLNNDPNVTELHGVEEAFVPVMKLKYSGVELDILFARLALKEVPDTQELSDDNLLRNLDQESVRSLNGCRVAEQLLKLVPRQKEFCVTLRAIKLWAKNHGIYSNSMGFFGGITWAILVARACQLYPNASPSRLVHRMFFIFSTWTWPHPVVLNEMNNDRNDIPTLCELVWDPRRKNTDRFHVMPIITPAFPEQNSTHNVTRSTATVIKNEICEALEICRDISEGKSKWTALFEEVNFFSRYKHFIALIMAAPNEEEELNYGGFLESRIRLLVQSLERNQDIIIAHNDPNKHKPSPNAKFDVNPENKRVTVWFIGLEFAEHAKTLDLTNEIQRFKTNVELQASNVKGIGPNCQVQIDMFYVKRNSLIQVISAADLRRGRRWKKVVPIATNTSVSSSTPRSVVRTTSTSSVPTTPTGLAAPKTPLSASVSATNEPDSTTNGTPLSRKRSMDEESSTTVTSQISDESVPKKKTRDDTLEENRVSMVVEVSNVVVEQRTKVVQEIVDLQADNGLNTSNGLEASEQKMEVPQSV

>gi|17536297|ref|NP_496067.1| hypothetical protein T15H9.6 [Caenorhabditis elegans]

MQSVNNATSSTPIFGVSQPISTAFPKANDVALTSSLQSSLEEFKSFESKQETLLRIKVLKNLDGLVKDWVQKTTISRIPKDQMFNAGGKLIPFGSYRLGVHSSGADIDSIVVAPRHITRSDFFNSFKDILAKNPEVTELCAVEKAFVPIMTLKYSGVDIDILFARLALKSVPEDLNILDDSLLKNLDEESVRSLNGCRVAEQLLKLVPHQKNFCITLRAIKLWAKNHGIYSNSMGFFGGITWAILVARICQLYPNAAPSRLIQKVFFIFSTWNWPAPVLLDYINCDRTDLAQLNQLVWDPRRNHADRYHLMPIITPAFPQQNSTHNVSRSSMKVIQDEMKKALIICDKIHEGTLEWRDLLEEINFFSKYHHFIALKLKAESVKEELAFGGFFESRIRQLVQILEKNQVIQVAQINPRKFKDVKDPKKSIWFIGLEFVANVKNVDLTSDIQQFKMNIDRQSMSVKEIAAGCQVETDFTYEKRSNLINIISKKDLSCQRIMKKTEVSVPVTKDSRNLKRHILDEAPTVQKKHKLDVLEKSTVDSLLSTLKKNTSHA

>gi|17533995|ref|NP_496524.1| hypothetical protein F43G6.5 [Caenorhabditis elegans]

MTSVNTLSVNITVPITRTFGVSQPISTALPNAKDLELTSSLQKTLNQYNSYEPQEETSQRVKVLQKLNGCVKQWIQKITEARYPKDQLFNAGGQLMAFGSYRLGVHSSGADIDTLVLAPRHATRADFFSSFKQMLAKDPEVTNLCAVENAFVPIMTMKYSGIEIDFLFARLALKEVPEDLDILDDALLKNLDQESVRSLNGCRVAENLLKLVPNQENFCITLRAIKLWAKNHGIYSNSMGFFGGITWAILVARACQLYPNASPSKLIQKVFFIFSTWKWPAPVLLDFISSDRMDLAQLNQLVWDPRRNQSDRYHLMPIVTPAFPQQNSTHNVSRSSMKVIQEEMKDALITCDNIQNGSCNWMDLLEEINFFSRYKHFISLSMTAETEKDELAFGGFFESRIRQLVLILEKNQGIKLAHINPKKFKAAKDPKKSVWFIGLQFDENVKNLDLTKDIQQFKRNIDYQAKSVKDIEANCAVETDFSYVKRSDLAQTISVKDLKQGKMYKKPESKDPVSKKRKVSENAPSVPKKQKLDVSEKSSVESLLKNLKKKSLIV

>gi|6322854|ref|NP_012927.1| Pap1p [Saccharomyces cerevisiae S288c]

MSSQKVFGITGPVSTVGATAAENKLNDSLIQELKKEGSFETEQETANRVQVLKILQELAQRFVYEVSKKKNMSDGMARDAGGKIFTYGSYRLGVHGPGSDIDTLVVVPKHVTREDFFTVFDSLLRERKELDEIAPVPDAFVPIIKIKFSGISIDLICARLDQPQVPLSLTLSDKNLLRNLDEKDLRALNGTRVTDEILELVPKPNVFRIALRAIKLWAQRRAVYANIFGFPGGVAWAMLVARICQLYPNACSAVILNRFFIILSEWNWPQPVILKPIEDGPLQVRVWNPKIYAQDRSHRMPVITPAYPSMCATHNITESTKKVILQEFVRGVQITNDIFSNKKSWANLFEKNDFFFRYKFYLEITAYTRGSDEQHLKWSGLVESKVRLLVMKLEVLAGIKIAHPFTKPFESSYCCPTEDDYEMIQDKYGSHKTETALNALKLVTDENKEEESIKDAPKAYLSTMYIGLDFNIENKKEKVDIHIPCTEFVNLCRSFNEDYGDHKVFNLALRFVKGYDLPDEVFDENEKRPSKKSKRKNLDARHETVKRSKSDAASGDNINGTTAAVDVN

Figure S11.

>Sbi:Sb01g028780

MAMTSKRGRSGDETRGSGHRARIGRIEEGETDHPSAVELRASLYTRDQEIEEMRRRLRELEKLEFEIPPAPSHEEESETAVAATTVDKAEVDVRSIYVGNVDYACSPEEVQQHFQFCGTINRVTILTDSFGQPKGFAYVEFDEVEAVQNALLLNETELHGRPLKVCPKRTNIPGMKQSRGRHSVYPFYPSYGKVPRFRRFLGYSYSPYY

>Sbi:Sb04g027340

MDEEEHEVYGQEIPEDGDMDGADVDMAAAGDDATKLQELDEMKRRLKEMEEEAAALRDMQAKVAKEMQGGDPSASTAEAKEQVDARSVYVGNVDYACTPEEVQQHFQACGTVNRVTILTDKFGQPKGFAYVEFLEQEAVQEAINLNESELHGRQIKVAPKRTNVPGMKQRPPRGYNPYHGYPYRSYGAPYFPPYGYGRAPRFRRPMRYRPYF

>Sbi:Sb10g007620

MEDEEHEVYGQEIPVDGEDVDMAAGDDATKLQELDEMKRRLKEMEEEAAALREMQAKVAKEMQGVDPNATTSENKEEMDSRSVFVGNVDYACTPEEVQQHFNSCGTVNRVTILTDKFGQPKGFAYVEFVEVEAVQEAVKLNESELHGRQLKVAPKRTNVPGMKQPRGRGFNPYHGHPYMRPYGYSPYGYGRFPRFRRPRRPYF

>Smo:102545

MEATHGVEDEHEVYGGEIPEEVEGDLEGDQGDDAGGDDPTSTELEEMKKRLKEMEDEAAALRDMQAKVEKEMGAGQDPAAAAQQASKEEADARSVYVGNVDYSCTPEEVQQHFQSCGTVNRVTILTDKFGQPKGFAYVEFLEVEAVQNALILNESELHGRPIKARISRTNVPGLKQHRGRGGFGAPYMPYFPRRPYMPYPSYPYPYGFGKVPRFRRPMRYRPYY

>Smo:74095

MEAAAAIDEEHEVYGAEIPDEVEEEAHEKAEGPARDDGSAKFKELEDMKKRLKEMEDEAAALRDMQAKVEKEMGAGQDANAASQAGKEEADARSVYVGNVDYSCTPEEVQQHFQSCGTVNRVTILTDKFGQPKGYAYVEFLEGEAVQNAILLNESELHGRPLKVSAKRTNVPGMKQYRGRRFDPSYAYMTRRAYMPPYAYAPYGFGKIPRFRRAMRYRPYF

>Aly:487865

MEEEEHEVYGGEIPEVEETDVPDPDIDMSAGDDDAVTELAEMKKRLKEMEEEAAALREMQAKVEKEMGATQDPATMAANQEGKEEVDARSVYVGNVDYACTPEEVQLHFQTCGTVNRVTILMDKFGQPKGFAYVEFVEVEAVQEALQLNESELHGRQLKVSPKRTNVPGMKQYHPGRFNPSIGYRFRRPFVPPYFYSPYGYG

>Aly:919857

MEEEEHEVYGGEIPDVGEMDGDMEALNPDLDMAAADDDAVKELDEMKKRLKEMEDEAAALREMQAKVEKEMGAQDPASIAANQAGKEEVDARSVFVGNVDYACTPEEVQQHFQTCGTVHRVTILTDKFGQPKGFAYVEFVEVEAVQEALQLNESELHGRQLKVLQKRTNVPGLKQFRGRRFNPYMGYRFRRPFMPPYMYSPYGYGKAPRFRRPMRYMPYQ

>Gma:Glyma05g31030

MEEEEHEVYGGEIPDEGEMEGDIDMSAADDDAAVKELDEMKRRLKEMEEEAAALREMQAKVDKEIGSVQDPANSAASQANKEEADSRSVFVGNVDYACTPEEVQQHFQSCGTVNRVTILTDKFGQPKGFAYVEFVEAEAVQEALLLNESELHGRQLKVLPKRTNVPGMKQYRPRRFNPYMAYGFRRPYTPPYFYSPYGYGKVPRFRRPNRYMPYH

>Gma:Glyma08g14240

MEEEEHEVYGGEIPDEGEMEGDIDMSAADDEAAVDDDAAVKELDEMKRRLKEMEEEAAALREMQAKVEKEIGSVQDPANAAASQANKEEADARSVFVGNVDYACTPEEVQQHFQSCGTVNRVTILTDKFGQPKGFAYVEFVEAEAVQEALLLNESELHGRQLKVLPKRTNVPGMKQYRPRCFNPYMAYGFRRPYTPYLYSPYGYGKVPRFRRPNRYMPYY

>Gma:Glyma08g18970

MENDDVDMHAADTNEELDDMKKRLKEMEDEAAALREMQAKVEKEMGSVQDPANASASQANKEEIDSRSVFVGNVDYSCTPEEVQQHFQSCGTVNRITIRTDKFGQPKGYAYVEFLEVEAVQEALLLNESELHGRQLKVTAKRTNIPGMKQYRPRRSTNPYMGGLRGRTPYAAPFIYSPYGYGKVPRFRMAMRHSPYY

>Gma:Glyma15g06030

MENDDVDMHAADTSEELDDMKKRLKEMEDEAAALREMQAKVEKEMGSAQDPANASASQANKEEIDSRSVFVGNVDYSCTPEEVQQHFQSCGTVNRVTIRTDKFGQPKGYAYVEFLEVEAVQEALLLNESELHGRQLKVTAKRTNIPGMKQYRPRRTINPYMGGFRGRTPYAPPFIYSPYGYGKVPRFRMAMRYSPYY

>Gma:Glyma20g34100

MEQEEHEVYGADIPDEEIDMDADAEQQDEQLAPNHTNKELEDMKKRLKEIEEEASALREMQAKVEKEMGAVQDSSGTSATQVEKEEVDARSIYVGNVDYACTPEEVQQHFQSCGTVNRVTILTDKFGQPKGFAYVEFVEIDAVQNALLLNESELHGRQLKVSAKRTNVPGMKQYFGRRPAGFRSRRPFMPAPFFPPYGYGRVPRYRRPTRYRPY

>:GSVIVT01004460001

MNDCCHLQVMWQKTLNVEKLIHFVDYACTPEEVQQHFQSCGTVNRVTILTDKYGQPKGFAYVEFLEVEAVQEALLLNESELHGRQLKVLAKRTNVPGMKQFRPRRFNPYMGYRFRRPYVPPYFYSPYGYGKAPRFRRPMRYMPYY

>:GSVIVT01015755001

MGGPESHDEHQIEDLNQRLEQMMEEKPKAHNDMPSVQDPAAAAAAAAQASREEVDSRSVFVGNVDYSCTPEEVQQHFQACGTVNRVTIRSNKYGQPKGYAYVEFLETEAVQEALLLNESELHGRQLKVSAKRTNVPGLKQFRPRRVGFRSRSTHMPPYLCPYGYGKVPRFRMPMRYNPYY

>Ppa:Pp1s433_16V6.2

MEEEHEVYGGDIPEEVEGDIEGDLDGEPDDLVLKGDDTTTDEAASKELEEMKKRLKEMEEEAAALRDMQAKVEKEMGAVQDSVGKDGSATNREEADARSVFVGNVDYSCTPEEVQQHFQSCGTVNRVTILTDKFGQPKGFAYVEFLEVEAVQNAILLSESELHNRPLKVTAKRTNVPGMKAYRGRPYYGYRSRRPYPPPYGKVPRFRRPMRYRPYY

>Ppa:Pp1s3_616V6.1

MEEEHEVYGGDIPDEVEGELEGDSDGDCEDLLLKGDIGIDEAASKELEEMKKRLKEMEEEAAALRDMQAKVEKEMGGSQDSAITSGAQANREEADARSVFVGNVDYSCTPEEVQQHFQSCGTVNRVTILTDKFGQPKGFAYVEFLEVEAVQNAILLSESELHNRPLKVTSKRTNVPGMKGFRGRPYAPYYGYRPRRPFSAPYGKVPRFRRPMRYRPYY

>Pp1s85_168V6.2

MEEEHEVYGGDIPDEVEGELEVDSDGDGEDLLLKGEDVGTDEVASKELEEMKKRLKEMEEEAAALRDMQAKVEKEMGGSQDSALTNGDQANREEADARSVFVGNVDYACTPEEVQQHFQSCGTVNRVTILTDKFGSPKGYAYVEFLEVEAVQNAILLSESELHNRPIKVTAKRTNVPGMKGYRGRPYAPYYGYRPRRPFSAPYGKVPRFRRPMRYRPYY

>Ptr:POPTR_0005s07440

MEEEEHEVYGGEIPDVEGDMDPHNADVDMSAADDDAVKELDEMKKRLKEMEEEAAALREMQAKVEKEMGAVQDPATAAANQANREEADSRSVFVGNVDYACTPEEVQQHFQSCGTVNRVTILTDKFGQPKGFAYVEFLEVEAVQEALALNESELHGRQLKVSPKRTNVPGMKQYRPRRINPYMGYRFRRPYAPPYFYSPYGYGKVPRFRRSMRYMPYY

>Ptr:POPTR_0017s07850

MDGDDVDMAAVETAEAVPELDDMKKRLKEMEDEAAALREMQAKVEKEMGSVQDPSASATASQANKEEVDSRSVFVGNVDYACTPEEVQQHFQACGTVNRVTIRSDKYGQPKGYAYVEFVEPEAVQEALLLNESELHGRQLKVTAKRTNVPGMKQFRARRPSPYMGFPPRAAIMPPYLFSPYGYGKVMRYRMPMRYSPYG

>Ptr:POPTR_0015s12230

MDQLHDHEEDEHEHEVYGGEIPDEGEMDADADMSSRAEEDEYQDPNSKDLEDMKRRLKEIEEEAGALREMQAKVEKEMGAVQDSPGASATQAEKEEVDSRSIYVGNVDYSCTPEEVQQHFQSCGTVNRVTILTDKFGQPKGFAYVEFIEVDAVQNAVLLNESELHGRQLKVSAKRTNVPGMKQYRGRRPSPYGFRSQRAFMPAPFYPAYGYGRVPRFRRPMRYRPY

>Ptr:POPTR_0001s31900

MTCRCISFFASERINCGEIGSVNYILASRIVCRSHSSPRELEKEGGRMDGDDVDMAAAETNESVPELDDMKKRLKEMEDEAAALLEMQAKVEKEMGSVQDPSASAAASQANREEVDSRSVFVGNVDYSCTPEEVQQHFQACGTINRITIRSDKYGQPKGYAYVEFLEPETVQEALLLNESELHGRQLKVTVKRTNLPGMKQFRARRPNPYMGFPPRGAPMPPYLFSPYGYGKVLRYRMPMRYSPYG

>Ptr:POPTR_0007s05140

MEEEEHEVYGGEIPDVEGDMDPHNADVDMSAAEDDAVKELDEMKKRLKEMEEEAAALREMQAKVEKEMGAVPDPASAAANQANKEEADSRSVFVGNVDYACTPEEVQQHFQSCGTVNRVTILTDKFGQPKGFAYVEFVEVEAVQEALALNESELHGRQLKVSPKRTNVPGMKQYHPRSFNPYMGNRFRRPYAPPYFYSPYGYGKVPRFRRSMRYMPYY

>Ath:AT5G65260

MEEEEHEVYGGEIPDVGEMDGDMEALNPDLDMAAADDDAVKELDEMKKRLKEMEDEAAALREMQAKVEKEMGAQDPASIAANQAGKEEVDARSVFVGNVDYACTPEEVQQHFQTCGTVHRVTILTDKFGQPKGFAYVEFVEVEAVQEALQLNESELHGRQLKVLQKRTNVPGLKQFRGRRFNPYMGYRFRRPFMSPYMYSPYGYGKAPRFRRPMRYMPYQ

>Ath:AT5G10350

MEEEEHEVYGGEIPEVGDTDVPDPDIDMSAADEDAVTELAEMKRRLKEMEEEAAALREMQAKVEKEMGATQDPASMAANQEGKEEVDARSVYVGNVDYACTPEEVQLHFQTCGTVNRVTILMDKFGQPKGFAYVEFVEVEAVQEALQLNESELHGRQLKVSPKRTNVPGMKQYHPGRFNPSMGYRFRRPFVPPYFYSPYGYGKAPRFRRPMRYMPYQ

>Cre:Cre06.g274500.t1.1

MADEELYGEEVGEGEEMGDVGEGEEMGEDLEQGVEELDALKRKLKEMEEEAARVKALSGGAPDAAAAGTSGAAGGAAGAAAAAAQAQTEAEKAEVDSRSIFVGNVDYGCTPEELQQHFASCGTVNRVTILTDKFGNPKAFAYVEFLEVDAVNNAVLLDNSELRGRQIKVSHKRTNVPGLKAGRGRGRGRGPPGGGFYGAPAPFYGGRGGGGYGYGAPPAYRGGRGGGYHGGAPGGFAPRGRGRGRFYAPY

>Bdi:Bradi1g45640

MEDEEHEVYGQEIPVDGEDVDMGAAGDDAAKLHELDEMKRRLKEMEEEAAALREMQAKVAKEMQGGDPNATTSEAKEEMDSRSVFVGNVDYACTPEEVQQHFNSCGTVNRVTILTDKFGQPKGFAYVEFVEAEAIQEAVKLSESELHGRQIKVAPKRTNVPGLKQPRGGRGYNPYGGHPYMRPYGAPFFNPYGGYGRVPRFRRPRRPFY

>Bdi:Bradi3g58600

MDEEEHEVYGQEIPVDGDMEGGDVDMTTGGDDAAKELDEMKRRLKEMEEEAAALRDMQAKVAKEMQGGDANASTAEAKEQVDARSVYVGNVDYACTPEEVQQHFQACGTVNRVTILTDKFGQPKGFAYVEFLEQEAVQEALNLNESELHGRQIKVSPKRTNVPGMKQRPPRGFNPYHGYPYRSYGAQYFPPYGYGRVPRFRRPTRYRPYF

>Bdi:Bradi3g34110

MDSNGNSDDRSLNPDAGDIVLGIGSSRSRIIDQEIEEMQRKLRELGEEIDLAIVSLNEQIEMTTDQISGVSAEEEEDPAVAIARHKSEVDSRSIYVGNVDYACLPEQIQAHFQDCGAINRVTILIDDFGDPKGYAYVEFAEVEAVEKALLMNDTKLLNRPMKVSPKRTNVPGRTHPWGRRPFQPYPTYGKFPRFRRPQGYSPYY

>Osa:LOC_Os02g52140

MDEEEHEVYGQEIPEDGDMDGADVDMASGGDDAAKLQELDQMKRRLKEMEEEAAALRDMQAKVAKEMQGGPPGGDPSASTAEAKEQVDARSVYVGNVDYACTPEEVQQHFQACGTVNRVTILTDKFGQPKGFAYVEFLEQEAVQEALNLNESELHGRQIKVAPKRTNVPGMKQRPPRGYNPYHGYPYRSYGAPYFPPYGYGRVPRFRRPMRYRPYF

>Osa:LOC_Os06g11620

MADEEHEVYGQEIPLDGEDVDMGAPGDEAAKMQELDEMKRRLKEMEEEANALREMQTKVAKEMQGLDPNASSSESKEEMDARSVYVGNVDYACTPEEVQQHFNSCGTVNRVTILTDKFGQPKGFAYVEFLEVEAVQEAVKLNESELHGRQIKVAPKRTNVPGMKQPRGGRGFGGHPYMRPYGAPFYNPYGYGYPRFRRPRRPYF

>Aly:918145

MPVHEEQEHEVYGGEISEEEEEEGEMDTEEYEEHGGEEGAAAGDEELEPGSSSKDLEDMKKRIKEIEEEAGALREMQAKAEKDMGAGQDPSGGVSAAEKEEVDSRSIYVGNVDYACTPEEVQQHFQSCGTVNRVTILTDKFGQPKGFAYVEFVEVEAVQNSLILNESELHGRQIKVSAKRTNVPGMRQFRGRRPFRPMRGFMPGVPFYPPYAYGRVPRFRRPMRYRPY

>Gma:Glyma10g33490

MEQEEHEVYGADIPDEEIDMDADAEQQDEQLAPNHTNKELEDMKKRLKEIEEEASALREMQAKVEKEMGAVQDSSGTSATQAEKEEVDARSIYVGNVDYACTPEEVQQHFQSCGTVNRVTILTDKFGQPKGFAYVEFVEIDAVQNALLLNESELHGRQLKVSAKRTNVPGMKQYFGRRPAGFRSRRPFMSAPFFPPYGYGRVPRYRRPTRYRPY

>Ppa:Pp1s422_18V6.1

MEEEHEVYGGDIPVEVEGDIEGDLDGEPDDLLLKGDDTATDEAASKELEEMKKRLKEMEEEAAALRDMQAKVEKEMSAVQDSVGKDGSPANREEADARSVFVGNVDYSCTPEEVQQHFQSCGTVNRVTILTDKFGQPKGFAYVEFLEVEAVQNAILLNESELHNRQLKVTAKRTNVPGMKAYRGRPYAPFYGYRPRRPYPPLYGKMPRFRRPMRYRPYY

>Ptr:POPTR_0012s11480.1

MDHLHDHEEDEHEHEVYGGEIPDEGEMDADVDMSSRAEEDEYQDPNSKDLEDMKKRLKEIEEEAGALREMQAKVEKEMGAVQDSPGASATQAEKEEVDSRSIYVGNVDYSCTPEEVQQHFQSCGTVNRVTILTDKFGQPKGFAYVEFVEVDAIQNALLLNESELHGRQLKVSAKRTNVPGMKQFRGRRPSPYGLRSRRPFMPAPFYPAYGYGRVPRFRRPMRYRPY

>Ath:AT5G51120

MPVHDEQEHEVYGGEIPEEEEGEMDTEEYEEHGGEEGAAAGDEELEPGSSSRDLEDMKKRIKEIEEEAGALREMQAKAEKDMGASQGLKLVLLIMILLWVYILESFSESMNLSRNLLEFEYEKEIINSGVSAAEKEEVDSRSIYVGNVDYACTPEEVQQHFQSCGTVNRVTILTDKFGQPKGFAYVEFVEVEAVQNSLILNESELHGRQIKVSAKRTNVPGMRQFRGRGRPFRPMRGFMPGVPFYPPYAYGRVPRFRRPMRYRPY

>gi|4758876|[Homo sapiens]

MAAAAAAAAAAGAAGGRGSGPGRRRHLVPGAGGEAGEGAPGGAGDYGNGLESEELEPEELLLEPEPEPEPEEEPPRPRAPPGAPGPGPGSGAPGSQEEEEEPGLVEGDPGDGAIEDPELEAIKARVREMEEEAEKLKELQNEVEKQMNMSPPPGNAGPVIMSIEEKMEADARSIYVGNVDYGATAEELEAHFHGCGSVNRVTILCDKFSGHPKGFAYIEFSDKESVRTSLALDESLFRGRQIKVIPKRTNRPGISTTDRGFPRARYRARTTNYNSSRSRFYSGFNSRPRGRVYRGRARATSWYSPY

>gi|122937418|[Homo sapiens]

MWPFPSRSLFPPPTQAWLQTVSSDPEAQGWGAWNETKEILGPEGGEGKEEKEEEEDAEEDQDGDAGFLLSLLEQENLAECPLPDQELEAIKMKVCAMEQAEGTPRPPGVQQQAEEEEGTAAGQLLSPETVGCPLSGTPEEKVEADHRSVYVGNVDYGGSAEELEAHFSRCGEVHRVTILCDKFSGHPKGYAYIEFATKGSVQAAVELDQSLFRGRVIKVLPKRTNFPGISSTDRGGLRGHPGSRGAPFPHSGLQGRPRLRPQGQNRTPAALTPLWARPLPWVVVSHVIK

>gi|9506945|[Mus musculus]

MAAAAAAAAAAGAAGGRGSGPGRRRHLVPGAGGEAGEGDPGGAGDYGNGLESEELEPGELLPEPEPEEEPPRPRAPPGAPGPGPGSGAPGSQEEEEEPGLVEADPGDGAIEDPELEAIKARVREMEEEAEKLKELQNEVEKQMNMSPPPGNAGPVIMSLEEKMEADARSIYVGNVDYGATAEELEAHFHGCGSVNRVTILCDKFSGHPKGFAYIEFSDKESVRTSLALDESLFRGRQIKVIPKRTNRPGISTTDRGFPRSRYRARTTNYNSSRSRFYSGFNSRPRGRIYRGRARATSWYSPY

>gi|149259131|[Mus musculus]

MEPYLSNELFPPPTEAWLQTVSSDPEAQGWGAWGRTEKTSLVPRAGSRAGSDKEAEENEDASFLLSLLEPENLAKSPVFNQELEAIKLKLWAMEHAEAQPEPPCVQRKATEEERAEVRQLLSPETVDCFFSRTSKENVEADHRSVFVGNVDYGGSAAELEAYFSPCGEIHRVTILCDKFSGHPKGYAYIEFASHRSVKAAVGLDESTFRGRVIKVLPKRTNFPGISSTDRGGLRTHSGSRAAFLHGSLHRKARLRAHGRSRGHGGAPQWFSPY

>gi|17136784|[Drosophila melanogaster]

MADEDITLNEDQLLESLEETNGEQETEIATEVEEEGSMQIDPELEAIKARVKEMEEEAEKIKQMQSEVDKQMAGGSTTGLATVPLSLEEKQEIDTRSVYVGNVDYGASAEELEAHFHGCGTINRVTILCNKADGHPKGFAYIEFGSKEFVETALAMNETLFRGRQIKVMSKRTNRPGLSTTNRFARGSFRGRGARVSRACCHSTFRGARRAMGYRGRANYYAPY

>gi|17505625|[Caenorhabditis elegans]

MSDNDIIDDDVLNIEDMTGDDADLSAIEGDLNEIEEEQKKLKAIQNEMVGHMNLNTSSQSNSSQSLLTPEEKAEADAKSVYVGNVDYGATAEEIEQHFHGCGSVSRVTIQCDRFSGHPKGFAYVEFTEKEGMQNALAMTDSLLRGRQIKVDPKRTNKPGMSTTNRPPFRGRGGRGRGNVIVKYVYAGGFRPRGRGARRRPGFAPY
